# In Vitro Antioxidant and In Vivo Antigenotoxic Features of a Series of 61 Essential Oils and Quantitative Composition–Activity Relationships Modeled through Machine Learning Algorithms

**DOI:** 10.3390/antiox12101815

**Published:** 2023-09-29

**Authors:** Milan Mladenović, Roberta Astolfi, Nevena Tomašević, Sanja Matić, Mijat Božović, Filippo Sapienza, Rino Ragno

**Affiliations:** 1 Kragujevac Center for Computational Biochemistry, Department of Chemistry, Faculty of Science, University of Kragujevac, Radoja Domanovića 12, P.O. Box 60, 34000 Kragujevac, Serbia; nevena.tomasevic@pmf.kg.ac.rs; 2 Rome Center for Molecular Design, Department of Drug Chemistry and Technology, Faculty of Pharmacy and Medicine, Rome Sapienza University, P. le A. Moro 5, 00185 Rome, Italy; roberta.astolfi@uniroma1.it (R.A.); filippo.sapienza@uniroma1.it (F.S.); 3 Department of Science, Institute for Information Technologies Kragujevac, University of Kragujevac, Jovana Cvijića bb, 34000 Kragujevac, Serbia; sanjamatic@kg.ac.rs; 4 Faculty of Science and Mathematics, University of Montenegro, Džordža Vašingtona bb, 81000 Podgorica, Montenegro; mijatboz@ucg.ac.me

**Keywords:** essential oils, antioxidant and antigenotoxic features in vitro and in vivo, machine learning

## Abstract

The antioxidant activity of essential oils (EOs) is an important and frequently studied property, yet it is not sufficiently understood in terms of the contribution of EOs mixtures’ constituents and biological properties. In this study, a series of 61 commercial EOs were first evaluated as antioxidants in vitro, following as closely as possible the cellular pathways of reactive oxygen species (ROS) generation. Hence, EOs were assessed for the ability either to chelate metal ions, thus interfering with ROS generation within the respiratory chain, or to neutralize 2,2-diphenyl-1-picrylhydrazyl (DPPH^•^) and lipid peroxide radicals (LOO^•^), thereby halting lipid peroxidation, as well as to neutralize 2,2′-azino-bis(3-ethylbenzothiazoline-6-sulfonic acid cation radicals (ABTS^•+^) and hydroxyl radicals (OH^•^), thereby preventing the ROS species from damaging DNA nucleotides. Showing noteworthy potencies to neutralize all of the radicals at the ng/mL level, the active EOs were also characterized as protectors of DNA double strands from damage induced by peroxyl radicals (ROO^•^), emerging from 2,2′-azobis-2-methyl-propanimidamide (AAPH) as a source, and OH^•^, indicating some genome protectivity and antigenotoxicity effectiveness in vitro. The chemical compositions of the EOs associated with the obtained activities were then analyzed by means of machine learning (ML) classification algorithms to generate quantitative composition–activity relationships (QCARs) models (models published in the AI4EssOil database available online). The QCARs models enabled us to highlight the key features (EOSs’ chemical compounds) for exerting the redox potencies and to define the partial dependencies of the features, viz. percentages in the mixture required to exert a given potency. The ML-based models explained either the positive or negative contribution of the most important chemical components: limonene, linalool, carvacrol, eucalyptol, α-pinene, thymol, caryophyllene, *p*-cymene, eugenol, and chrysanthone. Finally, the most potent EOs in vitro, Ylang-ylang (*Cananga odorata* (Lam.)) and Ceylon cinnamon peel (*Cinnamomum verum* J. Presl), were promptly administered in vivo to evaluate the rescue ability against redox damage caused by CCl_4_, thereby verifying their antioxidant and antigenotoxic properties either in the liver or in the kidney.

## 1. Introduction

Essential oils (EOs) are liquid mixtures of volatile compounds extracted from aromatic plants, usually by hydro-distillation and steam distillation [1], but also through a suitable mechanical process without heating [2]. Their medicinal potential is traditionally associated with antimicrobial activity or antiviral properties [3]. Although there is a scarce understanding of their mechanism of action, considerable attention has been raised regarding the antioxidant activity of EOs due to the presence of aliphatic compounds (hydrocarbons and their oxygenated derivatives), terpenes (hydrocarbons and oxygenated derivatives), phenols and phenol derivatives, *O*- and *O*,S-heterocycles, as well as *N*- and *N*,*S*-heterocycles, that contribute to free radical scavenging activity [1,4]. As oxidative stress reducers, EOs might contribute to preventing the pathogenesis of aging and degenerative diseases, such as atherosclerosis, cardiovascular diseases, diabetes, and cancer [5]. Similarly, equally important redox-potent EOs could preserve processed food enriched with fats and oils and prevent spoilage and quality deterioration, thus representing a replacement for common synthetic antioxidants (such as butylated hydroxyanisole (BHA) or butylhydroxytoluene (BHT)) that are suspected to be potentially harmful to human health [6].

Reactive oxygen species (ROS), including superoxide anion radical [O_2_^•−^], hydroperoxyl radical [HOO^•^], and hydroxyl radical [OH^•^]), reactive lipid species (viz. lipid peroxyl radical [LOO^•^]), reactive nitrogen species (RNS), such as nitric oxide radical [NO^•^], and non-free radicals, like hydrogen peroxide (H_2_O_2_) and peroxynitrite (ONOO), are in vivo oxidative stress inducers by interacting with cellular biomolecules (proteins, lipids, DNA, and carbohydrates) [7]. Therefore, in vitro and in vivo methods are used to screen EOs as antioxidants [8]. EOs can be considered preventive antioxidants as they can retard the initial formation of radical species within the Fenton-type [9] and Haber–Weiss reactions [10] by chelating the redox active metal ions (e.g., Fe^2+^) [1]. In addition, EOs could be investigated as either chain-breaking antioxidants or termination-enhancing antioxidants for their ability to slow (or block) autoxidation by competing with propagation reactions (i.e., reacting with peroxyl radicals faster than the oxidizable substrate to form species that do not propagate the oxidation chain) [1]. To stop the propagation of peroxyl radicals, EOs could either scavenge them or neutralize them by hydrogen donation [1].

In this report, a series of 61 commercial EOs were extensively evaluated as potential antioxidants agents through the determination of their total antioxidant capacity (TAC) [11], their ability to chelate transition metal ions (M^n+^) by means of the ferric reducing antioxidant power or FRAP assay [12], and their neutralizing affinities toward either 2,2-di(4-*tert*-octylphenyl)-1-picrylhydrazyl π-radical (DPPH^•^) [13], LOO^•^ [14], 2,2′-azinobis-(3-ethylbenzothiazoline-6-sulfonic acid) cation radical (ABTS^•+^) [15], and OH^•^ [16,17]. In addition, EOs have also been evaluated for their ability to protect against DNA damage induced by either peroxyl radicals (ROO^•^) or OH^•^ [18,19,20], to the best of the authors’ knowledge at the first time. Moreover, the results obtained in this study aided to update and upgrade the previously reported biological profiles of targeted EOs (Table 1 and Supplementary Materials Tables S1 and S3–S5) [21,22,23,24,25,26,27,28,29,30,31,32,33,34,35,36,37,38,39,40,41,42,43,44,45,46,47,48,49,50,51,52,53,54,55,56,57,58,59,60,61,62,63,64,65,66,67,68,69,70,71,72,73,74,75,76,77,78,79,80,81,82,83,84,85,86,87,88,89,90,91,92,93,94,95,96].

The in vitro antioxidant properties of the 61 EOs were analyzed by means of machine learning (ML) classification algorithms (Table 2) to correlate their chemical compositions (Supplementary Materials Table S2) with the obtained potencies, as a main objective of the enclosed study. Hence, a list of quantitative composition–activity relationships (QCARs) models was generated to shed light on the chemical components mainly responsible for the redox properties and to relate their contributions in terms of the positive or negative modulation of metal ion chelation/free radical neutralization. Previous applications of ML to EOs have enabled the correlation of their chemical composition to antimicrobial [96,97,98,99], antiviral [100], and anticancer properties [101,102].

In addition, EOs showing high potency in exerting antioxidant activity in vitro were selected for in vivo investigation using adult Wistar rats pre-exposed to carbon tetrachloride (CCl_4_) [103], in terms of their hepatoprotective/antioxidant effects and anti-genotoxic potentials.

To the best of the authors’ knowledge, this is the very first wide investigation in vitro and in vivo of EOs as antioxidants integrated with the application of ML algorithms to explain the antioxidant properties of EOs and their composition associated with in vivo data.

## 2. Materials and Methods

### 2.1. Essential Oils

The 61 EOs were acquired from Farmalabor srl (Assago, Italy). Their chemical compositions were analyzed by gas chromatography–mass spectrometry (GC-MS) as previously reported [97] (Table 3 and Supplementary Material Table S2). Briefly, each component was identified by comparing the obtained mass spectra with those reported in the Nist 02 and Wiley mass spectra libraries. Linear retention indices (LRIs) of each compound were also calculated using a mixture of aliphatic hydrocarbons. Knowing the chemical composition of the 61 EOs and their associated properties (Supplementary Material Table S2) [97] made them eligible as a dataset to develop QCARs models by means of ML algorithms [96,97,98,99,100,101,102]; therefore, they were also used herein to generate a list of ML models for antioxidant activities.

For experimental purposes, the EOs were dissolved in dimethyl–sulfoxide (DMSO) at 50 mg/mL to obtain complete solubilization and further diluted in the medium for in vitro and in vivo experiments, always resulting in a DMSO concentration that does not affect experimental protocols.

### 2.2. Chemicals and Reagents

Sulfuric acid (CAS No. 7664-93-9), sodium phosphate dibasic (CAS No. 7558-79-4), sodium phosphate monobasic (CAS No. 13472-35-0), ammonium molybdate (CAS No. 12054-85-2), ferrous sulfate (CAS No. 7782-63-0), ferrozine (3-(2-Pyridyl)-5,6-diphenyl-1,2,4-triazine-4′,4″-disulfonic acid sodium salt, CAS No. 69898-45-9), linoleic acid (CAS No. 60-33-3), Tween-40 (CAS No. 9005-66-7), ammonium thiocyanate (CAS No. 1762-95-4), DPPH (2,2-Diphenyl-1-picrylhdrazyl, CAS No. 1898-66-4), ABTS (2,2′-azino-bis(3-ethylbenzothiazoline-6-sulphonic acid), CAS No. 30931-67-0), potassium persulfate (CAS No. 7727-21-1), iron(III)chloride hexahydrate (CAS No. 10025-77-1), ascorbic acid (CAS No. 50-81-7), EDTA (Ethylenediaminetetraacetic acid, CAS No. 60-00-4), 2-deoxy-ribose (CAS No. 533-67-5), hydrogen peroxide (CAS No. 7722-84-1), TBA (2-thiobarbituric acid, CAS No. 504-17-6), TCA (trichloroacetic acid, CAS No. 76-03-9), DTNB (CAS No. 69-78-3), hydrochloric acid (CAS No. 7647-01-0), sodium carbonate (CAS No. 497-19-8), sodium bicarbonate (CAS No. 144-55-8), sodium dodecyl sulfate (CAS No. 151-21-3), acetic acid (CAS No. 64-19-7), sodium hydroxide (CAS No. 1310-73-2), and epinephrine (CAS No. 51-43-4) were purchased from Sigma-Aldrich Corp (St. Louis, MO, USA). Deoxyribonucleic acid from salmon sperm (CAS No. 100403-24-5) was purchased from Sigma Aldrich (St. Louis, MI, USA), while 2,2′-azo-bis(2-methylpropionamidine)dihydrochloride (AAPH) was obtained from Acros, Organics (New Jersey, USA). Low-melting-point agarose (CAS No. 9012-36-6), normal-melting-point agarose (CAS No. 9012-36-6), and ethidium bromide (CAS No. 11497653) were obtained from Alfatrade Enterprise D.O.O. (Serva Electrophoresis GmbH, Heidelberg, Germany). Assay kits for the determination of aspartate transaminase (AST, Ref. MX41264), alanine transaminase (ALT, Ref. MX41274), alkaline phosphatase (ALP), γ-glutamyltransferase (γ-GT, Ref. MX41288), and the total protein concentration (Ref. 1001290) were purchased from Spinreact (Girona, Spain). The Xanthine Oxidase Assay Kit (ab102522) and Nitric Oxide Assay Kit (ab 65328) were purchased from Abcam, while the cytochrome c reductase NADPH assay kit (CY0100-1KT) and Glutathione Peroxidase Assay Kit (MAK437) were purchased from Sigma-Aldrich Corp (St. Louis, MO, USA).

### 2.3. Antioxidant Activity

#### 2.3.1. Total Antioxidant Capacity

The TAC of the EOs was evaluated using the phosphomolibdenium method [11]. An aliquot of 100 μL of each EO was mixed with 1 mL of reagent solution (0.6 M sulfuric acid, 28 mM sodium phosphate, and 4 mM ammonium molybdate). The mixture was incubated at 95 °C for 90 min, and after cooling to room temperature (25 °C), the absorbance was measured at 695 nm. All measurements were performed using a Dynamica HALO DB-20 UV-Vis spectrophotometer. The results were calculated using a calibration curve for ascorbic acid and expressed as the mg of ascorbic acid equivalents per g of EO (mg AAE/g EOs).

#### 2.3.2. Metal Chelating Ability (Ferric-Reducing Antioxidant Power (FRAP) Assay)

To evaluate the ability of EOs to chelate transition metals and avoid the generation of free radicals, a ferrous ion chelation assay was used [12]. The reaction mixture contained 1 mL of 0.125 mM FeSO_4_ solution and 1 mL of 0.3125 mM ferrozine water solution, as well as 2 mL of serial dilutions of EOs or reference compound dissolved in methanol. The mixtures were kept for 10 min at room temperature (25 °C), and the absorbance was measured at 562 nm. The ability of EOs to chelate metal ions was expressed as the percentage of inhibition and calculated according to Formula (1):

(1) $$\%inhibition=\frac{A_{c}-A_{t}}{A_{c}}*100$$

where *A_t_* is the absorbance of the sample and *A_c_* is the absorbance of the control sample. The concentration of the tested EOs that reduces 50% of the initial metal ion/free radical concentration, i.e., the EC_50_ value, was calculated using the dose–response sigmoidal curve, as implemented in OriginPro v8.1 (OriginLab Corporation, Northampton, MA, USA).

#### 2.3.3. DPPH-Radical-Neutralizing Activity Assay (DPPH Assay)

The determination of the potency of EOs to neutralize the DPPH radical was per-formed according to the described method [13]. An aliquot of 2 mL of each EO (serial dilutions in methanol 2-0.0078 μg/mL) or the reference compound was mixed with 1 mL of 80 μg/mL DPPH solution in methanol. The control sample contained no EOs or reference compounds and was prepared using 1 mL of DPPH solution and 1 mL of methanol. After 30 min, the absorbance was measured at 517 nm. All measurements were performed in triplicate, and the average absorbance was calculated for each concentration. The ability of the EO to neutralize DPPH was expressed as the percentage of inhibition according to Formula (1), and the EC_50_ value was calculated similarly for the FRAP assay.

#### 2.3.4. Inhibition of Lipid Peroxidation

The potential of EOs to inhibit lipid peroxidation was estimated by the thiocyanate method [14]. Samples were prepared by adding 0.5 mL of each EO (serial dilutions in methanol 2-0.0078 µg/mL), 2.5 mL linoleic acid emulsion (prepared by mixing 0.2804 g linoleic acid), and 0.2804 g Tween-40 in 50 mL 40 mM sodium phosphate buffer, pH 7.0. The reaction mixtures were incubated at 37 °C for 72 h. To 0.1 mL of this solution, 4.7 mL of 75% ethanol, 0.1 mL of 30% ammonium thiocyanate solution, and 0.1 mL of 20 mM FeSO_4_ solution were added. After stirring for 3 min, the absorbance was measured at 500 nm against methanol (blank). The inhibition percentage of linoleic acid peroxidation was calculated using Formula (1), and the EC_50_ value was calculated similarly to the FRAP assay.

#### 2.3.5. ABTS-Radical-Cation-Neutralizing Activity (ABTS Assay)

The ability of EOs to neutralize the 2,2′-azino-bis(3-ethylbenzothiazoline-6-sulfonic acid (ABTS) radical cation [15] was measured as follows: 0.2 mL of each EO (serial dilutions in methanol 2-0.0078 µg/mL) or reference compound and 1.8 mL of the ABTS radical cation working reagent (prepared 16 h earlier by mixing equal amounts of 7 mM ABTS and 2.45 mM K_2_S_2_O_8_ and adjusted to an absorbance of 0.7 at 734 nm before addition to the tubes). In parallel, a control sample was prepared with only methanol. The reaction mixtures were kept in the dark for 30 min, and then the absorbance was measured at 734 nm. The percentage of neutralization of the ABTS radical cation was calculated using Formula (1), and the EC_50_ value was calculated similarly to the FRAP assay.

#### 2.3.6. Hydroxyl-Radical-Neutralizing Activity (Hydroxyl Radical Antioxidant Capacity (HORAC)) Assay

The determination of the hydroxyl-radical-neutralizing activity of EOs was measured according to the method described in [16]. Reaction mixtures were prepared as follows: 0.2 mL of serial dilutions of each EO dissolved in methanol were mixed with 0.2 mL of 10 mM FeCl_3_ solution, 0.1 mL of 1 mM ascorbic acid solution, 0.1 mL of 1 mM EDTA solution, 0.2 mL of 10 mM 2-deoxy-ribose solution, and 0.1 mL of 10 mM H_2_O_2_ solution. The tubes were then incubated at 37 °C for 1 h, 1 mL of 0.5% TBA in 10% TCA water solution was added, and the resulting mixture was incubated at 80 °C for 30 min. After cooling to room temperature (25 °C), the absorbance was measured at 535 nm. The percentage of inhibition was calculated using Formula (1), and the EC_50_ value was calculated similarly to the FRAP assay.

### 2.4. Antigenotoxic Activity In Vitro

#### The Protective Activity of Essential Oils against Peroxyl- and Hydroxyl-Radicals-Induced DNA Damage

The protective effect of the EOs (25, 50, 100, 200, and 400 μg/mL) against either peroxyl-radical-induced (generated by 2,2′-azobis-2-methyl-propanimidamide (AAPH)) or hydroxyl-radical-induced DNA damage was evaluated in vitro using salmon sperm DNA [18,20]. In both assays, quercetin (100 μg/mL) was used as a reference [19]. DNA bands on agarose gels were visualized under UV light (UV Transilluminator, Vilber Lourmat, France) at 365 nm, photographed, and analyzed using ImageJ software (version 1.48 for Windows, Softonic International, Barcelona, Spain).

### 2.5. Machine Learning

All analyses were performed using the Python programming language (version 3.7) [104,105] by executing in-house code in the Jupyter Notebook platform [96,97,106]. The chemical composition of each EO and the antioxidant activity data were imported and transformed into a dataframe and pre-processed to the final datasets to obtain the classification models. Scikit-learn (sklearn) [107] and the Pandas [108,109] libraries were used to implement ML algorithm protocols. Initial QCAR models were elaborated through 5 different classification algorithms (SVM, RF, GB, DT, and KNN). The dataset was divided into training and test sets using a ration (80:20) using the stratify splitting method implemented in sklearn.

During model development, an unsupervised dimensionality reduction/transformation was also performed with principal component analysis (PCA) [110] to extract 60%, 70%, 80%, 90%, and 99% of the explained variance (Table S6, Supplementary Material). Moreover, components represented by an occurrence as low as 1, 2, 3, and 4 times were therefore eliminated from the training set (n-level). Data were also processed using the MinMaxScaler function (scaling), which allows the original values to be scaled to ranges between 0 and 1.

Different cut-off values (thresholds) related to the antioxidant EC_50_ were used to develop ad hoc models with the optimal active/inactive ratio defined by the threshold value itself (Supplementary Material Table S7). The PCA, n-level, scaling, and threshold represented the dataset pretreatment parameters.

Differently from previously reported studies, the models were optimized in predictive ability; therefore, cross-validation was only performed on the final models to characterize their robustness. The optimal pretreatment parameters and ML hyperparameters (Supplementary Material Tables S8 and S9) were selected through the predictive Matthews correlation coefficient (MCC_pred_) obtained by comparing the experimental classes (active or inactive) with the predicted one.

Due to the high number of considered pretreatment parameters and hyperparameter combinations, the ML modeling strategy was conducted as follows:

1. Coarse ML models generation was run with 100 and 1000 random combination runs from all possible considered combinations (Tables S6–S8, Supplementary Material) [111], so we selected the suboptimal ML algorithms and associated pretreatment parameters (n-level, scaling, threshold, and PCA). For details, see Supplementary Material;
2. Refined models were investigated with 10,000 and 100,000 random combination runs from all possible considered combinations (Supplementary Material Tables S6, S7 and S9), while avoiding those non-selected in the previous point. The selection of the final models was based on the MCC_pred_ values. For details, see Supplementary Material (Tables S10–S65);
3. The final model was finally defined by retaining the optimal parameters selected at point 2 and using the full dataset. The accuracy (ACC), F1 score, and MCC were used to evaluate the binary classification models numerically and graphically. The importance of each chemical component present in the EOs was independently evaluated through the “feature importance” (FI) and partial dependence (PD) [112] methods, as implemented in the Skater Python library [113]. In addition, Spearman’s correlation coefficient was used to weight the correlation between the percentage presence of a component in the EOs and its partial dependence, thus obtaining the weighted FI values (WFI). Models were evaluated by leave-some-out CV by means of five groups using the stratified K-fold method while monitoring the average value of MCC_CV_ obtained from 50 random CV iterations [98,114].

### 2.6. Animals and Study Design

Forty male albino Wistar rats weighing 220 ± 20 g used in this study were obtained from the Animal House of the Military Medical Academy, Belgrade, Serbia, and acclimated for 3 days before the experiment. They were maintained under a 12 h light–dark cycle, and food and water were provided ad libitum. All animal procedures were ap-proved by the Ethical Committee of the Faculty of Science, University of Kragujevac (Ethical approval number 1-03/2023), which acts according to the relevant Serbian guidelines, including the Guidelines for the Care and Use of Laboratory Animals and the Law on Animal Welfare (“Official Gazette of the Republic of Serbia,” No. 810 41/09) and the Euro-pean Directive for the Welfare of Laboratory Animals Directive 2010/63/EU.

In the current study, the rats were randomly divided into 8 groups of 5 rats each. Group I (negative control) was injected intraperitoneally with 1 mL/kg body weight of commercial olive oil (Monini Olio Extra Vergine di Oliva). Group II (positive control) received a single intraperitoneal dose of CCl_4_, 1 mL/kg body weight, 1:1 mixture in olive oil [115]. The remaining groups of animals (groups III to VIII) were co-treated (1, 200, and 400 mg/kg body weight) with EOs selected among the more potent against each of the in vitro redox species and CCl_4_ (1 mL/kg body weight). The rats were sacrificed after 24 h under light ether anesthesia, and the kidney and liver organs were removed and cleaned.

### 2.7. Measurement of Antioxidant Markers

Isolated kidney and liver organs were used for the measurement of redox and toxicity markers and the determination of DNA-protective activity. Rat liver and kidney samples were homogenized in phosphate buffer (5 mM, pH 7.4) to obtain a 10% (*w*/*v*) homogenate and then centrifuged at 4000 rpm for 15 min at 4 °C. The supernatants were used to estimate the catalytic activity of superoxide dismutase (SOD) [116], the level of TBARS [117], the catalytic activity of catalase (CAT) [118], and the concentration of reduced glutathione (GSH) [119] through the colorimetric method. Total protein concentrations were determined according to the Lowry method [120]. All colorimetric measurements were performed using a Dynamica HALO DB-20 UV-VIS spectrophotometer.

#### 2.7.1. Measurement of Serum Toxicity Markers

Blood samples were collected from each animal for serum preparation according to the Quick method [121]. Serum for the determination of biochemical parameters, aspartate transaminase (AST) [122], alanine aminotransaminase (ALT) [123], alkaline phosphatase (ALP) [124], and γ-glutamyltransferase (γ-GT) [125] was prepared through the Quick method [121], immediately immersed in liquid nitrogen, and stored at −80 °C until use. The catalytic activities of AST and ALT at 340 nm and of ALP and γ-GT at 405 nm were determined through the UV-VIS kinetic methods according to the recommendations of the Expert Panel of the International Federation of Clinical Chemistry (IFCC) [59,60,61,62]. Total protein concentrations were determined through the Lowry method using bovine serum albumin as the standard [63]. All kinetic and colorimetric measurements were performed using a Dynamica HALO DB-20 UV-VIS spectrophotometer.

#### 2.7.2. Xanthine Oxidase Catalytic Activity

Xanthine oxidase activity in kidney homogenates was estimated using a spectro-photometric xanthine oxidase assay kit from Abcam (ab102522) [126]. Briefly, 50 μL of kidney homogenate (H_2_O_2_ standard or xanthine oxidase positive control diluted in dH_2_O) and 50 μL of sample reaction mix (containing assay buffer, xanthine oxidase substrate mix, developer solution V, and OxiRed probe) were added to each well and mixed well. Measurement was performed immediately at 570 nm and again after incubation of the reaction at 25 °C for 10–20 min. The calculation was performed using the H_2_O_2_ standard curve and expressed as U/L.

#### 2.7.3. NADPH Oxidase Catalytic Activity

Renal NADPH oxidase activity was measured using a cytochrome c reductase NADPH assay kit (Sigma, USA) [127]. This assay measures the reduction of cytochrome c by NADPH–cytochrome c reductase in the presence of NADPH. The absorbance spectrum of cytochrome c changes with its oxidation/reduction state. The reduction of cytochrome c is monitored by the increase in the absorbance of cytochrome c at 550 nm. The results are expressed as U/mg protein. One unit of enzyme activity reduces 1 μmol of oxidized cytochrome c per minute in the presence of 100 μmol of NADPH at pH 7.8 and 25 °C.

#### 2.7.4. Nitric Oxide Catalytic Activity

Nitric oxide (measured as nitrates/nitrites concentration) was determined using the Abcam colorimetric nitric oxide assay kit (ab 65328) [128]. To each well, 85 μL of kidney homogenates (previously deproteinized) or nitrate standard solutions diluted in assay buffer, 5 μL of nitrate reductase enzyme, and 5 μL of enzyme cofactor were added. The blank sample contained assay buffer only. The covered plate was incubated for 60 min at room temperature (25 °C). Next, 5 μL of Enhancer was added to the standard and sample wells only, and the incubation continued for an additional 10 min. Finally, 50 μL of Griess Reagent I and 50 μL of Griess Reagent II were added to the standard and sample wells, and the OD was measured at 540 nm. The concentration of nitrates/nitrites was determined from the standard curve.

#### 2.7.5. Glutathione Peroxidase Activity

Renal glutathione peroxidase (GPx) activities of EO were determined through the method based on the oxidation of GSH (Sigma-Aldrich Chemical Co., USA) by hydrogen peroxide (H_2_O_2_) (El-Nasr Pharmaceutical Co., Oubour, Qalyubia, Egypt) in the presence of GPx [48]. The decrease in absorbance was measured at 340 nm as NADPH (Sigma-Aldrich Chemical Co., USA) and it was converted to NADP^+^, reflecting the amount of oxidized glutathione formed and, consequently, the activity of GPx. Enzyme activity was expressed as U/mg protein, where one unit is defined as the amount of enzyme oxidizing 1 μmol NADPH per minute at 25 °C.

### 2.8. Assessment of In Vivo Antigenotoxic Activity

The DNA-protective potential of EO was detected using the alkaline comet assay [129]. Images were visualized and captured using a 40x objective of a Nikon (Ti-Eclipse) fluorescence microscope attached to a CCD camera. One hundred randomly selected cells (fifty cells per two replicate slides) per treatment were analyzed using a visual scoring method [130]. Comets were classified into five types defined as T0, T1, T2, T3, and T4 (no or very low damage, low, medium, and long DNA migration, and the highest level of degradation, respectively). Total comet scores and the percentage of reduction (%R) in the total comet scores were calculated as described elsewhere [131].

### 2.9. Statistical Analysis

The results are expressed as mean ±SEM, and the statistical evaluation of data was performed through one-way analysis of variance (ANOVA) using the SPSS statistical software package, version 13.0, running on Microsoft Windows version 10. The significance level was set at *p* < 0.05.

## 3. Results

### 3.1. Determination of In Vitro Antioxidant Activity

#### 3.1.1. Total Antioxidant Capacity

TAC (Supplementary Materials Table S3), for the majority of the EOs herein not previously reported (NPR, Supplementary Materials Table S1), refers to the general ability of a given EO to oxidize free radicals, and it was evaluated here in vitro using the phosphomolybdenum method However, because the TAC results do not explain the metal-chelating/free-radical-neutralizing potency of either ROS or RNS but instead only compare the potency of an antioxidant with an ascorbic acid (AA) by AA equivalents (AAE; the higher the number of μg AAE/mg, the greater should be the antioxidant potency), the test may lead to false positive values.

Therefore, EOs ranging from 0.002 (*Origanum hirtum* EO) to 1.231 μg AAE/mg (Black pepper EO) were considered suitable for future in vitro studies. Notably, Black pepper EO had no potency against M^n+^, DPPH^•^, LOO^•^, ABTS^•+^, or OH^•^, whereas *Origanum hirtum* EO showed remarkable potency for all radical species except for M^n+^ (Table 1). Consequently, TAC data poorly correlated with the antioxidant potentials of the tested EOs. For example, among all EOs with a TAC higher than 0.250 μg AAE/mg, only Birch EO with a TAC value of 0.522 μg AAE/mg exerted potency against all redox species except M^n+^. EOs with lower TAC, such as Cedar leaf EO, showed a similar profile. On the other hand, remarkable potency against M^n+^, DPPH^•^, LOO^•^, ABTS^•+^, and OH^•^ (Table 1) was observed for EOs with low TAC values (0.119 to 0.006 μg AAE/mg), such as Savory, Rosemary, Ylang-ylang (YY EO), and Ceylon cinnamon peel (CCP EO). In contrast, EOs showing TAC ranging from 0.131 μg to 0.003 μg AAE/mg had only the ability to chelate M^n+^ (Supplementary Materials Table S3).

#### 3.1.2. Metal-Ions-Chelating Activity

**Scheme 1:**
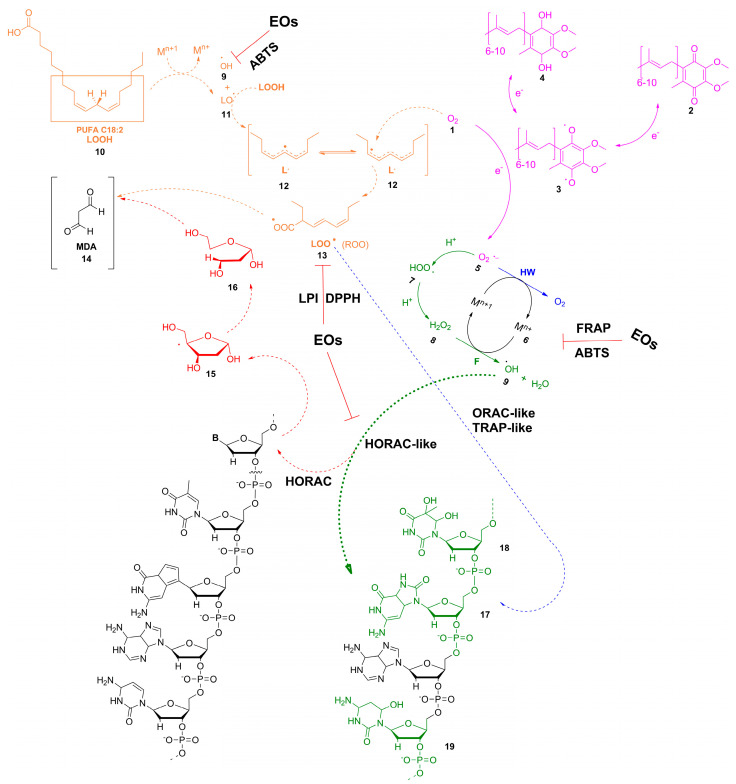
The inter-correlation of methods used for monitoring/neutralizing in vitro and in vivo, emphasizing the emergence of ROS by means of the EOs herein. The in vivo emergence of redox-inducing species (pink path, thick arrows): O_2_, (**1**), coenzyme Q quinone form (**2**), coenzyme Q radical form (**3**), coenzyme Q hydroquinone form (**4**), O_2_^•−^ (**5**); The in vitro assays: FRAP and ABTS assays (green path, thick arrows): Fe^2+^ ion (**6**), hydroperoxide radical (HOO^•^, **7**)_,_ H_2_O_2_ (**8**), OH^•^ (**9**); FRAP and ABTS assays (blue path, normal arrow): Fe^3+^ ion (**10**); DPPH and LPI assays (orange path, dashed arrows): polyunsaturated fatty acid (**10**)_,_ lipid oxy-radical, L^•^ (**11**), lipid radical, L^•^ (**12**), lipid peroxyl-radical, LOO^•^ (**13**), malondialdehyde (**14**); HORAC assay (red path, dashed arrows): 2′-deoxyribose radical (**15**), riboxyradical (**16**); ORAC- or TRAP-like electrophoretic assay (blue path, dashed arrow): 8-oxoG (**17**); HORAC-like electrophoretic assay (green path, dashed arrows): 8-oxoG (**17**), thymineglycol (**18**), 6-hydroxy-5,6-dihydrocytosine (**19**).

Regarding Eos’ chelating abilities of metal ions, the Fe^2+^ ion (M^n+^) is involved in the redox signaling of molecular oxygen O_2_ (Scheme 1, pink pathway, **1**), a major precursor of ROS in biological systems. O_2_, being an electron acceptor of coenzyme Q in the mitochondrial electron transport chains (Scheme 1, pink pathway, **2**–**4**), generates O_2_^•−^, the first oxidative stress inducer (Scheme 1, pink pathway, **5**) [7,132]. O_2_^•−^ is a short-living species undetectable by in vitro assays [7]; nevertheless, it reacts with other radicals to form other reactive species [133] that can be routinely determined either in vitro or in vivo [9,10], making the variety of in vitro or in vivo methods somewhat correlated (Scheme 1).

O_2_^•−^ has a low membrane permeability; therefore, it reacts mainly in the physiological compartment where it is generated and then passes through ion channels, initiating two crucial reactions for the triggering of oxidative stress: the Fenton-type (Scheme 1, green pathway, **F**) [9] and the Haber–Weiss reaction (Scheme 1, blue pathway, **HW**) [10]. Both reactions are catalyzed by transition metal ions (M^n+^, usually Fe^2+^ and Fe^3+^, but also Cu^2+^, Ni^2+^, Co^2+^, and V^2+^) [12]. Thus, the Fenton reaction catalyzed by Fe^2+^ ions (Scheme 1, green pathway, **6**) yields hydroperoxide radical (HOO^•^) (Scheme 1, green path, **7**)_,_ H_2_O_2_ (Scheme 1, green path, **8**) and OH^•^ (Scheme 1, green path, **9**) [9,133], whereas the Haber–Weiss reaction, catalyzed by Fe^3+^ ions, yields new amounts of O_2_ (Scheme 1, blue path **10**) [133]. Subsequently, either O_2_ or HOO^•^ is involved in the lipid autooxidation/peroxidation of polyunsaturated fatty acid (Scheme 1, orange pathway, **11**) [134,135], on the level of propagation, while targeting the carbon-centered lipid radicals (alkyl radicals, L^•^, Scheme 1, orange path, **12**) to yield LOO^•^ (Scheme 1, orange pathway, **13**), and, finally, malondialdehyde (MDA) (Scheme 1, orange pathway, **14**) [135]. Metal ion chelation in vivo could slow down peroxidation, prevent the decomposition of lipid hydroperoxides into other components capable of abstracting hydrogen, and stop the reaction chain of lipid peroxidation [134].

Because M^n+^ metal ions play an important role at the very beginning of redox signaling or later in the redox signaling cascade (Scheme 1, green path, thick arrows), the chelating ability of the present EOs was measured by FRAP assay. As already indicated, 12 out of 61 EOs (~20% of the TAC-active EOs) (namely, YY, Grapefruit, Eucalyptus, Bergamot, Fennel, Cedar fruit, Lemon, Roman chamomile, Savory, Rosemary, CCP, and *Eucalyptus globulus* EOs) showed chelating properties in the range of EC_50_ = 0.63–1.72 μg/mL. Nevertheless, no significant linear regression correlation was obtained between TAC and M^n+^ potencies. Interestingly, the YY, CCP, Rosemary, and Savory EOs were active in all antioxidant assays and characterized by medium to high EC_50_ values of 0.84, 095, 1.39, and 1.46 μg/mL, respectively (compared to NPR, 25–68,380 μg/mL, 195 μg/mL, and NPR, Supplementary Materials Table S3).

#### 3.1.3. DPPH^•^-Radical-Neutralizing Activity

The mechanism of DPPH^•^ neutralization is comparable to that against LOO^•^ (see next) during the rate-limiting propagation step of lipid peroxidation (Scheme 1, orange pathway, dashed arrows, **13**) [7,135,136]; therefore, the results of the DPPH assay could be used to predict lipid peroxidation inhibition potency [137]. Only 17 EOs (Red thyme, Oregano, YY, Lemongrass, Cade, Cedar leaves, Birch, Savory, Rosemary, CCP, White thyme, Clove, Laurel, *Coridothymus capitatus*, *Thymus vulgaris*, and *Origanum hirtum*) exhibited good affinity to neutralize DPPH radicals [13] over the EC_50_ concentration range of 0.007 µg/mL (Cade EO) to 0.99 µg/mL (*Thymus vulgaris* EO). CCP, Savory, Rosemary, and YY EO were characterized with an EC_50_s of 0.023, 0.110, 0.375, and 0.630 µg/mL, respectively (Table 1 and Supplementary Materials Table S3).

As for metal-ions-chelating activity, no significant linear correlation was observed between TAC and DPPH assays values. Nevertheless, the present EOs probably reduced the divalent N-atom of DPPH^•^ to the hydrazine DPPH^•^H by donating either their alcoholic or methylene protons [1] through the “hydrogen atom transfer” (HAT) mechanism or phenolic protons through the “proton-coupled electron transfer” (PCET) mechanism [137].

#### 3.1.4. Lipid Peroxidation Inhibition Activity

EOs were evaluated for their ability to stop lipid peroxidation processes within the membrane [135,136] by means of in vitro [14] and in vivo [103] lipid peroxidation inhibition (LPI) assays (see next). Here, each EO neutralized LOO^•^ in vitro within a linoleic acid/water emulsion system [14] and was compared against the BHT, used as a reference.

At first glance, all EOs active in the DPPH assay showed remarkable potency for LOO^•^, and none of the DPPH^•^-inactives exerted any affinity for LOO^•^. The total EC_50_ con-centration range of active EOs against LOO^•^ ranged from 0.005 (Oregano EO) to 0.75 µg/mL (Laurel EO). In addition, the results of the DPPH and LPI assays significantly correlated with an *r*^2^ value of 0.79 ([EC_50_] LOO^•^ = 1.0511 × [EC_50_] DPPH^•^ + 0.0599), indicating some predictivity ability for LPI from a DPPH assay. Among the 17 EOs found active in the DPPH^•^ assay above, Red thyme, Oregano, YY, Lemongrass, Rosemary, Natural nutmeg, *Thymus vulgaris*, *Coridothymus capitatus*, and White thyme were more potent against LOO^•^ and Cade and Birch EO were equally potent against both radicals, whereas Cedar leaf, CCP, Clove, and *Origanum hirtum* EO were more potent against DPPH^•^ with respect to LOO^•^. Interestingly, CCP, Savory, Rosemary, and YY EOs, potent against any given metal ion/radical species, showed EC_50_s against LOO^•^ of 0.032, 0.180, 0.230, and 0.230 µg/mL, respectively (Supplementary Materials Table S3).

By exerting potency through the lipid peroxidation assay, the above-listed EOs can be classified either as chain-breaking antioxidants (when containing hydroxyl groups), interrupting lipid peroxidation at the rate-limiting propagation step and neutralizing the LOO^•^, or as termination-enhancing antioxidants (when containing nonphenolic terpenoids, i.e., methylene C-H as proton donors) [1], resulting in an overall increase in the rate of oxidative chain termination [1] and for the most potent EOs confirmed in vivo (see text below) [103].

#### 3.1.5. ABTS-Cation-Radical-Neutralizing Activity

Either Fenton-type reactions [9] or lipid peroxidation initiation reactions [34,35] produce an excess of OH^•^ (Scheme 1, green and orange paths, dashed arrows, **9**). While the effect of these EOs on Fenton-type OH^•^ biosynthesis was later determined by modulating the catalase-driven decomposition of H_2_O_2_ in vivo (Scheme 1, dark green path, bold arrows) [7,103], it was first assessed indirectly in vitro using the ABTS assay [15] (ABTS^•+^/DPPH^•^ and ABTS^•+^/LOO^•^ interrelationships are reported in the Supplementary Material). Thus, in the ABTS/H_2_O_2_/peroxidase (catalase) in vitro system, ABTS acts as a reducing agent, substituting the enzyme’s compound I (i.e., porphyrin radical cation, Por-+-Fe^IV^=O) for compound II (i.e., hydroxoferryl derivative), for which the ABTS^•+^ is formed, and returning to the initial form of the enzyme [38,138], thus supporting H_2_O_2_ decomposition. The rationale for using the ABTS assay was due to the question: “If the EOs are capable of neutralizing the ABTS^•+^ in vitro, could they support catalase in vivo in terms of facilitating the decomposition of oxidative-stress-generated H_2_O_2_?”

ABTS^•+^ neutralization was evaluated after the radical was formed by ABTS oxidation with K_2_S_2_O_8_ [15], where each EO provided the reducing equivalent to the N atom, likely via the sequential proton loss electron transfer (SPLET) mechanism [40,139]. Thus, the overall EC_50_ concentration range of active EOs against ABTS^•+^ was from the most potent 0.003 (Red thyme EO) to the least potent 0.89 µg/mL (*Mentha suaveolens* EO) (Table 1 and Supplementary Materials Table S3). All EOs that were potent against DPPH^•^ also neutralized ABTS^•+^, except for Laurel, which had an EC_50_ against ABTS^•+^ greater than 1 μg/mL. On the other hand, *Mentha suaveolens* EO, which was virtually inactive against DPPH^•^ (EC_50_ > 1 μg/mL), was highly active against ABTS^•+^, with an EC_50_ value of 0.890 μg/mL. Again, as already observed above for other assays, Rosemary, CCP, Savory, and YY EOs continued to exert a good level of potency also against ABTS^•+^ with an EC_50_s of 0.065, 0.125, 0.260, and 0.760 μg/mL, respectively.

#### 3.1.6. Hydroxyl-Radical-Neutralizing Activity

Based on the results of the ABTS assay, EOs were further tested for their ability to neutralize the hydroxyl radicals in vitro (Table 1) by means of the HORAC assay, in terms of preventing the OH^•^ from attacking the C’4 position of 2’-deoxy-D-ribose (Scheme 1, red pathway, dashed arrows, **15**) and causing its conversion to the ribosyl radical (Scheme 1, red pathway, dashed arrows, **16**) and malondialdehyde (MDA) (Scheme 1, red pathway, dashed arrows, **14**) [16,17]. The neutralization of OH^•^ by each EO was compared to BHT. Thus, all EOs found potent OH^•^ scavengers by the ABTS^•+^ assay and did indeed show a good affinity for OH^•^, and none of the ABTS^•+^-inactives exerted an affinity for OH^•^. However, a non-perfect correlation was found (*r*^2^ = 0.7601) for the equation [EC_50_] OH^•^ = 0.6389 × [EC_50_] ABTS^•+^ + 0.0501, implying that the ABTS assay should be taken with caution as a predictor of potency against OH^•^.

The EC_50_ concentration of active EOs against OH_•_ ranged from 0.01 (Red thyme EO) to 0.72 µg/mL (Nutmeg natural EO). In summary, eight EOs (Oregano, Clove, Red thyme, *Coridothymus capitatus*, Rosemary, Birch, Cade, and Nutmeg natural) were about 10% to 600% more sensitive to ABTS^•+^, whereas *Origanum hirtum*, YY, Savory, White thyme, CCP, *Thymus vulgaris*, Cedar leaf, and Lemongrass EOs were from 1.12- to 3.25-folds more sensitive to OH^•^. Moreover, Savory-, CCP-, YY-, and Rosemary-derived EOs were also active against OH^•^, with EC_50_s of 0.032, 0.078, 0.35, and 0.42 µg/mL, respectively (Supplementary Materials Table S3).

### 3.2. Antigenotoxic Activity In Vitro

Being able to prevent the 2’-deoxy-D-ribose degradation, EOs were further evaluated in vitro for their ability to prevent OH^•^-induced damage to a DNA double-strand [18,19,20] by interfering with salmon sperm DNA, while the selected EOs were also indirectly evaluated for their potency to neutralize OH^•^ in vivo [104].

The damage within the DNA double-strand was induced by the 2-hydroperoxy-2-methylpropanimidamide radical (ROO^•^), released from AAPH by the homolytic cleavage of the two nitrogen–carbon bonds to form two isobutyrimidamide radicals (R^•^) and subsequent oxidation with O_2_ [20,21]. The antioxidant potential of each EO has been evaluated using the electrophoresis-based version of the ORAC or TRAP assays [137]. The ROO^•^ (and its homolog LOO^•^) is associated with the modification of DNA, such as 8-oxoG (Scheme 1, blue pathway, dashed arrows, **17**), which could lead to inflammation and degenerative pathologies, including cardiovascular and neurological diseases, as well as the development of some cancers [140]. On the other hand, OH^•^, here formed in vitro in the Fenton-type oxidation of H_2_O_2_ catalyzed by FeSO_4_, also attacked the salmon DNA double-strand [8,18], and probably generated the aberrant adducts, such as 8-oxo-dG (Scheme 1, green pathway, dashed arrows, **17**), thymine glycol (Scheme 1, green pathway, dashed arrows, **18**), and 6-hydroxy-5,6-dihydrocytosine (Scheme 1, green pathway, dashed arrows, **19**) [141,142].

**Figure 1:**
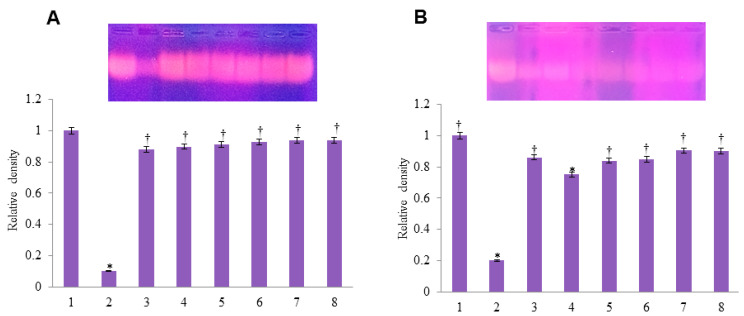
Protective effect of CCP EO against peroxyl- (**A**) and hydroxyl- (**B**) radicals-induced DNA damage. DNA from salmon sperm (lane 1, negative control), DNA damage control (lane 2, positive control), quercetin (lane 3, 100 μg/mL, standard), essential oils in concentrations of 25, 50, 100, 200, and 400 μg/mL (lanes 4, 5, 6, 7, and 8). * *p* < 0.05 when compared with the negative control group; ^†^ *p* < 0.05 when compared with the positive control group.

**Figure 2:**
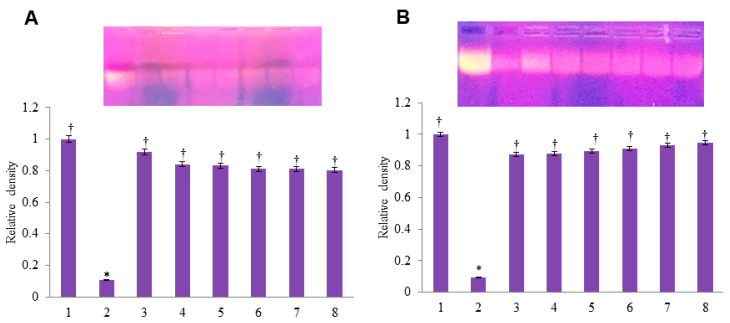
Protective effect of YY EO against peroxyl- (**A**) and hydroxyl- (**B**) radicals-induced DNA damage. DNA from salmon sperm (lane 1, negative control), DNA damage control (lane 2, positive control), quercetin (lane 3, 100 μg/mL, standard), essential oils in concentrations of 25, 50, 100, 200, and 400 μg/mL (lanes 4, 5, 6, 7, and 8). * *p* < 0.05 when compared with the negative control group; ^†^ *p* < 0.05 when compared with the positive control group.

To suppress the listed DNA lesions, EOs were evaluated using relative electrophoretic band densities (RBD) of DNA (Figure 1 and Figure 2, and Supplementary Material Figures S1–S59) knowing that RDB values approaching **1** indicate better protection (Supplementary Material Tables S4 and S5). Each EO was administered at double-diluted concentrations ranging from 400 to 25 µg/mL and compared to quercetin (Q, 100 µg/mL) as a reference compound [18,20]. The highest starting concentration tested against M^n+^, DPPH^•^, LOO^•^, ABTS^•+^, and OH^•^ radicals was acceptable considering that only high concentrations of an antioxidant can efficiently compete with LOO^•^ and OH^•^ at the cellular site of radical formation [8]; (2) was used as a metric to predict which EO concentration could be tested in vivo [103]. An analysis of RBD values alone (see Supplementary Materials) did not provide significant conclusions regarding the efficacy of EOs but only indicated their affinity for either ROO^•^ or OH^•^. Therefore, ROO^•^RBD_50_ or OH^•^RBD_50_ values, i.e., concentrations that stimulate a 50% increase in relative electrophoretic band density [RBD] (i.e., protection), were calculated (Table 1) to differentiate the potencies of all EOs.

#### 3.2.1. EOs with Increasing Dose-Dependent Potency to Protect DNA from ROO^•^ and OH^•^

Among the tested EOs, 33 of them (Chamomile Morocco, Tea tree, Melissa, Mountain pine, Geranium Bourbon, Oregano, Lavender, Myrtle, Hyssop, Lemongrass, Siberian pine, Camphor, Ginger, Cumin, Patchouli, Orange bitter, Eucalyptus, Bergamot, Fennel, Savory, Rosemary, CCP, *Eucalyptus globulus*, Artemisia, White thyme, Marjoram, Cypress, Peppermint, Palmarosa, Natural anise, Frankincense, *Mentha suaveolens*, and *Coridothymus capitatus*) protected salmon DNA from damage induced by either ROO^•^ or OH^•^ in a dose-dependent manner, with the level of protection increasing with increasing concentrations (Tables S4 and S5, detailed analysis of data is reported in the Supplementary Materials). By determining the ROO^•^RBD_50_ and OH^•^RBD_50_ values, it appeared that only CCP EO either neutralized M^n+^, DPPH^•^, LOO^•^, ABTS^•+^, and OH^•^ or protected the DNA from either ROO^•^ or OH^•^ (Figure 1), with ROO^•^RBD_50_ and OH^•^RBD_50_ values as low as 60.99 and 78.36 µg/mL, respectively (Table 1); therefore, the EO was selected for in vivo administration. Savory and Rosemary EOs, initially selected for in vivo assays, were discarded due to undetermined ROO^•^RBD_50_ or OH^•^RBD_50_ values (Table 1).

#### 3.2.2. EOs with Decreasing Dose-Dependent Potency to Protect from ROO^•^ and OH^•^

Clary sage, Red thyme, Coriander, Grapefruit, Roman chamomile, Black pepper, and Clove EOs dose-dependently reduced DNA damage induced by radicals, but with no efficacy against ROO^•^ or OH^•^ (Supplementary Materials Tables S4 and S5). However, despite showing remarkable ROO^•^RBD_50_s and OH^•^RBD_50_s (Table 1), none of the EOs within this subgroup showed satisfactory efficacy against M^n+^, DPPH^•^, LOO^•^, ABTS^•+^, or OH^•^ and were not further evaluated.

#### 3.2.3. EOs with Increasing and Decreasing Dose-Dependent Potency to Protect DNA from ROO^•^ and OH^•^, Respectively

Sage, Garlic, Cade, Cedar leaves, Pine Silvestre natural, Juniper, Birch, Orange sweet, Cajeput, Nutmeg natural, Verbena, Basil, Laurel, *Thymus vulgaris*, and *Origanum hirtum* EOs, at increased concentrations, protected DNA with more power from the damage induced by ROO^•^, while, at the same time, gradually losing their efficacy against OH^•^ (Supplementary Materials Tables S4 and S5). Again, despite showing remarkable ROO^•^RBD_50_s and OH^•^RBD_50_s (Table 1), none of the listed EOs had efficacy against M^n+^, DPPH^•^, LOO^•^, ABTS^•+^, or OH^•^ and were not further evaluated.

#### 3.2.4. EOs with Decreasing and Increasing Dose-Dependent Potency to Protect DNA from ROO^•^ and OH^•^, Respectively

YY, Cardamom, Mandarin, Cedar fruit, Lemon, and Niaouly EOs exhibited a decreasing ability to protect DNA from ROO^•^-induced damage with increasing concentration but showed increasing efficacy against OH^•^. The most interesting EO was YY EO with ROO^•^RBD_50_ of 64.12 µg/mL and OH^•^RBD_50_ of 134.11 µg/mL (Table 1, Figure 2), and CCP EO with exceptional affinity to neutralize M^n+^, DPPH^•^, LOO^•^, ABTS^•+^, and OH^•^. These data were induced to select YY and CCP EOs for the subsequent in vivo evaluation.

### 3.3. Machine-Learning-Based QCAR Models

As mentioned above, the correlations of the in vitro antioxidant potencies of EOs with their chemical compositions were analyzed by means of ML algorithms to generate QCAR models (Table 2 and Supplementary Material Model development section). The predictivity and robustness of each derived model were evaluated using the MCC_Pred_ and MCCcv values. For comparison purposes with other metrics (i.e., accuracy), Normalized MCC (nMCC) was considered, defined as nMCC = MCC + 1/2, and linearly projecting the original range into the 0-1 interval. A model was generated for each of the evaluated in vitro assays (Table 2). In general, for all of the in vitro antioxidant activities, the procedure led to obtaining a model endowed with acceptable to good nMCC_Pred_ and nMCC_CV_ values in the ranges of 0.68–0.98 and 0.58–0.91, respectively, indicating the models to be endowed with good levels of both predictivity and robustness. The models can be freely used for the prediction of any untested EOs antioxidant potencies, as a part of the AI4EssOil Project (https://www.ai4essoil.com/front/, accessed on 20 September 2023).

**Figure 3:**
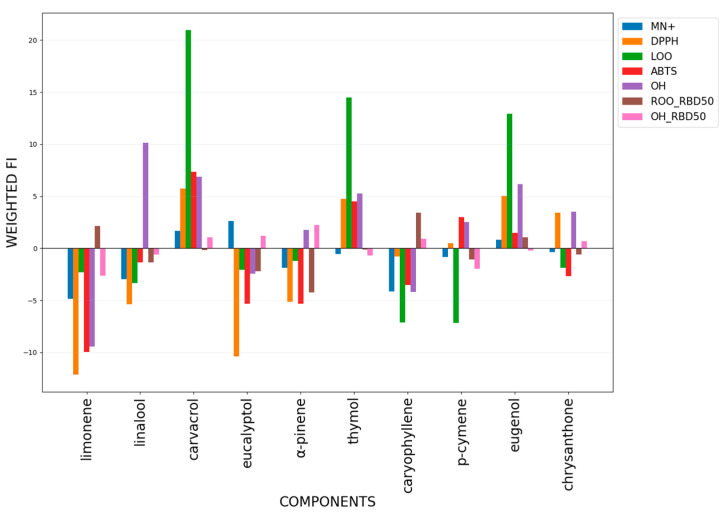
The WFI trends of the 10 components, which are most important and present in the 61 essential oils.

**Figure 4:**
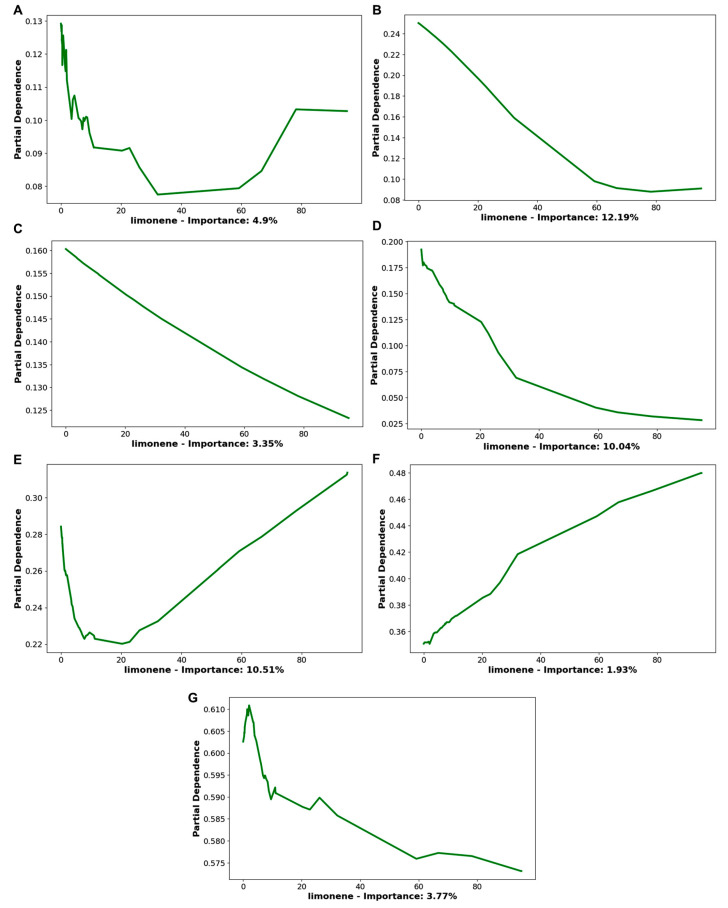
Partial dependence plots of limonene in the model of M^n+^ (**A**), DPPH^•^ (**B**), LOO^•^ (**C**), ABTS^•+^ (**D**), OH^•^ (**E**), ROO-RBD_50_ (**F**), HO-RBD_50_ (**G**).

**Figure 5:**
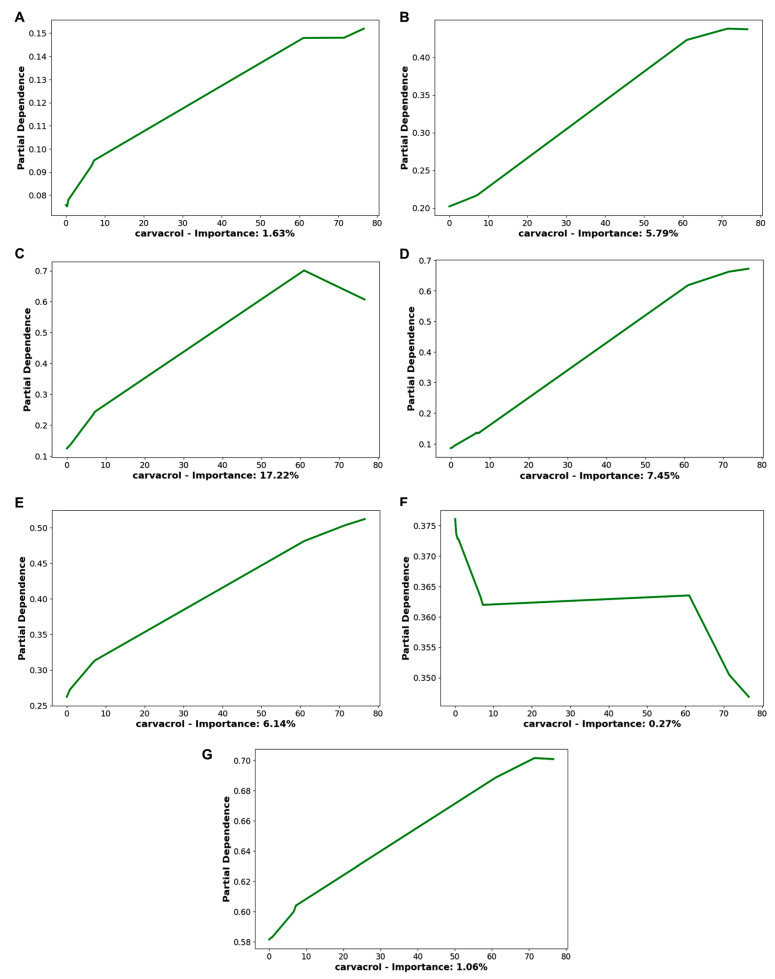
Partial dependence plots of carvacrol in the model of M^n+^ (**A**), DPPH^•^ (**B**), LOO^•^ (**C**), ABTS^•+^ (**D**), OH^•^ (**E**), ROO-RBD_50_ (**F**), HO-RBD_50_ (**G**).

Among the models, ML3 obtained for the lipid peroxidation arrest and generated by the SVM was that with the highest nMCC_Pred_ and nMCC_CV_ values (Table 2). The same ML algorithm “recognized” that DPPH^•^ radical neutralization could be used to predict potency against LOO^•^, giving a model with excellent prediction accuracy (Table 2, model ML2). These data are in good agreement with the above reported considerations. Good models were obtained when describing the potencies against ABTS^•+^ (Table 2, model ML4) and OH^•^ (Table 2, model ML5), but using KNN and RF algorithms, respectively. The ML model describing metal ion chelation (Table 1, model ML1) was of slightly lower predictive power, while the least quality models were those describing DNA double-strand protection (Table 1, models ML6 and ML7). Analysis of feature importance revealed that all models shared limonene (cyclic monoterpene, hydrocarbon), linalool (acyclic monoterpene, alcohol), carvacrol (cyclic monoterpene, phenol), eucalyptol (bicyclic monoterpene, hydrocarbon), α-pinene (bicyclic monoterpene, hydrocarbon), thymol (cyclic monoterpene, phenol), caryophyllene (bicyclic sesquiterpene, hydrocarbon), *p*-cymene (cyclic monoterpene, hydrocarbon), eugenol (cyclic monoterpene, hydrocarbon/phenol), and chrysanthone (tricyclic sesquiterpene, hydrocarbon/phenol) as the most important chemical components, which were characterized by the WFI (Figure 3) and PD plots (Figure 4 and Figure 5, and Supplementary Materials Figures S60–S67). In general, the results of all models were in good agreement with the previously reported chemical/biological properties.

#### 3.3.1. The Contribution of Limonene to the Antioxidant Activity

The presence of limonene was recognized by the ML models as negative and somehow opposite to the antioxidant activity in all models except for the one obtained to describe the neutralization of the ROO^•^ radical (Figure 3). Moreover, by analyzing the partial dependence plots of limonene for each free radical, it can be confirmed that the curve is downward in five out of seven radicals, i.e., against M^n+^ (Figure 4A), DPPH^•^ (Figure 4B), LOO^•^ (Figure 4C), ABTS^•+^ (Figure 4D), and HO^•^RBD_50_ (Figure 4G), which agreed with the WFI trends. Notably, limonene was the most abundant component (32.20%, Supplementary Materials Table S2) in Cedar fruit EO, the most potent metal chelator EO, which is likely to decrease the potency (Table 1). On the other hand, the component was missing in Cade EO, the most potent EO against DPPH^•^ (Table 1), and was present in low amounts in the Oregano (0.54%) and Red thyme (0.29%) EOs; thus, it was not high enough to disturb the high potencies of the EOs during lipid peroxidation and against ABTS^•+^, respectively (Table 1). Moreover, limonene was also missing in YY EO, which was very active for OH^•^, while protecting salmon DNA (Table 1 and Supplementary Materials Table S2). Nevertheless, in the PD plots, limonene shows also a double aspect; in the case of the OH^•^ radical (Figure 4E), it can be seen that when the limonene percentage is more than 20%, its influence is positively related to the EC_50_, in agreement with the observation that the potency of Red thyme EO (Table 1) could be even higher. In contrast, in the case of ROO^•^, the curve for limonene was fully ascending and, therefore, a positive partial dependence curve (Figure 4F). Considering that limonene is a nonphenolic terpenoid with a cyclohexadiene structure, it could exert some activity against ROO^•^ similar to that of γ-terpinene in terms of donating its methylene C-H while neutralizing the radical [1]. The low amount of limonene found in CCP EO (0.20%) likely did not contribute to decreasing its high potency against ROO^•^. In addition, all EOs active against ROO^•^ and having limonene as one of the most abundant components should also be associated with cytotoxic antioxidant activity [1]. For the above observation, the prediction of ML6 regarding the biological activity of limonene was quite consistent.

#### 3.3.2. The Contribution of Linalool to the Antioxidant Activity

Compared to limonene, similar results were obtained from the inspection of the WFIs for linalool, where the compound exerted a negative impact on EOs’ antioxidant activities in all ML models but the one obtained against the OH^•^ radical (Figure 3). The PD plots, except for the OH^•^-related (Supplementary Materials Figure S60E) confirmed a descending curve and thus an associated negative importance for this component to the antioxidant activity as its concentration increases. In that sense, the highest potency of Red thyme EO against the OH^•^ radical (Table 1) could be associated with the limited presence of linalool (5.16%, Supplementary Materials Table S2). Some affinity of linalool to neutralize the OH^•^ could be associated with its ability to donate either alcoholic or methylene protons through the HAT mechanism [1,143]. However, a difficult-to-interpret graph was obtained from the model for describing the EOs’ protective activities on a DNA double-strand against the OH^•^ radical (i.e., based on the HO^•^RBD_50_ values) where an alternating line trend can be seen when the concentration of this component is between 0 and 20%. The descending curve was in part a reflection of 10.36% of linalool in the composition of YY EO (Supplementary Materials Table S2). Moreover, it was interesting that linalool was indicated to contribute negatively to the neutralization of DPPH^•^ (Figure S60B), despite the reported potency of this component against that radical [1,143], as well as against the ABTS^•+^ (Supplementary Materials Figure S60D), confirming its hydroxyl portion has not been a reaction center in either HAT [138] or SPLET fashion [139]. While linalool has been absent in the chemical composition of Cade EO (Table 1, Supplementary Materials Table S2,), thus not diminishing its highest potency against DPPH^•^ (Supplementary Materials Figure S60C), its presence in Red thyme EO’s chemical composition in 5.16% (Supplementary Materials Table S2) likely contributed to lower potency against ABTS^•+^ (Table 1). Additionally, having just a minor positive impact on lipid peroxidation inhibition, if occurring in more than 40% (Supplementary Materials Figure S60C) (in agreement with the low quantity of 2.43%, Supplementary Materials Table S2, within Oregano EO, Table 1), and no positive impact against ROO^•^ (Supplementary Materials Figure S60F) (following low quantity of 3.19%, Supplementary Materials Table S2, in the chemical composition of CCP EO, Table 1) whatsoever, linalool has not been able to act as an antioxidant in a termination-enhancing antioxidant activity fashion [1] (somehow in disagreement with the reported affinity of linalool to act against ROO^•^ [143]).

#### 3.3.3. The Contribution of Carvacrol to EOs’ Antioxidant Activity

The presence of carvacrol was classified as positive in all models except for the model obtained on ROO^•^ (RBD_50_) (Figure 3). A very interesting feature of carvacrol is its ability to influence metal ions chelation in a concentration-dependent way (Figure 5A), despite the fact that carvacrol, as a monohydroxylated, might not to be able to form the complex with Fe^2+^ [144,145,146] as its phenolic portion alone could not be strong enough to hold the interaction. Nevertheless, Horvathova et al. confirmed some chelating potential [147], and carvacrol was not contained in the Cedar fruit EO (Table 1 and Supplementary Materials Table S2), the most potent EO in the metal chelation. On the other hand, carvacrol contributed to the neutralization against either DPPH^•^ (Figure 5B) or OH^•^ (Figure 5E), likely due to its phenolic proton by PCET mechanism [137]. Still, it was not found in the chemical composition of Cade EO (Supplementary Materials Table S2), the most potent against DPPH^•^ (Table 1), but it was found in Red thyme EO at 7.20% (Table 1 and Supplementary Materials Table S2), the most potent OH^•^ scavenger EO sample. However, while protecting the DNA double-strand from OH^•^, carvacrol was not listed in the 20 most important and influential components by model ML5 (Table 2 and Figure 5G). In fact, it was missing in YY EO (Table 1 and Supplementary Materials Table S2). On the other hand, the neutralization potential of carvacrol-containing EO toward ABTS^•+^ (Figure 5D) is likely associated with the SPLET mechanism [139], an effect associated with Red thyme EO. Moreover, by inspecting the partial dependence trend (Figure 5C), the presence of this component is particularly important and positive against LOO^•^. Indeed, the most potent EO against the LOO^•^, Oregano EO (Table 1), contained carvacrol at a percentage as high as 76.54% (Supplementary Materials Table S2), which was not surprising, as the phenolic group of carvacrol guaranteed the activity of containing EOs against the LOO^•^ in a chain-breaking antioxidants fashion [1,147,148]. However, regarding the affinity against the ROO^•^ (Figure 5F), this component was not listed among the 20 most important and influential components regarding antioxidant activity against that free radical (Figure 5F), proving carvacrol’s inability to stop the ROO^•^ by means of termination-enhancing antioxidant activity [1].

#### 3.3.4. The Contribution of Thymol to the Antioxidant Activity

Both thymol and eugenol were associated with positive WFI in most models, thus representing two important positive components for antioxidant activity (Figure 3). However, thymol, like carvacrol, and as a structural isomer, does not appear among the ML model’s recognized 20 most important components for the chelation of metal ions (Supplementary Materials Figure S61A), which was somehow in disagreement with the report in which thymol should better stabilize the Fe^2+^ than carvacrol [147,148]. Thymol was likewise not among the 20 most important components related to the neutralization of of ROO^•^ (Supplementary Materials Figure S61F), likely due to the lack of termination-enhancing antioxidant activity features [1]. On the contrary, thymol was proposed as positively modulating the LOO^•^ radical neutralization (Supplementary Materials Figure S61C), likely owing to chain-breaking antioxidant features [1,146]. However, it was not contained in the Oregano EO, the most active EO against LOO^•^ radical neutralization (Table 1 and Table S2, Supporting Information). Regarding other radicals’ activities, thymol (Supplementary Materials Figure S61B,D,E,G) was calculated with a similar profile to that of carvacrol, although not contained (Supplementary Materials Table S2) in Cade and YY EOs (Table 1) but found in even 66.31% (Supplementary Materials Table S2) in Red thyme EO (Table 1).

#### 3.3.5. The Contribution of Eugenol to the Antioxidant Activity

Considering the eugenol, ML7 is the only model in which its presence seems to have a slightly negative WFI (Supplementary Materials Figure S62G). In fact, in YY EO, only 0.58% of eugenol was found (Table 1 and Supplementary Materials Table S2), and it was not listed among the 20 most important compounds. Its chelating features (Supplementary Materials Figure S62A) could be attributed partially to both phenolic and methoxy portions [146]. Although eugenol was not contained in the Cedar fruit EO (Table 1 and Supplementary Materials Table S2), its ability to neutralize DPPH^•^ (Supplementary Materials Figure S62B), OH^•^ (Supplementary Materials Figure S62E), or ABTS^•+^ (Supplementary Materials Figure S62D) radicals is due to its phenolic proton via PCET [137] or SPLET [139] mechanisms, while against LOO^•^ (Supplementary Materials Figure S62C), eugenol contained in EO might act in a chain-breaking antioxidants fashion [1,148,149]. In CCP EO (Table 1), eugenol was contained at 34.63% (Supplementary Materials Table S2) and, likely differently from carvacrol and thymol, it managed to neutralize the ROO^•^ (Supplementary Materials Figure S62F) via the allylic double bond’s termination-enhancing antioxidant activity [1].

#### 3.3.6. The Contribution of Chrysanthone to the Antioxidant Activity

Chrysanthone was classified as a positive component for the EOs’ antioxidant activities against the DPPH^•^ (Supplementary Materials Figure S63C) and the OH^•^ (Supplementary Materials Figures S63E and S63G) by models ML2 and ML5, respectively (Table 2). Its activity was likely due to the phenolic protons through a PCET mechanism [137]. In contrast, a negative trend was associated with metal ions chelation and neutralization of ABTS^•+^, LOO^•^, and ROO^•^ (Supplementary Materials Figure S63). Interestingly, chrysanthone was correctly not listed in the composition of the most potent EOs against the listed radicals (Table 1 and Supplementary Materials Table S2).

#### 3.3.7. The Contribution of Eucalyptol to the Antioxidant Activity

Overall, contradictory results between radicals were obtained for some components, such as eucalyptol, α-pinene, caryophyllene, and *p*-cymene, (Figure 3). These may be due either to different antioxidant activity against various free radicals from the same component or imperfect model accuracy. In particular, eucalyptol was classified as strongly influencing metal ions chelation (Supplementary Materials Figure S64A), but it was not contained in the most active Cedar fruit EO (Table 1 and Supplementary Materials Table S2). Eucalyptol was also found with a definite positive trend for the OH^•^ neutralization, leading to damaging either 2′-deoxyribose (Supplementary Materials Figure S64E) or DNA (Supplementary Materials Figure S64G), although it was contained at a very low percentage (0.25%) in the Red thyme EO (Table 1 and Supplementary Materials Table S2), in agreement with previous findings [149]. As for the remaining redox species (Supplementary Materials Figure S64B–D,F), the negative slopes of partial dependence plots curves confirmed the lack of potency for eucalyptol in agreement with its absence in Cade, Oregano, CCP, and YY EOs (Table 1 and Supplementary Materials Table S2).

#### 3.3.8. The Contribution of α-Pinene to the Antioxidant Activity

As for α-pinene, the component exerted a positive modulation on antioxidant activity only against OH^•^ radicals (Figure 3), being a constituent of Red thyme EO at 0.38% (Supplementary Materials Table S2). As proposed by the ML5 and ML7 models, to fully contribute to the protection of either 2’-deoxy-D-ribose or a DNA double-strand, at least 7.72% and 8% of α-pinene would be required in the EO mixture (Supplementary Materials Figure S65E,G), respectively. The component’s contribution was likely due to its ability to undergo photooxidation in the presence of OH^•^, thus neutralizing the radical [150]. Surprisingly, α-pinene was calculated to have a negative trend contribution targeting both LOO^•^ (Supplementary Materials Figure S65C) and ROO^•^ (Supplementary Materials Figure S65G), as confirmed by 0.37% and 0.25% of α-pinene in Oregano EO and CCP EO, respectively (Table 1 and Supplementary Materials Table S2). As for the descendent partial dependence curve, while targeting DPPH^•^ (Supplementary Materials Figure S65C), it corresponds to the previously reported very low potency of α-pinene against distinct radicals [151] and in agreement with its lack of presence in Cade EO (Table 1 and Supplementary Materials Table S2).

#### 3.3.9. The Contribution of Caryophyllene to the Antioxidant Activity

Regarding caryophyllene, remarkably, it was the only abundant compound (Supplementary Materials Table S2) in all of the above-listed potent EOs (Table 1). Still, its contribution was recognized as positive only for ROO^•^ and OH^•^ (Supplementary Materials Figure S66F,G). Thus, its role in preventing DNA damage can be associated with its 4.01% and 15.47% in CCP and YY EO, respectively (Supplementary Material Table S2). Its positive trend in the activity against ROO^•^ (Supplementary Material Figure S66F) could be associated with the termination-enhancing antioxidant mechanism, donating the methylene proton of the endocyclic double bond [1]; its interaction with the OH^•^ (Supplementary Materials Figure S66G) was expected [152] due to the known ability to make a covalent bond while reacting with alkoxy radicals [153]. As for the metal ions chelating ability (Supplementary Material Figure S66A), caryophyllene could have a positive impact if present between 5% and 15%. It also had no impact on the neutralization of DPPH^•^, LOO^•^, ABTS^•+^, or ABTS^•+^ (Table S2, Supplementary Material Figure S66B–E), as it was poorly present in Cade EO, Oregano EO, and Red thyme EO (Supplementary Material Table S2).

#### 3.3.10. The Contribution of *p*-Cymene to the Antioxidant Activity

The EOs’ potency contribution of *p*-cymene was positive while neutralizing DPPH^•^, ABTS^•+^, and OH^•^ (Figure 3). The low affinity of *p*-cymene toward DPPH^•^ (Supplementary Material Figure S67B) could be associated with the fact that hydrocarbons are very rarely H-donors to DPPH^•^ [136]. This component was not found in the Cade EO (Table 1 and Supplementary Material Table S2). It was also surprisingly negatively contributing to the neutralization of LOO^•^ (Supplementary Material Figure S67C), given that its occurrence in the Oregano EO was 6.80% (Table 1 and Supplementary Material Table S2). Yet, *p*-cymene, as expected [154], was predicted to be of high importance for ABTS^•+^, which could be associated with its presence at 10.46% in the chemical composition of Red thyme EO (Supplementary Material Table S2 and Figure S67D). On the other hand, despite not being expected to occur [154], the hydroxyl radical addition onto *p*-cymene was surprising and could be considered an inevitable limitation of the model ML6 (Supplementary Material Figure S67E). The negative trend of p-cymene in model ML7 suggested its failure to counteract OH^•^, which could be associated with its incapability of reaching the DNA environment (Supplementary Material Figure S67G). The strong negative trend visible in the partial dependence plot is consistent with the lack of metal-chelating features for *p*-cymene (Supplementary Material Figure S67A) required to chelate the Fe^2+^ [146]. Moreover, the negative influence against ROO^•^ (Supplementary Material Figure S67F) of *p*-cymene was likewise expected due to the absence of chain-breaking features [1,146], in agreement with its absence in the CCP EO (Table 1 and, Supplementary Materials Table S2).

### 3.4. Antioxidant Activity of Targeted EOs In Vivo

**Scheme 2:**
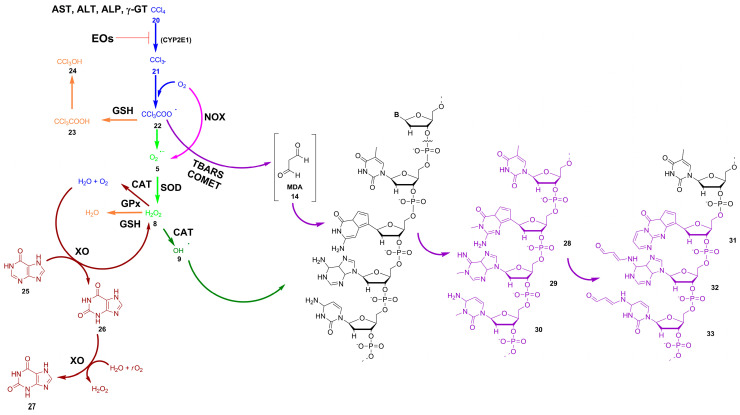
The inter-correlation of methods used for monitoring/neutralizing in vitro and in vivo, emphasizing the emergence of ROS, by means of the EOs herein. The in vivo assays: (blue pathway, bold arrows) CCl_4_ (**20**), trichloromethyl (CCl_3_^•^, **21**), trichloromethylperoxy radical (CCl_3_OO^•^, **22**); TBARS assay (purple path, bold arrows): malondialdehyde (**14**); SOD assay (light green pathway, bold arrows): H_2_O_2_ (**8**); CAT assay (dark green pathway, bold arrows): OH^•^ (**9**); GSH assay (orange pathway, bold arrows): CCl_3_OOH (**23**), CCl_3_OH (**24**); XO assay (brown path, bold arrows): hypoxanthine (**25**), xanthine (**26**), uric acid (**27**); comet assay: (purple and dark green paths, bold arrows) M_1_G (**28**), M_1_A (**29**), M_1_C (**30**), *N*^2^-oxopropenyl-dG (**31**), *N*^2^-oxopropenyl-dA (**32**), *N*^2^-oxopropenyl-dC (**33**).

YY and CCP EOs, the EOs exerting high potencies against each redox species in in vitro assays, (Table 1 and Table 3), were promptly evaluated against the cellular damage caused by intraperitoneal (i.p.) administration of 1 mL/kg body weight (bwt) of CCl_4_ [155] to adult Wistar rats [103] (Scheme 2, blue pathway, **23**). To counteract the hazardous behavior of CCl_4_, EOs were i.p.administered in a separate protocol simultaneously with CCl_4_ [156] in three different concentrations (1, 200, and 400 mg/kg bwt) in agreement with the in vitro results discussed above. Upon administration, CCl_4_ is likely to be oxidized by rat CYP2E1 for the production of trichloromethyl radical (*r*CCl_3_^•^, Scheme 2, blue pathway, **24**), an intermediate metabolite that is further oxidized to trichloromethylperoxy radical (*r*CCl_3_OO^•^, Scheme 2, blue pathway, **25**) [155,157,158]. The *r*CCl_3_OO^•^ could initiate the peroxidation of polyunsaturated fatty acids in the membrane of either hepatocytes or kidneys (Table 1, Table 2, Table 3 and Table 4: group II), leading to *r*MDA formation (Scheme 2, blue pathway, **14**), whereas its excess may generate cellular *r*O_2_^•−^ (Scheme 2, blue pathway, **5**) and H_2_O_2_ (Scheme 2, blue pathway, **5**) [155,157,158].

Therefore, the efficacies of EOs as lipid peroxidation inhibitors and consequent redox-stress-reducing mediators in vivo within either the liver or kidneys were assessed by monitoring the concentrations of the thiobarbituric-acid-reactive substance (*r*TBARS) [118] and reduced glutathione (*r*GSH) [119], as well as through the catalytic activities of superoxide dismutase (*r*SOD) [116] and catalase (*r*CAT) [118] (Scheme 2, blue pathway, Table 1 and Table 3, respectively). The *r*TBARS [117], *r*GSH [119], and *r*CAT [118] values were expressed through the total protein content (*r*TP) [120]. On the other hand, the radical-induced damage of the hepatocyte membrane was elucidated through the liver toxicity markers, i.e., catalytic activities of aspartate transaminase (*r*AST) [159,160] and alanine transaminase (*r*ALT) [123], while damage to the bile was assessed through the catalytic activity of alkaline phosphatase (*r*ALP) [124] and γ-glutamyltransferase (*r*γ-GT) [125] (Table 2). Finally, the early indicators of oxidative-stress-associated chronic kidney disease (CDK) [127] after CCl_4_-induced intoxication and its prevention by EOs were investigated by monitoring the catalytic activities of renal toxicity markers, such as xanthine oxidase (*r*XO) [126] and NADPH oxidase (*r*NOX) [127], as well as the protectors of the kidneys, nitric oxidase (*r*NO) [128] and glutathione peroxidase (*r*GPx) [48].

#### 3.4.1. Liver Redox Status

In general, within all of the observed results, dose-dependent effects were observed upon administration of either YY EO or CCP EO.

##### The rTBARS Concentrations

On the liver hepatocytes membrane, *r*CCl_3_OO^•^ radical can induce the formation of malondialdehyde (*r*MDA), a toxic metabolite major side-product of lipid peroxidation (Scheme 2, blue pathway, **14**) [134]. Therefore, the hepatoprotective properties of YY and CCP EOs were determined upon the complexation of *r*MDA with thiobarbituric acid, leading to the formation of *r*TBARS (Scheme 2), whose increased concentration is related to membrane damage [157]. While counterbalancing the CCl_4_ administration consequence and metabolites, an interesting dose-dependent trend of increased hepatoprotective effects with the rise of EOs’ concentration and any hepatic or renal toxicity was noted. Therefore, to avoid any redundancy, only the results in the concentration of 400 mg/kg bwt are discussed herein, whereas the effects of EOs in the lower quantities are reported in the Supplementary Materials. Administration of CCl_4_ resulted in a 1.77-fold increase in *r*TBARS concentration in liver homogenates compared to the negative control (compare *r*TBARS of group II with I in Table 4). On the other hand, either YY or CCP EO dose-dependently decreased *r*TBARS concentration, where the CCP EO was more potent than the YY EO (compare V and VIII with I in Table 3), and the *r*TBARS was only 26.61% and 8.87% of that for the negative control and 15.00% and 5.00% of that caused by CCl_4_ (compare V and VIII with II in Table 3). In agreement with the in vitro data and ML models, it can be speculated that the slightly higher affinity of CCP vs. YY EO to counteract the *r*CCl_3_OO^•^ in vivo could be attributed to the percentages profile of eugenol (34.63% in CCP EO vs. 0.58% in YY EO, Table 3), limonene (0.20% in CCP EO vs. 0.00% in YY EO, Table 3), and caryophyllene (4.01% in CCP EO vs. 15.47% in YY EO, Table 3), components all recognized as positively important by the ML3 model for the LOO^•^.

##### The *r*SOD Catalytic Activities

The CCP and YY EOs were also of notable efficacy in modulating the catalytic activity of *r*SOD for the *r*CCl_3_OO^•^ to *r*CCl_3_^•^ and *r*O_2_^•−^ conversion (Scheme 2, light green pathway, bold arrows, **24** and blue pathway, **5**) [155]. Moreover, the produced *r*O_2_^•-^ likely acts as a secondary source of lipid peroxidation, and *r*SOD catalyzes the dismutation of *r*O_2_^•−^ into oxygen (O_2_) and hydrogen peroxide (H_2_O_2_) (Scheme 2, blue pathway, 8); as a consequence, any decrease in *r*SOD catalytic activity is associated with undergoing oxidative stress [116]. This scenario was confirmed by a 1.91-fold decrease in *r*SOD catalytic activity compared to the negative control (compare group II with I in Table 4) because of the produced *r*CCl_3_OO^•^ breakdown. Either CCP EO or YY EO were very efficient (compare groups V and VII with I in Table 4) in recovering up to 97.72% and 99.43% of the *r*SOD catalytic activity, respectively.

##### The *r*CAT Catalytic Activities

The in-vivo-generated *r*H_2_O_2_ is promptly decomposed to *r*OH^•^ (Scheme 2, dark green pathway, bold arrows, **9**) and this could lead to 8-oxo-dG, thymineglycol, and 6-hydroxy-5,6-dihydrocytosine (Scheme 2, dark green path, bold arrows **17**, **18** and **19**) [140]. Considering that the decrease in the catalytic activity of *r*CAT induces oxidative stress in tissue [118], either CCP EO or YY EO were found efficient in positively modulating the catalytic activity of *r*CAT, so that an excess of *r*H_2_O_2_ is promptly decomposed to water and molecular oxygen (Scheme 2, blue pathway). Thus, CCl_4_ caused a moderate decrease (58%) in the catalytic activity of *r*CAT (compare group II with I in Table 4), which was associated with kidney necrosis [156]. The maximal protection from *r*H_2_O_2_ was obtained with the higher concentration of either EO, leading to complete recovery of the native *r*CAT activity (compare groups V and VIII with I in Table 4).

##### The *r*GSH Concentrations

CCP and YY EOs were also assessed through the monitoring of *r*GSH concentration (Scheme 2, dark orange path, bold arrows), as its decrease indicates oxidative stress [155]. Hence, rGSH may provide two-level protection, either by reducing the *r*CCl_3_OO^•^ to form *r*CCl_3_OOH (Scheme 2, orange pathway, bold arrows, **23**) and *r*CCl_3_OH (Scheme 2, orange pathway, bold arrows, **24**) [155] or by converting the excess of *r*H_2_O_2_ to *r*H_2_O (Scheme 2, orange pathway, bold arrows) [160]. Therefore, after the administration of CCl_4_ in liver homogenates, a lower rGSH concentration (2.21-fold) was observed than in the negative control (compare group II with I in Table 4). The administration of either YY or CCP EO at the concentration of 400 mg/kg bwt resulted in expressive hepatoprotective features against CCl_4_ (compare groups V and VIII with I in Table 4), as 400 mg/kg bwt of the two essential oils rescued about 32% and 83% of the lost *r*GSH concentration for the two EOs, respectively. In the presence of CCl_4_, the YY or CCP EO restored about 63% and 91% of the basal *r*GSH, respectively.

#### 3.4.2. The Hepatocytes Toxicity Status

##### The *r*AST and *r*ALT Catalytic Activities

The disruption of hepatocytes’ membrane upon the CCl_4_-induced damage was further monitored through the catalytic activities of *r*AST and *r*ALT, whereas the condition of the bile duct was determined by means of the catalytic activities of *r*ALP and *r*γ-GT (Table 5). The increments of *r*AST and *r*ALT catalytic activities (compare group II with I in Table 5) by 7.64- and 5.71-fold, respectively, were indicative of the hepatocyte’s membrane damage by CCl_4_ administration, revealing the early symptoms of hepatocellular toxicity and cirrhosis [159], whereas the increase of *r*ALP and *r*γ-GT catalytic activities (compare group II with I in Table 5) by 2.06- and 4.94-fold, respectively, indicated bile problems [161]. Complementary with the data for *r*TBARS and *r*SOD (Table 4), both YY and CCP EO downgraded the catalytic activities of liver and bile toxicity markers with the rise of concentration.

The *r*AST and *r*ALT catalytic activities associated to YY and CCP EOs, indicated that both EOs at the highest EO concentrationsreatly prevented the CCl_4_-induced hepatotoxicity (compare groups V and VII with I in Table 5). In particular, YY and CCP EOs slowed down the *r*AST CCl_4_ enhanced activity from about 7.64- to 1.79- and 2.22-fold, respectively. More remarkably, the YY and CCP EOs almost completely restored the *r*ALT activity, being 5% and 27% faster when co-administered with CCl_4_ [161]. While increasing the dosage, it seemed that YY EO was overall better tolerated by hepatocytes than CCP EO.

##### The *r*ALP and γ-GT Catalytic Activities

Similar dose-response protective features of either YY or CCP EO were verified in bile, where the highest concentration displayed similar potencies (compare group V and VIII with I in Table 5), reducing the CCl_4_-induced *r*ALP catalytic activity increase of more 200% to about 121% and 125%, respectively. In contrast, for γ-GT, CCP EO maintained the catalytic activity of the enzyme more effectively than YY EO (compare groups V and VIII with I in Table 5), rescuing almost 95% of the γ-GT activity enhancement induced by CCl_4_.

#### 3.4.3. Kidneys’ Redox Status

##### The *r*TBARS Concentrations

Through the analysis of kidney homogenates, it was determined that *r*MDA had also been generated within the cell membranes of the kidneys’ nephrons, for which it was measured as a 4.05-fold increase in the *r*TBARS concentration in samples saturated with CCl_4_ compared to the negative control (compare group II with I in Table 6), indicating a higher rate of toxicity than that in the liver [157]. Either YY or CCP EO at the highest tested concentration showed full potential for the kidneys’ recovery (compare groups V and VII with I in Table 6), holding the CCl_4_ *r*TBARS’s augmented value of about 71% and 63%, respectively. Data contained in Table 4 and Table 6 generally indicate that EOs were more selective for the liver system than for the kidneys, considering the lipid peroxidation stopping, which was also confirmed by the kidneys’ *r*SOD catalytic activities.

##### The *r*SOD Catalytic Activities

EOs were of notable efficiency while restoring the catalytic activity of *r*SOD, which was about 61% of the basal value after the application of CCl_4_ (compare groups II and I in Table 6), confirming the high-intensity lipid peroxidation of the nephrons’ cell membrane. At the highest dosage, CCP EO overpowered YY EO, raising the *r*GSH concentration 1.88-fold (vs. 1.76-fold of YY EO) and 114-fold (vs. 1.07-fold of YY EO) above the CCl_4_ and negative control groups (compare groups V and VIII with II and I in Table 6).

##### The rCAT Catalytic Activities

An excess of *r*H_2_O_2_ in the kidneys, such as that caused by CCl_4_, was confirmed by lowering the *r*CAT catalytic activity to about 30% of the basal value (compare group II with I in Table 6). Either YY or CCP EO was shown to be very effective in neutralizing this effect by avoiding any *r*CAT catalytic activity loss. Although the maximal protection was achieved at the highest tested concentration, a significant effect was also visible at the lowest concentration (Table 6); for the YY or CCP EO, more than 86% and 89% of the *r*CAT activity was retained, respectively. At higher concentrations, the catalytic activity was almost completely rescued.

##### The rGSH Concentration

An evaluation of EOs’ impact on kidneys’ *r*GSH revealed that they have been capable of restoring the marker’s concentration downregulated by CCl_4_ to about 50% of the basal concentration (compare group II with I in Table 6). The maximal dosage restored the *r*GSH’s concentration almost entirely, to about 93 and 99% of the basal *r*GSH concentration (compare groups V and VIII with I in Table 6), i.e., 1.17- and 2.01-fold higher *r*GSH concentration than that caused by CCl_4_ (compare groups V and VIII with II in Table 6), with CCP EO being slightly more potent than YY EO.

#### 3.4.4. Chronic Kidney Disease Markers

Kidneys’ homogenates were further examined, as oxidative stress in kidneys is a biochemical hallmark of chronic kidney disease that could influence the progression of renal function deterioration and the onset of major systemic co-morbidities involving cardiovascular diseases [127]. Hence, the administration impact of either CCl_4_/YY EO or CCl_4_/CCP EO on renal redox status was elaborated on the level of ROS inducers *r*XO and *r*NOX, as well as on the level of renal redox defensive mechanisms, like *r*NO and *r*GPx.

##### The *r*XO Catalytic Activities

Upon the rats’ kidneys toxication with CCl_4_, *r*XO likely employed the *r*O_2_ to act as a secondary co-factor, alongside NAD^+^, to catalyze the oxidation of hypoxanthine to xanthine and then xanthine to uric acid (Scheme 2, brown path, bold arrows, **25**, **26** and **27**), ending in an additional generation of *r*O_2_^•−^ [125]. The process was quantified by the significative 2.76-fold upregulation of *r*XO’s catalytic activity (compare group II with I in Table 7). The *r*XO’s catalytic activity augmentation was prevented by either YY or CCP EO at the highest tested concentrations (compare groups V and VIII with I in Table 7) by reducing to about 43% and 51% the CCl_4_-increased *r*XO, respectively, which was about 18% and 40% higher than the activity recorded in the presence of only olive oil, as YY EO is more potent than CCP EO in neutralizing the effect of CCl_4_.

##### The *r*NOX Catalytic Activities

NADPH oxidase (NOX), usually considered as a marker for cardiovascular, hemorrhagic-shock-induced organ injury [162,163], hepatic ischemia/reperfusion (I/R) injury [161], and lipopolysaccharide-induced lung injury [164], catalyzes the conversion of O_2_ into O_2_^•−^ (Scheme 2, pink path, bold arrows, **5**) within kidneys’ endoplasmic reticulum and may lead to the progression of oxidative stress. Therefore, a significant upregulation of NOX (alongside the downregulation of SOD in CKD) represents an indicator of renal insufficiency [164]. In fact, *r*NOX did respond intensively to the oxidative damage caused by CCl_4_, as the positive control sample was characterized by a 3.67-fold higher enzyme catalytic activity than the negative control (compare group II with I in Table 7).

Such a *r*NOX response agrees with the EOs’ rescued catalytic activity of *r*CAT, which can decompose *r*H_2_O_2_ generated by *r*SOD from the *r*O_2_^•−^ decomposition. In this scenario, *r*NOX then captures *r*O_2_ and converts it into *r*O_2_^•−^ (Table 7: II) and, in the presence of either YY or CCP EO (compare groups V and VIII in Table 7), the *r*NOX catalytic activity was not higher than 30% of the basal value, thus reducing to 35% and 32% the *r*NOX catalytic activity caused by CCl_4_ administration, with a global damage recovery of more than 65%.

##### The *r*NO Concentrations

The CCl_4_-generated *r*O_2_^•−^ in kidneys was administered, formed by either *r*XO or *r*NOX and quantified by the decrease in *r*NO concentration to only 38% of the negative control value (compare group II with I in Table 7). Still, both YY and CCP EO as potential supplements managed to restore the *r*NO concentration efficiently, reaching full potential at the highest concentrations (compare groups V and VIII with I in Table 7), showing 95.12 and 91.34% of the basal *r*NO concentration, respectively, with a slightly higher potency of YY EO.

##### The GPx Catalytic Activities

Glutathione peroxidase (GPx, Scheme 2, orange pathway, bold arrows) shares its affinity with CAT to decompose an excess of produced H_2_O_2_; therefore, a decrease in its catalytic activity is associated with oxidative stress [127]. Therefore, *r*GPx was involved in protecting the kidneys from *r*H_2_O_2_ and was downregulated by CCl_4_ to only 48% of the basal catalytic activity (compare group II with I in Table 7). Both YY and CCP EO managed to gradually recuperate *r*GPx’s catalytic activity, whereas YY EO performed slightly better (compare groups V and VIII with I in Table 7). The rescue of 96% and 88% of the basal *r*GPx reduced catalytic activity caused by CCl_4_ was recorded for YY EO and CCP EO, respectively.

### 3.5. EOs Antigenotoxic Activity In Vivo

In parallel with the in vivo antioxidant evaluation, the Wistar rats’ liver and kidney samples were used for determining the selected YY and CCP EOs’ antigenotoxic activities by means of the comet assay (Table 6, Table 7 and Table 8) [128,165]. While, to the best of the authors’ knowledge, no genotoxicity and/or antigenotoxicity study has been reported for YY EO, the CCP EO isolated from the bark of *Cinnamomum burmanii* was already assessed using doxorubicin-induced Chinese Hamster Ovary (CHO-K1) cells using micronucleus assay [166], Ames Salmonella reversion assay, *Bacillus subtilis* DNA-repair test (Rec-assay), *Escherichia coli* WP2uvrA reversion test [167], and *Drosophila melanogaster* Somatic Mutation and Recombination Test [168]. Herein, the antigenotoxicity of EOs was evaluated against the *r*CCl_3_OO^•^ that could cause the formation of *r*MDA and transform the DNA into the aberrant adducts, like M_1_G, M_1_A, and M_1_C (Scheme 2, purple pathway, bold arrows, **28**, **29** and **30**), which root the transversions and transitions of bases and, upon the quantitative ring-opening, ultimately form *N*^2^-oxopropenyl-dG, *N*^2^-oxopropenyl-dA, and *N*^2^-oxopropenyl-dC adducts (Scheme 2, purple pathway, bold arrows, **31**, **32** and **33**), leading to the DNA–DNA inter-strand cross-links or DNA–protein inter-strand crosslinks [169] to which the comet assay is sensitive [128,165].

#### 3.5.1. Antigenotoxicity in Liver

As for the comet assay, both EOs have been compared with olive oil, characterized by a larger number of comets with no DNA damage (T_0_) and a small number of comets associated with very low damage (T_1_) (Table 8, I), or CCl_4_, whose administration caused a significant increase in DNA damage (compare group II with I in Table 7), with a 5.62-fold higher total comet score (TCS) value. The co-administration of EOs with CCl_4_ showed the YY EO as the more potent DNA protector; at the highest tested concentration, it provided TCS values that remained as low as 1.47-fold with respect to the negative control (compare group V with I in Table 8), corresponding to only 26.20% of the value associated with the administration of CCl_4_, with a percentage reduction of DNA damage (%R) of 89.8% and an absence of comet types T_3_ and T_4_. Although the CCP EO delivered 1.8-fold higher TCS than in negative control group (Table 8), 31.24% of the TCS was associated with CCl_4_ alone, and there was a %R of 83.6% alongside the absence of comet types T_3_ and T_4_. The higher potency of YY EO to protect the DNA in vivo from the damage induced by *r*CCl_3_OO^•^ (i.e., the ROO^•^-type radical) compared to that observed for CCP EO could be, in part, associated with the higher percentage of caryophyllene (Table 3, 15.47 vs. 4.01%, respectively), which had the expressive affinity to neutralize the ROO^•^ in vitro (Figure 3). Eugenol, which was 59.71-fold more abundant in CCP EO (Table 3), was a less significant feature against ROO^•^ than caryophyllene. Yet, as previously noted, this speculation needs to be confirmed in further studies.

#### 3.5.2. Antigenotoxicity in Kidneys

The potential protective features of selected EOs against the CCl_4_-induced DNA damage were also assessed in the kidney cells of albino Wistar rats (Table 8) and compared to the negative control group, in which most of the comets showed no DNA damage (type T_0_) and a few of them indicated very low damage (type T_1_) (Table 9, I), or CCl_4_ alone, which significantly increased TSC in kidney cells compared with the CCl_4_-free group (Table 9, II vs. I) due to large-damage comets’ presence (viz. types T_3_ and T_4_). Differently from what was observed in the case of the liver, CCP EO was more potent than YY EO at the highest tested concentration. Both YY and CCP EO showed lower DNA protection (compare data in Table 8 and Table 9), dropping the TCSs to 1.69- and 1.31-fold higher values than the basal ones (compare groups V and VIII with I in Table 9), reducing to about 27% and 21% the TCSs induced by CCl_4_, and causing the %Rs equal to 87.2 and 94.4%, respectively.

## 4. Conclusions

Extensive in vitro assays on a list of 61 EOs were performed, leading to interesting and unique results. Many EO samples were shown to be able to modulate the TAC, to chelate the transition metal ions, and to neutralize the DPPH^•^, LOO^•^, ABTS^•+^, and OH^•^. The titled EOs also protected the DNA from damage induced by ROO^•^ or OH^•^. Among the tested EOs, those obtained from YY and CCP showed the best antioxidant profile and were selected for in vivo investigations.

By correlating the chemical compositions of 61 commercial EOs with their in vitro experimentally determined abilities against M^n+^, DPPH^•^, LOO^•^, ABTS^•+^, OH^•^ (either on the level of nucleotides or a DNA double-strand), or ROO^•^ (protecting the DNA) by means of the machine learning (ML) classification algorithms, like SVM, RF, GB, DT, and KNN, various QCAR models were generated, yielding the most important features to be limonene, linalool, carvacrol, eucalyptol, α-pinene, thymol, caryophyllene, *p*-cymene, eugenol, and chrysanthone, and characterizing their either positive or negative contributions through features importance plots as well as indicating the percentages of features within the EO mixtures required to either positively or negatively contribute to the listed antioxidant activities through the Partial Dependencies plots. The features’ predicted contributions were in good agreement with their previously reported experimentally defined biological profiles while exerting antioxidant potential, thus validating the models’ accuracies. As an added value of this research, all of the generated models can be freely used for the prediction of any untested EO for its antioxidant potencies, as a part of the AI4EssOil project (https://www.ai4essoil.com/front/, accessed on 20 September 2023).

The features’ potential contributions in vivo were thereafter anticipated upon the administration of the most potent EOs (from YY and CCP) in adult Wistar rats against the redox and genotoxic damage caused by CCl_4_. Experimental in vivo data strongly suggested that both YY and CCP EO represent potential new and effective antioxidant agents able to protect the cell from injuries caused by toxicants, such as CCl_4_. Further studies are in due course to investigate the isolated components indicated by the ML models as possible new antioxidant compounds.

## Figures and Tables

**Table 1:** Antioxidant activity of examined EOs and referent compounds against specific radicals.

EOs IDs	M ^n+ a^	DPPH^• b^	LOO^• c^	ABTS^•+ d^	OH^• e^	ROO^• f^	OH^• g^
Official Latin names of biological source/sources	EC_50_^h^ (µg/mL)					ROO-RBD_50_^i^ (µg/mL)	OH-RBD_50_^j^ (µg/mL)
Chamomile Morocco *Cladanthus mixtus* (L.) Chevall.	>1	>1	>1	>1	>1	137.71 ± 0.53 ^k^	143.71 ± 0.37
Clary sage *Salvia sclarea* L.	>1	>1	>1	>1	>1	176.43 ± 0.32	168.31 ± 0.95
Sage oil *Salvia officinalis* L.	>1	>1	>1	>1	>1	142.56 ± 0.46	144.13 ± 0.32
Red thyme *Thymus praecox* Opiz “coccineus”	>1	0.230 ± 0.23	0.007 ± 0.006	0.003 ± 0.003	0.010 ± 0.06	NA	NA ^l^
Tea tree *Melaleuca alternifolia* (Maiden & Betche) Cheel	>1	>1	>1	>1	>1	23.33 ± 0.13	46.14 ± 0.46
Melissa *Melissa officinalis* L.	>1	>1	>1	>1	>1	172.46 ± 0.57	129.13 ± 0.34
Mountain pine Pinus mugo Turra	>1	>1	>1	>1	>1	NA	110.92 ± 0.42
Geranium Bourbon Pelargonium x asperum Ehrh. ex Willd.	>1	>1	>1	>1	>1	NA	105.16 ± 0.11
Oregano *Origanum* *vulgare* L.	>1	0.110 ± 0.46	0.005 ± 0.005	0.004 ± 0.003	0.024 ± 0.011	184.90 ± 0.23	155.00 ± 0.54
Ylang-ylang *Cananga odorata* (Lam.) Hook. f. & Thomson	0.84 ± 0.47	0.630 ± 0.45	0.32 ± 0.26	0.76 ± 0.12	0.35 ± 0.14	64.12 ± 0.43	134.11 ± 0.67
Coriander *Coriandrum* *sativum* L.	>1	>1	>1	>1	>1	NA	143.54 ± 0.36
Lavender *Lavandula* *angustifolia* Mill.	>1	>1	>1	>1	>1	44.10 ± 0.54	209.76 ± 0.33
Myrtle *Myrtus communis* L.	>1	>1	>1	>1	>1	113.05 ± 0.54	NA
Garlic *Allium sativum* L.	>1	>1	>1	>1	>1	195.62 ± 0.31	NA
Cardamom *Elettaria cardamomum* (L.) Maton	>1	>1	>1	>1	>1	NA	202.89 ± 0.65
Mandarin *Citrus reticulata* Blanco	>1	>1	>1	>1	>1	197.55 ± 0.54	NA
Hyssop *Hyssopus* *officinalis* L.	>1	>1	>1	>1	>1	141.42 ± 0.56	31.07 ± 0.64
Grapefruit *Citrus paradisi* Macfad.	1.58 ± 0.76	>1	>1	>1	>1	126.74 ± 0.21	103.10 ± 0.47
Lemongrass *Cymbopogon* *citratus* (DC.) Stapf	>1	0.720 ± 0.37	0.45 ± 0.18	0.65 ± 0.38	0.58 ± 0.27	52.85 ± 0.43	80.93 ± 0.41
Siberian pine *Abies sibirica* Ledeb.	>1	>1	>1	>1	>1	108.20 ± 0.11	132.61 ± 0.48
Camphor *Cinnamomum* *camphora* (L.) J. Presl	>1	>1	>1	>1	>1	NA	133.43 ± 0.73
Cade *Juniperus* *oxycedrus* L.	>1	0.007 ± 0.006	0.007 ± 0.003	0.017 ± 0.01	0.023 ± 0.014	NA	197.70 ± 0.51
Cedar leaves *Thuja occidentalis* L.	>1	0.550 ± 0.21	0.60 ± 0.34	0.35 ± 0.14	0.27 ± 0.12	96.44 ± 0.34	150.16 ± 0.38
Ginger *Zingiber officinale* Roscoe	>1	>1	>1	>1	>1	8.29 ± 0.54	56.34 ± 0.24
Cumin *Cuminum cyminum* L.	>1	>1	>1	>1	>1	168.58 ± 0.13	173.92 ± 0.43
Patchouli *Pogostemon* *cablin* Benth.	>1	>1	>1	>1	>1	63.56 ± 0.43	136.71 ± 0.47
Orange bitter *Citrus* *aurantium* L.	>1	>1	>1	>1	>1	NA	25.70 ± 0.32
Eucalyptus *Eucalyptus globulus* Labill.	0.78 ± 0.42	>1	>1	>1	>1	NA	NA
Pine Silvestre Natural *Pinus sylvestris* L.	>1	>1	>1	>1	>1	NA	213.0 ± 0.24
Bergamot *Citrus limon* (L.) Osbeck (syn. Citrus × bergamia Risso & Poit.)	1.72 ± 0.84	>1	>1	>1	>1	NA	58.70 ± 0.54
Juniper *Juniperus* *communis* L.	>1	>1	>1	>1	>1	NA	198.26 ± 0.67
Birch *Betula lenta* L.	>1	0.014 ± 0.012	0.014 ± 0.007	0.08 ± 0.04	0.12 ± 0.11	205.29 ± 0.41	NA
Fennel *Foeniculum vulgare* Mill.	0.75 ± 0.26	>1	>1	>1	>1	NA	NA
Cedar fruit *Citrus medica* L.	0.63 ± 0.37	>1	>1	>1	>1	54.50 ± 0.42	95.01 ± 0.54
Lemon *Citrus limon* (L.) Osbeck	1.28 ± 0.79	>1	>1	>1	>1	NA	136.18 ± 0.32
Roman chamomile *Chamaemelum* *nobile* (L.) All.	1.34 ± 0.49	>1	>1	>1	>1	NA	65.90 ± 0.21
Savory *Satureja hortensis* L.	1.46 ± 0.49	0.110 ± 0.03	0.18 ± 0.09	0.065 ± 0.012	0.032 ± 0.007	NA	NA
Rosemary *Rosmarinus* *officinalis* L.	1.39 ± 0.67	0.375 ± 0.25	0.23 ± 0.11	0.26 ± 0.11	0.42 ± 0.24	NA	NA
Ceylon cinnamon peel Cinnamomum verum J. Presl	0.95 ± 0.78	0.023 ± 0.11	0.032 ± 0.006	0.125 ± 0.45	0.078 ± 0.06	60.99 ± 0.51	78.36 ± 0.43
*Eucalyptus* *globulus*	1.26 ± 0.56	>1	>1	>1	>1	NA	54.84 ± 0.11
Orange sweet *Citrus sinensis* (L.) Osbeck	>1	>1	>1	>1	>1	NA	NA
Niaouly *Melaleuca* *quinquenervia* (Cav.) S.T.Blake	>1	>1	>1	>1	>1	138.65 ± 0.46	195.34 ± 0.45
Artemisia *Artemisia vulgaris* L.	>1	>1	>1	>1	>1	197.40 ± 0.31	30.40 ± 0.46
Cajeput Melaleuca cajuputi Powell	>1	>1	>1	>1	>1	86.55 ± 0.41	NA
Black pepper *Piper nigrum* L.	>1	>1	>1	>1	>1	NA	NA
White thyme *Thymus vulgaris* L.	>1	0.120 ± 0.06	0.18 ± 0.12	0.089 ± 0.022	0.046 ± 0.031	204.18 ± 0.63	88.03 ± 0.23
Marjoram *Origanum* *marjorana* L.	>1	>1	>1	>1	>1	324.83 ± 0.41	60.02 ± 0.64
Clove *Syzygium* *aromaticum* (L.) Merr. & L. M. Perry	>1	0.008 ± 0.003	0.024 ± 0.007	0.0173 ± 0.009	0.098 ± 0.034	163.54 ± 0.46	88.36 ± 0.54
Cypress *Cupressus* *sempervirens* L.	>1	>1	>1	>1	>1	NA	99.03 ± 0.63
Nutmeg natural Myristica fragrans Houtt.	>1	0.840 ± 0.34	0.54 ± 0.27	0.66 ± 0.28	0.72 ± 0.24	NA	NA
Peppermint *Mentha piperita* L.	>1	>1	>1	>1	>1	NA	NA
Lemon verbena *Aloysia citriodora* Palau	>1	>1	>1	>1	>1	159.59 ± 0.54	95.99 ± 0.24
Basil *Ocimum* *basilicum* L.	>1	>1	>1	>1	>1	194.21 ± 0.31	NA
Palmarosa *Cymbopogon* *martini* (Roxb.) W.Watson	>1	>1	>1	>1	>1	NA	NA
Laurel *Laurus nobilis* L.	>1	0.680 ± 0.12	0.75 ± 0.36	>1	>1	NA	78.70 ± 0.54
Natural anise pure *Pimpinella anisum* L.	>1	>1	>1	>1	>1	NA	83.71 ± 0.15
*Incense* *Boswellia* spp.	>1	>1	>1	>1	>1	NA	83.96 ± 0.78
Mentha suaveolens (Sicily) Mentha suaveolens Ehrh.	>1	>1	>1	0.89 ± 0.24	0.56 ± 0.12	NA	145.21 ± 0.34
Coridotthymus capitatus (Sicily) *Thymbra capitata* (L.) Cav. (syn. Thymus capitatus (L.) Hoffmanns. & Link)	>1	0.256 ± 0.18	0.232 ± 0.14	0.09 ± 0.06	0.22 ± 0.17	215.90 ± 0.45	24.26 ± 0.41
*Thymus vulgaris* (Sicily) *Thymus vulgaris* L.	>1	0.990 ± 0.35	0.69 ± 0.36	0.84 ± 0.15	0.56 ± 0.50	232.47 ± 0.51	200.03 ± 0.53
*Origanum hirtum* (Sicily) *Origanum vulgare* subsp. hirtum (Link) Ietsw.	>1	0.570 ± 0.27	0.70 ± 0.28	0.39 ± 0.17	0.12 ± 0.08	NA	NA
AA ^m^	NA	5.180 ± 0.46	>100	15.47 ± 0.36	>100	NA	NA
BHT ^n^	NA	13.25 ± 0.41	3.12 ± 0.98	10.16 ± 1.41	30.14 ± 0.24	NA	NA
EDTA ^o^	3.18 ± 0.59	NA	NA	NA	NA	NA	NA
Q ^p^	NA	NA	NA	NA	NA	NA	NA

^a^ Metal-ion-chelating capacity; ^b^ Neutralization of DPPH radical; ^c^ Interruption of lipid peroxidation; ^d^ Neutralization of ABTS cation radical; ^e^ Neutralization of hydroxyl radical; ^f^ Protection of DNA against the damage induced by the alkoxy radical; ^g^ Protection of DNA against the damage induced by the hydroxyl radical; ^h^ Effective concentration that neutralizes the 50% of free radical; ^i^ Concentration that stimulates 50% of relative electrophoretic bands density (RBD) increase (viz. protection) of DNA damaged by alkoxy radical; ^j^ Concentration that stimulates 50% of relative electrophoretic bands density (RBD) increase (viz. protection) of DNA damaged by hydroxyl radical; ^k^ The measurement is presented with standard deviation from three independent experiments; ^l^ Not available; ^m^ Ascorbic acid; ^n^ Butylated hydroxytoluene; ^o^ Ethylenediaminetetraacetic acid; ^p^ Quercetin.

**Table 2:** Parameters and statistical coefficients of the best ML models.

Model	ML1	ML2	ML3	ML4	ML5	ML6	ML7
Free Radical	M^n+^	DPPH^•^	LOO^•^	ABTS^+•^	OH^•^	ROO-RBD_50_s	OH-RBD_50_
Threshold	1.752	0.63	0.19	0.097	0.111	164.548	157.662
Algorithm	GB	SVM	SVM	KNN	RF	GB	KNN
N level	3	2	0	0	1	1	3
Scaling	1	0	0	0	0	1	1
PCA	0.9	0.0	0.99	0.6	0.8	0.7	0.8
nMCC_Pred_	0.81	0.90	0.98	0.93	0.95	0.73	0.68
nMCC_CV_	0.60	0.90	0.91	0.80	0.82	0.68	0.58

**Table 3:** The Ylang-ylang and Ceylon cinnamon peel Eos’ chemical compositions.

Ylang-Ylang EO	Ceylon Cinnamon Peel EO
Component (%)	Component (%)
eucalyptol (0.24)	eugenol (34.63)
eugenol (0.58)	linalool (3.19)
linalool (10.36)	α-pinene (0.25)
methyl benzoate (2.06)	acetyleugenol (1.29)
*p*-methylanisole (3.28)	α-phellandrene (0.34)
α-copaene (1.24)	*o*-cymene (0.69)
farnesyl acetate	β-phellandrene (0.51)
τ-cadinol (1.69)	α-terpineol (0.31)
δ-cadinene (3.84)	α-copaene (0.48)
farnesol (2.61)	limonene (0.20)
geraniol (1.50)	tetradecanal (0.46)
nerol acetate (12.21)	*trans*-3-phenyl-2-propenal (49.11)
δ-cadinene (3.84)	β-isosafrole (0.89)
farnesol (2.61)	2-methoxycinnamaldehyde (0.20)
geraniol (1.50)	caryophyllene oxide (0.21)
nerol acetate (12.21)	caryophyllene (4.01)
caryophyllene (15.47)	humulene (0.63)
(*E*)-β-farnesene (13.92)	cinnamyl ester acetic acid (2.61)
humulene (4.29)	
cinnamyl ester acetic acid (0.96)	
germacrene D (18.23)	
*trans*-calamenene (0.30)	
γ-cadinene (2.77)	
β-elemene (0.48)	
β-ylangene (0.47)	

**Table 4:** Total protein content, catalytic activities of enzymes, and concentrations of antioxidant markers in rat liver exposed to different doses of YY and CCP EO co-administered with CCl_4_.

Group	*r*TP (g/L)	*r*TBARS (nmol/mg)	*r*SOD (U/mg)	*r*CAT (U/mg)	*r*GSH (mg/g)
I	5.56 ± 0.14 ^a†^	2.48 ± 0.02 ^†^	5.27 ± 0.95 ^†^	120.87 ± 0.15 ^†^	32.24 ± 0.12 ^†^
II	6.97 ± 0.12 *	4.40 ± 0.14 *	2.76 ± 0.36 *	70.10 ± 0.3 *	14.62 ± 0.16 *
III	18.94 ± 0.17 *^†^	1.84 ± 0.08 *^†^	4.95 ± 0.74 *^†^	78.93 ± 0.08 *^†^	15.76 ± 0.20 *^†^
IV	15.82 ± 0.23 *^†^	0.84 ± 0.09 *^†^	5.07 ± 0.63 *^†^	113.24 ± 0.10 *^†^	16.96 ± 0.12 *^†^
V	12.90 ± 0.34 *^†^	0.66 ± 0.08 *^†^	5.15 ± 0.69 *^†^	118.15 ± 0.15 *^†^	20.26 ± 0.13 *^†^
VI	8.07 ± 0.24 *^†^	2.01 ± 0.02 *^†^	4.83 ± 0.44 *^†^	73.47 ± 0.23 *^†^	17.15 ± 0.14 *^†^
VII	14.56 ± 0.17 *^†^	0.94 ± 0.03 *^†^	5.11 ± 0.78 *^†^	111.76 ± 0.22 *^†^	21.62 ± 0.02 *^†^
VIII	14.14 ± 0.41 *^†^	0.22 ± 0.06 *^†^	5.24 ± 0.85 ^†^	118.87 ± 0.11 *^†^	29.32 ± 0.18 *^†^

^a^ Values represent mean ± SEM from three independent experiments; n = 5 rats per group; I—Negative control, 1 mL/kg bwt olive oil, i.p.; II—Positive control, 1 mL/kg bwt of CCl_4_, 1:1 mixture in olive oil, i.p.; III—1 mg/kg bwt of YY EO + 1 mL/kg bwt of CCl_4_, i.p.; IV—200 mg/kg bwt of YY EO + 1 mL/kg bwt of CCl_4_, i.p.; V—400 mg/kg bwt of YY EO + 1 mL/kg bwt of CCl_4_, i.p.; VI—1 mg/kg bwt of CCP EO + 1 mL/kg bwt of CCl_4_, i.p.; VII—200 mg/kg bwt of CCP EO + 1 mL/kg bwt of CCl_4_, i.p.; VIII—400 mg/kg bwt of CCP EO + 1 mL/kg bwt of CCl_4_, i.p.; * *p* < 0.05 when compared with the negative control group; ^†^ *p* < 0.05 when compared with the positive control group.

**Table 5:** Catalytic activities of serum biochemical markers within the rats exposed to different doses of YY and CCP EO and CCl_4_.

Group	*r*AST (U/L)	*r*ALT (U/L)	*r*ALP (U/L)	*r*γ-GT (U/L)
I	7.88 ± 0.14 ^a†^	36.66 ± 0.02 ^†^	142.45 ± 0.15 ^†^	3.35 ± 0.32 ^†^
II	60.18 ± 0.12 *	209.31 ± 0.14 *	292.82 ± 0.31 *	16.54 ± 0.35 *
III	38.60 ± 0.17 *^†^	91.33 ± 0.08 *^†^	246.10 ± 0.08 *^†^	10.24 ± 0.37 *^†^
IV	24.37 ± 0.23 *^†^	41.65 ± 0.09 *^†^	210.66 ± 0.10 *^†^	6.45 ± 0.43 *^†^
V	14.07 ± 0.34 *^†^	38.35 ± 0.08 *^†^	172.18 ± 0.15 *^†^	4.43 ± 0.21 *^†^
VI	20.60 ± 0.24 *^†^	80.62 ± 0.02 *^†^	279.78 ± 0.23 *^†^	9.43 ± 0.34 *^†^
VII	19.87 ± 0.17 *^†^	51.80 ± 0.03 *^†^	259.88 ± 0.22 *^†^	6.12 ± 0.36 *^†^
VIII	17.50 ± 0.41 *^†^	46.66 ± 0.06 *^†^	179.26 ± 0.11 *^†^	4.06 ± 0.21 *^†^

^a^ Values represent mean ± SEM from three independent experiments; n = 5 rats per group; I—Negative control, 1 mL/kg bwt olive oil, i.p.; II—Positive control, 1 mL/kg bwt of CCl_4_, 1:1 mixture in olive oil, i.p.; III—1 mg/kg bwt of YY EO + 1 mL/kg bwt of CCl_4_, i.p.; IV—200 mg/kg bwt of YY EO + 1 mL/kg bwt of CCl_4_, i.p.; V—400 mg/kg bwt of YY EO + 1 mL/kg bwt of CCl_4_, i.p.; VI—1 mg/kg bwt of CCP EO + 1 mL/kg bwt of CCl_4_, i.p.; VII—200 mg/kg bwt of CCP EO + 1 mL/kg bwt of CCl_4_, i.p.; VIII—400 mg/kg bwt of CCP EO + 1 mL/kg bwt of CCl_4_, i.p.; * *p* < 0.05 when compared with the negative control group; ^†^ *p* < 0.05 when compared with the positive control group.

**Table 6:** Total protein content, catalytic activities of enzymes, and concentrations of antioxidant markers in rat kidneys exposed to different doses of YY EO, CCP EO, and CCl_4_.

Group	*r*TP (g/L)	*r*TBARS (nmol/mg)	*r*SOD (U/mg)	*r*CAT (U/mg)	*r*GSH (mg/g)
I	3.91 ± 0.14 ^a†^	0.43 ± 0.02 ^†^	5.11 ± 0.32 ^†^	170.78 ± 0.15 ^†^	39.58 ± 0.12 ^†^
II	5.78 ± 0.12 *	1.74 ± 0.14 *	3.12 ± 0.43 *	52.54 ± 0.3 *	19.67 ± 0.16 *
III	3.96 ± 0.17 ^†^	3.85 ± 0.08 *^†^	4.26 ± 0.23 *^†^	147.05 ± 0.08 *^†^	24.64 ± 0.20 *^†^
IV	2.59 ± 0.23 *^†^	3.72 ± 0.09 *^†^	4.44 ± 0.25 *^†^	166.27 ± 0.10 *^†^	31.78 ± 0.12 *^†^
V	3.22 ± 0.34 *^†^	1.23 ± 0.08 *^†^	5.49 ± 0.69 *^†^	168.98 ± 0.15 *^†^	37.18 ± 0.13 *^†^
VI	3.16 ± 0.24 *^†^	2.77 ± 0.02 *^†^	4.12 ± 0.32 *^†^	152.46 ± 0.23 *^†^	21.20 ± 0.14 *^†^
VII	5.76 ± 0.17 *	2.73 ± 0.03 *^†^	4.32 ± 0.34 *^†^	164.16 ± 0.22 *^†^	27.83 ± 0.02 *^†^
VIII	4.64 ± 0.41 *^†^	1.09 ± 0.06 *^†^	5.85 ± 0.85 *^†^	165.52 ± 0.11 *^†^	39.22 ± 0.18 ^†^

^a^ Values represent mean ± SEM from three independent experiments; n = 5 rats per group; I—Negative control, 1 mL/kg bwt olive oil, i.p.; II—Positive control, 1 mL/kg bwt of CCl_4_, 1:1 mixture in olive oil, i.p.; III—1 mg/kg bwt of YY EO + 1 mL/kg bwt of CCl_4_, i.p.; IV—200 mg/kg bwt of YY EO + 1 mL/kg bwt of CCl_4_, i.p.; V—400 mg/kg bwt of YY EO + 1 mL/kg bwt of CCl_4_, i.p.; VI—1 mg/kg bwt of CCP EO + 1 mL/kg bwt of CCl_4_, i.p.; VII—200 mg/kg bwt of CCP EO + 1 mL/kg bwt of CCl_4_, i.p.; VIII—400 mg/kg bwt of CCP EO + 1 mL/kg bwt of CCl_4_, i.p.; * *p* < 0.05 when compared with the negative control group; ^†^ *p* < 0.05 when compared with the positive control group.

**Table 7:** The catalytic activities of enzymes, and the concentrations of antioxidant markers in rat kidneys exposed to different doses of YY EO, CCP EO, and CCl_4_.

Group	*r*XO (U/L)	*r*NOX (U/mg Protein)	*r*NO (µmol/L)	*r*GPx (U/mg Protein)
I	18.14 ± 0.58 ^a†^	1.40 ± 0.05 ^†^	30.15 ± 0.10 ^†^	0.25 ± 0.09 ^†^
II	50.01 ± 0.64 *	5.14 ± 0.24 *	11.58 ± 0.11 *	0.12 ± 0.03 *
III	37.64 ± 0.31 *^†^	4.62 ± 0.15 *^†^	20.95 ± 0.31 *^†^	0.15 ± 0.04 ^†^
IV	30.57 ± 0.14 *^†^	2.11 ± 0.31 *^†^	27.98 ± 0.22 *^†^	0.20 ± 0.03 *^†^
V	21.36 ± 0.95 *^†^	1.82 ± 0.03 *^†^	28.68 ± 0.20 *^†^	0.24 ± 0.02 ^†^
VI	33.03 ± 0.60 *^†^	3.56 ± 0.17 *^†^	16.41 ± 0.09 *^†^	0.18 ± 0.01 *^†^
VII	29.16 ± 0.36 *^†^	1.99 ± 0.09 *^†^	21.16 ± 0.15 *^†^	0.21 ± 0.03 ^†^
VIII	25.31 ± 0.22 *^†^	1.66 ± 0.26 *^†^	27.54 ± 0.30 *^†^	0.22 ± 0.06 ^†^

^a^ Values represent mean ± SEM from three independent experiments; n=5 rats per group; I—Negative control, 1 mL/kg bwt olive oil, i.p.; II—Positive control, 1 mL/kg bwt of CCl_4_, 1:1 mixture in olive oil, i.p.; III—1 mg/kg bwt of YY EO + 1 mL/kg bwt of CCl_4_, i.p.; IV—200 mg/kg bwt of YY EO + 1 mL/kg bwt of CCl_4_, i.p.; V—400 mg/kg bwt of YY EO + 1 mL/kg bwt of CCl_4_, i.p.; VI—1 mg/kg bwt of CCP EO + 1 mL/kg bwt of CCl_4_, i.p.; VII—200 mg/kg bwt of CCP EO + 1 mL/kg bwt of CCl_4_, i.p.; VIII—400 mg/kg bwt of CCP EO + 1 mL/kg bwt of CCl_4_, i.p.; * *p* < 0.05 when compared with the negative control group; ^†^ *p* < 0.05 when compared with the positive control group.

**Table 8:** The DNA-protective activity of different concentrations of YY and CCP EO against DNA damage induced by CCl_4_ in the livers of albino Wistar rats.

Groups	Comet Types ^a^					Total Comet Score (TCS)	%R ^b^
	T_0_	T_1_	T_2_	T_3_	T_4_		
I	72.5 ± 0.37 ^c^	27.5 ± 0.51	NO ^d^	NO	NO	27.5 ± 0.41 ^†^	NA ^e^
II	NO	61.5 ± 0.32	26.2 ± 0.81	8.5 ± 0.12	3.8 ± 0.72	154.6 ± 0.53 *	NA
III	54.2 ± 0.82	21.5 ± 0.81	19.5 ± 0.23	4.8 ± 0.56	NO	74.9 ± 0.55 *^†^	62.7
IV	55.3 ± 0.61	31.6 ± 0.23	11.2 ± 0.32	1.9 ± 0.51	NO	59.7 ± 0.34 *^†^	74.7
V	67.8 ± 0.90	23.9 ± 0.41	8.3 ± 0.71	NO	NO	40.5 ± 0.84 *^†^	89.8
VI	49.4 ± 0.23	35.5 ± 0.92	11.9 ± 1.24	3.2 ± 0.12	NO	68.9 ± 0.51 *^†^	67.4
VII	66.1 ± 0.17	17.7 ± 0.13	14.1 ± 0.82	2.1 ± 0.85	NO	52.2 ± 0.72 *^†^	80.6
VIII	61.9 ± 0.11	27.9 ± 0.72	10.2 ± 0.84	NO	NO	48.3 ± 0.92 *^†^	83.6

^a^ Comet types defined as T_0_, T_1_, T_2_, T_3_, and T_4_ (no or very low damage, low, medium, and long DNA migration, and the highest level of DNA damage, respectively); ^b^ %R, percentage reduction of DNA damage; ^c^ Values represent mean ± SEM from three independent experiments; n = 5 rats per group; ^d^ Not observed; ^e^ Not available; I—Negative control, 1 mL/kg bwt olive oil, i.p.; II—Positive control, 1 mL/kg bwt of CCl_4_, 1:1 mixture in olive oil, i.p.; III—1 mg/kg bwt of YY EO + 1 mL/kg bwt of CCl_4_, i.p.; IV—200 mg/kg bwt of YY EO + 1 mL/kg bwt of CCl_4_, i.p.; V—400 mg/kg bwt of YY EO + 1 mL/kg bwt of CCl_4_, i.p.; VI—1 mg/kg bwt of CCP EO + 1 mL/kg bwt of CCl_4_, i.p.; VII—200 mg/kg bwt of CCP EO + 1 mL/kg bwt of CCl_4_, i.p.; VIII—400 mg/kg bwt of CCP EO + 1 mL/kg bwt of CCl_4_, i.p.; * *p* < 0.05 when compared with the negative control group; ^†^ *p* < 0.05 when compared with the positive control group.

**Table 9:** The DNA-protective activity of different concentrations of YY and CCP EO against DNA damage induced by CCl_4_ in kidneys of albino Wistar rats.

Groups	Comet Types ^a^					Total Comet Score (TCS)	%R ^b^
	T_0_	T_1_	T_2_	T_3_	T_4_		
I	78.2 ± 0.6 ^c^	21.8 ± 0.54	NO ^d^	NO	NO	21.8 ± 0.52 ^†^	NA ^e^
II	20.6 ± 0.72	38.3 ± 0.81	27.2 ± 0.11	9.6 ± 0.24	4.3 ± 0.83	138.7 ± 0.42 *	NA
III	54.5 ± 0.25	36.4 ± 0.54	9.1 ± 0.32	NO	NO	54.6 ± 0.6 *^†^	71.9
IV	64.1 ± 0.8	29.3 ± 0.68	6.6 ± 0.51	NO	NO	42.5 ± 0.21 *^†^	82.3
V	67.5 ± 1.24	28.2 ± 0.62	4.3 ± 0.21	NO	NO	36.8 ± 0.38 *^†^	87.2
VI	62.3 ± 0.25	31.5 ± 0.71	6.2 ± 0.34	NO	NO	43.9 ± 0.51 *^†^	81.1
VII	75.1 ± 0.12	18.3 ± 0.36	4.5 ± 0.50	2.1 ± 0.24	NO	33.6 ± 0.12 *^†^	89.9
VIII	71.5 ± 0.9	28.5 ± 0.40	NO	NO	NO	28.5 ± 0.31 ^†^	94.4

^a^ Comet types defined as T_0_, T_1_, T_2_, T_3_, and T_4_ (no or very low damage, low, medium, and long DNA migration, and the highest level of DNA damage, respectively); ^b^ %R, percentage reduction of DNA damage; ^c^ Values represented mean ± SEM from three independent experiments; n = 5 rats per group; ^d^ Not observed; ^e^ Not available; I—Negative control, 1 mL/kg bwt olive oil, i.p.; II—Positive control, 1 mL/kg bwt of CCl_4_, 1:1 mixture in olive oil, i.p.; III—1 mg/kg bwt of YY EO + 1 mL/kg bwt of CCl_4_, i.p.; IV—200 mg/kg bwt of YY EO + 1 mL/kg bwt of CCl_4_, i.p.; V—400 mg/kg bwt of YY EO + 1 mL/kg bwt of CCl_4_, i.p.; VI—1 mg/kg bwt of CCP EO + 1 mL/kg bwt of CCl_4_, i.p.; VII—200 mg/kg bwt of CCP EO + 1 mL/kg bwt of CCl_4_, i.p.; VIII—400 mg/kg bwt of CCP EO + 1 mL/kg bwt of CCl_4_, i.p.; * *p* < 0.05 when compared with the negative control group; ^†^ *p* < 0.05 when compared with the positive control group.

## Data Availability

All of the experimental results in vitro and in vivo can be obtained upon request from Milan Mladenović. All of the Python notebooks considering the ML models can be obtained upon request from Rino Ragno.

## Supplementary Materials

The following Supplementary Materials can be downloaded at: https://www.mdpi.com/article/10.3390/antiox12101815/s1, Table S1. Essential oils previously investigated as antioxidant and their main chemical components. Table S2. Chemical composition of the tested essential oils chemical. Table S3. Total antioxidant capacity of examined EOs. Table S4. DNA-protective potential of selected 61 commercial essential oils on peroxyl-radical-induced DNA damage. Table S5. DNA-protective potential of selected 61 commercial essential oils on hydroxyl-radical-induced DNA damage. Table S6. List of dataset pretreatment parameters settings randomly varied during ML hyperparameters optimization. Table S7. List of the initial thresholds used for the ML models for each antioxidant evaluation. Table S8. List of hyperparameters settings used for the ML models through random search optimization performed at 100 and 1000 iterations. Table S9. List of hyperparameters settings used for the ML models through random search optimization performed at 10,000 and 100,000 iterations. **Final ML Models Development. 1. M^n+^** 1.1. 100 random iterations. Table S10. Coarse best models obtained for each classifier. Table S11. Hyperparameters associated with models **MN1–MN5**. The list is reported as Python dictionaries. 1.2. 1000 random iterations. Table S12. Coarse best models obtained for each classifier. Table S13. Hyperparameters associated with models **MN6–MN9**. The list is reported as Python dictionaries. 1.3. 10,000 random iterations. Table S14. Refined best models obtained for each classifier. Table S15. Hyperparameters associated with models **MN10–MN13**. The list is reported as Python dictionaries. 1.4. 100,000 random iterations. Table S16. Refined best models obtained for each classifier. Table S17. Hyperparameters associated with models **MN14–MN16**. The list is reported as Python dictionaries. 2. **DPPH^•^** 2.1. 100 random iterations. Table S18. Coarse best models obtained for each classifier. Table S19. Hyperparameters associated with models **DPPH1–DPPH5**. The list is reported as Python dictionaries. 2.2. 1000 random iterations. Table S20. Coarse best models obtained for each classifier. Table S21. Hyperparameters associated with models **DPPH6–DPPH10**. The list is reported as Python dictionaries. 2.3. 10,000 random iterations. Table S22. Refined best models obtained for each classifier. Table S23. Hyperparameters associated with models **DPPH11–DPPH13**. The list is reported as Python dictionaries. 2.4. 100,000 random iterations. Table S24. Refined best models obtained for each classifier. Table S25. Hyperparameters associated with models **DPPH14–DPPH16**. The list is reported as Python dictionaries. 3. **LOO^•^** 3.1. 100 random iterations. Table S26. Coarse best models obtained for each classifier. Table S27. Hyperparameters associated with models **LOO1–LOO5**. The list is reported as Python dictionaries. 3.2. 1000 random iterations. Table S28. Coarse best models obtained for each classifier. Table S29. Hyperparameters associated with models **LOO6–LOO10**. The list is reported as Python dictionaries. 3.3. 10,000 random iterations. Table S30. Refined best models obtained for each classifier. Table S31. Hyperparameters associated with models **LOO10–LOO14**. The list is reported as Python dictionaries. 3.4. 100,000 random iterations Table S32. Refined best models obtained for each classifier. Table S33. Hyperparameters associated with models **LOO15–LOO17**. The list is reported as Python dictionaries. 4. **ABTS^+•^** 4.1. 100 random iterations. Table S34. Coarse best models obtained for each classifier. Table S35. Hyperparameters associated with models **ABTS1–ABTS5.** The list is reported as Python dictionaries. 4.2. 1000 random iterations. Table S36. Coarse best models obtained for each classifier. Table S37. Hyperparameters associated with models **ABTS6–ABTS10**. The list is reported as Python dictionaries. 4.3. 10,000 random iterations. Table S38. Refined best models obtained for each classifier. Table S39. Hyperparameters associated with models **ABTS11–ABTS14**. The list is reported as Python dictionaries. 4.4. 100,000 random iterations. Table S40. Refined best models obtained for each classifier. Table S41. Hyperparameters associated with models **ABTS15–ABTS17**. The list is reported as Python dictionaries. 5. **OH^•^** 5.1. 100 random iterations. Table S42. Coarse best models obtained for each classifier. Table S43. Hyperparameters associated with models **OH1–OH5**. The list is reported as Python dictionaries. 5.2. 1000 random iterations. Table S44. Coarse best models obtained for each classifier. Table S45. Hyperparameters associated with models **OH6–OH10**. The list is reported as Python dictionaries. 5.3. 10,000 random iterations. Table S46. Refined best models obtained for each classifier. Table S47. Hyperparameters associated with models **OH11–OH14**. The list is reported as Python dictionaries. 5.4. 100,000 random iterations. Table S48. Refined best models obtained for each classifier. Table S49. Hyperparameters associated with models **OH15–OH16**. The list is reported as Python dictionaries. 6. **ROO-RBD_50_s** 6.1. 100 random iterations. Table S50. Coarse best models obtained for each classifier. Table S51. Hyperparameters associated with models **ROO1–ROO5**. The list is reported as Python dictionaries. 6.2. 1000 random iterations. Table S52. Coarse best models obtained for each classifier. Table S53. Hyperparameters associated with models **ROO6–ROO9**. The list is reported as Python dictionaries. 6.3. 10,000 random iterations. Table S54. Refined best models obtained for each classifier. Table S55. Hyperparameters associated with models **ROO10–ROO13**. The list is reported as Python dictionaries. 6.4. 100,000 random iterations. Table S56. Refined best models obtained for each classifier. Table S57. Hyperparameters associated with models **ROO14–ROO16**. The list is reported as Python dictionaries. 7. **OH-RBD_50_** 7.1. 100 random iterations. Table S58. Coarse best models obtained for each classifier. Table S59. Hyperparameters associated with models **OH-RBD1**-**OH-RBD5**. The list is reported as Python dictionaries. 7.2. 1000 random iterations. Table S60. Coarse best models obtained for each classifier. Table S61. Hyperparameters associated with models **OH-RBD6**-**OH-RBD10**. The list is reported as Python dictionaries. 7.3. 10,000 random iterations. Table S62. Refined best models obtained for each classifier. Table S63. Hyperparameters associated with models **OH-RBD11**-**OH-RBD13**. The list is reported as Python dictionaries. 7.4. 100,000 random iterations. Table S64. Refined best models obtained for each classifier. Table S65. Hyperparameters associated with models **OH-RBD14**-**OH-RBD16**. The list is reported as Python dictionaries. Figure S1. Protective effect of Chamomile Morocco EO against peroxyl- (A) and hydroxyl- (B) radicals-induced DNA damage. DNA from salmon sperm (lane 1, negative control), DNA damage control (lane 2, positive control), quercetin (lane 3, 100 μg/mL, standard), essential oils in concentrations of 25, 50, 100, 200, and 400 μg/mL (lanes 4, 5, 6, 7, and 8). * *p* < 0.05 when compared with the negative control group; ^†^ *p* < 0.05 when compared with the positive control group. Figure S2. Protective effect of Clary sage EO against peroxyl- (A) and hydroxyl- (B) radicals-induced DNA damage. DNA from salmon sperm (lane 1, negative control), DNA damage control (lane 2, positive control), quercetin (lane 3, 100 μg/mL, standard), essential oils in concentrations of 25, 50, 100, 200, and 400 μg/mL (lanes 4, 5, 6, 7, and 8). * *p* < 0.05 when compared with the negative control group; ^†^ *p* < 0.05 when compared with the positive control group. Figure S3. Protective effect of Sage EO against peroxyl- (A) and hydroxyl- (B) radicals-induced DNA damage. DNA from salmon sperm (lane 1, negative control), DNA damage control (lane 2, positive control), quercetin (lane 3, 100 μg/mL, standard), essential oils in concentrations of 25, 50, 100, 200, and 400 μg/mL (lanes 4, 5, 6, 7, and 8). * *p* < 0.05 when compared with the negative control group; ^†^ *p* < 0.05 when compared with the positive control group. Figure S4. Protective effect of Red thyme EO against peroxyl- (A) and hydroxyl- (B) radicals-induced DNA damage. DNA from salmon sperm (lane 1, negative control), DNA damage control (lane 2, positive control), quercetin (lane 3, 100 μg/mL, standard), essential oils in concentrations of 25, 50, 100, 200, and 400 μg/mL (lanes 4, 5, 6, 7, and 8). * *p* < 0.05 when compared with the negative control group; ^†^ *p* < 0.05 when compared with the positive control group. Figure S5. Protective effect Tea tree EO against peroxyl- (A) and hydroxyl- (B) radicals-induced DNA damage. DNA from salmon sperm (lane 1, negative control), DNA damage control (lane 2, positive control), quercetin (lane 3, 100 μg/mL, standard), essential oils in concentrations of 25, 50, 100, 200, and 400 μg/mL (lanes 4, 5, 6, 7, and 8). * *p* < 0.05 when compared with the negative control group; ^†^ *p* < 0.05 when compared with the positive control group. Figure S6. Protective effect of Melissa EO against peroxyl- (A) and hydroxyl- (B) radicals-induced DNA damage. DNA from salmon sperm (lane 1, negative control), DNA damage control (lane 2, positive control), quercetin (lane 3, 100 μg/mL, standard), essential oils in concentrations of 25, 50, 100, 200, and 400 μg/mL (lanes 4, 5, 6, 7, and 8). * *p* < 0.05 when compared with the negative control group; ^†^ *p* < 0.05 when compared with the positive control group. Figure S7. Protective effect of Mountain pine EO against peroxyl- (A) and hydroxyl- (B) radicals-induced DNA damage. DNA from salmon sperm (lane 1, negative control), DNA damage control (lane 2, positive control), quercetin (lane 3, 100 μg/mL, standard), essential oils in concentrations of 25, 50, 100, 200, and 400 μg/mL (lanes 4, 5, 6, 7, and 8). * *p* < 0.05 when compared with the negative control group; ^†^ *p* < 0.05 when compared with the positive control group. Figure S8. Protective effect of Geranium Bourbon EO against peroxyl- (A) and hydroxyl- (B) radicals-induced DNA damage. DNA from salmon sperm (lane 1, negative control), DNA damage control (lane 2, positive control), quercetin (lane 3, 100 μg/mL, standard), essential oils in concentrations of 25, 50, 100, 200, and 400 μg/mL (lanes 4, 5, 6, 7, and 8). * *p* < 0.05 when compared with the negative control group; ^†^ *p* < 0.05 when compared with the positive control group. Figure S9. Protective effect of Oregano EO against peroxyl- (A) and hydroxyl- (B) radicals-induced DNA damage. DNA from salmon sperm (lane 1, negative control), DNA damage control (lane 2, positive control), quercetin (lane 3, 100 μg/mL, standard), essential oils in concentrations of 25, 50, 100, 200, and 400 μg/mL (lanes 4, 5, 6, 7, and 8). * *p* < 0.05 when compared with the negative control group; ^†^ *p* < 0.05 when compared with the positive control group. Figure S10. Protective effect of Coriander EO against peroxyl- (A) and hydroxyl- (B) radicals-induced DNA damage. DNA from salmon sperm (lane 1, negative control), DNA damage control (lane 2, positive control), quercetin (lane 3, 100 μg/mL, standard), essential oils in concentrations of 25, 50, 100, 200, and 400 μg/mL (lanes 4, 5, 6, 7, and 8). * *p* < 0.05 when compared with the negative control group; ^†^ *p* < 0.05 when compared with the positive control group. Figure S11. Protective effect of Lavender EO against peroxyl- (A) and hydroxyl- (B) radicals-induced DNA damage. DNA from salmon sperm (lane 1, negative control), DNA damage control (lane 2, positive control), quercetin (lane 3, 100 μg/mL, standard), essential oils in concentrations of 25, 50, 100, 200, and 400 μg/mL (lanes 4, 5, 6, 7, and 8). * *p* < 0.05 when compared with the negative control group; ^†^ *p* < 0.05 when compared with the positive control group. Figure S12. Protective effect of Myrtle EO against peroxyl- (A) and hydroxyl- (B) radicals-induced DNA damage. DNA from salmon sperm (lane 1, negative control), DNA damage control (lane 2, positive control), quercetin (lane 3, 100 μg/mL, standard), essential oils in concentrations of 25, 50, 100, 200, and 400 μg/mL (lanes 4, 5, 6, 7, and 8). * *p* < 0.05 when compared with the negative control group; ^†^ *p* < 0.05 when compared with the positive control group. Figure S13. Protective effect of Garlic EO against peroxyl- (A) and hydroxyl- (B) radicals-induced DNA damage. DNA from salmon sperm (lane 1, negative control), DNA damage control (lane 2, positive control), quercetin (lane 3, 100 μg/mL, standard), essential oils in concentrations of 25, 50, 100, 200, and 400 μg/mL (lanes 4, 5, 6, 7, and 8). * *p* < 0.05 when compared with the negative control group; ^†^ *p* < 0.05 when compared with the positive control group. Figure S14. Protective effect of Cardamom EO against peroxyl- (A) and hydroxyl- (B) radicals-induced DNA damage. DNA from salmon sperm (lane 1, negative control), DNA damage control (lane 2, positive control), quercetin (lane 3, 100 μg/mL, standard), essential oils in concentrations of 25, 50, 100, 200, and 400 μg/mL (lanes 4, 5, 6, 7, and 8). * *p* < 0.05 when compared with the negative control group; ^†^ *p* < 0.05 when compared with the positive control group. Figure S15. Protective effect of Mandarin EO against peroxyl- (A) and hydroxyl- (B) radicals-induced DNA damage. DNA from salmon sperm (lane 1, negative control), DNA damage control (lane 2, positive control), quercetin (lane 3, 100 μg/mL, standard), essential oils in concentrations of 25, 50, 100, 200, and 400 μg/mL (lanes 4, 5, 6, 7, and 8). * *p* < 0.05 when compared with the negative control group; ^†^ *p* < 0.05 when compared with the positive control group. Figure S16. Protective effect of Hyssop EO against peroxyl- (A) and hydroxyl- (B) radicals-induced DNA damage. DNA from salmon sperm (lane 1, negative control), DNA damage control (lane 2, positive control), quercetin (lane 3, 100 μg/mL, standard), essential oils in concentrations of 25, 50, 100, 200, and 400 μg/mL (lanes 4, 5, 6, 7, and 8). * *p* < 0.05 when compared with the negative control group; ^†^ *p* < 0.05 when compared with the positive control group. Figure S17. Protective effect of Grapefruit EO against peroxyl- (A) and hydroxyl- (B) radicals-induced DNA damage. DNA from salmon sperm (lane 1, negative control), DNA damage control (lane 2, positive control), quercetin (lane 3, 100 μg/mL, standard), essential oils in concentrations of 25, 50, 100, 200, and 400 μg/mL (lanes 4, 5, 6, 7, and 8). * *p* < 0.05 when compared with the negative control group; ^†^ *p* < 0.05 when compared with the positive control group. Figure S18. Protective effect of Lemongrass EO against peroxyl- (A) and hydroxyl- (B) radicals-induced DNA damage. DNA from salmon sperm (lane 1, negative control), DNA damage control (lane 2, positive control), quercetin (lane 3, 100 μg/mL, standard), essential oils in concentrations of 25, 50, 100, 200, and 400 μg/mL (lanes 4, 5, 6, 7, and 8). * *p* < 0.05 when compared with the negative control group; ^†^ *p* < 0.05 when compared with the positive control group. Figure S19. Protective effect of Siberian pine EO against peroxyl- (A) and hydroxyl- (B) radicals-induced DNA damage. DNA from salmon sperm (lane 1, negative control), DNA damage control (lane 2, positive control), quercetin (lane 3, 100 μg/mL, standard), essential oils in concentrations of 25, 50, 100, 200, and 400 μg/mL (lanes 4, 5, 6, 7, and 8). * *p* < 0.05 when compared with the negative control group; ^†^ *p* < 0.05 when compared with the positive control group. Figure S20. Protective effect of Camphor EO against peroxyl- (A) and hydroxyl- (B) radicals-induced DNA damage. DNA from salmon sperm (lane 1, negative control), DNA damage control (lane 2, positive control), quercetin (lane 3, 100 μg/mL, standard), essential oils in concentrations of 25, 50, 100, 200, and 400 μg/mL (lanes 4, 5, 6, 7, and 8). * *p* < 0.05 when compared with the negative control group; ^†^ *p* < 0.05 when compared with the positive control group. Figure S21. Protective effect of Cade EO against peroxyl- (A) and hydroxyl- (B) radicals-induced DNA damage. DNA from salmon sperm (lane 1, negative control), DNA damage control (lane 2, positive control), quercetin (lane 3, 100 μg/mL, standard), essential oils in concentrations of 25, 50, 100, 200, and 400 μg/mL (lanes 4, 5, 6, 7, and 8). * *p* < 0.05 when compared with the negative control group; ^†^ *p* < 0.05 when compared with the positive control group. Figure S22. Protective effect of Cedar leaves EO against peroxyl- (A) and hydroxyl- (B) radicals-induced DNA damage. DNA from salmon sperm (lane 1, negative control), DNA damage control (lane 2, positive control), quercetin (lane 3, 100 μg/mL, standard), essential oils in concentrations of 25, 50, 100, 200, and 400 μg/mL (lanes 4, 5, 6, 7, and 8). * *p* < 0.05 when compared with the negative control group; ^†^ *p* < 0.05 when compared with the positive control group. Figure S23. Protective effect of Ginger EO against peroxyl- (A) and hydroxyl- (B) radicals-induced DNA damage. DNA from salmon sperm (lane 1, negative control), DNA damage control (lane 2, positive control), quercetin (lane 3, 100 μg/mL, standard), essential oils in concentrations of 25, 50, 100, 200, and 400 μg/mL (lanes 4, 5, 6, 7, and 8). * *p* < 0.05 when compared with the negative control group; ^†^ *p* < 0.05 when compared with the positive control group. Figure S24. Protective effect of Cumin EO against peroxyl- (A) and hydroxyl- (B) radicals-induced DNA damage. DNA from salmon sperm (lane 1, negative control), DNA damage control (lane 2, positive control), quercetin (lane 3, 100 μg/mL, standard), essential oils in concentrations of 25, 50, 100, 200, and 400 μg/mL (lanes 4, 5, 6, 7, and 8). * *p* < 0.05 when compared with the negative control group; ^†^ *p* < 0.05 when compared with the positive control group. Figure S25. Protective effect of Patchouli EO against peroxyl- (A) and hydroxyl- (B) radicals-induced DNA damage. DNA from salmon sperm (lane 1, negative control), DNA damage control (lane 2, positive control), quercetin (lane 3, 100 μg/mL, standard), essential oils in concentrations of 25, 50, 100, 200, and 400 μg/mL (lanes 4, 5, 6, 7, and 8). * *p* < 0.05 when compared with the negative control group; ^†^ *p* < 0.05 when compared with the positive control group. Figure S26. Protective effect of Orange bitter EO against peroxyl- (A) and hydroxyl- (B) radicals-induced DNA damage. DNA from salmon sperm (lane 1, negative control), DNA damage control (lane 2, positive control), quercetin (lane 3, 100 μg/mL, standard), essential oils in concentrations of 25, 50, 100, 200, and 400 μg/mL (lanes 4, 5, 6, 7, and 8). * *p* < 0.05 when compared with the negative control group; ^†^ *p* < 0.05 when compared with the positive control group. Figure S27. Protective effect of Eucalyptus EO against peroxyl- (A) and hydroxyl- (B) radicals-induced DNA damage. DNA from salmon sperm (lane 1, negative control), DNA damage control (lane 2, positive control), quercetin (lane 3, 100 μg/mL, standard), essential oils in concentrations of 25, 50, 100, 200, and 400 μg/mL (lanes 4, 5, 6, 7, and 8). * *p* < 0.05 when compared with the negative control group; ^†^ *p* < 0.05 when compared with the positive control group. Figure S28. Protective effect of Pine Silvestre natural EO against peroxyl- (A) and hydroxyl- (B) radicals-induced DNA damage. DNA from salmon sperm (lane 1, negative control), DNA damage control (lane 2, positive control), quercetin (lane 3, 100 μg/mL, standard), essential oils in concentrations of 25, 50, 100, 200, and 400 μg/mL (lanes 4, 5, 6, 7, and 8). * *p* < 0.05 when compared with the negative control group; ^†^ *p* < 0.05 when compared with the positive control group. Figure S29. Protective effect of Bergamot EO against peroxyl- (A) and hydroxyl- (B) radicals-induced DNA damage. DNA from salmon sperm (lane 1, negative control), DNA damage control (lane 2, positive control), quercetin (lane 3, 100 μg/mL, standard), essential oils in concentrations of 25, 50, 100, 200, and 400 μg/mL (lanes 4, 5, 6, 7, and 8). * *p* < 0.05 when compared with the negative control group; ^†^ *p* < 0.05 when compared with the positive control group. Figure S30. Protective effect of Juniper EO against peroxyl- (A) and hydroxyl- (B) radicals-induced DNA damage. DNA from salmon sperm (lane 1, negative control), DNA damage control (lane 2, positive control), quercetin (lane 3, 100 μg/mL, standard), essential oils in concentrations of 25, 50, 100, 200, and 400 μg/mL (lanes 4, 5, 6, 7, and 8). * *p* < 0.05 when compared with the negative control group; ^†^ *p* < 0.05 when compared with the positive control group. Figure S31. Protective effect of Birch EO against peroxyl- (A) and hydroxyl- (B) radicals-induced DNA damage. DNA from salmon sperm (lane 1, negative control), DNA damage control (lane 2, positive control), quercetin (lane 3, 100 μg/mL, standard), essential oils in concentrations of 25, 50, 100, 200, and 400 μg/mL (lanes 4, 5, 6, 7, and 8). * *p* < 0.05 when compared with the negative control group; ^†^ *p* < 0.05 when compared with the positive control group. Figure S32. Protective effect of Fennel EO against peroxyl- (A) and hydroxyl- (B) radicals-induced DNA damage. DNA from salmon sperm (lane 1, negative control), DNA damage control (lane 2, positive control), quercetin (lane 3, 100 μg/mL, standard), essential oils in concentrations of 25, 50, 100, 200, and 400 μg/mL (lanes 4, 5, 6, 7, and 8). * *p* < 0.05 when compared with the negative control group; ^†^ *p* < 0.05 when compared with the positive control group. Figure S33. Protective effect of Cedar fruit EO against peroxyl- (A) and hydroxyl- (B) radicals-induced DNA damage. DNA from salmon sperm (lane 1, negative control), DNA damage control (lane 2, positive control), quercetin (lane 3, 100 μg/mL, standard), essential oils in concentrations of 25, 50, 100, 200, and 400 μg/mL (lanes 4, 5, 6, 7, and 8). * *p* < 0.05 when compared with the negative control group; ^†^ *p* < 0.05 when compared with the positive control group. Figure S34. Protective effect of Lemon EO against peroxyl- (A) and hydroxyl- (B) radicals-induced DNA damage. DNA from salmon sperm (lane 1, negative control), DNA damage control (lane 2, positive control), quercetin (lane 3, 100 μg/mL, standard), essential oils in concentrations of 25, 50, 100, 200, and 400 μg/mL (lanes 4, 5, 6, 7, and 8). * *p* < 0.05 when compared with the negative control group; ^†^ *p* < 0.05 when compared with the positive control group. Figure S35. Protective effect of Roman chamomile EO against peroxyl- (A) and hydroxyl- (B) radicals-induced DNA damage. DNA from salmon sperm (lane 1, negative control), DNA damage control (lane 2, positive control), quercetin (lane 3, 100 μg/mL, standard), essential oils in concentrations of 25, 50, 100, 200, and 400 μg/mL (lanes 4, 5, 6, 7, and 8). * *p* < 0.05 when compared with the negative control group; ^†^ *p* < 0.05 when compared with the positive control group. Figure S36. Protective effect of Savory EO against peroxyl- (A) and hydroxyl- (B) radicals-induced DNA damage. DNA from salmon sperm (lane 1, negative control), DNA damage control (lane 2, positive control), quercetin (lane 3, 100 μg/mL, standard), essential oils in concentrations of 25, 50, 100, 200, and 400 μg/mL (lanes 4, 5, 6, 7, and 8). * *p* < 0.05 when compared with the negative control group; ^†^ *p* < 0.05 when compared with the positive control group. Figure S37. Protective effect of Rosemary EO against peroxyl- (A) and hydroxyl- (B) radicals-induced DNA damage. DNA from salmon sperm (lane 1, negative control), DNA damage control (lane 2, positive control), quercetin (lane 3, 100 μg/mL, standard), essential oils in concentrations of 25, 50, 100, 200, and 400 μg/mL (lanes 4, 5, 6, 7, and 8). * *p* < 0.05 when compared with the negative control group; ^†^ *p* < 0.05 when compared with the positive control group. Figure S38. Protective effect of *Eucalyptus globulus* EO against peroxyl- (A) and hydroxyl- (B) radicals-induced DNA damage. DNA from salmon sperm (lane 1, negative control), DNA damage control (lane 2, positive control), quercetin (lane 3, 100 μg/mL, standard), essential oils in concentrations of 25, 50, 100, 200, and 400 μg/mL (lanes 4, 5, 6, 7, and 8). * *p* < 0.05 when compared with the negative control group; ^†^ *p* < 0.05 when compared with the positive control group. Figure S39. Protective effect of Orange sweet EO against peroxyl- (A) and hydroxyl- (B) radicals-induced DNA damage. DNA from salmon sperm (lane 1, negative control), DNA damage control (lane 2, positive control), quercetin (lane 3, 100 μg/mL, standard), essential oils in concentrations of 25, 50, 100, 200, and 400 μg/mL (lanes 4, 5, 6, 7, and 8). * *p* < 0.05 when compared with the negative control group; ^†^ *p* < 0.05 when compared with the positive control group. Figure S40. Protective effect of Niaouly EO against peroxyl- (A) and hydroxyl- (B) radicals-induced DNA damage. DNA from salmon sperm (lane 1, negative control), DNA damage control (lane 2, positive control), quercetin (lane 3, 100 μg/mL, standard), essential oils in concentrations of 25, 50, 100, 200, and 400 μg/mL (lanes 4, 5, 6, 7, and 8). * *p* < 0.05 when compared with the negative control group; ^†^ *p* < 0.05 when compared with the positive control group. Figure S41. Protective effect of Artemisia EO against peroxyl- (A) and hydroxyl- (B) radicals-induced DNA damage. DNA from salmon sperm (lane 1, negative control), DNA damage control (lane 2, positive control), quercetin (lane 3, 100 μg/mL, standard), essential oils in concentrations of 25, 50, 100, 200, and 400 μg/mL (lanes 4, 5, 6, 7, and 8). * *p* < 0.05 when compared with the negative control group; ^†^ *p* < 0.05 when compared with the positive control group. Figure S42. Protective effect of Cajeput EO against peroxyl- (A) and hydroxyl- (B) radicals-induced DNA damage. DNA from salmon sperm (lane 1, negative control), DNA damage control (lane 2, positive control), quercetin (lane 3, 100 μg/mL, standard), essential oils in concentrations of 25, 50, 100, 200, and 400 μg/mL (lanes 4, 5, 6, 7, and 8). * *p* < 0.05 when compared with the negative control group; ^†^ *p* < 0.05 when compared with the positive control group. Figure S43. Protective effect of Black pepper EO against peroxyl- (A) and hydroxyl- (B) radicals-induced DNA damage. DNA from salmon sperm (lane 1, negative control), DNA damage control (lane 2, positive control), quercetin (lane 3, 100 μg/mL, standard), essential oils in concentrations of 25, 50, 100, 200, and 400 μg/mL (lanes 4, 5, 6, 7, and 8). * *p* < 0.05 when compared with the negative control group; ^†^ *p* < 0.05 when compared with the positive control group. Figure S44. Protective effect of White thyme EO against peroxyl- (A) and hydroxyl- (B) radicals-induced DNA damage. DNA from salmon sperm (lane 1, negative control), DNA damage control (lane 2, positive control), quercetin (lane 3, 100 μg/mL, standard), essential oils in concentrations of 25, 50, 100, 200, and 400 μg/mL (lanes 4, 5, 6, 7, and 8). * *p* < 0.05 when compared with the negative control group; ^†^ *p* < 0.05 when compared with the positive control group. Figure S45. Protective effect of Marjoram EO against peroxyl- (A) and hydroxyl- (B) radicals-induced DNA damage. DNA from salmon sperm (lane 1, negative control), DNA damage control (lane 2, positive control), quercetin (lane 3, 100 μg/mL, standard), essential oils in concentrations of 25, 50, 100, 200, and 400 μg/mL (lanes 4, 5, 6, 7, and 8). * *p* < 0.05 when compared with the negative control group; ^†^ *p* < 0.05 when compared with the positive control group. Figure S46. Protective effect of Clove EO against peroxyl- (A) and hydroxyl- (B) radicals-induced DNA damage. DNA from salmon sperm (lane 1, negative control), DNA damage control (lane 2, positive control), quercetin (lane 3, 100 μg/mL, standard), essential oils in concentrations of 25, 50, 100, 200, and 400 μg/mL (lanes 4, 5, 6, 7, and 8). * *p* < 0.05 when compared with the negative control group; ^†^ *p* < 0.05 when compared with the positive control group. Figure S47. Protective effect of Cypress EO against peroxyl- (A) and hydroxyl- (B) radicals-induced DNA damage. DNA from salmon sperm (lane 1, negative control), DNA damage control (lane 2, positive control), quercetin (lane 3, 100 μg/mL, standard), essential oils in concentrations of 25, 50, 100, 200, and 400 μg/mL (lanes 4, 5, 6, 7, and 8). * *p* < 0.05 when compared with the negative control group; ^†^ *p* < 0.05 when compared with the positive control group. Figure S48. Protective effect of Nutmeg natural EO against peroxyl- (A) and hydroxyl- (B) radicals-induced DNA damage. DNA from salmon sperm (lane 1, negative control), DNA damage control (lane 2, positive control), quercetin (lane 3, 100 μg/mL, standard), essential oils in concentrations of 25, 50, 100, 200, and 400 μg/mL (lanes 4, 5, 6, 7, and 8). * *p* < 0.05 when compared with the negative control group; ^†^ *p* < 0.05 when compared with the positive control group. Figure S49. Protective effect of Peppermint EO against peroxyl- (A) and hydroxyl- (B) radicals-induced DNA damage. DNA from salmon sperm (lane 1, negative control), DNA damage control (lane 2, positive control), quercetin (lane 3, 100 μg/mL, standard), essential oils in concentrations of 25, 50, 100, 200, and 400 μg/mL (lanes 4, 5, 6, 7, and 8). * *p* < 0.05 when compared with the negative control group; ^†^ *p* < 0.05 when compared with the positive control group. Figure S50. Protective effect of Verbena EO against peroxyl- (A) and hydroxyl- (B) radicals-induced DNA damage. DNA from salmon sperm (lane 1, negative control), DNA damage control (lane 2, positive control), quercetin (lane 3, 100 μg/mL, standard), essential oils in concentrations of 25, 50, 100, 200, and 400 μg/mL (lanes 4, 5, 6, 7, and 8). * *p* < 0.05 when compared with the negative control group; ^†^ *p* < 0.05 when compared with the positive control group. Figure S51. Protective effect of Basil EO against peroxyl- (A) and hydroxyl- (B) radicals-induced DNA damage. DNA from salmon sperm (lane 1, negative control), DNA damage control (lane 2, positive control), quercetin (lane 3, 100 μg/mL, standard), essential oils in concentrations of 25, 50, 100, 200, and 400 μg/mL (lanes 4, 5, 6, 7, and 8). * *p* < 0.05 when compared with the negative control group; ^†^ *p* < 0.05 when compared with the positive control group. Figure S52. Protective effect of Palmarosa EO against peroxyl- (A) and hydroxyl- (B) radicals-induced DNA damage. DNA from salmon sperm (lane 1, negative control), DNA damage control (lane 2, positive control), quercetin (lane 3, 100 μg/mL, standard), essential oils in concentrations of 25, 50, 100, 200, and 400 μg/mL (lanes 4, 5, 6, 7, and 8). * *p* < 0.05 when compared with the negative control group; ^†^ *p* < 0.05 when compared with the positive control group. Figure S53. Protective effect of Laurel EO against peroxyl- (A) and hydroxyl- (B) radicals-induced DNA damage. DNA from salmon sperm (lane 1, negative control), DNA damage control (lane 2, positive control), quercetin (lane 3, 100 μg/mL, standard), essential oils in concentrations of 25, 50, 100, 200, and 400 μg/mL (lanes 4, 5, 6, 7, and 8). * *p* < 0.05 when compared with the negative control group; ^†^ *p* < 0.05 when compared with the positive control group. Figure S54. Protective effect of Natural anise pure EO against peroxyl- (A) and hydroxyl- (B) radicals-induced DNA damage. DNA from salmon sperm (lane 1, negative control), DNA damage control (lane 2, positive control), quercetin (lane 3, 100 μg/mL, standard), essential oils in concentrations of 25, 50, 100, 200, and 400 μg/mL (lanes 4, 5, 6, 7, and 8). * *p* < 0.05 when compared with the negative control group; ^†^ *p* < 0.05 when compared with the positive control group. Figure S55. Protective effect of Incense EO against peroxyl- (A) and hydroxyl- (B) radicals-induced DNA damage. DNA from salmon sperm (lane 1, negative control), DNA damage control (lane 2, positive control), quercetin (lane 3, 100 μg/mL, standard), essential oils in concentrations of 25, 50, 100, 200, and 400 μg/mL (lanes 4, 5, 6, 7, and 8). * *p* < 0.05 when compared with the negative control group; ^†^ *p* < 0.05 when compared with the positive control group. Figure S56. Protective effect of *Mentha suaveolens* EO against peroxyl- (A) and hydroxyl- (B) radicals-induced DNA damage. DNA from salmon sperm (lane 1, negative control), DNA damage control (lane 2, positive control), quercetin (lane 3, 100 μg/mL, standard), essential oils in concentrations of 25, 50, 100, 200, and 400 μg/mL (lanes 4, 5, 6, 7, and 8). * *p* < 0.05 when compared with the negative control group; ^†^ *p* < 0.05 when compared with the positive control group. Figure S57. Protective effect of *Coridotthymus capitatus* EO against peroxyl- (A) and hydroxyl- (B) radicals-induced DNA damage. DNA from salmon sperm (lane 1, negative control), DNA damage control (lane 2, positive control), quercetin (lane 3, 100 μg/mL, standard), essential oils in concentrations of 25, 50, 100, 200, and 400 μg/mL (lanes 4, 5, 6, 7, and 8). * *p* < 0.05 when compared with the negative control group; ^†^ *p* < 0.05 when compared with the positive control group. Figure S58. Protective effect of *Thymus vulgaris* EO against peroxyl- (A) and hydroxyl- (B) radicals-induced DNA damage. DNA from salmon sperm (lane 1, negative control), DNA damage control (lane 2, positive control), quercetin (lane 3, 100 μg/mL, standard), essential oils in concentrations of 25, 50, 100, 200, and 400 μg/mL (lanes 4, 5, 6, 7, and 8). * *p* < 0.05 when compared with the negative control group; ^†^ *p* < 0.05 when compared with the positive control group. Figure S59. Protective effect of *Origanum hirtum* EO against peroxyl- (A) and hydroxyl- (B) radicals-induced DNA damage. DNA from salmon sperm (lane 1, negative control), DNA damage control (lane 2, positive control), quercetin (lane 3, 100 μg/mL, standard), essential oils in concentrations of 25, 50, 100, 200, and 400 μg/mL (lanes 4, 5, 6, 7, and 8). * *p* < 0.05 when compared with the negative control group; ^†^ *p* < 0.05 when compared with the positive control group. Figure S60. Partial dependence graphs of limonene in the model of M^n+^ (A), DPPH^•^ (B), LOO^•^ (C), ABTS^•+^ (D), OH^•^ (E), ROO-RBD_50_ (F), HO-RBD_50_ (G). Figure S61. Partial dependence graphs of thymol in the model of M^n+^ (A), DPPH^•^ (B), LOO^•^ (C), ABTS^•+^ (D), OH^•^ (E), ROO-RBD_50_ (F), HO-RBD_50_ (G). Figure S62. Partial dependence graphs of eugenol in the model of M^n+^ (A), DPPH^•^ (B), LOO^•^ (C), ABTS^•+^ (D), OH^•^ (E), ROO-RBD_50_ (F), HO-RBD_50_ (G). Figure S63. Partial dependence graphs of chrysanthone in the model of M^n+^ (A), DPPH^•^ (B), LOO^•^ (C), ABTS^•+^ (D), OH^•^ (E), ROO-RBD_50_ (F), HO-RBD_50_ (G). Figure S64. Partial dependence graphs of chrysanthone in the model of M^n+^ (A), DPPH^•^ (B), LOO^•^ (C), ABTS^•+^ (D), OH^•^ (E), ROO-RBD_50_ (F), HO-RBD_50_ (G). Figure S65. Partial dependence graphs of α-pinene in the model of M^n+^ (A), DPPH^•^ (B), LOO^•^ (C), ABTS^•+^ (D), OH^•^ (E), ROO-RBD_50_ (F), HO-RBD_50_ (G). Figure S66. Partial dependence graphs of caryophillene in the model of M^n+^ (A), DPPH^•^ (B), LOO^•^ (C), ABTS^•+^ (D), OH^•^ (E), ROO-RBD_50_ (F), HO-RBD_50_ (G). Figure S67. Partial dependence graphs of *p*-cymene in the model of M^n+^ (A), DPPH^•^ (B), LOO^•^ (C), ABTS^•+^ (D), OH^•^ (E), ROO-RBD_50_ (F), HO-RBD_50_ (G). Results. ABTS cation-radical-neutralizing activity of EOs. Antigenotoxic activity in vitro. EOs with increasing dose-dependent potency to protect DNA from ROO^•^ and OH^•^. EOs with decreasing dose-dependent potency to protect from ROO^•^ and OH^•^. EOs with increasing and decreasing dose-dependent potency to protect DNA from ROO^•^ and OH^•^, respectively. EOs with decreasing and increasing dose-dependent potency to protect DNA from ROO^•^ and OH^•^, respectively. Liver redox status. 3.4.1.1. The rTBARS concentrations. 3.4.1.2. The rSOD catalytic activities. 3.4.1.3. The rCAT catalytic activities. The rGSH concentrations. The hepatocytes toxicity status. The rAST and rALT catalytic activities. The rALP and γ-GT catalytic activities. Kidneys’ redox status. The rTBARS concentrations. The rSOD catalytic activities. The rCAT catalytic activities. The rGSH concentration. Chronic kidney disease markers. The rXO catalytic activities. The rXO catalytic activities. The rNO concentrations. The GPx catalytic activities. EOs antigenotoxic activity in vivo. Antigenotoxicity in liver. Antigenotoxicity in kidneys.
