# Supplementary material for: In Vitro Antioxidant and In Vivo Antigenotoxic Features of a Series of 61 Essential Oils and Quantitative Composition–Activity Relationships Modeled through Machine Learning Algorithms
Source: Antioxidants (Basel). 2023 Sep 29;12(10):1815. doi: 10.3390/antiox12101815 (PMC10604248; doi:10.3390/antiox12101815)
Supplement: Supplementary file 1 [file antioxidants-12-01815-s001.zip › antioxidants-2601580-supplementary.pdf]

In vitro Antioxidant and *in vivo* Antigenotoxic Features of a series of 61 Essential Oils and Quantitative-Composition Activity Relationships Modeling Through Machine Learning Algorithms

Milan Mladenović[1*], Roberta Astolfi[2], Nevena Tomašević[1], Sanja Matić[3], Mijat Božović[4], Filippo Sapienza[2], and Rino Ragno[2*]

[1] Kragujevac Center for Computational Biochemistry, Department of Chemistry, Faculty of Science, University of Kragujevac, Radoja Domanovića 12, 34000 Kragujevac, P.O. Box 60, Serbia milan.mladenovic@pmf.kg.ac.rs (M.M.) nevena.tomasevic@pmf.kg.ac.rs (N.T.)

[2] Rome Center for Molecular Design, Department of Drug Chemistry and Technology, Faculty of Pharmacy and Medicine, Rome Sapienza University, P.le A. Moro 5, 00185, Rome, Italy filippo.sapienza@uniroma1.it (F.S.) roberta.astolfi@uniroma1.it (R.A.) rino.ragno@uniroma1.it (R.R.)

[3] Department of Science, Institute for Information Technologies Kragujevac, University of Kragujevac, Jovana Cvijića bb, 34000 Kragujevac, Serbia sanjamatic@kg.ac.rs (S.M.)

[4] University of Montenegro, Faculty of Science and Mathematics, Džordža Vašingtona bb, 81000 Podgorica, Montenegro mijatboz@ucg.ac.me (M.B)

* Correspondence: milan.mladenovic@pmf.kg.ac.rs Tel.: +381 638932160, Fax: +381 34335040 (M.M.) rino.ragno@uniroma1.it Tel.: +39 49913937, Fax: +39 49913627 (R.R.)

Content

Table S1. Investigated essential oils and their previously literature-reported chemical compositions and antioxidant features.
Table S2. Herein investigated essential oils chemical compositions.
Table S3. Total antioxidant capacity of examined EOs.
Table S4. DNA protective potential of selected 61 commercial essential oils on peroxyl radical-induced DNA damage.
Table S5. DNA protective potential of selected 61 commercial essential oils on hydroxyl radical–induced DNA damage.
Table S6. List of dataset pretreatment paramenters settings randomly varied during ML hyperparameters optimization.
Table S7. List of the initial thresholds[a] used for the ML models' for each antioxidant evaluation.
Table S8. List of hyperparameters setting used for the ML models' through random search optimization performed at 100 and 1000 iterations.
Table S9. List of hyperparameters setting used for the ML models' through random search optimization performed at 10000 and 100000 iterations.
Final ML Models Development
1. M^{n+}
1.1. 100 random iterations.
Table S10. Coarse best models obtained for each classifier.
Table S11. Hyperparameters associated to models **MN1-MN5**. The list is reported as python dictionaries.
1.2. 1000 random iterations.
Table S12. Coarse best models obtained for each classifier.
Table S13. Hyperparameters associated to models **MN6-MN9**. The list is reported as python dictionaries.
1.3. 10000 random iterations.
Table S14. Refined best models obtained for each classifier.
Table S15. Hyperparameters associated to models **MN10-MN13**. The list is reported as python dictionaries.
1.4. 100000 random iterations.
Table S16. Refined best models obtained for each classifier.

Table S17. Hyperparameters associated to models **MN14-MN16**. The list is reported as python dictionaries.

2. $DPPH^{\bullet}$

2.1. 100 random iterations.

Table S18. Coarse best models obtained for each classifier.

Table S19. Hyperparameters associated to models **DPPH1-DPPH5**. The list is reported as python dictionaries.

2.2. 1000 random iterations.

Table S20. Coarse best models obtained for each classifier.

Table S21. Hyperparameters associated to models **DPPH6-DPPH10**. The list is reported as python dictionaries.

2.3. 10000 random iterations.

Table S22. Refined best models obtained for each classifier.

Table S23. Hyperparameters associated to models **DPPH11-DPPH13**. The list is reported as python dictionaries.

2.4. 100000 random iterations.

Table S24. Refined best models obtained for each classifier.

Table S25. Hyperparameters associated to models **DPPH14-DPPH16**. The list is reported as python dictionaries.

3. $LOO^{\bullet}$

3.1. 100 random iterations.

Table S26. Coarse best models obtained for each classifier.

Table S27. Hyperparameters associated to models **LOO1-LOO5**. The list is reported as python dictionaries.

3.2. 1000 random iterations.

Table S28. Coarse best models obtained for each classifier.

Table S29. Hyperparameters associated to models **LOO6-LOO10**. The list is reported as python dictionaries.

3.3. 10000 random iterations.

Table S30. Refined best models obtained for each classifier.

Table S31. Hyperparameters associated to models **LOO10-LOO14**. The list is reported as python dictionaries.

3.4. 100000 random iterations.

Table S32. Refined best models obtained for each classifier.

Table S33. Hyperparameters associated to models **LOO15-LOO17**. The list is reported as python dictionaries.

4. $ABTS^{+\bullet}$

4.1. 100 random iterations.

Table S34. Coarse best models obtained for each classifier.

Table S35. Hyperparameters associated to models **ABTS1-ABTS5** The list is reported as python dictionaries.

4.2. 1000 random iterations.

Table S36. Coarse best models obtained for each classifier.

Table S37. Hyperparameters associated to models **ABTS6-ABTS10**. The list is reported as python dictionaries.

4.3. 10000 random iterations.

Table S38. Refined best models obtained for each classifier.

Table S39. Hyperparameters associated to models **ABTS11-ABTS14**. The list is reported as python dictionaries.

4.4. 100000 random iterations.

Table S40. Refined best models obtained for each classifier.

Table S41. Hyperparameters associated to models **ABTS15-ABTS17**. The list is reported as python dictionaries.

5. $OH^{\bullet}$

5.1. 100 random iterations.

Table S42. Coarse best models obtained for each classifier.

Table S43. Hyperparameters associated to models **OH1-OH5**. The list is reported as python dictionaries.

5.2. 1000 random iterations.

Table S44. Coarse best models obtained for each classifier.

Table S45. Hyperparameters associated to models **OH6-OH10**. The list is reported as python dictionaries.

5.3. 10000 random iterations.

Table S46. Refined best models obtained for each classifier.

Table S47. Hyperparameters associated to models **OH11-OH14**. The list is reported as python dictionaries.

5.4. 100000 random iterations.

Table S48. Refined best models obtained for each classifier.

Table S49. Hyperparameters associated to models **OH15-OH16**. The list is reported as python dictionaries.

6. ROO-RBD$_{50}$s

6.1. 100 random iterations.

Table S50. Coarse best models obtained for each classifier.

Table S51. Hyperparameters associated to models **ROO1-ROO5**. The list is reported as python dictionaries.

6.2. 1000 random iterations.

Table S52. Coarse best models obtained for each classifier.

Table S53. Hyperparameters associated to models **ROO6-ROO9**. The list is reported as python dictionaries.

6.3. 10000 random iterations.

Table S54. Refined best models obtained for each classifier.

Table S55. Hyperparameters associated to models **ROO10-ROO13**. The list is reported as python dictionaries.

6.4. 100000 random iterations.

Table S56. Refined best models obtained for each classifier.

Table S57. Hyperparameters associated to models **ROO14-ROO16**. The list is reported as python dictionaries.

7. OH-RBD$_{50}$

7.1. 100 random iterations.

Table S58. Coarse best models obtained for each classifier.

Table S59. Hyperparameters associated to models **OH-RBD1-OH-RBD5**. The list is reported as python dictionaries.

7.2. 1000 random iterations.

Table S60. Coarse best models obtained for each classifier.

Table S61. Hyperparameters associated to models **OH-RBD6-OH-RBD10**. The list is reported as python dictionaries.

7.3. 10000 random iterations.

Table S62. Refined best models obtained for each classifier.

Table S63. Hyperparameters associated to models **OH-RBD11-OH-RBD13**. The list is reported as python dictionaries.

7.4. 100000 random iterations.

Table S64. Refined best models obtained for each classifier.

Table S65. Hyperparameters associated to models **OH-RBD14-OH-RBD16**. The list is reported as python dictionaries.

Figure S1. Protective effect of Chamomile morocco EO against peroxyl (A) and hydroxyl (B) radicals-induced DNA damage. DNA from salmon sperm (lane 1, negative control), DNA damage control (lane 2, positive control), quercetin (lane 3, 100 μg/mL, standard), essential oils in concentrations of 25, 50, 100, 200, and 400 μg/mL (lanes 4, 5, 6, 7, and 8). $^*p < 0.05$ when compared with the negative control group $^+p < 0.05$ when compared with the positive control group.

Figure S2. Protective effect of Clary sage EO against peroxyl (A) and hydroxyl (B) radicals-induced DNA damage. DNA from salmon sperm (lane 1, negative control), DNA damage control (lane 2, positive control), quercetin (lane 3, 100 μg/mL, standard), essential oils in concentrations of 25, 50, 100, 200, and 400 μg/mL (lanes 4, 5, 6, 7, and 8). $^*p < 0.05$ when compared with the negative control group $^+p < 0.05$ when compared with the positive control group.

Figure S3. Protective effect of Sage EO against peroxyl (A) and hydroxyl (B) radicals-induced DNA damage. DNA from salmon sperm (lane 1, negative control), DNA damage control (lane 2, positive control), quercetin (lane 3, 100 μg/mL, standard), essential oils in concentrations of 25, 50, 100, 200, and 400 μg/mL (lanes 4, 5, 6, 7, and 8). $^*p < 0.05$ when compared with the negative control group $^+p < 0.05$ when compared with the positive control group.

Figure S4. Protective effect of Red thyme EO against peroxyl (A) and hydroxyl (B) radicals-induced DNA damage. DNA from salmon sperm (lane 1, negative control), DNA damage control (lane 2, positive control), quercetin (lane 3, 100 μg/mL, standard), essential oils in concentrations of 25, 50, 100, 200, and 400 μg/mL (lanes 4, 5, 6, 7, and 8). $^*p < 0.05$ when compared with the negative control group $^+p < 0.05$ when compared with the positive control group.

Figure S5. Protective effect Tea tree EO against peroxyl (A) and hydroxyl (B) radicals-induced DNA damage. DNA from salmon sperm (lane 1, negative control), DNA damage control (lane 2, positive control), quercetin (lane 3, 100 μg/mL, standard), essential oils in concentrations of 25, 50, 100, 200, and 400 μg/mL (lanes 4, 5, 6, 7, and 8). $^*p < 0.05$ when compared with the negative control group $^+p < 0.05$ when compared with the positive control group.

Figure S6. Protective effect of Melissa EO against peroxyl (A) and hydroxyl (B) radicals-induced DNA damage. DNA from salmon sperm (lane 1, negative control), DNA damage control (lane 2, positive control), quercetin (lane 3, 100 μg/mL, standard), essential oils in concentrations of 25, 50, 100, 200, and 400 μg/mL (lanes 4, 5, 6, 7, and 8). $^*p < 0.05$ when compared with the negative control group $^+p < 0.05$ when compared with the positive control group.

Figure S7. Protective effect of Mountain pine EO against peroxyl (A) and hydroxyl (B) radicals-induced DNA damage. DNA from salmon sperm (lane 1, negative control), DNA damage control (lane 2, positive control), quercetin (lane 3, 100

μg/mL, standard), essential oils in concentrations of 25, 50, 100, 200, and 400 μg/mL (lanes 4, 5, 6, 7, and 8). $^*p < 0.05$ when compared with the negative control group $^+p < 0.05$ when compared with the positive control group.

Figure S8. Protective effect of Geranium bourbon EO against peroxyl (A) and hydroxyl (B) radicals-induced DNA damage. DNA from salmon sperm (lane 1, negative control), DNA damage control (lane 2, positive control), quercetin (lane 3, 100 μg/mL, standard), essential oils in concentrations of 25, 50, 100, 200, and 400 μg/mL (lanes 4, 5, 6, 7, and 8). $^*p < 0.05$ when compared with the negative control group $^+p < 0.05$ when compared with the positive control group.

Figure S9. Protective effect of Oregano EO against peroxyl (A) and hydroxyl (B) radicals-induced DNA damage. DNA from salmon sperm (lane 1, negative control), DNA damage control (lane 2, positive control), quercetin (lane 3, 100 μg/mL, standard), essential oils in concentrations of 25, 50, 100, 200, and 400 μg/mL (lanes 4, 5, 6, 7, and 8). $^*p < 0.05$ when compared with the negative control group $^+p < 0.05$ when compared with the positive control group.

Figure S10. Protective effect of Coriander EO against peroxyl (A) and hydroxyl (B) radicals-induced DNA damage. DNA from salmon sperm (lane 1, negative control), DNA damage control (lane 2, positive control), quercetin (lane 3, 100 μg/mL, standard), essential oils in concentrations of 25, 50, 100, 200, and 400 μg/mL (lanes 4, 5, 6, 7, and 8). $^*p < 0.05$ when compared with the negative control group $^+p < 0.05$ when compared with the positive control group.

Figure S11. Protective effect of Lavander EO against peroxyl (A) and hydroxyl (B) radicals-induced DNA damage. DNA from salmon sperm (lane 1, negative control), DNA damage control (lane 2, positive control), quercetin (lane 3, 100 μg/mL, standard), essential oils in concentrations of 25, 50, 100, 200, and 400 μg/mL (lanes 4, 5, 6, 7, and 8). $^*p < 0.05$ when compared with the negative control group $^+p < 0.05$ when compared with the positive control group.

Figure S12. Protective effect of Myrtle EO against peroxyl (A) and hydroxyl (B) radicals-induced DNA damage. DNA from salmon sperm (lane 1, negative control), DNA damage control (lane 2, positive control), quercetin (lane 3, 100 μg/mL, standard), essential oils in concentrations of 25, 50, 100, 200, and 400 μg/mL (lanes 4, 5, 6, 7, and 8). $^*p < 0.05$ when compared with the negative control group $^+p < 0.05$ when compared with the positive control group.

Figure S13. Protective effect of Garlic EO against peroxyl (A) and hydroxyl (B) radicals-induced DNA damage. DNA from salmon sperm (lane 1, negative control), DNA damage control (lane 2, positive control), quercetin (lane 3, 100 μg/mL, standard), essential oils in concentrations of 25, 50, 100, 200, and 400 μg/mL (lanes 4, 5, 6, 7, and 8). $^*p < 0.05$ when compared with the negative control group $^+p < 0.05$ when compared with the positive control group.

Figure S14. Protective effect of Cardamom EO against peroxyl (A) and hydroxyl (B) radicals-induced DNA damage. DNA from salmon sperm (lane 1, negative control), DNA damage control (lane 2, positive control), quercetin (lane 3, 100 μg/mL, standard), essential oils in concentrations of 25, 50, 100, 200, and 400 μg/mL (lanes 4, 5, 6, 7, and 8). $^*p < 0.05$ when compared with the negative control group $^+p < 0.05$ when compared with the positive control group.

Figure S15. Protective effect of Mandarin EO against peroxyl (A) and hydroxyl (B) radicals-induced DNA damage. DNA from salmon sperm (lane 1, negative control), DNA damage control (lane 2, positive control), quercetin (lane 3, 100 μg/mL, standard), essential oils in concentrations of 25, 50, 100, 200, and 400 μg/mL (lanes 4, 5, 6, 7, and 8). $^*p < 0.05$ when compared with the negative control group $^+p < 0.05$ when compared with the positive control group.

Figure S16. Protective effect of Hyssop EO against peroxyl (A) and hydroxyl (B) radicals-induced DNA damage. DNA from salmon sperm (lane 1, negative control), DNA damage control (lane 2, positive control), quercetin (lane 3, 100 μg/mL, standard), essential oils in concentrations of 25, 50, 100, 200, and 400 μg/mL (lanes 4, 5, 6, 7, and 8). $^*p < 0.05$ when compared with the negative control group $^+p < 0.05$ when compared with the positive control group.

Figure S17. Protective effect of Grapefruit EO against peroxyl (A) and hydroxyl (B) radicals-induced DNA damage. DNA from salmon sperm (lane 1, negative control), DNA damage control (lane 2, positive control), quercetin (lane 3, 100 μg/mL, standard), essential oils in concentrations of 25, 50, 100, 200, and 400 μg/mL (lanes 4, 5, 6, 7, and 8). $^*p < 0.05$ when compared with the negative control group $^+p < 0.05$ when compared with the positive control group.

Figure S18. Protective effect of Lemongrass EO against peroxyl (A) and hydroxyl (B) radicals-induced DNA damage. DNA from salmon sperm (lane 1, negative control), DNA damage control (lane 2, positive control), quercetin (lane 3, 100 μg/mL, standard), essential oils in concentrations of 25, 50, 100, 200, and 400 μg/mL (lanes 4, 5, 6, 7, and 8). $^*p < 0.05$ when compared with the negative control group $^+p < 0.05$ when compared with the positive control group.

Figure S19. Protective effect of Siberian pine EO against peroxyl (A) and hydroxyl (B) radicals-induced DNA damage. DNA from salmon sperm (lane 1, negative control), DNA damage control (lane 2, positive control), quercetin (lane 3, 100 μg/mL, standard), essential oils in concentrations of 25, 50, 100, 200, and 400 μg/mL (lanes 4, 5, 6, 7, and 8). $^*p < 0.05$ when compared with the negative control group $^+p < 0.05$ when compared with the positive control group.

Figure S20. Protective effect of Camphor EO against peroxyl (A) and hydroxyl (B) radicals-induced DNA damage. DNA from salmon sperm (lane 1, negative control), DNA damage control (lane 2, positive control), quercetin (lane 3, 100 μg/mL,

standard), essential oils in concentrations of 25, 50, 100, 200, and 400 μg/mL (lanes 4, 5, 6, 7, and 8). *p < 0.05 when compared with the negative control group $^{+}$p < 0.05 when compared with the positive control group.

Figure S21. Protective effect of Cade EO against peroxyl (A) and hydroxyl (B) radicals-induced DNA damage. DNA from salmon sperm (lane 1, negative control), DNA damage control (lane 2, positive control), quercetin (lane 3, 100 μg/mL, standard), essential oils in concentrations of 25, 50, 100, 200, and 400 μg/mL (lanes 4, 5, 6, 7, and 8). *p < 0.05 when compared with the negative control group $^{+}$p < 0.05 when compared with the positive control group.

Figure S22. Protective effect of Cedar leaves EO against peroxyl (A) and hydroxyl (B) radicals-induced DNA damage. DNA from salmon sperm (lane 1, negative control), DNA damage control (lane 2, positive control), quercetin (lane 3, 100 μg/mL, standard), essential oils in concentrations of 25, 50, 100, 200, and 400 μg/mL (lanes 4, 5, 6, 7, and 8). *p < 0.05 when compared with the negative control group $^{+}$p < 0.05 when compared with the positive control group.

Figure S23. Protective effect of Ginger EO against peroxyl (A) and hydroxyl (B) radicals-induced DNA damage. DNA from salmon sperm (lane 1, negative control), DNA damage control (lane 2, positive control), quercetin (lane 3, 100 μg/mL, standard), essential oils in concentrations of 25, 50, 100, 200, and 400 μg/mL (lanes 4, 5, 6, 7, and 8). *p < 0.05 when compared with the negative control group $^{+}$p < 0.05 when compared with the positive control group.

Figure S24. Protective effect of Cumin EO against peroxyl (A) and hydroxyl (B) radicals-induced DNA damage. DNA from salmon sperm (lane 1, negative control), DNA damage control (lane 2, positive control), quercetin (lane 3, 100 μg/mL, standard), essential oils in concentrations of 25, 50, 100, 200, and 400 μg/mL (lanes 4, 5, 6, 7, and 8). *p < 0.05 when compared with the negative control group $^{+}$p < 0.05 when compared with the positive control group.

Figure S25. Protective effect of Patchouli EO against peroxyl (A) and hydroxyl (B) radicals-induced DNA damage. DNA from salmon sperm (lane 1, negative control), DNA damage control (lane 2, positive control), quercetin (lane 3, 100 μg/mL, standard), essential oils in concentrations of 25, 50, 100, 200, and 400 μg/mL (lanes 4, 5, 6, 7, and 8). *p < 0.05 when compared with the negative control group $^{+}$p < 0.05 when compared with the positive control group.

Figure S26. Protective effect of Orange bitter EO against peroxyl (A) and hydroxyl (B) radicals-induced DNA damage. DNA from salmon sperm (lane 1, negative control), DNA damage control (lane 2, positive control), quercetin (lane 3, 100 μg/mL, standard), essential oils in concentrations of 25, 50, 100, 200, and 400 μg/mL (lanes 4, 5, 6, 7, and 8). *p < 0.05 when compared with the negative control group $^{+}$p < 0.05 when compared with the positive control group.

Figure S27. Protective effect of Eucalyptus EO against peroxyl (A) and hydroxyl (B) radicals-induced DNA damage. DNA from salmon sperm (lane 1, negative control), DNA damage control (lane 2, positive control), quercetin (lane 3, 100 μg/mL, standard), essential oils in concentrations of 25, 50, 100, 200, and 400 μg/mL (lanes 4, 5, 6, 7, and 8). *p < 0.05 when compared with the negative control group $^{+}$p < 0.05 when compared with the positive control group.

Figure S28. Protective effect of Pine silvestre natural EO against peroxyl (A) and hydroxyl (B) radicals-induced DNA damage. DNA from salmon sperm (lane 1, negative control), DNA damage control (lane 2, positive control), quercetin (lane 3, 100 μg/mL, standard), essential oils in concentrations of 25, 50, 100, 200, and 400 μg/mL (lanes 4, 5, 6, 7, and 8). *p < 0.05 when compared with the negative control group $^{+}$p < 0.05 when compared with the positive control group.

Figure S29. Protective effect of Bergamot EO against peroxyl (A) and hydroxyl (B) radicals-induced DNA damage. DNA from salmon sperm (lane 1, negative control), DNA damage control (lane 2, positive control), quercetin (lane 3, 100 μg/mL, standard), essential oils in concentrations of 25, 50, 100, 200, and 400 μg/mL (lanes 4, 5, 6, 7, and 8). *p < 0.05 when compared with the negative control group $^{+}$p < 0.05 when compared with the positive control group.

Figure S30. Protective effect of Juniper EO against peroxyl (A) and hydroxyl (B) radicals-induced DNA damage. DNA from salmon sperm (lane 1, negative control), DNA damage control (lane 2, positive control), quercetin (lane 3, 100 μg/mL, standard), essential oils in concentrations of 25, 50, 100, 200, and 400 μg/mL (lanes 4, 5, 6, 7, and 8). *p < 0.05 when compared with the negative control group $^{+}$p < 0.05 when compared with the positive control group.

Figure S31. Protective effect of Birch EO against peroxyl (A) and hydroxyl (B) radicals-induced DNA damage. DNA from salmon sperm (lane 1, negative control), DNA damage control (lane 2, positive control), quercetin (lane 3, 100 μg/mL, standard), essential oils in concentrations of 25, 50, 100, 200, and 400 μg/mL (lanes 4, 5, 6, 7, and 8). *p < 0.05 when compared with the negative control group $^{+}$p < 0.05 when compared with the positive control group.

Figure S32. Protective effect of Fennel EO against peroxyl (A) and hydroxyl (B) radicals-induced DNA damage. DNA from salmon sperm (lane 1, negative control), DNA damage control (lane 2, positive control), quercetin (lane 3, 100 μg/mL, standard), essential oils in concentrations of 25, 50, 100, 200, and 400 μg/mL (lanes 4, 5, 6, 7, and 8). *p < 0.05 when compared with the negative control group $^{+}$p < 0.05 when compared with the positive control group.

Figure S33. Protective effect of Cedar fruit EO against peroxyl (A) and hydroxyl (B) radicals-induced DNA damage. DNA from salmon sperm (lane 1, negative control), DNA damage control (lane 2, positive control), quercetin (lane 3, 100 μg/mL,

standard), essential oils in concentrations of 25, 50, 100, 200, and 400 μg/mL (lanes 4, 5, 6, 7, and 8). *$p < 0.05$ when compared with the negative control group ⁺$p < 0.05$ when compared with the positive control group.

Figure S34. Protective effect of Lemon EO against peroxyl (A) and hydroxyl (B) radicals-induced DNA damage. DNA from salmon sperm (lane 1, negative control), DNA damage control (lane 2, positive control), quercetin (lane 3, 100 μg/mL, standard), essential oils in concentrations of 25, 50, 100, 200, and 400 μg/mL (lanes 4, 5, 6, 7, and 8). *$p < 0.05$ when compared with the negative control group ⁺$p < 0.05$ when compared with the positive control group.

Figure S35. Protective effect of Roman chamomile EO against peroxyl (A) and hydroxyl (B) radicals-induced DNA damage. DNA from salmon sperm (lane 1, negative control), DNA damage control (lane 2, positive control), quercetin (lane 3, 100 μg/mL, standard), essential oils in concentrations of 25, 50, 100, 200, and 400 μg/mL (lanes 4, 5, 6, 7, and 8). *$p < 0.05$ when compared with the negative control group ⁺$p < 0.05$ when compared with the positive control group.

Figure S36. Protective effect of Savory EO against peroxyl (A) and hydroxyl (B) radicals-induced DNA damage. DNA from salmon sperm (lane 1, negative control), DNA damage control (lane 2, positive control), quercetin (lane 3, 100 μg/mL, standard), essential oils in concentrations of 25, 50, 100, 200, and 400 μg/mL (lanes 4, 5, 6, 7, and 8). *$p < 0.05$ when compared with the negative control group ⁺$p < 0.05$ when compared with the positive control group.

Figure S37. Protective effect of Rosemary EO against peroxyl (A) and hydroxyl (B) radicals-induced DNA damage. DNA from salmon sperm (lane 1, negative control), DNA damage control (lane 2, positive control), quercetin (lane 3, 100 μg/mL, standard), essential oils in concentrations of 25, 50, 100, 200, and 400 μg/mL (lanes 4, 5, 6, 7, and 8). *$p < 0.05$ when compared with the negative control group ⁺$p < 0.05$ when compared with the positive control group.

Figure S38. Protective effect of *Eucalyptus globulus* EO against peroxyl (A) and hydroxyl (B) radicals-induced DNA damage. DNA from salmon sperm (lane 1, negative control), DNA damage control (lane 2, positive control), quercetin (lane 3, 100 μg/mL, standard), essential oils in concentrations of 25, 50, 100, 200, and 400 μg/mL (lanes 4, 5, 6, 7, and 8). *$p < 0.05$ when compared with the negative control group ⁺$p < 0.05$ when compared with the positive control group.

Figure S39. Protective effect of Orange sweet EO against peroxyl (A) and hydroxyl (B) radicals-induced DNA damage. DNA from salmon sperm (lane 1, negative control), DNA damage control (lane 2, positive control), quercetin (lane 3, 100 μg/mL, standard), essential oils in concentrations of 25, 50, 100, 200, and 400 μg/mL (lanes 4, 5, 6, 7, and 8). *$p < 0.05$ when compared with the negative control group ⁺$p < 0.05$ when compared with the positive control group.

Figure S40. Protective effect of Niaouly EO against peroxyl (A) and hydroxyl (B) radicals-induced DNA damage. DNA from salmon sperm (lane 1, negative control), DNA damage control (lane 2, positive control), quercetin (lane 3, 100 μg/mL, standard), essential oils in concentrations of 25, 50, 100, 200, and 400 μg/mL (lanes 4, 5, 6, 7, and 8). *$p < 0.05$ when compared with the negative control group ⁺$p < 0.05$ when compared with the positive control group.

Figure S41. Protective effect of Artemisia EO against peroxyl (A) and hydroxyl (B) radicals-induced DNA damage. DNA from salmon sperm (lane 1, negative control), DNA damage control (lane 2, positive control), quercetin (lane 3, 100 μg/mL, standard), essential oils in concentrations of 25, 50, 100, 200, and 400 μg/mL (lanes 4, 5, 6, 7, and 8). *$p < 0.05$ when compared with the negative control group ⁺$p < 0.05$ when compared with the positive control group.

Figure S42. Protective effect of Cajeput EO against peroxyl (A) and hydroxyl (B) radicals-induced DNA damage. DNA from salmon sperm (lane 1, negative control), DNA damage control (lane 2, positive control), quercetin (lane 3, 100 μg/mL, standard), essential oils in concentrations of 25, 50, 100, 200, and 400 μg/mL (lanes 4, 5, 6, 7, and 8). *$p < 0.05$ when compared with the negative control group ⁺$p < 0.05$ when compared with the positive control group.

Figure S43. Protective effect of Black pepper EO against peroxyl (A) and hydroxyl (B) radicals-induced DNA damage. DNA from salmon sperm (lane 1, negative control), DNA damage control (lane 2, positive control), quercetin (lane 3, 100 μg/mL, standard), essential oils in concentrations of 25, 50, 100, 200, and 400 μg/mL (lanes 4, 5, 6, 7, and 8). *$p < 0.05$ when compared with the negative control group ⁺$p < 0.05$ when compared with the positive control group.

Figure S44. Protective effect of White thyme EO against peroxyl (A) and hydroxyl (B) radicals-induced DNA damage. DNA from salmon sperm (lane 1, negative control), DNA damage control (lane 2, positive control), quercetin (lane 3, 100 μg/mL, standard), essential oils in concentrations of 25, 50, 100, 200, and 400 μg/mL (lanes 4, 5, 6, 7, and 8). *$p < 0.05$ when compared with the negative control group ⁺$p < 0.05$ when compared with the positive control group.

Figure S45. Protective effect of Marjoram EO against peroxyl (A) and hydroxyl (B) radicals-induced DNA damage. DNA from salmon sperm (lane 1, negative control), DNA damage control (lane 2, positive control), quercetin (lane 3, 100 μg/mL, standard), essential oils in concentrations of 25, 50, 100, 200, and 400 μg/mL (lanes 4, 5, 6, 7, and 8). *$p < 0.05$ when compared with the negative control group ⁺$p < 0.05$ when compared with the positive control group.

Figure S46. Protective effect of Clove EO against peroxyl (A) and hydroxyl (B) radicals-induced DNA damage. DNA from salmon sperm (lane 1, negative control), DNA damage control (lane 2, positive control), quercetin (lane 3, 100 μg/mL,

standard), essential oils in concentrations of 25, 50, 100, 200, and 400 μg/mL (lanes 4, 5, 6, 7, and 8). *p < 0.05 when compared with the negative control group ⁺p < 0.05 when compared with the positive control group.

Figure S47. Protective effect of Cypress EO against peroxyl (A) and hydroxyl (B) radicals-induced DNA damage. DNA from salmon sperm (lane 1, negative control), DNA damage control (lane 2, positive control), quercetin (lane 3, 100 μg/mL, standard), essential oils in concentrations of 25, 50, 100, 200, and 400 μg/mL (lanes 4, 5, 6, 7, and 8). *p < 0.05 when compared with the negative control group ⁺p < 0.05 when compared with the positive control group.

Figure S48. Protective effect of Nutmeg natural EO against peroxyl (A) and hydroxyl (B) radicals-induced DNA damage. DNA from salmon sperm (lane 1, negative control), DNA damage control (lane 2, positive control), quercetin (lane 3, 100 μg/mL, standard), essential oils in concentrations of 25, 50, 100, 200, and 400 μg/mL (lanes 4, 5, 6, 7, and 8). *p < 0.05 when compared with the negative control group ⁺p < 0.05 when compared with the positive control group.

Figure S49. Protective effect of Peppermint EO against peroxyl (A) and hydroxyl (B) radicals-induced DNA damage. DNA from salmon sperm (lane 1, negative control), DNA damage control (lane 2, positive control), quercetin (lane 3, 100 μg/mL, standard), essential oils in concentrations of 25, 50, 100, 200, and 400 μg/mL (lanes 4, 5, 6, 7, and 8). *p < 0.05 when compared with the negative control group ⁺p < 0.05 when compared with the positive control group.

Figure S50. Protective effect of Verbena EO against peroxyl (A) and hydroxyl (B) radicals-induced DNA damage. DNA from salmon sperm (lane 1, negative control), DNA damage control (lane 2, positive control), quercetin (lane 3, 100 μg/mL, standard), essential oils in concentrations of 25, 50, 100, 200, and 400 μg/mL (lanes 4, 5, 6, 7, and 8). *p < 0.05 when compared with the negative control group ⁺p < 0.05 when compared with the positive control group.

Figure S51. Protective effect of Basil EO against peroxyl (A) and hydroxyl (B) radicals-induced DNA damage. DNA from salmon sperm (lane 1, negative control), DNA damage control (lane 2, positive control), quercetin (lane 3, 100 μg/mL, standard), essential oils in concentrations of 25, 50, 100, 200, and 400 μg/mL (lanes 4, 5, 6, 7, and 8). *p < 0.05 when compared with the negative control group ⁺p < 0.05 when compared with the positive control group.

Figure S52. Protective effect of Palmarosa EO against peroxyl (A) and hydroxyl (B) radicals-induced DNA damage. DNA from salmon sperm (lane 1, negative control), DNA damage control (lane 2, positive control), quercetin (lane 3, 100 μg/mL, standard), essential oils in concentrations of 25, 50, 100, 200, and 400 μg/mL (lanes 4, 5, 6, 7, and 8). *p < 0.05 when compared with the negative control group ⁺p < 0.05 when compared with the positive control group.

Figure S53. Protective effect of Laurel EO against peroxyl (A) and hydroxyl (B) radicals-induced DNA damage. DNA from salmon sperm (lane 1, negative control), DNA damage control (lane 2, positive control), quercetin (lane 3, 100 μg/mL, standard), essential oils in concentrations of 25, 50, 100, 200, and 400 μg/mL (lanes 4, 5, 6, 7, and 8). *p < 0.05 when compared with the negative control group ⁺p < 0.05 when compared with the positive control group.

Figure S54. Protective effect of Narural anise pure EO against peroxyl (A) and hydroxyl (B) radicals-induced DNA damage. DNA from salmon sperm (lane 1, negative control), DNA damage control (lane 2, positive control), quercetin (lane 3, 100 μg/mL, standard), essential oils in concentrations of 25, 50, 100, 200, and 400 μg/mL (lanes 4, 5, 6, 7, and 8). *p < 0.05 when compared with the negative control group ⁺p < 0.05 when compared with the positive control group.

Figure S55. Protective effect of Incense EO against peroxyl (A) and hydroxyl (B) radicals-induced DNA damage. DNA from salmon sperm (lane 1, negative control), DNA damage control (lane 2, positive control), quercetin (lane 3, 100 μg/mL, standard), essential oils in concentrations of 25, 50, 100, 200, and 400 μg/mL (lanes 4, 5, 6, 7, and 8). *p < 0.05 when compared with the negative control group ⁺p < 0.05 when compared with the positive control group.

Figure S56. Protective effect of *Mentha suaveolens* EO against peroxyl (A) and hydroxyl (B) radicals-induced DNA damage. DNA from salmon sperm (lane 1, negative control), DNA damage control (lane 2, positive control), quercetin (lane 3, 100 μg/mL, standard), essential oils in concentrations of 25, 50, 100, 200, and 400 μg/mL (lanes 4, 5, 6, 7, and 8). *p < 0.05 when compared with the negative control group ⁺p < 0.05 when compared with the positive control group.

Figure S57. Protective effect of *Coridotthymus capitatus* EO against peroxyl (A) and hydroxyl (B) radicals-induced DNA damage. DNA from salmon sperm (lane 1, negative control), DNA damage control (lane 2, positive control), quercetin (lane 3, 100 μg/mL, standard), essential oils in concentrations of 25, 50, 100, 200, and 400 μg/mL (lanes 4, 5, 6, 7, and 8). *p < 0.05 when compared with the negative control group ⁺p < 0.05 when compared with the positive control group.

Figure S58. Protective effect of *Thymus vulgaris* EO against peroxyl (A) and hydroxyl (B) radicals-induced DNA damage. DNA from salmon sperm (lane 1, negative control), DNA damage control (lane 2, positive control), quercetin (lane 3, 100 μg/mL, standard), essential oils in concentrations of 25, 50, 100, 200, and 400 μg/mL (lanes 4, 5, 6, 7, and 8). *p < 0.05 when compared with the negative control group ⁺p < 0.05 when compared with the positive control group.

Figure S59. Protective effect of *Origanum hirtum* EO against peroxyl (A) and hydroxyl (B) radicals-induced DNA damage. DNA from salmon sperm (lane 1, negative control), DNA damage control (lane 2, positive control), quercetin (lane 3, 100

μg/mL, standard), essential oils in concentrations of 25, 50, 100, 200, and 400 μg/mL (lanes 4, 5, 6, 7, and 8). *p < 0.05 when compared with the negative control group ⁺p < 0.05 when compared with the positive control group.
Figure S60. Partial dependence graphs of limonene in the model of M^{n+} (A), DPPH$^{\bullet}$ (B), LOO$^{\bullet}$ (C), ABTS$^{\bullet+}$ (D), OH$^{\bullet}$ (E), ROO-RBD$_{50}$ (F), HO-RBD$_{50}$ (G).
Figure S61. Partial dependence graphs of thymol in the model of M^{n+} (A), DPPH$^{\bullet}$ (B), LOO$^{\bullet}$ (C), ABTS$^{\bullet+}$ (D), OH$^{\bullet}$ (E), ROO-RBD$_{50}$ (F), HO-RBD$_{50}$ (G).
Figure S62. Partial dependence graphs of eugenol in the model of M^{n+} (A), DPPH$^{\bullet}$ (B), LOO$^{\bullet}$ (C), ABTS$^{\bullet+}$ (D), OH$^{\bullet}$ (E), ROO-RBD$_{50}$ (F), HO-RBD$_{50}$ (G).
Figure S63. Partial dependence graphs of chrysanthone in the model of M^{n+} (A), DPPH$^{\bullet}$ (B), LOO$^{\bullet}$ (C), ABTS$^{\bullet+}$ (D), OH$^{\bullet}$ (E), ROO-RBD$_{50}$ (F), HO-RBD$_{50}$ (G).
Figure S64. Partial dependence graphs of chrysanthone in the model of M^{n+} (A), DPPH$^{\bullet}$ (B), LOO$^{\bullet}$ (C), ABTS$^{\bullet+}$ (D), OH$^{\bullet}$ (E), ROO-RBD$_{50}$ (F), HO-RBD$_{50}$ (G).
Figure S65. Partial dependence graphs of α-pinene in the model of M^{n+} (A), DPPH$^{\bullet}$ (B), LOO$^{\bullet}$ (C), ABTS$^{\bullet+}$ (D), OH$^{\bullet}$ (E), ROO-RBD$_{50}$ (F), HO-RBD$_{50}$ (G).
Figure S66. Partial dependence graphs of caryophillene in the model of M^{n+} (A), DPPH$^{\bullet}$ (B), LOO$^{\bullet}$ (C), ABTS$^{\bullet+}$ (D), OH$^{\bullet}$ (E), ROO-RBD$_{50}$ (F), HO-RBD$_{50}$ (G).
Figure S67. Partial dependence graphs of ***p*-cymene** in the model of M^{n+} (A), DPPH$^{\bullet}$ (B), LOO$^{\bullet}$ (C), ABTS$^{\bullet+}$ (D), OH$^{\bullet}$ (E), ROO-RBD$_{50}$ (F), HO-RBD$_{50}$ (G).

Results

ABTS cation radical neutralizing activity of EOs.
Antigenotoxic activity *in vitro*.
EOs with increasing dose-dependent potency to protect DNA from ROO$^{\bullet}$ and OH$^{\bullet}$.
EOs with decreasing dose-dependent potency to protect from ROO$^{\bullet}$ and OH$^{\bullet}$.
EOs with increasing and decreasing dose-dependent potency to protect DNA from ROO$^{\bullet}$ and OH$^{\bullet}$, respectively.
EOs with decreasing and increasing dose-dependent potency to protect DNA from ROO$^{\bullet}$ and OH$^{\bullet}$, respectively.
Liver redox status.
The *r*TBARS concentrations.
The *r*SOD catalytic activities.
The *r*CAT catalytic activities.
The *r*GSH concentrations.
The hepatocytes toxicity status.
The *r*AST and rALT catalytic activities.
The *r*ALP and γ-GT catalytic activities.
Kidneys redox status.
The *r*TBARS concentrations.
The *r*SOD catalytic activities.
The *r*CAT catalytic activities.
The *r*GSH concentration.
Chronic kidney disease markers.
The *r*XO catalytic activities.
The *r*XO catalytic activities.
The *r*NO concentrations.
The GPx catalytic activities.
EOs antigenotoxic activity *in vivo*.
Antigenotoxicity in liver.
Antigenotoxicity in kidneys.

Table S1. Investigated essential oils and their previously literature-reported chemical compositions and antioxidant features.

EOs IDs<br>*Official Latin names of biological source/sources*	Components (%)	TAC[a] µg AAE/mg	M^{n+}[b]	DPPH•[c]	LOO•[d]	ABTS•+[e]	OH•[f]	Ref.
				EC$_{50}$[i] (µg/mL)				
Chamomile Morocco *Cladanthus mixtus* (L.) Chevall.	tiglic aldehyde (36 %)<br>methacrylate (34 %)	NPR[g]	NPR	342 ± 0.2	NPR	NPR	NPR	[21]
Clary sage *Salvia sclarea* L.	linalyl acetate (62.6%)<br>linalool (11.1%)	NPR	NPR	7.79 ±1.06	NPR	2.26 ± 0.05	NPR	[22]
Sage oil *Salvia officinalis* L.	*cis*-thujone (43.2%)<br>camphor (17.6%)<br>1,8-cineole (13.8%)	NPR	NPR	6160-9650	NPR	43.64 ± 2.51	NPR	[23]
		NPR	126.85 - 212.91	113.56 - 88.43	NPR	141.55 - 244.65	NPR	[94]
	1,8-cineole (30.3%)<br>camphor (17.1%)	NPR	NPR	14.10 ± 0.17	NPR	NPR	NPR	[22]
Red thyme *Thymus praecox* Opiz ''coccineus ''	NPR	NPR	NPR	NPR	NPR	NPR	NPR	
Tea tree *Melaleuca alternifolia* (Maiden & Betche) Cheel	4-carvomenthenol (31.11%)<br>γ-terpinene (25.30%)<br>α-terpinene (12.70%)	NPR	NPR	48.35	135.9	NPR	43.71	[24]
	4-carvomenthenol (40.44%)<br>γ-terpinene (19.54%)<br>α-terpinene (7.69%)	NPR	NPR	12.5	NPR	NPR	NPR	[25]
Melissa *Melissa officinalis* L.	NPR	NPR	472.50 ± 0.20	7.58	NPR	73.65 ± 0.07	NPR	[26]
	NPR	NPR	NPR	62.38 ± 0.63	NPR	NPR		[27]

Mountain pine *Pinus mugo* Turra	3-carene (12.11-18.74%) α-pinene (7.21-12.92%	NPR	NPR	2510 - 4260	NPR	43.08 ±6.95	NPR	[28]
	β-pinene (43.3%) α-pinene (16.6%) β-phellandrene (16%) limonene (9.5%)	NPR	NPR	3.08 ± 0.65	NPR	NPR	NPR	[29]
	3-carene (12.11-18.74 %) α-pinene (7.21-12.92%) germacrene D (2.38-11.81 %)	NPR	NPR	2510 - 4260	2590 - 4140	NPR	NPR	[29]
Geranium Bourbon *Pelargonium x asperum* Ehrh. ex Willd.	citronellol (25.07%) citronellyl acetate (10.52%) geraniol (10.46%)	NPR	NPR	14620	NPR	NPR	NPR	[30]
	NPR	NPR	NPR	16990	NPR	NPR	NPR	[31]
Oregano *Origanum vulgare* L.	carvacrol (30.73%) thymol (18.81%) *p*-cymene (10.88%)	NPR	NPR	332 ± 0.040	NPR	NPR	NPR	[32]
	NPR	NPR	NPR	2.99	NPR	NPR	NPR	[33]
	thymol (45%) carvacrol (37.4%)	NPR	NPR	590	NPR	NPR	NPR	[34]
Ylang-ylang *Cananga odorata* (Lam.) Hook. f. & Thomson	α-farnesene (10.33%) linalool (9.97%) α-amorphene (9.4%) geraniol (7.54%)	NPR	NPR	1030 ± 20	NPR	NPR	NPR	[35]
Coriander *Coriandrum sativum* L.	linalool (60.06%)	NPR	159.70 ± 0.13	21050	NPR	1397 ± 3.13	NPR	[36]
	NPR	NPR	NPR	97.84	NPR	NPR	NPR	[37]
	NPR	NPR	NPR	147	NPR	NPR	NPR	[26]

	NPR	NPR	NPR	385.00 ± 1.21	NPR	NPR	NPR	[38]
Lavander *Lavandula angustifolia* Mill.	linalool (24.63%) camphor (13.58%) linalyl acetate (8.89%)	NPR	NPR	216	NPR	42.60±0.22	NPR	[39]
	linalool (37.65%) linalyl acetate (15.29%)	NPR	NPR	60.53±0.21	NPR	NPR	NPR	[40]
	1,8-cineole (21.5%) linalool (44.94%)	NPR	NPR	133	NPR	NPR	NPR	[41]
Myrtle *Myrtus communis* L.	*α*-pinene (48.9%) 1,8-cineole (15.3%)	NPR	NPR	941	NPR	NPR	NPR	[42]
	α-pinene (24.83%) 1,8-cineole (10.8%)	NPR	NPR	794.75	NPR	NPR	NPR	[43]
Garlic *Allium sativum* L.	diallyl trisulfide (44.21%) diallyl disulfide (22.08%) allyl methyl trisulfide (9.72%)	NPR	146.40 ± 0.03	124.60	NPR	159.60 ± 0.02	NPR	[44]
	NPR	NPR	NPR	300	NPR	NPR	NPR	[26]
	diallyl disulfide (49.1%) diallyl trisulfide (30.38%)	NPR	NPR	165.80 ± 0.20	NPR	NPR	NPR	[45]
Cardamom *Elettaria cardamomum* (L.) Maton	NPR	NPR	NPR	29.53	NPR	NPR	NPR	[36]
Mandarin *Citrus reticulata* Blanco	limonene (76.5%) *p*-cymene (16.7%)	NPR	105	890	NPR	NPR	NPR	[47]
	γ-terpinene (47.89%) methyl 2-(methylamino)benzoate (13.17%)	NPR	NPR	79.84	NPR	NPR	NPR	[48]
Hyssop *Hyssopus officinalis* L.	camphor (23.61%) β-pinene (21.91%)	NPR	NPR	11.22	NPR	NPR	NPR	[49]
	isopinocamphone	NPR	NPR	16.37	NPR	NPR	NPR	[50]

	(57.27%) β-pinene (7.23%) 4-carvomenthenol (7.13%)							
Grapefruit *Citrus paradisi* Macfad.	limonene (93.33%)	NPR	NPR	22060	NPR	15720	NPR	[51]
	limonene (56.31%) myrcene (35.83%)	NPR	98	12420	NPR	NPR	NPR	[48]
Lemongrass *Cymbopogon citratus* (DC.) Stapf	NPR	NPR	794.80 ± 0.28	601.60 ± 0.53	NPR	253.20 ± 0.34	NPR	[26]
Siberian Pine *Abies sibirica* Ledeb.	NPR	NPR	NPR	NPR	NPR	NPR	NPR	
Camphor *Cinnamomum camphora* (L.) J. Presl	neral (28.7-34.1%) citral (31.8-44.7%)	NPR	NPR	28133	NPR	117220	NPR	[52]
		NPR	NPR	12229	NPR	66900	NPR	[52]
		NPR	NPR	7527	NPR	37870	NPR	[52]
		NPR	NPR	7065	NPR	29910	NPR	[52]
Cade *Juniperus oxycedrus* L	*α*-pinene (67.33%) 3-carene (7.21%)	NPR	NPR	22.14±1.16 6.56	NPR	NPR	NPR	[53]
	NPR	NPR	NPR	6.56	NPR	NPR	NPR	[54]
Cedar leaves *Thuja occidentalis* L.	NPR	NPR	NPR	NPR	NPR	NPR	NPR	
Ginger *Zingiber officinale* Roscoe	NPR	NPR	363.90 ± 0.24	129.40 ± 0.26	NPR	275 ± 0.82	NPR	[26]
Cumin *Cuminum cyminum* L.	4-isopropylbenzaldehyde (30.42–33.24 %) *p*-mentha-1,4-dien-7-al (20.54–28.36 %)	NPR	17020	3320	NPR	NPR	NPR	[55]
Patchouli *Pogostemon cablin* Benth.	patchouli alcohol (18.12%) (+)-*γ*-curcumene (35.07%)	NPR	NPR	22.45 ± 0.30	NPR	NPR	NPR	[57]
	patchouli alcohol (26.31%) (+)-*γ*-curcumene (34.79%)	NPR	NPR	19.87 ± 0.40	NPR	NPR	NPR	[57]

Orange bitter *Citrus aurantium* L.	limonene (57.57%) linalool (8.01%)	NPR	20.62 ± 2.36	1040 ± 0.9	NPR	25.31 ± 2.66	NPR	[56]
	D-limonene (76.00-89.17%)	NPR	NPR	33.01 ± 1.71	NPR	25.31 ± 2.66	NPR	[58]
	linalool (59%) linalyl acetate (23%)	NPR	NPR	4786	652	NPR	NPR	[95]
Eucalyptus *Eucalyptus globulus* Labill.	1,8-cineole (13.23%) *p*-cymene (32.19-37.82%)	NPR	2000	740	NPR	NPR	NPR	[24]
	1,8-cineole (95.61%)	NPR	NPR	57	NPR	NPR	NPR	[59]
	1,8-cineole (38%) limonene (58%)	NPR	NPR	NPR	1109	NPR	NPR	[95]
Pine silvestre natural	NPR	NPR	NPR	NPR	NPR	NPR	NPR	
Bergamot *Citrus limon* (L.) Osbeck (syn. *Citrus* × *bergamia* Risso & Poit.)	limonene (31.58%) linalool (21.47%)	NPR	NPR	212	NPR	NPR	NPR	[60]
Juniper *Juniperus communis* L.	α-pinene (35.4%) myrcene (15.3%)	NPR	NPR	944	NPR	NPR	0.024	[61]
Birch *Betula lenta* L.	NPR	NPR	NPR	NPR	NPR	NPR	NPR	
Fennel *Foeniculum vulgare* Mill.	anethole (15.23%-90.11%) estragole (4.00-63.72%) fenchone (0.03-12.62%) limonene (1.05-13.04%)	NPR	NPR	11830 - 36900	NPR	7650 - 20130	NPR	[63]
	anethole (64-75.5%) α-phellandrene (11.0%) fenchone (4.8-13.7%) estragole (9.5-10.3%)	NPR	NPR	12370 - 37200	NPR	NPR	NPR	[64]
	anethole	NPR	NPR	NPR	652	NPR	NPR	[95]

	(75%)							
Cedar fruit<br>*Citrus medica* L.	limonene<br>(33.60%)<br>myristicin<br>(24.36%)	NPR	NPR	19.59 ± 0.01	NPR	NPR	NPR	[65]
Lemon<br>*Citrus limon* (L.) Osbeck	limonene<br>(67.1%)<br>α-pinene<br>(11.0%)<br>α-terpinene<br>(8.0%)	NPR	730	15.056	NPR	NPR	NPR	[66]
	limonene<br>(67.1%)<br>α-pinene<br>(11.0%)<br>α-terpinene<br>(8.0%)	NPR	113.63 - 180.90	660	NPR	NPR	NPR	[67]
	D-limonene<br>(48.56-53.44%)<br>β-pinene<br>(17.37-18.29%)<br>γ-terpinene<br>(12.33-12.84%)	NPR	NPR	40.57 - 100.22	NPR	NPR	NPR	[68]
	limonene<br>(99%)	NPR	NPR	16145	3193	NPR	NPR	[95]
Roman chamomile<br>*Chamaemelum nobile* (L.) All.	chamazulene<br>(27.80 %)<br>β-pinene<br>(7.93 %)<br>1,8-cineole<br>(7.51 %)	NPR	NPR	195.8	NPR	NPR	NPR	[69]
Savory<br>*Satureja montana* L.	carvacrol<br>(41.5%)<br>*p*-cymene<br>(11.0%)<br>thymol<br>(8.6%)	NPR	NPR	18.7	NPR	NPR	0.007	[70]
	carvacrol<br>(53.35%)<br>γ-terpinene<br>(13.54%)<br>*p*-cymene<br>(13.03%)	NPR	NPR	410.5 ± 4.27	NPR	NPR	NPR	[62]
Rosemary<br>*Rosmarinus officinalis* L.	α-pinene<br>(24.6%)<br>1,8-cineole<br>(14.1%)<br>camphor<br>(13.5%)	NPR	195.90 ± 0.24	444.30 ± 0.58	NPR	484.10 ± 0.58	NPR	[71]
		NPR	NPR	24800	NPR	NPR	NPR	[26]
	1,8-cineole<br>(42.86-46.76%)<br>camphor<br>(16.26-23.42%)	NPR	NPR	16450 - 23800	7024 - 13340	NPR	NPR	[96]

Ceylon cinnamon peel *Cinnamomum verum* J. Presl	cinnamaldehyde (58.7%) linalool (5.8%) eugenol (4.9 %)	229.15 ± 29.54	25.18 ± 0.03	410	NPR	11.42 ± 0.10	NPR	[72]
	NPR	NPR	NPR	7.17 ± 0.17	NPR	NPR	NPR	[26]
	cinnamicaldehyde (35.04%) 1,1-dimethoxyethane (64.50%)	NPR	68380	39800	NPR	NPR	NPR	[35]
Eucalyptus globulus	NPR	NPR	NPR	NPR	NPR	NPR	NPR	[24] [59] [95]
Orange sweet *Citrus sinensis* (L.) Osbeck	limonene (88.94%)	NPR	410	12	NPR	NPR	NPR	[59]
Niaouly *Melaleuca quinquenervia* (Cav.) S.T.Blake	1,8-cineole (31%) 2-(4-methylphenyl)propan-2-ol (19.7%) *p*-cymene (16.5%) α-terpineol (9.91%)	NPR	NPR	44500	NPR	NPR	NPR	[95]
Artemisia *Artemisia vulgaris* L.	camphor (39.9%) β-thujone (15.63%)	NPR	NPR	42000	NPR	NPR	NPR	[75]
Cajeput *Melaleuca cajuputi* Powell	1,8-cineole (up to 43.0%) viridiflorol (24.2-47.6%)	NPR	NPR	2400	NPR	NPR	NPR	[76]
Black pepper *Piper nigrum* L.	caryophyllene (25.38%) limonene (15.64%) sabinene (13.63%) 3-carene (9.34%)	NPR	NPR	103.3 - 316.27	NPR	NPR	NPR	[77]
White thyme *Thymus vulgaris* L.	carvacrol (45,85%) borneol (22,46%)	350710	NPR	5833	NPR	6460	NPR	[78]
	NPR	NPR	NPR	4050	NPR	NPR	NPR	[35]
	NPR	NPR	NPR	9.69	NPR	NPR	NPR	[79]

	thymol (42.13%)	NPR	NPR	990 ± 20	NPR	NPR	NPR	[34]
	p-cymene (24%) borneol (16%) thymol (12%) carvacrol (16%)	NPR	NPR	259	116	NPR	NPR	[95]
Marjoram *Origanum marjorana* L.	4-carvomenthenol (32.1-33.35%) linalool (13.8-15.37%) γ-terpinene (9.5%) *p*-cymene (6.9%)	NPR	374.80 ± 0.21	524.00 ± 3.42	NPR	162.00 ± 0.12	NPR	[26]
	NPR	NPR	372.72 ± 0.84	225.61 ± 0.05	NPR	NPR	NPR	[80]
Clove *Syzygium aromaticum* (L.) Merr. & L. M. Perry	eugenol (82.43%) caryophyllene (8.97%)	NPR	820	2.55 ± 0.40	NPR	5.81 ± 1.35	NPR	[26]
	NPR	NPR	5.96 ± 0.71	NPR	NPR	NPR	NPR	[35]
Cypress *Cupressus sempervirens* L.	α-pinene (47.51%)	NPR	NPR	NPR	NPR	176.45	NPR	[81]
	α-pinene (29.21%) 3-carene (18.92%) cedrol (12.25%) isoterpinene (7.66%)	NPR	NPR	NPR	NPR	NPR	NPR	[82]
	α-pinene (49%) 3-carene (18%) limonene (32%)	NPR	NPR	8245	766	NPR	NPR	[95]
Nutmeg natural *Myristica fragrans* Houtt.	sabinene (42.3 %)	NPR	NPR	NPR	NPR	NPR	NPR	[83]
Peppermint *Mentha piperita* L.	menthol (45.4%) menthone (21.8%)	NPR	257.40 ± 0.88	6.88 ± 0.13	NPR	34.08 ± 0.13	NPR	[26]
		NPR	NPR	58410	NPR	NPR	NPR	[84]
		NPR	NPR	70290	NPR	NPR	NPR	[33]

Verbena *Aloysia citriodora* Palau	citral (18.7–21.1%) neral (15.3–16.2%)	NPR	NPR	9583	NPR	3080	NPR	[85]
	neral (17.2%) citral (26.4%)	NPR	NPR	6300	NPR	NPR	NPR	[86]
Basil *Ocimum basilicum* L.	linalool (39.9%)	NPR	1092.00 ± 1.61	309.60 ± 0.37	NPR	756.0 ± 3.36	NPR	[87]
	estragole (27.82%) linalool (25.35%)	NPR	NPR	2	NPR	NPR	NPR	[26]
	eugenol (25.85%) linalool (13.41%)	NPR	NPR	210	NPR	NPR	NPR	[33]
Palmarosa *Cymbopogon martini* (Roxb.) W.Watson	NPR	NPR	NPR	125	NPR	NPR	NPR	[88]
Laurel *Laurus nobilis* L.	1,8-cineole (42.2%) α-pinene (16.7%) β-pinene (13.6%)	NPR	684.90 ± 1.03	152.40 ± 0.63	NPR	49.48 ± 0.06	NPR	[26]
	NPR	NPR	NPR	0.18 ± 0.04	NPR	NPR		[22]
Natural anise pure *Pimpinella anisum* L.	anethole (84.21%) estragole (3.82%)	NPR	58650	114.87	NPR	NPR	NPR	[89]
	anethole (94.82%)	NPR	60	118	NPR	NPR	NPR	[90]
Incense *Boswellia* spp.	NPR	NPR	NPR	NPR	NPR	NPR	NPR	
Mentha suaveolens (Sicily) *Mentha suaveolens* Ehrh.	rotundifolone (74.69%)	697.45	350	360	NPR	NPR	NPR	[91]
	menthol (31.28%) isomenthol (14.28%) (+)-pulegone (9.03%)	NPR	NPR	64.76 ± 2.24	NPR	NPR	NPR	[92]
		NPR	NPR	200	NPR	NPR		[92]
		NPR	NPR	16320	NPR	NPR		[92]
	p-cymene (18.9%) carvacrol	NPR	NPR	103	NPR	NPR	NPR	[93]

Coridothymus capitatus (Sicily) *Thymbra capitata* (L.) Cav. (syn. *Thymus capitatus* (L.) Hoffmanns. & Link)	(13.4%) geranyl acetate (12.2%) borneol (10.2%)<br>NPR	NPR	NPR	102 ± 1.01	NPR	NPR	NPR	[104]
Thymus vulgaris (Sicily) *Thymus vulgaris* L.	NPR	NPR	NPR	NPR	NPR	NPR	NPR	[78] [35] [79] [34]
Origanum hirtum (Sicily) *Origanum vulgare* subsp. *hirtum* (Link) Ietsw.	NPR	NPR	NPR	NPR	NPR	NPR	NPR	

[a]Total antioxidant capacity [b]Metal ion chelating capacity [c]Neutralization of DPPH radical [d]Interuption of lipid peroxidation [e]Neutralization of ABTS cation radical [f]Neutralization of hydroxyl radical [g]Not previously reported.

Table S2. Herein investigated essential oils chemical compositions.

EOs	Components (%)
Chamomile Morocco	eucalyptol (2.03)<br>linalool (0.31)<br>α-pinene (14.39)<br>myrtenol (0.35)<br>*o*-cymene (0.25)<br>pinocamphone (0.34)<br>4-carvomenthenol (0.41)<br>limonene (6.75)<br>myrcene (1.45)<br>isobornyl isovalerate (3.05)<br>borneol (1.20)<br>spathulenol (4.03)<br>bornyl butyrate (4.29)<br>artemisia alcohol (1.32)<br>isopinocarveol (7.43)<br>cedrelanol (2.63)<br>δ-cadinene (0.52)<br>caryophyllene oxide (1.29)<br>caryophyllene (1.04)<br>caryophyllene oxide (5.23)<br>yomogi alcohol (1.29)<br>germacrene D (4.54)<br>*trans*-2,7-dimethyl-4,6-octadien-2-ol (33.21)<br>sativene (0.89)<br>β-elemene (1.69)
Clary sage	linalool (19.93)<br>*p*-menth-1-en-8-ol (3.84)<br>copaene (0.59)<br>linalol oxide (2.15)<br>linalyl anthranilate (60.02)<br>myrcene (0.27)<br>α-bergamotene (0.77)<br>geraniol (1.67)<br>neryl acetate (5.72)<br>caryophyllene oxide (2.33)<br>caryophyllene (2.03)<br>β-bisabolene (0.67)
Sage oil	camphor (21.32)<br>eucalyptol (11.69)<br>bornyl acetate (2.64)<br>camphene (3.46)<br>α-pinene (3.02)<br>γ-terpinene (0.51)<br>*o*-cymene (1.30)<br>chrysanthone (32.97)<br>4-carvomenthenol (0.61)<br>β-pinene (1.42)<br>limonene (1.28)<br>borneol (4.62)<br>ledol (0.22)

	caryophyllene oxide (0.34)
	caryophyllene (6.83)
	humulene (7.46)
	humulene epoxide II (0.31)
Red thyme	eucalyptol (0.25)
	linalool (5.16)
	α-pinene (0.38)
	thymol (66.31)
	γ-terpinene (2.27)
	α-terpinene (0.37)
	p-cymene (10.46)
	carvacrol (7.20)
	4-carvomenthenol (1.85)
	thymol methyl ether(0.23)
	p-menth-1-en-8-ol (0.22)
	limonene (0.29)
	myrcene (0.46)
	borneol (1.53)
	α-citral (0.12)
	caryophyllene (2.32)
Tea tree	eucalyptol (14.90)
	α-pinene (11.15)
	γ-terpinene (11.80)
	α-terpinene (4.57)
	o-cymene (3.47)
	4-carvomenthenol (37.49)
	terpinolene (1.67)
	β-pinene (2.45)
	p-menth-1-en-8-ol (8.10)
	limonene (1.99)
	myrcene (0.20)
	ledol (0.44)
	linalool oxide (0.23)
	longifolene (0.24)
	viridiflorene (1.07)
	α-gurjunene (0.24)
Mountain pine	4-isopropylbenzaldehyde (0.55)
	bornyl acetate (13.44)
	linalool (0.18)
	α-pinene (12.52)
	o-cymene (2.14)
	β-phellandrene (6.98)
	2-(4-methylphenyl)propan-2-ol (0.85)
	β-pinene (7.63)
	D-carvone (0.28)
	p-menth-1-en-8-ol (0.44)
	copaene (0.92)
	limonene (10.95)
	3-carene (10.75)
	4-isopropyl-2-cyclohexenone (3.70)
	β-cubebene (1.17)
	isopinocarveol (0.47)
	(+)-*cis*-verbenol (0.46)

	δ-cadinene (1.61)
	trans-2-caren-4-ol (0.59)
	caryophyllene (21.41)
	humulene (1.20)
	trans-calamenene (0.67)
	α-muurolene (1.08)
Geranium Bourbon	acetic acid (4.97)
	linalool (9.65)
	α-pinene (0.52)
	1-indanone (3.77)
	isomenthone (4.56)
	citronellyl formate (8.00)
	heptanoic acid (1.21)
	citronellyl butyrate (1.55)
	citronellol (26.30)
	hexanoic acid (0.64)
	6,10,14-trimethylpentadecan-2-one (0.43)
	o-cymene (1.35)
	β-pinene (1.23)
	limonene (8.36)
	linalyl anthranilate (6.78)
	rose oxide (0.95)
	myrcene (0.35)
	geraniol (12.91)
	geraniol formate (3.84)
	geranyl propionate (0.79)
	2,6-dimethyl-2,6-octadiene (0.62)
	geranyl isobutyrate (1.23)
Oregano	eucalyptol (0.37)
	linalool (2.43)
	α-pinene (0.37)
	γ-terpinene (1.93)
	α-terpinene (0.33)
	p-cymene (6.80)
	carvacrol (76.54)
	4-carvomenthenol (0.54)
	limonene (0.54)
	myrcene (0.77)
	borneol (0.45)
	caryophyllene oxide (2.26)
	caryophyllene (6.67)
Coriander	benzyl benzoate (6.41)
	camphor (5.67)
	eucalyptol (0.45)
	linalool (66.67)
	camphene (0.45)
	α-pinene (3.41)
	γ-terpinene (0.93)
	benzyl salicyclate (2.20)
	o-cymene (2.51)
	4-carvomenthenol (0.24)
	β-pinene (0.31)
	p-menth-1-en-8-ol (0.82)

	linalol oxide (0.62)
	limonene (1.75)
	myrcene (0.45)
	2,6-dimethyl-3,7-octadiene-2,6-diol (0.23)
	geraniol (1.71)
	neryl acetate (5.18)
Lavander	eucalyptol (2.37)
	linalool (34.66)
	α-pinene (0.22)
	linalyl acetate (41.43)
	4-carvomenthenol (3.77)
	3-octanol (0.31)
	p-menth-1-en-8-ol (1.27)
	hexyl isovalerate (0.46)
	1-octen-3-ol (0.25)
	limonene (0.52)
	lavandulyl acetate (4.34)
	myrcene (0.69)
	borneol (1.44)
	3-octanone (0.93)
	geraniol (0.39)
	caryophyllene (2.73)
	(*E*)-β-farnesene (1.88)
	β-ocimene (2.35)
Myrtle	eucalyptol (40.78)
	linalool (3.74)
	α-pinene (17.39)
	methyleugenol (1.98)
	isobutyl isobutyrate (0.28)
	o-cymene (5.26)
	4-carvomenthenol (0.63)
	2-(4-methylphenyl)propan-2-ol (0.73)
	β-pinene (0.25)
	p-menth-1-en-8-ol (3.85)
	limonene (7.15)
	linalyl anthranilate (1.23)
	3-carene (0.24)
	myrcene (0.22)
	isopinocarveol (0.23)
	isobutyl 2-methylbutyrate (0.54)
	α-terpineol acetate (1.91)
	trans-*p*-menth-2-ene-1,4-diol (0.32)
	geraniol (0.67)
	neryl acetate (9.71)
	caryophyllene (2.18)
	humulene (0.47)
	γ-elemene (0.24)
Garlic	eucalyptol (0.30)
	diallyl sulfide (3.76)
	diallyl trisulfide (2.15)
	diallyl disulphide (57.65)
	dimethyl trisulfide (0.55)
	limonene (0.53)

	allyl methyl trisulfide (26.06)<br>allyl methyl disulfide (2.71)<br>1,2-dithiolane (0.54)<br>3-vinyl-1,2-dithiacyclohex-4-ene (2.65)<br>1,1-dioxide 1,2-dithiolane (2.77)<br>*trans*-3,5-diethyl-1,2,4-trithiolane (0.34)
Cardamom	eucalyptol (34.68)<br>linalool (5.01)<br>α-pinene (1.28)<br>*cis*-β-terpineol (0.67)<br>*o*-cymene (0.36)<br>β-pinene (0.27)<br>*p*-menth-1-en-8-ol (1.87)<br>sabinene (0.35)<br>limonene (2.02)<br>linalyl anthranilate (8.18)<br>myrcene (1.07)<br>α-terpinyl acetate (41.79)<br>geraniol (1.13)<br>nerolidol (1.33)
Mandarin	α-pinene (2.36)<br>γ-terpinene (19.03)<br>*o*-cymene (7.61)<br>terpinolene (0.90)<br>β-pinene (1.33)<br>β-terpineol (0.27)<br>sabinene (0.22)<br>limonene (66.71)<br>myrcene (1.56)
Hyssop	eucalyptol (0.57)<br>linalool (18.04)<br>α-pinene (8.61)<br>estragole (0.24)<br>myrtenol (0.96)<br>*o*-cymene (0.53)<br>pinocamphone (52.97)<br>4-carvomenthenol (0.93)<br>β-pinene (6.37)<br>*p*-menth-1-en-8-ol (0.85)<br>sabinene (0.58)<br>limonene (1.39)<br>myrcene (0.26)<br>myrtenal (0.46)<br>2,3-pinanediol (0.62)<br>borneol (1.08)<br>elemol (1.13)<br>spathulenol (1.11)<br>isopinocarveol (0.34)<br>caryophyllene oxide (0.57)<br>caryophyllene (1.32)
Grapefruit	α-pinene (0.42)<br>carveol (5.20)<br>*o*-cymene (0.28)

	D-carvone (4.73)
	limonene (78.20)
	myrcene (0.26)
	limonene-1,2-diol (0.74)
	cis-p-mentha-2,8-dien-1-ol (1.88)
	trans-limonene oxide (5.85)
	caryophyllene oxide (0.35)
	trans-p-mentha-2,8-dienol (2.09)
Lemongrass	eugenol (0.85)
	linalool (0.36)
	citronellal (38.81)
	citronellol (18.96)
	citronellyl acetate (2.89)
	isopulegol (0.68)
	myrcene (3.02)
	elemol (2.54)
	cedrelanol (0.59)
	δ-cadinene (0.90)
	geraniol (24.82)
	α-citral (3.21)
	caryophyllene (0.22)
	germacrene D (0.67)
	germacrene-D-4-ol (0.29)
	γ-cadinene (0.32)
	δ-elemene (0.86)
Siberian pine	*p*-cymen-7-ol (0.24)
	camphor (0.41)
	bornyl acetate (55.01)
	camphene (18.10)
	α-pinene (8.37)
	o-cymene (0.45)
	santene (0.97)
	β-phellandrene (1.13)
	2-(4-methylphenyl)propan-2-ol (0.67)
	β-pinene (1.61)
	p-menth-1-en-8-ol (0.44)
	limonene (3.89)
	borneol (2.73)
	tricyclene (1.46)
	4-isopropyl-2-cyclohexenone (0.58)
	α-bisabolol (0.41)
	caryophyllene oxide (1.25)
	caryophyllene (0.87)
	humulene (0.53)
	β-bisabolene (0.25)
	humulene epoxide II (0.62)
Camphor	palmitic acid (1.59)
	eucalyptol (56.98)
	camphene (0.39)
	α-pinene (0.38)
	estragole (0.32)
	m-cymene (13.40)
	β-pinene (3.06)

	sabinene (0.56)<br>limonene (22.81)<br>*trans-p*-menth-2-ene-1,4-diol (0.52)
Cade	2-methyl-phenol (1.25)<br>*m*-cresol (1.40)<br>2-methoxy-phenol (4.21)<br>phenol (1.05)<br>creosol (9.32)<br>2-methoxy-4-propylphenol (3.02)<br>3-methyl-1,2-cyclopentanedione (1.17)<br>4-ethyl-2-methoxyphenol (7.26)<br>cedrol (0.50)<br>*α*-curcumene (1.08)<br>cedrelanol (0.45)<br>δ-cadinene (18.65)<br>cubenol (2.66)<br>isoledene (3.70)<br>8,14-cedranoxide (0.30)<br>4,5,9,10-dehydro-isolongifolene (2.36)<br>isoeugenol (1.90)<br>caryophyllene (1.04)<br>humulene (1.58)<br>*trans*-calamenene (17.44)<br>cedrene (8.83)
Cedar leaves	camphor (3.75)<br>eucalyptol (0.27)<br>bornyl acetate (0.65)<br>camphene (0.32)<br>*α*-pinene (0.40)<br>chrysanthone (90.30)<br>4-carvomenthenol (0.71)<br>fenchone (2.70)<br>sabinene (0.31)<br>limonene (0.27)<br>myrcene (0.31)
Ginger<br>*Zingiber officinale*<br>Roscoe	eucalyptol (6.99)<br>linalool (0.31)<br>camphene (5.73)<br>*α*-pinene (1.70)<br>6-methyl-5-hepten-2-one (0.61)<br>*p*-menth-1-en-8-ol (0.74)<br>copaene (0.59)<br>limonene (1.15)<br>myrcene (0.63)<br>borneol (1.09)<br>β-bergamotene (0.37)<br>β-eudesmol (0.27)<br>elemol (0.53)<br>*α*-curcumene (31.56)<br>zingiberene (29.85)<br>geraniol (0.43)<br>*α*-farnesene (4.33)<br>nerolidol (0.61)

	γ-cadinene (0.68)
	β-elemene (1.24)
	β-bisabolene (10.61)
Cumin	*p*-cymen-7-ol (0.50)
	4-isopropylbenzaldehyde (37.92)
	α-pinene (0.35)
	α-phellandrene (1.02)
	α-terpinene (13.86)
	p-cymene (7.56)
	carvacrol (0.29)
	β-phellandrene (0.30)
	2-(4-methylphenyl)propan-2-ol (0.21)
	β-pinene (5.56)
	sabinene (0.20)
	limonene (0.32)
	myrcene (0.37)
	carotol (1.34)
	caryophyllene oxide (0.51)
	caryophyllene (0.70)
	(*E*)-β-farnesene (0.90)
	2-caren-10-ol (28.10)
Patchouli	β-guaiene (19.04)
	ledol (1.06)
	β-patchoulene (2.05)
	seychellene (6.84)
	α-patchoulene (5.87)
	ledene oxide-(I) (0.45)
	α-panasinsene (0.29)
	caryophyllene oxide (0.57)
	caryophyllene (3.19)
	α-guaiene (15.27)
	patchouli alcohol (45.36)
Orange bitter	linalool (0.39)
	α-pinene (0.47)
	γ-terpinene (0.11)
	decanal (0.32)
	linalyl acetate (0.61)
	β-pinene (2.70)
	sabinene (0.25)
	limonene (95.15)
Eucalyptus	eucalyptol (83.86)
	α-pinene (1.11)
	p-cymene (8.11)
	β-pinene (0.19)
	D-carvone (0.33)
	limonene (6.12)
	myrcene (0.28)
Pine silvestre natural	bornyl acetate (1.37)
	camphene (0.86)
	α-pinene (22.82)
	myrtenol (0.22)
	o-cymene (0.49)
	terpinolene (2.01)

	2-(4-methylphenyl)propan-2-ol (1.61)
	β-pinene (17.11)
	β-terpineol (0.89)
	limonene (11.22)
	3-carene (16.56)
	isopinocarveol (0.35)
	(+)-*cis*-verbenol (0.25)
	longifolene (1.98)
	α-cubebene (0.49)
	caryophyllene oxide (3.54)
	caryophyllene (16.74)
	humulene (1.22)
	humulene epoxide II (0.28)
Bergamot	linalool (13.83)
	α-pinene (0.74)
	carveol (0.25)
	o-cymene (6.47)
	β-pinene (4.57)
	D-carvone (0.36)
	sabinene (0.54)
	limonene (26.11)
	linalyl anthranilate (45.28)
	myrcene (0.67)
	trans-limonene oxide (0.26)
	neryl acetate (0.68)
	β-bisabolene (0.23)
Juniper	α-pinene (27.08)
	γ-terpinene (0.97)
	o-cymene (3.00)
	4-carvomenthenol(6.29)
	terpinolene (0.91)
	2-(4-methylphenyl)propan-2-ol (0.60)
	β-pinene (3.64)
	p-menth-1-en-8-ol (0.64)
	sabinene (5.95)
	copaene (1.03)
	limonene (7.52)
	myrcene (9.27)
	spathulenol (0.81)
	cedrelanol (0.36)
	β-cadinene (0.35)
	α-cubebene (0.90)
	caryophyllene oxide (2.91)
	caryophyllene (13.69)
	(*E*)-β-farnesene (0.60)
	humulene (3.58)
	trans-calamenene (0.93)
	γ-elemene (1.96)
	γ-cadinene (1.53)
	β-elemene (3.60)
	4-epi-cubebol (1.12)
	α-muurolene (0.78)
Birch	2-methyl-phenol (1.15)

	m-cresol (1.29)<br>2-methoxy-phenol (6.64)<br>2-methoxy-4-methylphenol (7.20)<br>cadalene (5.54)<br>(-)-β-cadinene (0.74)<br>1,8-dimethylnaphthalene (4.56)<br>2-methoxy-4-propylphenol (2.73)<br>3-methyl-1,2-cyclopentanedione (0.81)<br>benzocycloheptatriene (0.87)<br>cedrelanol (1.44)<br>δ-cadinene (18.45)<br>dihydrocurcumene (3.39)<br>4,5,9,10-dehydro-isolongifolene, (1.69)<br>isoeugenol (1.59)<br>cadina-1,4-diene (2.69)<br>*trans*-calamenene (18.12)<br>cedrene (8.18)<br>3,4-dihydrocadalene (5.76)<br>4-epi-cubebol (2.77)<br>*α*-muurolene (4.38)
Fennel	camphor (0.22)<br>*α*-pinene (0.97)<br>estragole (2.20)<br>*o*-cymene (0.40)<br>fenchone (3.37)<br>limonene (3.40)<br>4-methoxyphenylacetone (4.73)<br>4-methoxybenzaldehyde (6.34)<br>myrcene (0.20)<br>*trans*-*α*-bergamotene (0.11)<br>anethole (78.05)
Cedar fruit	linalool (6.36)<br>*α*-pinene (0.44)<br>carveol (0.39)<br>*o*-cymene (17.39)<br>β-pinene (3.22)<br>D-carvone (1.37)<br>sabinene (0.21)<br>limonene (32.20)<br>linalyl anthranilate (25.03)<br>myrcene (0.40)<br>trans-*α*-bergamotene (0.33)<br>*cis*-*p*-mentha-2,8-dien-1-ol (0.44)<br>*trans*-limonene oxide (1.15)<br>*α*-citral (6.98)<br>neryl acetate (1.59)<br>caryophyllene oxide (0.44)<br>geranic acid (0.95)<br>caryophyllene (0.22)<br>β-bisabolene (0.46)<br>*trans*-*p*-mentha-2,8-dienol (0.43)
Lemon	*α*-pinene (1.11)<br>carveol (0.33)

	γ-terpinene (1.11)<br>*o*-cymene (7.89)<br>β-pinene (7.21)<br>sabinene (1.02)<br>limonene (59.22)<br>myrcene (0.70)<br>myrtenal (0.21)<br>limonene-1,2-diol (2.85)<br>*cis*-*p*-mentha-2,8-dien-1-ol (0.36)<br>*trans*-limonene oxide (0.73)<br>geraniol (2.24)<br>α-citral (11.11)<br>neryl acetate (1.87)<br>caryophyllene oxide (0.44)<br>geranic acid (1.58)
Roman chamomile	eucalyptol (2.50)<br>bornyl acetate (1.00)<br>camphene (0.40)<br>α-pinene (18.90)<br>*o*-cymene (0.20)<br>pinocamphone (0.30)<br>4-carvomenthenol (0.30)<br>terpinolene (0.20)<br>sabinene (0.40)<br>limonene (7.30)<br>myrcene (1.70)<br>borneol (0.80)<br>spathulenol (1.30)<br>bornyl butyrate (1.70)<br>artemisia alcohol (1.70)<br>isopinocarveol (4.80)<br>pinocarvone (0.30)<br>caryophyllene oxide (0.70)<br>yomogi alcohol (1.30)<br>germacrene D (1.80)<br>*trans*-2,7-dimethyl-4,6-octadien-2-ol (49.30)<br>sativene (0.70)<br>δ-elemene (1.50)
Savory	eucalyptol (0.41)<br>linalool (1.48)<br>α-pinene (0.28)<br>thymol (1.55)<br>γ-terpinene (2.30)<br>α-terpinene (0.54)<br>*p*-cymene (5.63)<br>carvacrol (71.47)<br>4-carvomenthenol (1.06)<br>*p*-menth-1-en-8-ol (0.25)<br>1-octen-3-ol (0.93)<br>limonene (0.24)<br>myrcene (0.32)<br>borneol (0.95)<br>carvacrol acetate (0.34)

	spathulenol (0.35)<br>β-cadinene (0.78)<br>caryophyllene oxide (0.98)<br>caryophyllene (5.02)<br>γ-cadinene (1.03)<br>β-bisabolene (4.08)
Rosemary	camphor (21.44)<br>eucalyptol (53.20)<br>eugenol (0.68)<br>bornyl acetate (0.43)<br>camphene (0.85)<br>α-pinene (6.66)<br>*o*-cymene (0.43)<br>4-carvomenthenol (0.35)<br>β-pinene (5.28)<br>*p*-menth-1-en-8-ol (1.19)<br>limonene (1.66)<br>myrcene (0.35)<br>borneol (1.27)<br>caryophyllene (6.21)
Eucalyptus globulus	eucalyptol (85.89)<br>α-pinene (0.62)<br>carveol (0.19)<br>*o*-cymene (8.34)<br>D-carvone (0.47)<br>limonene (4.50)
Orange sweet	linalool (0.82)<br>α-pinene (0.37)<br>carveol (0.51)<br>D-carvone (0.76)<br>sabinene (0.24)<br>limonene (94.98)<br>myrcene (1.73)<br>*cis*-*p*-mentha-2,8-dien-1-ol (0.27)<br>*trans*-*p*-mentha-2,8-dienol (0.33)
Niaouly	eucalyptol (65.95)<br>linalool (0.20)<br>α-pinene (5.34)<br>γ-terpinene (0.76)<br>*o*-cymene (1.46)<br>4-carvomenthenol(0.88)<br>terpinolene (0.44)<br>β-pinene (1.48)<br>p-menth-1-en-8-ol(8.32)<br>limonene (5.87)<br>myrcene (0.71)<br>ledol (5.45)<br>β-terpineol acetate (1.37)<br>caryophyllene (1.76)
Artemisia	4-isopropylbenzaldehyde (0.41)<br>camphor (26.98)<br>eucalyptol (2.04)<br>camphene (2.98)

	α-pinene (0.20)
	γ-terpinene (0.25)
	myrtenol (0.28)
	o-cymene (1.24)
	chrysanthone (54.59)
	4-carvomenthenol (2.52)
	sabinene (0.91)
	borneol (0.87)
	artemisia alcohol (1.97)
	isopinocarveol (0.68)
	santolina triene (2.16)
	yomogi alcohol (1.61)
	germacrene D (0.33)
Cajeput	eucalyptol (52.66)
	linalool (2.24)
	α-pinene (1.47)
	γ-terpinene (0.88)
	p-cymene (18.41)
	cis-β-terpineol (0.68)
	terpinolene (0.47)
	p-menth-3-en-1-ol (0.26)
	β-pinene (0.81)
	p-menth-1-en-8-ol (7.61)
	limonene (3.55)
	myrcene (0.65)
	α-terpinyl acetate (1.19)
	caryophyllene (9.13)
Black pepper	linalool (0.58)
	α-pinene (6.92)
	p-cymene (1.08)
	4-carvomenthenol (0.36)
	β-pinene (6.26)
	sabinene (7.21)
	copaene (5.39)
	limonene (11.08)
	3-carene (5.86)
	trans-α-bergamotene (0.10)
	spathulenol (0.38)
	β-cubebene (0.70)
	δ-cadinene (0.94)
	β-selinene (0.50)
	caryophyllene oxide (9.71)
	caryophyllene (33.58)
	humulene (2.42)
	trans-calamenene (0.32)
	γ-cadinene (0.24)
	β-elemene (0.75)
	β-bisabolene (1.97)
	humulene epoxide II (0.54)
	α-muurolene (0.41)
White thyme	camphor (2.38)
	eucalyptol (1.21)
	linalool (7.51)

	camphene (1.02)<br>α-pinene (1.32)<br>thymol (44.43)<br>γ-terpinene (5.19)<br>α-terpinene (0.64)<br>*p*-cymene (18.20)<br>*cis*-β-terpineol (0.20)<br>carvacrol (6.62)<br>4-carvomenthenol (3.04)<br>thymol methyl ether (0.36)<br>β-pinene (0.35)<br>*p*-menth-1-en-8-ol (0.22)<br>1-octen-3-ol (0.52)<br>limonene (0.46)<br>myrcene (1.12)<br>borneol (2.18)<br>caryophyllene oxide (0.67)<br>caryophyllene (2.37)
Marjoram	eucalyptol (1.02)<br>linalool (40.67)<br>α-pinene (0.44)<br>*cis*-β-terpineol (15.42)<br>*o*-cymene (8.48)<br>4-carvomenthenol (23.19)<br>2-(4-methylphenyl)propan-2-ol (0.60)<br>*p*-menth-1-en-8-ol(0.44)<br>sabinene (2.62)<br>linalol oxide (0.53)<br>limonene (0.75)<br>myrcene (0.46)<br>*trans*-*p*-menth-2-ene-1,4-diol (0.78)<br>caryophyllene oxide (1.00)<br>caryophyllene (0.65)<br>sabinene hydrate (2.95)
Clove	eugenol (80.09)<br>methylsalicylate (0.22)<br>eugenol acetate (9.91)<br>caryophyllene (8.75)<br>humulene (1.03)
Cypress	eucalyptol (0.55)<br>linalool (0.73)<br>camphene (0.64)<br>α-pinene (47.69)<br>*o*-cymene (1.44)<br>4-carvomenthenol (1.52)<br>terpinolene (0.69)<br>2-(4-methylphenyl)propan-2-ol (1.21)<br>β-pinene (1.25)<br>*p*-menth-1-en-8-ol (0.38)<br>sabinene (0.68)<br>limonene (3.95)<br>3-carene (29.33)<br>verbenone (0.290

	cedrol (4.73)
	α-terpinyl acetate (3.82)
	(+)-*cis*-verbenol (0.27)
	trans-2-caren-4-ol (0.37)
	cedrene (0.48)
Nutmeg Natural	eucalyptol (2.91)
	eugenol (2.24)
	myristicin (21.86)
	linalool (0.54)
	α-pinene (12.01)
	methyleugenol (0.32)
	α-phellandrene (0.77)
	γ-terpinene (3.83)
	α-terpinene (0.64)
	o-cymene (1.64)
	4-carvomenthenol(16.55)
	terpinolene (0.68)
	β-pinene (11.07)
	p-menth-1-en-8-ol (0.73)
	sabinene (13.87)
	copaene (0.26)
	limonene (7.80)
	α-terpinyl acetate (0.15)
	isosafrole (1.55)
	isoeugenol (0.33)
	neryl acetate (0.22)
Peppermint	menthol (57.84)
	eucalyptol (0.94)
	isomenthone (33.17)
	α-terpinyl acetate (0.30)
	limonene (0.52)
	isopulegol (0.88)
	menthyl acetate (5.00)
	piperitenone (0.75)
	(+)-pulegone (0.36)
	caryophyllene (0.24)
Verbena	eucalyptol (0.62)
	linalool (1.33)
	α-pinene (1.58)
	citronellyl formate (1.15)
	citronellal (8.03)
	6-methyl-5-hepten-2-one (1.33)
	4-carvomenthenol (0.24)
	β-pinene (2.08)
	sabinene (0.47)
	copaene (0.11)
	limonene (9.51)
	myrcene (0.36)
	elemol (0.51)
	(+)-*cis*-verbenol (0.35)
	δ-cadinene (0.21)
	geraniol (6.19)
	α-citral (64.53)

	geranic acid (0.73)
	caryophyllene (0.69)
Basil	menthol (0.44)
	linalool (19.72)
	estragole (75.17)
	trans-α-bergamotene (0.86)
	α-citral (0.68)
	caryophyllene oxide (0.33)
	caryophyllene (0.35)
	(*E*)-β-farnesene (0.31)
	cis-α-bisabolene (2.15)
Palmarosa	linalool (4.07)
	D-carvone (0.58)
	farnesol (0.74)
	geraniol (76.48)
	α-citral (0.67)
	neryl acetate (13.96)
	caryophyllene (1.80)
	humulene (0.24)
	β-ocimene (1.45)
Laurel	eucalyptol (53.91)
	eugenol (1.86)
	linalool (3.02)
	α-pinene (3.18)
	methyleugenol (4.17)
	γ-terpinene (1.85)
	myrtenol (0.29)
	o-cymene (2.39)
	4-carvomenthenol (3.02)
	β-pinene (2.30)
	sabinene (3.20)
	limonene (1.99)
	α-terpinyl acetate (18.83)
Natural anise pure	linalool (0.92)
	α-pinene (0.28)
	estragole (3.77)
	4-carvomenthenol (0.25)
	p-menth-1-en-8-ol (0.22)
	limonene (1.65)
	4-methoxyphenylacetone (0.92)
	4-methoxybenzaldehyde (2.13)
	trans-α-bergamotene (0.46)
	anethole (88.51)
	isohomogenol (0.24)
	caryophyllene (0.37)
	nerolidol (0.28)
Incense	acetic acid (0.70)
	bornyl acetate (0.97)
	α-pinene (26.98)
	α-phellandrene (1.95)
	o-cymene (3.72)
	β-phellandrene (0.95)
	4-carvomenthenol(0.64)

	2-(4-methylphenyl)propan-2-ol (0.22)
	β-pinene (1.01)
	p-menth-1-en-8-ol (0.53)
	sabinene (2.82)
	copaene (2.34)
	limonene (20.32)
	3-carene (0.54)
	myrcene (2.99)
	myrtenyl acetate (0.34)
	β-eudesmol (0.34)
	ledol (0.53)
	isopinocarveol (0.93)
	cedrelanol (2.35)
	(+)-*cis*-verbenol (1.78)
	δ-cadinene (2.12)
	α-cubebene (0.37)
	δ-selinene (1.62)
	caryophyllene oxide (2.97)
	caryophyllene (9.11)
	humulene (1.96)
	γ-eudesmol (0.52)
	β-elemene (4.02)
	humulene epoxide II (1.11)
	α-selinene (1.09)
	4-epi-cubebol (1.74)
	α-muurolene (0.39)
Mentha suaveolens (Sicily)	α-pinene (1.04)
	thymol (1.21)
	γ-terpinene (0.11)
	4-carvomenthenol (0.67)
	β-pinene (3.83)
	sabinene (0.57)
	limonene (8.76)
	2-(1-methylethylidene)-cyclohexanone (1.02)
	myrtenal (0.31)
	cedrelanol (0.35)
	α-cubebene (0.52)
	(+)-pulegone (59.77)
	cubenol (0.22)
	cis-jasmone (0.61)
	caryophyllene oxide (0.19)
	caryophyllene (7.32)
	humulene (1.06)
	β-ocimene (0.15)
	germacrene D (5.17)
	cinerolon (4.64)
	trans-calamenene (0.73)
	γ-cadinene (0.19)
	α-muurolene (0.20)
	α-gurjunene (0.43)
	epi-bicyclosesquiphellandrene (0.90)
*Coridothymus capitatu*s (Sicily)	linalool (1.85)
	thymol (0.49)

	γ-terpinene (6.03)<br>γ-terpinene (1.46)<br>*p*-cymene (7.14)<br>*cis*-β-terpineol (0.22)<br>carvacrol (61.00)<br>β-phellandrene (0.33)<br>α-thujene (1.01)<br>1-octen-3-ol (0.73)<br>limonene (0.28)<br>myrcene (1.59)<br>borneol (0.60)<br>α-citral (0.22)<br>caryophyllene oxide (1.35)<br>caryophyllene (13.58)<br>humulene (0.63)<br>β-bisabolene (1.60)
Thymus vulgaris (Sicily)	eucalyptol (0.86)<br>linalool (3.70)<br>camphene (0.55)<br>α-pinene (0.94)<br>thymol (34.05)<br>γ-terpinene (1.76)<br>*p*-cymene (30.45)<br>carvacrol (0.66)<br>thymol methyl ether (10.70)<br>*p*-menth-1-en-8-ol (0.32)<br>1-octen-3-ol (0.56)<br>limonene (0.45)<br>myrcene (0.98)<br>borneol (2.54)<br>isothymol methyl ether (5.97)<br>caryophyllene oxide (1.78)<br>caryophyllene (3.45)<br>γ-cadinene (0.28)
Origanum hirtum (Sicily)	camphor (0.34)<br>eucalyptol (0.49)<br>thymol (34.70)<br>α-phellandrene (0.22)<br>γ-terpinene (17.13)<br>*p*-cymene (18.55)<br>carvacrol (0.84)<br>thymol methyl ether (4.87)<br>2-(4-methylphenyl)propan-2-ol (0.15)<br>*p*-menth-1-en-8-ol (0.21)<br>α-thujene (1.12)<br>1-octen-3-ol (0.48)<br>limonene (0.40)<br>myrcene (1.34)<br>β-bourbonene (0.35)<br>borneol (0.34)<br>thymol acetate (0.29)<br>isothymol methyl ether (7.42)<br>δ-cadinene (0.72)

caryophyllene oxide (0.58)
caryophyllene (2.86)
humulene (0.34)
γ-cadinene (1.00)
β-bisabolene (5.04)
α-muurolene (0.24)

Table S3. Total antioxidant capacity of examined EOs.

EOs	TAC[a] µg AAE/mg
Chamomile morocco	0.023±0.007[1]
Clary sage	0.062±0.013
Sage oil	0.530±0.024
Red thyme	0.030±0.011
Tea tree	0.250±0.023
Melissa	0.006±0.002
Mountain pine	0.009±0.004
Geranium Bourbon	0.008±0.002
Oregano	0.007±0.005
Ylang-ylang	0.009±0.007
Coriander	0.006±0.003
Lavander	0.006±0.002
Myrtle	0.005±0.002
Garlic	0.269±0.029
Cardamom	0.314±0.046
Mandarin	0.336±0.058
Hyssop	0.287±0.053
Grapefruit	0.005±0.004
Lemongrass	0.007±0.005
Siberian pine	0.458±0.026
Camphor	0.449±0.015
Cade	0.125±0.057
Cedar leaves	0.146±0.097
Ginger	0.005±0.004
Cumin	0.004±0.002
Patchouli	0.004±0.004
Orange bitter	0.006±0.002
Eucalyptus	0.003±0.003
Pine silvestre natural	0.004±0.002
Bergamot	0.006±0.001
Juniper	0.125±0.029
Birch	0.522±0.079
Fennel	0.008±0.002
Cedar fruit	0.131±0.067
Lemon	0.009±0.004
Roman chamomile	0.007±0.002
Savory	0.119±0.036
Rosemary	0.098±0.057
Ceylon cinnamon peel	0.006±0.004
Eucalyptus globulus	0.007±0.003
Orange sweet	0.033±0.029
Niaouly	0.048±0.041
Artemisia	0.007±0.005
Cajeput	0.058±0.036
Black pepper	1.231±0.536
White thyme	0.063±0.036
Marjoram	1.032±0.056
Clove	0.003±0.003
Cypress	0.003±0.003

Nutmeg natural	0.004±0.002
Peppermint	0.003±0.003
Verbena	0.005±0.004
Basil	0.006±0.002
Palmarosa	0.005±0.002
Laurel	0.003±0.003
Narural anise pure	0.005±0.002
Incense	0.004±0.002
Mentha suaveolens (Sicily)	0.004±0.004
*Coridotthymus capitatu*s (Sicily)	0.006±0.005
Thymus vulgaris (Sicily)	0.007±0.002
Origanum hirtum (Sicily)	0.002±0.002
AA[n]	NA
BHT[o]	NA
EDTA[p]	NA
Q[q]	NA

[a]Total antioxidant capacity

Table S4. DNA protective potential of selected 61 commercial essential oils on peroxyl radical-induced DNA damage

	Relative band density[a]							
	NC[b]	PC[c]	Quercetin[d]	25 μg/mL	50 μg/mL	100 μg/mL	200 μg/mL	400 μg/mL
Chamomile morocco	1†	0.066±0.12*‡	0.909±0.82†	0.837±0.52†	0.84±1.4†	0.877±0.58†	0.892±1.62†	0.92±1.57†
Clary sage	1†	0.068±0.32*‡	0.828±1.02†	0.935±0.92†	0.922±0.87†	0.901±0.45†	0.881±0.23†	0.859±1.06†
Sage oil	1†	0.049±0.43*‡	0.971±0.32†	0.782±1.03*†	0.779±0.78	0.818±0.54†	0.902±0.32†	0.938±1.03†
Red thyme	1†	0.128±1.23*‡	0.934±0.8†	0.945±0.58†	0.923±1.02†	0.916±0.34†	0.902±0.62†	0.849±1.4†
Tea tree	1†	0.208±1.11*‡	0.970±0.61†	0.927±0.41†	0.985±0.32†	0.981±0.43†	0.966±0.7†	0.979±1.02†
Melissa	1†	0.106±1.5*‡	0.841±0.53†	0.815±0.3†	0.823±1.73†	0.837±0.58†	0.911±0.81†	0.959±0.67†
Mountain pine	1†	0.171±0.54*‡	0.969±0.43†	0.924±1.03†	0.949±0.62†	0.964±0.73†	0.973±0.78†	0.987±1.6†
Geranium bourbon	1†	0.171±0.5*‡	0.947±0.92†	0.848±1.02†	0.905±1.11†	0.89±0.34†	0.923±0.82†	0.949±0.52†
Oregano	1†	0.097±0.32*‡	0.869±0.56†	0.807±1.23†	0.827±0.73†	0.839±0.45†	0.901±1.7†	0.949±0.85†
Ylang-ylang	1†	0.108±1.2*‡	0.917±0.78†	0.840±0.73†	0.831±0.45†	0.811±0.53†	0.809±1.86†	0.803±1.17†
Coriander	1†	0.108±0.6*‡	0.874±1.05†	0.936±0.45†	0.897±0.34†	0.899±0.2†	0.861±0.49†	0.831±1.2†
Lavander	1†‡	0.171±1.04*‡	0.886±0.54†	0.959±0.82†	0.976±0.94†	0.988±0.61†	0.997±0.35†	0.993±0.65†
Myrtle	1†	0.16±0.54*‡	0.938±0.76†	0.887±1.04†	0.903±0.63†	0.941±1.06†	0.968±0.22†	0.996±0.35†
Garlic	1†	0.23±1.43*‡	0.867±1.5†	0.927±0.9†	0.932±0.34†	0.931±0.63†	0.959±0.81†	0.984±0.25†
Cardamom	1†	0.147±1.7*‡	0.935±0.34†	0.879±0.28†	0.851±1.63†	0.848±0.55†	0.828±0.32†	0.913±0.81†
Mandarin	1†	0.221±0.61*‡	0.902±0.93†	0.932±0.43†	0.95±0.71†	0.951±0.63†	0.911±0.32†	0.887±0.54†
Hyssop	1†‡	0.163±0.9*‡	0.891±0.91†	0.88±0.43†	0.895±0.82†	0.917±1.03†	0.937±1.14†	0.958±0.54†
Grapefruit	1†	0.073±0.83*‡	0.854±0.72†	0.843±1.72†	0.836±0.62†	0.819±0.85†	0.775±1.36*†	0.765±0.91*†
Lemongrass	1†	0.141±0.47*‡	0.874±0.74†	0.853±1.37†	0.861±0.62†	0.909±1.63†	0.912±0.61†	0.879±0.96†
Siberian pine	1†	0.088±0.32*‡	0.853±0.48†	0.817±0.92†	0.828±1.36†	0.831±0.85†	0.949±0.62†	0.887±0.53†
Camphor	1†	0.264±0.51*‡	0.856±0.67†	0.879±0.9†	0.885±0.57†	0.896±0.65†	0.908±0.73†	0.935±1.3†
Cade	1†	0.097±0.43*‡	0.833±0.72†	0.810±0.81†	0.873±0.52†	0.884±1.9†	0.908±0.74†	0.942±0.71†
Cedar leaves	1†	0.108±0.56*‡	0.826±0.53†	0.856±1.31†	0.859±0.7†	0.901±0.81†	0.934±0.51†	0.941±0.21†
Ginger	1†	0.17±1.12*‡	0.855±0.72†	0.897±0.23	0.913±0.41†	0.924±1.9†	0.937±0.23†	0.944±0.64†
Cumin	1†	0.157±0.56*‡	0.95±0.77†	0.88±0.52†	0.883±0.43†	0.91±1.38†	0.927±0.82†	0.952±0.73†
Patchouli	1†	0.083±1.9*‡	0.897±0.52†	0.811±0.56†	0.829±0.53†	0.866±1.25†	0.875±1.85†	0.878±0.72†
Orange bitter	1†	0.085±1.03*‡	0.921±0.62†	0.837±0.37†	0.871±0.42†	0.889±0.61†	0.902±0.73†	0.929±1.02†
Eucalyptus	1†‡	0.113±0.18*‡	0.724±1.56*†	0.805±0.64†	0.815±0.62†	0.828±0.82†	0.847±0.72†	0.879±0.8†
Pine silvestre natural	1†	0.185±0.42*‡	0.939±0.6†	0.809±1.3†	0.842±0.34†	0.854±0.3†	0.869±1.9†	0.872±0.6†
Bergamot	1†	0.048±0.56*‡	0.919±0.73†	0.818±0.9†	0.822±0.91†	0.845±1,02†	0.859±0.23†	0.899±0.52†
Juniper	1†	0.168±0.34*‡	0.957±0.27†	0.778±1.04*†	0.805±0.43†	0.809±0.54†	0.821±0.72†	0.950±0.81†
Birch	1†	0.118±0.61*‡	0.905±0.8†	0.962±1.6†	0.93±0.53†	0.943±0.43†	0.953±0.72†	0.973±1.4†
Fennel	1†	0.175±0.7*‡	0.896±0.58†	0.897±1.2†	0.902±0.57†	0.906±1.5†	0.913±1.5†	0.946±1.36†
Cedar fruit	1†	0.106±0.32*‡	0.987±0.32†	0.971±0.5†	0.932±0.91†	0.883±0.51†	0.873±0.82†	0.866±0.41†
Lemon	1†	0.189±0.26*‡	0.899±0.72†	0.881±1.4†	0.861±1.54†	0.840±0.9†	0.83±0.92†	0.81±1.25†
Roman chamomile	1†	0.210±0.43*‡	0.83±1.46†	0.873±0.54†	0.84±0.34†	0.826±0.4†	0.816±0.57†	0.809±1.8†
Savory	1†	0.071±0.92*‡	0.902±0.92†	0.808±0.35†	0.812±0.83†	0.839±1.23†	0.85±0.57†	0.86±0.22†
Rosemary	1†	0.17±0.23*‡	0.82±0.84†	0.851±0.64†	0.86±0.56†	0.885±1.03†	0.894±0.59†	0.927±0.32†
Ceylon cinnamon peel	1†	0.101±0.34*‡	0.882±0.67†	0.898±1.37†	0.911±0.34†	0.929±0.81†	0.937±1.17†	0.941±0.59†
Eucalyptus globulus	1†	0.138±1.12*‡	0.827±0.45†	0.862±0.62†	0.866±1.9†	0.888±1.2†	0.906±0.6†	0.951±0.78†
Orange sweet	1†	0.239±1.02*‡	0.878±0.34†	0.930±1.53†	0.953±0.64†	0.95±0.82†	0.96±0.81†	0.962±1.17†
Niaouly	1†	0.184±0.32*‡	0.95±0.45†	0.926±0.54†	0.964±0.12†	0.896±1.09†	0.84±1.14	0.801±1.01†

Artemisia	1†	0.179±0.32*‡	0.826±0.85†	0.915±0.43†	0.901±1.8†	0.900±0.47†	0.928±0.77†	0.944±1.04†
Cajeput	1†	0.143±0.53*‡	0.832±0.92†	0.877±0.72†	0.884±0.42†	0.925±0.46†	0.933±0.64†	0.955±0.52†
Black pepper	1†‡	0.159±0.71*‡	0.864±0.3†	0.93±0.47†	0.898±0.32†	0.888±0.4†	0.877±1.11†	0.852±0.45†
White thyme	1†	0.133±1.02*‡	0.861±1.3†	0.831±0.8†	0.870±0.71†	0.835±1.03†	0.858±0.32†	0.879±1.02†
Marjoram	1†‡	0.106±1.15*‡	0.859±0.34†	0.869±0.26†	0.846±0.28†	0.882±0.45†	0.896±0.56†	0.94±0.62†
Clove	1†	0.146±0.52*‡	0.919±1.07†	0.894±0.71†	0.904±0.93†	0.872±1.3†	0.818±0.4†	0.779±1.5*†
Cypress	1†‡	0.104±0.34*‡	0.824±1.4†	0.815±0.3†	0.861±0.43†	0.858±0.3†	0.871±1.02†	0.910±0.8†
Nutmeg natural	1†	0.154±0.52*‡	0.853±0.48†	0.890±1.3†	0.899±0.56†	0.911±0.13†	0.921±0.34†	0.951±1.5†
Peppermint	1†	0.179±1.04*‡	0.816±0.5†	0.808±0.43†	0.817±0.26†	0.875±1.2†	0.879±1.4†	0.931±0.43†
Verbena	1†	0.113±1.72*‡	0.967±0.6†	0.916±0.69†	0.923±1.6†	0.929±0.69†	0.958±0.32†	0.974±0.48†
Basil	1†	0.162±0.42*‡	0.978±0.13†	0.907±0.6†	0.909±0.51†	0.911±0.38†	0.931±0.67†	0.949±0.45†
Palmarosa	1†‡	0.160±0.54*‡	0.945±1.02†	0.917±0.32†	0.925±0.47†	0.932±0.61†	0.943±0.32†	0.972±1.13†
Laurel	1†	0.076±0.51*‡	0.906±0.93†	0.945±1.4†	0.952±0.55†	0.973±0.54†	0.952±1.03†	0.984±0.45†
Narural anise pure	1†	0.199±0.9*‡	0.944±0.43†	0.894±1.6†	0.899±0.4†	0.926±0.54†	0.928±1.03†	0.963±0.4†
Incense	1†	0.077±0.81*‡	0.843±0.59†	0.819±1.65†	0.88±0.91†	0.908±0.5†	0.916±1.12†	0.937±1.9†
Mentha suaveolens (Sicily)	1†	0.089±1.32*‡	0.858±0.32†	0.863±1.34†	0.888±0.5†	0.898±1.01†	0.88±1.36†	0.93±1.4†
Coridotthymus capitatus (Sicily)	1†	0.114±1.03*‡	0.885±0.8†	0.878±0.43†	0.861±1.02†	0.864±0.92†	0.870±1.4†	0.882±0.71†
Thymus vulgaris (Sicily)	1†	0.172±0.43*‡	0.962±0.3†	0.904±1.11†	0.909±1.4†	0.917±0.6†	0.947±0.67†	0.983±0.3†
Origanum Hirtum (Sicily)	1†	0.091±0.52*‡	0.902±0.68†	0.868±1.3†	0.874±0.5†	0.889±0.43†	0.909±1.26†	0.938±0.45†

[a]The values are mean ± S.D. from three independent experiments [b]NC: negative control, DNA control
[c]PC: positive control, DNA damage control [d]Quercetin: 100 µg/mL, standard drug. $^{*}p < 0.05$ when compared with the negative control $^{\dagger}p < 0.05$ when compared with the positive control $^{\ddagger}p < 0.05$ when compared with the standard.

Table S5. DNA protective potential of selected 61 commercial essential oils on hydroxyl radical–induced DNA damage

	Relative band density[a]							
	NC[b]	PC[c]	Quercetin[d]	25 μg/mL	50 μg/mL	100 μg/mL	200 μg/mL	400 μg/mL
Chamomile morocco	1†‡	0.078±0.9*‡	0.723±1.32*†	0.823±1.2†	0.846±1.03†	0.850±0.9†	0.917±0.18†	0.935±0.51†
Clary sage	1†	0.06±0.61*‡	0.857±0.8†	0.956±0.12†	0.961±0.91†	0.938±0.32†	0.837±0.5†	0.781±0.67*†
Sage oil	1†‡	0.146±0.23*‡	0.735±0.5*†	0.921±0.91†	0.908±0.51†	0.851±0.6†	0.778±0.8*†	0.723±0.21*†
Red thyme	1†	0.126±0.82*‡	0.839±1.43†	0.997±0.91†	0.903±0.45†	0.894±1.43†	0.833±0.23†	0.810±1.23†
Tea tree	1†‡	0.042±0.51*‡	0.778±0.64*†	0.864±0.11†	0.848±0.22†	0.840±0.6†	0.842±0.23†	0.854±0.33†
Melissa	1†	0.036±0.24*‡	0.801±0.34†	0.818±0.91†	0.810±1.03†	0.808±1.03†	0.872±1.05†	0.844±0.38†
Mountain pine	1†	0.073±0.74*‡	0.832±0.41†	0.81±0.32†	0.870±1.23†	0.874±1.11†	0.966±0.32†	0.982±0.37†
Geranium bourbon	1†	0.104±0.61*‡	0.809±1.4†	0.908±0.43†	0.92±0.78†	0.95±0.12†	0.97±0.72†	0.99±1.43†
Oregano	1†‡	0.042±0.66*‡	0.799±0.61*†	0.858±0.28†	0.851±0.92†	0.877±0.83†	0.910±0.54†	0.939±1.04†
Ylang-ylang	1†	0.094±0.12*‡	0.872±0.25†	0.878±0.37†	0.895±0.34†	0.908±0.21†	0.931±0.23†	0.946±0.23†
Coriander	1†	0.082±0.81*‡	0.931±0.75†	0.843±0.23†	0.828±0.61†	0.805±1.3†	0.743±0.5*†	0.713±1.9*†
Lavander	1†‡	0.040±0.64*‡	0.793±1.6*†	0.893±1.18†	0.887±1.9†	0.877±0.54†	0.897±0.33†	0.931±0.24†
Myrtle	1†‡	0.054±0.12*‡	0.780±0.4*†	0.87±0.37†	0.865±0.11†	0.883±0.65†	0.892±0.57†	0.94±0.37†
Garlic	1†	0.063±0.23*‡	0.835±0.14†	0.908±0.32†	0.900±0.34†	0.868±1.23†	0.805±0.32†	0.67±0.53*†‡
Cardamom	1†	0.017±0.11*‡	0.848±0.32†	0.857±0.54†	0.854±0.81†	0.815±0.15†	0.89±0.81†	0.96±0.11†
Mandarin	1†	0.196±0.36*‡	0.879±0.35†	0.800±0.31†	0.826±0.41†	0.835±0.6†	0.843±0.4†	0.879±0.84†
Hyssop	1†‡	0.011±0.23*‡	0.724±1.3*†	0.804±1.3†	0.877±1.44†	0.930±0.16†	0.931±0.42†	0.953±0.32†
Grapefruit	1†‡	0.059±0.32*‡	0.797±0.91*†	0.916±0.32†	0.91±0.75†	0.88±0.41†	0.802±0.62†	0.806±0.54†
Lemongrass	1†	0.023±0.9*‡	0.826±0.32†	0.705±0.61*†	0.719±0.34*†	0.819±0.8†	0.836±1.41†	0.883±0.45†
Siberian pine	1†	0.205±0.83*‡	0.869±0.51†	0.751±0.8*†	0.73±0.34*†	0.735±1.43*†	0.845±1.32†	0.838±0.33†
Camphor	1†	0.051±0.61*‡	0.992±0.2†	0.954±0.43†	0.922±1.32†	0.952±0.43†	0.974±0.71†	0.984±0.61†
Cade	1†	0.134±0.81*‡	0.902±0.24†	0.893±0.32†	0.912±0.21†	0.900±0.23†	0.883±0.21†	0.866±0.32†
Cedar leaves	1†	0.144±0.76*‡	0.970±0.23†	0.951±0.5†	0.915±0.32†	0.872±0.32†	0.793±0.61*†‡	0.739±0.4*†‡
Ginger	1†‡	0.161±0.91*‡	0.73±0.41*†	0.807±1.8†	0.840±0.11†	0.869±0.32†	0.891±0.35†	0.936±0.16†‡
Cumin	1†‡	0.048±0.15*‡	0.745±0.3*†	0.787±0.24*†	0.831±0.62†	0.846±0.63†	0. 925±0.48†‡	0.968±1.54†‡
Patchouli	1†	0.089±0.42*‡	0.823±1.9†	0.847±1.8†	0.862±0.74	0.884±0.65†	0.944±0.52†	0.968±1.12†
Orange bitter	1†	0.084±0.5*‡	0.962±0.21†	0.832±1.2†	0.902±0.55†	0.907±1.9†	0.895±0.17†	0.904±1.13†
Eucalyptus	1†‡	0.059±0.71*‡	0.710±0.32*†	0.795±0.32*†	0.854±1.16†	0.892±0.31†	0.926±1.05†‡	0.973±1.03†‡
Pine silvestre natural	1†	0.056±0.14*‡	0.896±0.83†	0.893±0.84†	0.882±0.34†	0.868±0.77†	0.828±0.61†	0.789±0.23*†
Bergamot	1†	0.115±0.1*‡	0.802±0.23†	0.60±1.23*†‡	0.67±0.45*†‡	0.795±0.33*†	0.814±0.7†	0.877±0.9†
Juniper	1†‡	0.081±0.33*‡	0.693±0.21*†	0.842±0.32†	0.898±0.23†‡	0.870±0.72†	0.754±0.51*†	0.668±0.34*†
Birch	1†‡	0.058±0.24*‡	0.772±0.23*†	0.873±0.33†	0.838±0.46†	0.803±0.7†	0.767±0.8*†	0.718±0.21*†
Fennel	1†‡	0.184±0.8*‡	0.731±0.22*†	0.833±0.18†	0.887±0.23†	0.896±0.92†	0.928±1.92†	0.979±1.8†‡
Cedar fruit	1†‡	0.040±0.23*‡	0.713±0.21*†	0.740±0.27*†	0.721±0.23*†	0.859±0.81†	0.891±0.54†	0.961±0.37†‡
Lemon	1†‡	0.048±0.34*‡	0.745±0.22*†	0.744±0.54*†	0.770±0.21*†	0.811±0.22†	0.925±0.33†	0.968±0.22†‡
Roman chamomile	1†	0.136±0.14*‡	0.916±0.61†	0.893±1.31†	0.872±0.61†	0.833±1.8†	0.819±1.8†	0.813±0.3†
Savory	1†‡	0.058±0.32*‡	0.704±1.34*†	0.840±1.71†	0.86±0.3†	0.892±0.32†	0.913±0.9†	0.981±1.8†‡
Rosemary	1†	0.061±0.51*‡	0.889±0.63†	0.828±0.5†	0.847±0.62†	0.856±0.36†	0.876±1.23†	0.925±0.72†
Ceylon cinnamon peel	1†	0.20±0.33*‡	0.861±0.37†	0.751±0.55*†	0.84±0.9†	0.848±0.14†	0.904±0.64†	0.901±0.15†
Eucalyptus globulus	1†	0.084±0.23*‡	0.926±0.18†	0.859±0.61†	0.891±1.7†	0.954±0.4†	0.962±1.43†	0.999±0.17†
Orange sweet	1†	0.089±0.54*‡	0.870±0.21†	0.986±0.22†	0.927±0.34†	0.915±0.9†	0.911±0.5†	0.908±0.7†

Niaouly	1†	0.182±0.32*‡	0.826±0.41†	0.742±0.4*†	0.736±0.32*†	0.732±0.56*†	0.862±0.61†	0.926±0.35†
Artemisia	1†	0.042±0.54*‡	0.825±0.67†	0.861±0.21†	0.908±0.24†	0.924±0.81†	0.929±0.92†	0.926±0.54†
Cajeput	1†	0.121±0.63*‡	0.852±0.22†	0.950±0.61†	0.967±0.44†	0.944±0.45†	0.931±0.64†	0.837±0.9†
Black pepper	1†‡	0.036±0.52*‡	0.738±0.11*†	0.957±0.13†	0.904±0.72†	0.895±0.92†	0.850±0.7†	0.760±0.91*†
White thyme	1†‡	0.116±0.57*‡	0.787±0.34*†	0.825±1.23†	0.816±0.10†	0.912±0.12†	0.921±1.22†	0.905±0.82†
Marjoram	1†‡	0.064±0.5*‡	0.765±0.34*†	0.814±0.32†	0.849±1.17†	0.927±1.16†	0.932±0.14†	0.941±1.12†
Clove	1†	0.077±0.12*‡	0.803±0.45†	0.928±1.4†	0.890±0.56†	0.874±0.9†	0.827±1.5†	0.806±0.41†
Cypress	1†‡	0.101±0.31*‡	0.680±0.31*†	0.760±0.16*†	0.706±0.16*†	0.857±0.51†	0.920±1.12†	0.989±1.32†‡
Nutmeg natural	1†	0.164±0.12*‡	0.891±0.87†	0.873±0.34†	0.839±0.21†	0.826±0.74†	0.821±0.54†	0.813±0.31†
Peppermint	1†	0.052±0.2*‡	0.871±0.31†	0.788±0.18*†	0.790±0.61*†	0.830±0.48†	0.824±1.15†	0.948±1.02†
Verbena	1†	0.035±0.18*‡	0.974±0.71†	0.932±0.7†	0.926±0.71†	0.814±0.23†	0.731±1.01*†‡	0.71±0.45*†‡
Basil	1†	0.061±0.51*‡	0.901±0.1†	0.922±0.9†	0.871±0.23†	0.846±0.64†	0.814±0.6†	0.78±0.21*†‡
Palmarosa	1†‡	0.033±0.21*‡	0.795±0.33*†	0.852±0.51†	0.844±0.71†	0.840±0.54†	0.809±0.21†	0.91±0.35†
Laurel	1†	0.175±0.58*‡	0.884±0.24†	0.935±0.54†	0.904±0.6†	0.877±0.32†	0.839±0.8†	0.824±0.22†
Narural anise pure	1†	0.058±0.3*‡	0.883±1.2†	0.818±1.52†	0.826±1.14†	0.881±1.62†	0.910±0.61†	0.906±0.54†
Incense	1†	0.055±0.5*‡	0.827±0.52†	0.805±0.54†	0.816±0.43†	0.917±1.45†	0.953±0.91†	0.979±1.04†
Mentha suaveolens (Sicily)	1†	0.039±0.31*‡	0.826±1.3†	0.858±0.32†	0.867±0.44†	0.875±1.09†	0.954±1.04†	0.97±1.03†
Coridotthymus capitatus (Sicily)	1†‡	0.065±0.2*‡	0.755±0.8*†	0.821±1.6†	0.973±0.37†	0.981±1.16†	0.947±0.5†	0.987±1.05†‡
Thymus vulgaris (Sicily)	1†	0.157±0.56*‡	0.913±0.21†	0.918±0.56†	0.887±0.12†	0.933±0.12†	0.895±0.41†	0.874±0.13†
Origanum Hirtum (Sicily)	1†‡	0.081±0.23*‡	0.736±0.51*†	0.857±0.23†	0.800±0.33†	0.803±0.34†	0.852±0.31†	0.847±0.17†

[a]The values are mean ± S.D. from three independent experiments [b]NC: negative control, DNA control
[c]PC: positive control, DNA damage control [d]Quercetin 100 μg/mL, standard drug. $^{*}p < 0.05$ when compared with the negative control $^{\dagger}p < 0.05$ when compared with the positive control $^{\ddagger}p < 0.05$ when compared with the standard.

Table S6. List of dataset pretreatment paramenters settings randomly varied during ML hyperparameters optimization.

Parameter	Settings	Description
MinMaxScaling	True and False	Data were also processed using the MinMaxScaler function (scaling), which allows the original values to be scaled to values between 0 and 1. True and False indicated whether the dataset will be scaled or not.
n_level	0, 1, 2, 3, 4	This represent the number of least occurrences for EO's components to be maitained in the training set
PCA	60 %, 70%, 80 %, 90 %, 99%, 100 %	Amount of variance extracted with the PCA. 100% indicate tha the whole EOs' data matrix was used but trasformed to avoid the usage of correlate variables.

Table S7. List of the initial thresholds[a] used for the ML models' for each antioxidant evaluation.

M^{n+} [b]	[1.28, 1.316, 1.35, 1.418, 1.532, 1.608, 1.752]
DPPH• [c]	[0.63, 0.66, 0.688, 0.768, 0.84, 1.092]
LOO• [d]	[0.18, 0.19, 0.231, 0.285, 0.346, 0.486, 0.576, 0.618, 0.694, 0.73, 0.9]
ABTS•+ [e]	[0.089, 0.09, 0.097, 0.179, 0.314, 0.358, 0.494, 0.656, 0.68, 0.792, 0.87, 1.072]
OH• [f]	[0.098, 0.111, 0.16, 0.25, 0.286, 0.378, 0.504, 0.568, 0.664, 0.876]
ROO• [g]	[113.05, 121.264, 128.934, 138.086, 140.312, 141.648, 149.372, 159.59, 164.548, 170.132, 172.46, 178.124, 188.624, 194.21, 195.976, 197.46, 197.55, 204.402, 209.534, 215.9, 250.942]
OH• [h]	[80.93, 82.598, 83.76, 85.588, 88.228, 89.69, 95.402, 95.99, 99.844, 103.924, 105.16, 114.562, 130.522, 133.102, 133.566, 134.938, 136.498, 138.076, 143.608, 143.962, 144.346, 147.19, 150.16, 157.662]

[a] The threshols were obtained by varying the percentiles between 0.1 and 0.6 at 0.01 increment.Percentile = 0.1 means that 10% of data has lower values at a given threshold, therefore are defined actives obtaining a dataset with unbalanced distribution towards inactives. Percentile = 0.5 means the dataset is perfectly divided in two as the threshold is the median value. Percentile = 0.6 indicates the number of actives is slighly higher than inactives [b]Metal ion chelating capacity [c]Neutralization of DPPH radical [d]Interuption of lipid peroxidation [e]Neutralization of ABTS cation radical [f]Neutralization of hydroxyl radical [g]Protection of DNA against the damage induced by the alkoxy radical [h]Protection of DNA against the damage induced by the hydroxyl radical

Table S8. Hyperparameters used for the ML models' through random search optimization at 100 and 1000 iterations.

Algorithm	Parameters	Settings	Total Combinations
RF	class_weight	list_weight	42000
	n_estimators	from 100 to 1100, step 50	
	max_depth	from 1 to 15, step 1	
	min_samples_split	From 0.1 to 1.0, select 10 values	
	min_samples_leaf	from 0.1 to 0.5, select 5 values	
GB	n_estimators	from 10 to 210, step 10	56000
	max_depth	from 1 to 15, step 1	
	min_samples_leaf	from 0.1 to 0.5, select 5 values	
	min_samples_split	from 0.1 to 1.0, select 10 values	
	max_features	auto, sqrt, log2, None	
SVM	class_weight	list_weight	800
	C	from 0.001 to 100, select 100 values	
	kernel	linear, poly, rbf, sigmoid	
	probability	TRUE	
DT	class_weight	list_weight	
	criterion	gini, entropy	22400
	splitter	best, random	
	max_depth	from 1 to 15, step 1	
	min_samples_split	from 0.1 to 1.0, select 10 values	
	min_samples_leaf	from 0.1 to 0.5, select 5 values	
	max_features	auto, sqrt, log2, None	
KNN	n_neighbors	from 5 to 35, step 5	9600
	weights	uniform, distance	
	algorithm	auto, ball_tree, kd_tree, brute	
	leaf_size	from 5 to 55, step 5	
	metric	minkowski, euclidean, manhattan, chebyshev	
	metric_params	None	
	p	1, 2, 3, 4, 5	

Table S9. List of hyperparameters setting used for the ML models' through random search optimization performed at 10000 and 100000 iterations.

Algorithm	Parameters	Settings	Total Combinations
RF	class_weight	list_weight	210000000
	n_estimators	from 1 to 1001, step 1	
	max_depth	from 1 to 15, step 1	
	min_samples_split	From 0.1 to 1.0, select 100 values	
	min_samples_leaf	from 0.1 to 0.5, select 50 values	
GB	n_estimators	from 1 to 201, step 1	56000000
	max_depth	from 1 to 15, step 1	
	min_samples_leaf	from 0.1 to 0.5, select 50 values	
	min_samples_split	from 0.1 to 1.0, select 100 values	
	max_features	auto, sqrt, log2, None	
SVM	class_weight	list_weight	8000
	C	from 0.001 to 100, select 1000 values	
	kernel	linear, poly, rbf, sigmoid	
	probability	TRUE	
DT	class_weight	list_weight	
	criterion	gini, entropy	1680000
	splitter	best, random	
	max_depth	from 1 to 15, step 1	
	min_samples_split	from 0.1 to 1.0, select 100 values	
	min_samples_leaf	from 0.1 to 0.5, select 50 values	
	max_features	auto, sqrt, log2, None	
KNN	n_neighbors	from 5 to 35, step 1	240000
	weights	uniform, distance	
	algorithm	auto, ball_tree, kd_tree, brute	
	leaf_size	from 1 to 51, step 1	
	metric	minkowski, euclidean, manhattan, chebyshev	
	metric_params	None	
	p	1, 2, 3, 4, 5	

Final ML Models Development

The datasets were divided into training set and test set using Stratified K fold at 5 groups. Predictive ability was evaluated by the mean of MCC_{Pred} values obtained.

1. M^{n+}

1.1. 100 random iterations.

During the random search with 100 iterations, the classifiers used were: SVM, GB, KNN, RF and DT with the threshold listed in Table S7.

Table S10. Coarse best models obtained for each classifier.

# Model	Threshold	ML	Average MCC_{Pred}	Average Normalized MCC_{Pred}	MCC_{Fit}	MCC_{CV}	Average ACC_{Pred}	Average $F1_{Pred}$	Average ROC_AUC_{Pred}
**MN1**	1.35	SVM	0.44	0.72	1.00	0.16	0.88	0.43	0.65
**MN2**	1.28	GB	0.47	0.73	1.00	- 0.10	0.93	0.47	0.18
**MN3**	1.316	KNN	0.37	0.69	1.00	0.36	0.88	0.37	0.13
**MN4**	1.752	RF	0.36	0.68	0.63	0.37	0.82	0.45	0.43
**MN5**	1.35	DT	0.30	0.65	0.22	- 0.03	0.49	0.34	0.29

Table S11. Hyperparameters associated to models **MN1-MN5**. The list is reported as python dictionaries.

# Model	Hyperparameters
**MN1**	{'class_weight': None, 'C': 87.88, 'kernel': 'rbf', 'probability': True}
**MN2**	{'n_estimators': 160, 'max_depth': 3, 'min_samples_leaf': 0.2, 'min_samples_split': 0.6, 'max_features': 'log2'}
**MN3**	{'n_neighbors': 5, 'weights': 'distance', 'algorithm': 'auto', 'leaf_size': 25, 'metric': 'chebyshev', 'metric_params': None, 'p': 1}
**MN4**	{'class_weight': 'balanced', 'n_estimators': 600, 'max_depth': 11, 'min_samples_split': 0.5, 'min_samples_leaf': 0.1}
**MN5**	{'class_weight': 'balanced', 'criterion': 'gini', 'splitter': 'best', 'max_depth': 13, 'min_samples_split': 1.0, 'min_samples_leaf': 0.1, 'max_features': 'sqrt'}

1.2. 1000 random iterations.

For the intermediate random search with 1000 iterations, the ML classifiers and the thresholds were selected on the basis of best 5 models (Tables S10 and Table S11). Here the random search with 1000 iterations was run using SVM, GB, KNN, and RF and the thresholds values were reduced to 1.28, 1.316, 1.35, 1.418, 1.532, 1.608 and 1.752.

Table S12. Coarse best models obtained for each classifier.

# Model	Threshold	ML	Average MCC_{Pred}	Average Normalized MCC_{Pred}	MCC_{Fit}	MCC_{CV}	Average ACC_{Pred}	Average $F1_{Pred}$	Average ROC_AUC_{Pred}
**MN6**	1.418	SVM	0.49	0.75	0.86	0.14	0.88	0.53	0.45
**MN7**	1.752	GB	0.51	0.75	1.00	0.11	0.83	0.56	0.28
**MN8**	1.316	KNN	0.37	0.69	1.00	0.36	0.88	0.37	0.13
**MN9**	1.532	RF	0.49	0.74	1.00	0.00	0.88	0.5	0.44

Table S13. Hyperparameters associated to models **MN6-MN9**. The list is reported as python dictionaries.

# Model	Hyperparameters
**MN6**	{'class_weight': None, 'C': 64.65, 'kernel': 'rbf', 'probability': True}
**MN7**	{'n_estimators': 140, 'max_depth': 9, 'min_samples_leaf': 0.30, 'min_samples_split': 0.9, 'max_features': 'sqrt'}
**MN8**	{'n_neighbors': 5, 'weights': 'distance', 'algorithm': 'auto', 'leaf_size': 25, 'metric': 'chebyshev', 'metric_params': None, 'p': 3}
**MN9**	{'class_weight': 'balanced_subsample', 'n_estimators': 750, 'max_depth': 6, 'min_samples_split': 0.2, 'min_samples_leaf': 0.2}

1.3. 10000 random iterations.

For the random search with 10000 iterations, the ML classifiers and the thresholds were selected on the basis of best 5 models (Tables S12 and Table S13). Here the random search with 1000 iterations was run using SVM, GB, KNN, and RF and the thresholds values were reduced to 1.28, 1.316, 1.35, 1.418, 1.532, 1.608 and 1.752.

Table S14. Refined best models obtained for each classifier

# Model	Threshold	ML	Average MCC_{Pred}	Average Normalized MCC_{Pred}	MCC_{Fit}	MCC_{CV}	Average ACC_{Pred}	Average $F1_{Pred}$	Average ROC_AUC_{Pred}
**MN10**	1.418	SVM	0.49	0.75	0.86	0.14	0.88	0.53	0.65
**MN11**	1.35	GB	0.60	0.80	1.00	0.00	0.93	0.6	0.24
**MN12**	1.752	KNN	0.35	0.67	0.29	0.06	0.77	0.42	0.33
**MN13**	1.752	RF	0.62	0.81	0.56	0.08	0.87	0.63	0.19

Table S15. Hyperparameters associated to models **MN10-MN13**. The list is reported as python dictionaries.

# Model	Hyperparameters
**MN10**	{'class_weight': None, 'C': 63.66, 'kernel': 'rbf', 'probability': True}
**MN11**	{'n_estimators': 59, 'max_depth': 14, 'min_samples_leaf': 0.28, 'min_samples_split': 0.57, 'max_features': 'log2'}
**MN12**	{'n_neighbors': 5, 'weights': 'uniform', 'algorithm': 'brute', 'leaf_size': 16, 'metric': 'minkowski', 'metric_params': None, 'p': 5}
**MN13**	{'class_weight': 'balanced_subsample', 'n_estimators': 21, 'max_depth': 13, 'min_samples_split': 0.1, 'min_samples_leaf': 0.23}

1.4. 100000 random iterations.

For the random search with 10000 iterations, the ML classifiers and the thresholds were selected on the basis of models in Tables S14 and S15. Here the random search with 100000 iterations was run using SVM, GB, and RF and the thresholds values were reduced to 1.28 and 1.752.

Table S16. Refined best models obtained for each classifier

# **Model**	**Threshold**	**ML**	**Average MCC_{Pred}**	**Average Normalized MCC_{Pred}**	**MCC_{Fit}**	**MCC_{CV}**	**Average ACC_{Pred}**	**Average $F1_{Pred}$**	**Average ROC_AUC_{Pred}**
**MN14**	1.28	SVM	0.45	0.72	0.80	- 0.08	0.78	0.44	0.33
**MN15**	1.752	GB	0.61	0.81	1.00	0.20	0.88	0.6	0.27
**MN16**	1.28	RF	0.49	0.75	0.56	- 0.10	0.48	0.20	0.20

Table S17. Hyperparameters associated to models **MN14-MN16**. The list is reported as python dictionaries.

# **Model**	**Hyperparameters**
**MN14**	{'class_weight': 'balanced', 'C': 88.79, 'kernel': 'poly', 'probability': True}
**MN15**	{'n_estimators': 134, 'max_depth': 11, 'min_samples_leaf': 0.2795918367346939, 'min_samples_split': 0.55, 'max_features': 'sqrt'}
**MN16**	{'class_weight': 'balanced', 'n_estimators': 7, 'max_depth': 5, 'min_samples_split': 0.35, 'min_samples_leaf': 0.179}

2. DPPH·

2.1. 100 random iterations.

During the random search with 100 iterations, the classifiers used were: SVM, GB, KNN, RF and DT with the threshold listed in Table S7.

Table S18. Coarse best models obtained for each classifier.

# **Model**	**Threshold**	**ML**	**Average MCC_{Pred}**	**Average Normalized MCC_{Pred}**	**MCC_{Fit}**	**MCC_{CV}**	**Average ACC_{Pred}**	**Average $F1_{Pred}$**	**Average ROC_AUC_{Pred}**
**DPPH1**	0.63	SVM	0.75	0.88	1.00	0.67	0.92	0.80	0.08
**DPPH2**	0.63	GB	0.84	0.92	1.00	0.35	0.95	0.85	0.06
**DPPH3**	0.63	KNN	0.64	0.82	1.00	0.72	0.87	0.71	0.17
**DPPH4**	0.66	RF	0.64	0.82	0.72	0.44	0.87	0.71	0.14
**DPPH5**	1.092	DT	0.50	0.75	0.61	0.36	0.75	0.62	0.25

Table S19. Hyperparameters associated to models **DPPH1-DPPH5**. The list is reported as python dictionaries.

# **Model**	**Hyperparameters**
**DPPH1**	{'class_weight': 'balanced', 'C': 34.34, 'kernel': 'rbf', 'probability': True}
**DPPH2**	{'n_estimators': 200, 'max_depth': 13, 'min_samples_leaf': 0.2, 'min_samples_split': 1.0, 'max_features': 'auto'}
**DPPH3**	{'n_neighbors': 5, 'weights': 'distance', 'algorithm': 'brute', 'leaf_size': 40, 'metric': 'manhattan', 'metric_params': None, 'p': 2}
**DPPH4**	{'class_weight': 'balanced_subsample', 'n_estimators': 550, 'max_depth': 10, 'min_samples_split': 0.30, 'min_samples_leaf': 0.1}
**DPPH5**	{'class_weight': None, 'criterion': 'entropy', 'splitter': 'best', 'max_depth': 7, 'min_samples_split': 0.9, 'min_samples_leaf': 0.2, 'max_features': None}

2.2. 1000 random iterations.

For the intermediate random search with 1000 iterations, the ML classifiers and the thresholds were selected on the basis of best 5 models (Tables S10 and S11). Here the random search with 1000 iterations was run using SVM, GB, KNN, RF and DT and the thresholds values were reduced to 0.63, 0.66, 0.688, 0.768, 0.84 and 1.092.

Table S20. Coarse best models obtained for each classifier

# Model	Threshold	ML	Average MCC_{Pred}	Average Normalized MCC_{Pred}	MCC_{Fit}	MCC_{CV}	Average ACC_{Pred}	Average $F1_{Pred}$	Average ROC_AUC_{Pred}
**DPPH6**	0.63	SVM	0.81	0.90	0.90	0.79	0.93	0.82	0.13
**DPPH7**	0.63	GB	0.84	0.92	1.00	0.30	0.95	0.85	0.06
**DPPH8**	0.688	KNN	0.69	0.84	1.00	0.48	0.90	0.71	0.06
**DPPH9**	0.66	RF	0.75	0.87	0.69	0.51	0.90	0.79	0.04
**DPPH10**	0.66	DT	0.67	0.83	0.57	0.51	0.88	0.66	0.17

Table S21. Hyperparameters associated to models **DPPH6-DPPH10**. The list is reported as python dictionaries.

# Model	Hyperparameters
**DPPH6**	{'class_weight': 'balanced', 'C': 1.01, 'kernel': 'rbf', 'probability': True}
**DPPH7**	{'n_estimators': 200, 'max_depth': 14, 'min_samples_leaf': 0.2, 'min_samples_split': 0.8, 'max_features': 'auto'}
**DPPH8**	{'n_neighbors': 5, 'weights': 'distance', 'algorithm': 'auto', 'leaf_size': 50, 'metric': 'chebyshev', 'metric_params': None, 'p': 3}
**DPPH9**	{'class_weight': 'balanced', 'n_estimators': 600, 'max_depth': 14, 'min_samples_split': 0.4, 'min_samples_leaf': 0.30}
**DPPH10**	{'class_weight': None, 'criterion': 'entropy', 'splitter': 'best', 'max_depth': 11, 'min_samples_split': 0.4, 'min_samples_leaf': 0.1, 'max_features': 'auto'}

2.3. 10000 random iterations.

For the random search with 10000 iterations, the ML classifiers and the thresholds were selected on the basis of best 5 models (Tables S12 and S13). Here the random search with 1000 iterations was run using SVM, GB and DT and the thresholds values were reduced to 0.63, 0.66 and 1.092.

Table S22. Refined best models obtained for each classifier.

# Model	Threshold	ML	Average MCC_{Pred}	Average Normalized MCC_{Pred}	MCC_{Fit}	MCC_{CV}	Average ACC_{Pred}	Average $F1_{Pred}$	Average ROC_AUC_{Pred}
**DPPH11**	0.63	SVM	0.81	0.90	0.90	0.79	0.93	0.82	0.13
**DPPH12**	0.66	GB	0.86	0.93	1.00	0.41	0.95	0.88	0.04
**DPPH13**	0.66	DT	0.71	0.85	0.72	0.63	0.82	0.76	0.14

Table S23. Hyperparameters associated to models **DPPH11-DPPH13**. The list is reported as python dictionaries.

# Model	Hyperparameters
**DPPH11**	{'class_weight': 'balanced', 'C': 0.90, 'kernel': 'rbf', 'probability': True}
**DPPH12**	{'n_estimators': 116, 'max_depth': 7, 'min_samples_leaf': 0.31, 'min_samples_split': 0.39, 'max_features': 'auto'}
**DPPH13**	{'class_weight': 'balanced', 'criterion': 'gini', 'splitter': 'best', 'max_depth': 3, 'min_samples_split': 0.36, 'min_samples_leaf': 0.12, 'max_features': 'sqrt'}

2.4. 100000 random iterations.

For the random search with 10000 iterations, the ML classifiers and the thresholds were selected on the basis of models in Tables S14 and S15. Here the random search with 100000 iterations was run using SVM, GB and DT and the thresholds values were reduced to 0.63, 0.66 and 1.092.

Table S24. Refined best models obtained for each classifier.

# Model	Threshold	ML	Average MCC_{Pred}	Average Normalized MCC_{Pred}	MCC_{Fit}	MCC_{CV}	Average ACC_{Pred}	Average $F1_{Pred}$	Average ROC_AUC_{Pred}
**DPPH14**	0.63	SVM	0.81	0.90	0.90	0.79	0.93	0.82	0.14
**DPPH15**	0.66	GB	0.91	0.95	1.00	0.27	0.97	0.92	0.05
**DPPH16**	0.66	DT	0.76	0.88	0.86	0.18	0.92	0.76	0.11

Table S25. Hyperparameters associated to models **DPPH14-DPPH16**. The list is reported as python dictionaries.

# Model	Hyperparameters
**DPPH14**	{'class_weight': 'balanced', 'C': 0.70, 'kernel': 'rbf', 'probability': True}
**DPPH15**	{'n_estimators': 114, 'max_depth': 10, 'min_samples_leaf': 0.32, 'min_samples_split': 0.31, 'max_features': 'log2'}
**DPPH16**	{'class_weight': None, 'criterion': 'entropy', 'splitter': 'best', 'max_depth': 12, 'min_samples_split': 0.1, 'min_samples_leaf': 0.13, 'max_features': 'log2'}

3. LOO·

3.1. 100 random iterations.

During the random search with 100 iterations, the classifiers used were: SVM, GB, KNN, RF and DT with the threshold listed in Table S7.

Table S26. Coarse best models obtained for each classifier

# Model	Threshold	ML	Average MCC_{Pred}	Average Normalized MCC_{Pred}	MCC_{Fit}	MCC_{CV}	Average ACC_{Pred}	Average $F1_{Pred}$	Average ROC_AUC_{Pred}
**LOO1**	0.19	SVM	0.95	0.98	0.93	0.93	0.98	0.96	0.02
**LOO2**	0.19	GB	0.93	0.97	1.00	0.28	0.98	0.93	0.01
**LOO3**	0.346	KNN	0.76	0.88	1.00	0.70	0.93	0.76	0.14
**LOO4**	0.346	RF	0.65	0.83	1.00.	0.00	0.90	0.67	0.08
**LOO5**	0.73	DT	0.61	0.80	0.64	0.38	0.82	0.68	0.19

Table S27. Hyperparameters associated to models **LOO1-LOO5**. The list is reported as python dictionaries.

# Model	Hyperparameters
**LOO1**	{'class_weight': None, 'C': 60.61, 'kernel': 'sigmoid', 'probability': True}
**LOO2**	{'n_estimators': 120, 'max_depth': 6, 'min_samples_leaf': 0.1, 'min_samples_split': 1.0, 'max_features': 'log2'}
**LOO3**	{'n_neighbors': 5, 'weights': 'distance', 'algorithm': 'auto', 'leaf_size': 50, 'metric': 'manhattan', 'metric_params': None, 'p': 4}
**LOO4**	{'class_weight': 'balanced', 'n_estimators': 550, 'max_depth': 10, 'min_samples_split': 0.30, 'min_samples_leaf': 0.1}
**LOO5**	{'class_weight': None, 'criterion': 'entropy', 'splitter': 'best', 'max_depth': 7, 'min_samples_split': 0.9, 'min_samples_leaf': 0.2, 'max_features': None}

3.2. 1000 random iterations.

For the intermediate random search with 1000 iterations, the ML classifiers and the thresholds were selected on the basis of best 5 models (Tables S10 and S11). Here the random search with 1000 iterations was run using SVM, GB, KNN, RF and DT and the thresholds values were reduced to 0.18, 0.19, 0.231, 0.285, 0.346, 0.576, 0.618, 0.694, 0.73 and 0.9.

Table S28. Coarse best models obtained for each classifier.

# **Model**	**Threshold**	**ML**	**Average MCC$_{Pred}$**	**Average Normalized MCC$_{Pred}$**	**MCC$_{Fit}$**	**MCC$_{CV}$**	**Average ACC$_{Pred}$**	**Average F1$_{Pred}$**	**Average ROC_AUC$_{Pred}$**
**LOO6**	0.19	SVM	0.95	0.98	0.93	0.88	0.98	0.96	0.02
**LOO7**	0.19	GB	1.00	1.00	1.00	0.36	1.0	1.0	0.0
**LOO8**	0.73	KNN	0.77	0.88	1.00	0.74	0.92	0.81	0.15
**LOO9**	0.19	RF	0.78	0.89	0.65.	0.60	0.9	0.79	0.06
**LOO10**	0.19	DT	0.80	0.90	0.71	0.45	0.9	0.8	0.07

Table S29. Hyperparameters associated to models **LOO6-LOO10**. The list is reported as python dictionaries.

# **Model**	**Hyperparameters**
**LOO6**	{'class_weight': 'balanced', 'C': 97.98, 'kernel': 'sigmoid', 'probability': True}
**LOO7**	{'n_estimators': 120, 'max_depth': 9, 'min_samples_leaf': 0.1, 'min_samples_split': 0.4, 'max_features': 'log2'}
**LOO8**	{'n_neighbors': 5, 'weights': 'distance', 'algorithm': 'auto', 'leaf_size': 50, 'metric': 'chebyshev', 'metric_params': None, 'p': 3}
**LOO9**	{'class_weight': 'balanced_subsample', 'n_estimators': 850, 'max_depth': 13, 'min_samples_split': 0.2, 'min_samples_leaf': 0.2}
**LOO10**	{'class_weight': 'balanced', 'criterion': 'gini', 'splitter': 'best', 'max_depth': 3, 'min_samples_split': 0.30, 'min_samples_leaf': 0.1, 'max_features': None}

3.3. 10000 random iterations.

For the random search with 10000 iterations, the ML classifiers and the thresholds were selected on the basis of best 5 models (Tables S12 and S13). Here the random search with 1000 iterations was run using SVM, GB, RF and DT and the thresholds values were reduced to 0.18, 0.19, 0.231, 0.285, 0.346, 0.576, 0.694 and 0.9.

Table S30. Refined best models obtained for each classifier

# **Model**	**Threshold**	**ML**	**Average MCC$_{Pred}$**	**Average Normalized MCC$_{Pred}$**	**MCC$_{Fit}$**	**MCC$_{CV}$**	**Average ACC$_{Pred}$**	**Average F1$_{Pred}$**	**Average ROC_AUC$_{Pred}$**
**LOO11**	0.19	SVM	0.95	0.98	0.93	0.80	0.98	0.96	0.02
**LOO12**	0.19	GB	1.0	1.0	1.0	0.75	1.0	1.0	0.0
**LOO13**	0.19	RF	0.80	0.90	0.78.	0.36	0.95	0.8	0.04
**LOO14**	0.19	DT	0.83	0.91	0.71	0.45	0.92	0.83	0.05

Table S31. Hyperparameters associated to models **LOO10-LOO14**. The list is reported as python dictionaries.

# **Model**	**Hyperparameters**
**LOO11**	{'class_weight': None, 'C': 75.78, 'kernel': 'sigmoid', 'probability': True}
**LOO12**	{'n_estimators': 190, 'max_depth': 3, 'min_samples_leaf': 0.26, 'min_samples_split': 0.22, 'max_features': 'sqrt'}
**LOO13**	{'class_weight': 'balanced', 'n_estimators': 81, 'max_depth': 9, 'min_samples_split': 0.19, 'min_samples_leaf': 0.21}
**LOO14**	{'class_weight': 'balanced', 'criterion': 'gini', 'splitter': 'best', 'max_depth': 7, 'min_samples_split': 0.2, 'min_samples_leaf': 0.11, 'max_features': None}

3.4. 100000 random iterations

For the random search with 10000 iterations, the ML classifiers and the thresholds were selected on the basis of models in Tables S14 and S15. Here the random search with 100000 iterations was run using SVM, GB and RF and the thresholds values were reduced to 0.18, 0.19, 0.231 and 0.576.

Table S32. Refined best models obtained for each classifier

# Model	Threshold	ML	Average MCC_{Pred}	Average Normalized MCC_{Pred}	MCC_{Fit}	MCC_{CV}	Average ACC_{Pred}	Average $F1_{Pred}$	Average ROC_AUC_{Pred}
**LOO15**	0.19	SVM	0.95	0.98	0.93	0.82	0.98	0.96	0.02
**LOO16**	0.19	GB	1.0	1.0	1.0	0.69	1.0	1.0	0.0
**LOO17**	0.19	RF	0.82	0.91	0.93.	0.20	0.95	0.83	0.01

Table S33. Hyperparameters associated to models **LOO15-LOO17**. The list is reported as python dictionaries.

# Model	Hyperparameters
**LOO15**	{'class_weight': None, 'C': 35.64, 'kernel': 'sigmoid', 'probability': True}
**LOO16**	{'n_estimators': 111, 'max_depth': 12, 'min_samples_leaf': 0.13, 'min_samples_split': 0.12, 'max_features': None}
**LOO17**	{'class_weight': 'balanced_subsample', 'n_estimators': 926, 'max_depth': 8, 'min_samples_split': 0.72, 'min_samples_leaf': 0.22}

4. ABTS$^{+•}$

4.1. 100 random iterations.

During the random search with 100 iterations, the classifiers used were: SVM, GB, KNN, RF and DT with the threshold listed in Table S7.

Table S34. Coarse best models obtained for each classifier

# Model	Threshold	ML	Average MCC_{Pred}	Average Normalized MCC_{Pred}	MCC_{Fit}	MCC_{CV}	Average ACC_{Pred}	Average $F1_{Pred}$	Average ROC_AUC_{Pred}
**ABTS1**	0.179	SVM	0.94	0.97	1.00	0.64	0.98	0.93	0.00
**ABTS2**	0.179	GB	0.89	0.94	1.00	0.28	0.97	0.89	0.02
**ABTS3**	0.097	KNN	0.87	0.93	1.00	0.59	0.97	0.87	0.03
**ABTS4**	0.358	RF	0.71	0.85	0.86	0.48	0.92	0.70	0.03
**ABTS5**	1.072	DT	0.61	0.81	0.61	0.51	0.85	0.61	0.27

Table S35. Hyperparameters associated to models **ABTS1-ABTS5** The list is reported as python dictionaries.

# Model	Hyperparameters
**ABTS1**	{'class_weight': 'balanced', 'C': 34.34, 'kernel': 'rbf', 'probability': True}
**ABTS2**	{'n_estimators': 150, 'max_depth': 12, 'min_samples_leaf': 0.30, 'min_samples_split': 0.2, 'max_features': 'sqrt'}
**ABTS3**	{'n_neighbors': 10, 'weights': 'distance', 'algorithm': 'brute', 'leaf_size': 20, 'metric': 'manhattan', 'metric_params': None, 'p': 1}
**ABTS4**	{'class_weight': 'balanced', 'n_estimators': 450, 'max_depth': 11, 'min_samples_split': 0.5, 'min_samples_leaf': 0.1}
**ABTS5**	{'class_weight': None, 'criterion': 'entropy', 'splitter': 'best', 'max_depth': 7, 'min_samples_split': 0.2, 'min_samples_leaf': 0.1, 'max_features': None}

4.2. 1000 random iterations.
For the intermediate random search with 1000 iterations, the ML classifiers and the thresholds were selected on the basis of best 5 models (Tables S10 and S11). Here the random search with 1000 iterations was run using : SVM, GB, KNN, RF and DT and the thresholds values were reduced to 0.089, 0.09, 0.097, 0.179, 0.314, 0.358, 0.494, 0.656, 0.68, 0.792 and 1.072.

Table S36. Coarse best models obtained for each classifier

# **Model**	**Threshold**	**ML**	**Average MCC_{Pred}**	**Average Normalized MCC_{Pred}**	**MCC_{Fit}**	**MCC_{CV}**	**Average ACC_{Pred}**	**Average $F1_{Pred}$**	**Average ROC_AUC_{Pred}**
**ABTS6**	0.179	SVM	0.95	0.98	1.00	0.72	0.98	0.96	0.00
**ABTS7**	0.179	GB	0.93	0.96	1.00	0.00	0.98	0.93	0.01
**ABTS8**	0.097	KNN	0.87	0.93	1.00	0.68	0.97	0.87	0.03
**ABTS9**	0.097	RF	0.83	0.91	0.79	0.62	0.95	0.83	0.05
**ABTS10**	0.179	DT	0.80	0.90	0.67	0.48	0.92	0.81	0.05

Table S37. Hyperparameters associated to models **ABTS6-ABTS10**. The list is reported as python dictionaries.

# **Model**	**Hyperparameters**
**ABTS6**	{'class_weight': None, 'C': 75.76, 'kernel': 'sigmoid', 'probability': True}
**ABTS7**	{'n_estimators': 120, 'max_depth': 14, 'min_samples_leaf': 0.1, 'min_samples_split': 0.8, 'max_features': 'log2'}
**ABTS8**	{'n_neighbors': 10, 'weights': 'distance', 'algorithm': 'brute', 'leaf_size': 50, 'metric': 'minkowski', 'metric_params': None, 'p': 1}
**ABTS9**	{'class_weight': 'balanced', 'n_estimators': 700, 'max_depth': 14, 'min_samples_split': 0.5, 'min_samples_leaf': 0.2}
**ABTS10**	{'class_weight': 'balanced', 'criterion': 'gini', 'splitter': 'best', 'max_depth': 4, 'min_samples_split': 0.30, 'min_samples_leaf': 0.1, 'max_features': 'sqrt'}

4.3. 10000 random iterations.
For the random search with 10000 iterations, the ML classifiers and the thresholds were selected on the basis of best 5 models (Tables S12 and S13). Here the random search with 1000 iterations was run using SVM, GB, KNN and DT and the thresholds values were reduced to 0.09, 0.097 and 0.179.

Table S38. Refined best models obtained for each classifier.

# **Model**	**Threshold**	**ML**	**Average MCC_{Pred}**	**Average Normalized MCC_{Pred}**	**MCC_{Fit}**	**MCC_{CV}**	**Average ACC_{Pred}**	**Average $F1_{Pred}$**	**Average ROC_AUC_{Pred}**
**ABTS11**	0.179	SVM	0.95	0.98	1.00	0.64	0.98	0.96	0.00
**ABTS12**	0.097	GB	1.0	1.0	1.00	0.71	1.0	1.0	0.0
**ABTS13**	0.097	KNN	0.87	0.93	1.00	0.52	0.97	0.87	0.02
**ABTS14**	0.097	RF	0.89	0.94	0.70	0.62	0.97	0.89	0.02

Table S39. Hyperparameters associated to models **ABTS11-ABTS14**. The list is reported as python dictionaries.

# **Model**	**Hyperparameters**
**ABTS11**	{'class_weight': None, 'C': 35.14, 'kernel': 'sigmoid', 'probability': True}
**ABTS12**	{'n_estimators': 192, 'max_depth': 3, 'min_samples_leaf': 0.21, 'min_samples_split': 0.28, 'max_features': 'log2'}
**ABTS13**	{'n_neighbors': 8, 'weights': 'distance', 'algorithm': 'kd_tree', 'leaf_size': 11, 'metric': 'minkowski', 'metric_params': None, 'p': 5}
**ABTS14**	{'class_weight': 'balanced', 'n_estimators': 571, 'max_depth': 6, 'min_samples_split': 0.62, 'min_samples_leaf': 0.22}

4.4. 100000 random iterations.

For the random search with 10000 iterations, the ML classifiers and the thresholds were selected on the basis of models in Tables S14 and S15. Here the random search with 100000 iterations was run using SVM, GB and KNN and the thresholds values were reduced to 0.09, 0.097 and 0.179.

Table S40. Refined best models obtained for each classifier.

# Model	Threshold	ML	Average MCC_{Pred}	Average Normalized MCC_{Pred}	MCC_{Fit}	MCC_{CV}	Average ACC_{Pred}	Average $F1_{Pred}$	Average ROC_AUC_{Pred}
**ABTS15**	0.179	SVM	0.95	0.98	1.00	0.64	0.98	0.96	0.00
**ABTS16**	0.097	GB	1.00	1.00	1.00	0.85	1.0	1.00	0.00
**ABTS17**	0.097	KNN	0.87	0.93	1.00	0.59	0.97	0.87	0.03

Table S41. Hyperparameters associated to models **ABTS15-ABTS17**. The list is reported as python dictionaries.

# Model	Hyperparameters
**ABTS15**	{'class_weight': None, 'C': 36.34, 'kernel': 'sigmoid', 'probability': True}
**ABTS16**	{'n_estimators': 154, 'max_depth': 3, 'min_samples_leaf': 0.13, 'min_samples_split': 0.32, 'max_features': 'auto'}
**ABTS17**	{'n_neighbors': 8, 'weights': 'distance', 'algorithm': 'kd_tree', 'leaf_size': 22, 'metric': 'manhattan', 'metric_params': None, 'p': 3}

5. OH$^{\cdot}$

5.1. 100 random iterations

During the random search with 100 iterations, the classifiers used were: SVM, GB, KNN, RF and DT with the threshold listed in Table S7.

Table S42. Coarse best models obtained for each classifier.

# Model	Threshold	ML	Average MCC_{Pred}	Average Normalized MCC_{Pred}	MCC_{Fit}	MCC_{CV}	Average ACC_{Pred}	Average $F1_{Pred}$	Average ROC_AUC_{Pred}
**OH1**	0.16	SVM	0.89	0.94	0.87	0.74	0.97	0.89	0.01
**OH2**	0.286	GB	0.82	0.91	1.00	0.37	0.95	0.83	0.08
**OH3**	0.16	KNN	0.70	0.85	1.00	0.61	0.92	0.73	0.08
**OH4**	0.286	RF	0.78	0.89	1.00	0.15	0.93	0.79	0.08
**OH5**	0.16	DT	0.66	0.83	0.48	0.39	0.83	0.68	0.10

Table S43. Hyperparameters associated to models **OH1-OH5**. The list is reported as python dictionaries.

# Model	Hyperparameters
**OH1**	{'class_weight': 'balanced', 'C': 17.17, 'kernel': 'sigmoid', 'probability': True}
**OH2**	{'n_estimators': 120, 'max_depth': 9, 'min_samples_leaf': 0.2, 'min_samples_split': 0.1, 'max_features': 'sqrt'}
**OH3**	{'n_neighbors': 5, 'weights': 'distance', 'algorithm': 'kd_tree', 'leaf_size': 30, 'metric': 'minkowski', 'metric_params': None, 'p': 5}
**OH4**	{'class_weight': 'balanced_subsample', 'n_estimators': 1050, 'max_depth': 10, 'min_samples_split': 0.4, 'min_samples_leaf': 0.1}
**OH5**	{'class_weight': 'balanced', 'criterion': 'gini', 'splitter': 'best', 'max_depth': 8, 'min_samples_split': 0.70, 'min_samples_leaf': 0.4, 'max_features': None}

5.2. 1000 random iterations.

For the intermediate random search with 1000 iterations, the ML classifiers and the thresholds were selected on the basis of best 5 models (Tables S10 and S11). Here the random search with 1000 iterations was run using SVM, GB, KNN, RF and DT and the thresholds values were reduced to 0.098, 0.111, 0.16, 0.25, 0.286, 0.378, 0.504, 0.568, 0.664 and 0.876.

Table S44. Coarse best models obtained for each classifier.

# Model	Threshold	ML	Average MCC_{Pred}	Average Normalized MCC_{Pred}	MCC_{Fit}	MCC_{CV}	Average ACC_{Pred}	Average $F1_{Pred}$	Average ROC_AUC_{Pred}
**OH6**	0.16	SVM	0.89	0.94	0.89	0.74	0.97	0.89	0.01
**OH7**	0.111	GB	0.93	0.97	1.00	0.40	0.98	0.93	0.09
**OH8**	0.25	KNN	0.75	0.88	1.00	0.60	0.95	0.76	0.1
**OH9**	0.111	RF	0.87	0.93	0.92	0.57	0.97	0.87	0.12
**OH10**	0.286	DT	0.89	0.94	0.72	0.54	0.95	0.90	0.03

Table S45. Hyperparameters associated to models **OH6-OH10**. The list is reported as python dictionaries.

# Model	Hyperparameters
**OH6**	{'class_weight': 'balanced', 'C': 18.18, 'kernel': 'sigmoid', 'probability': True}
**OH7**	{'n_estimators': 120, 'max_depth': 6, 'min_samples_leaf': 0.1, 'min_samples_split': 0.8, 'max_features': 'sqrt'}
**OH8**	{'n_neighbors': 5, 'weights': 'distance', 'algorithm': 'ball_tree', 'leaf_size': 25, 'metric': 'minkowski', 'metric_params': None, 'p': 2}
**OH9**	{'class_weight': 'balanced_subsample', 'n_estimators': 1000, 'max_depth': 5, 'min_samples_split': 0.4, 'min_samples_leaf': 0.1}
**OH10**	{'class_weight': 'balanced', 'criterion': 'gini', 'splitter': 'best', 'max_depth': 4, 'min_samples_split': 0.1, 'min_samples_leaf': 0.1, 'max_features': 'sqrt'}

5.3. 10000 random iterations.

For the random search with 10000 iterations, the ML classifiers and the thresholds were selected on the basis of best 5 models (Tables S12 and S13). Here the random search with 1000 iterations was run using SVM, GB, RF and DT and the thresholds values were reduced to 0.098, 0.111, 0.286 and 0.568.

Table S46. Refined best models obtained for each classifier.

# Model	Threshold	ML	Average MCC_{Pred}	Average Normalized MCC_{Pred}	MCC_{Fit}	MCC_{CV}	Average ACC_{Pred}	Average $F1_{Pred}$	Average ROC_AUC_{Pred}
**OH11**	0.286	SVM	0.89	0.94	1.00	0.71	0.97	0.89	0.1
**OH12**	0.111	GB	0.93	0.97	1.00	0.73	0.98	0.93	0.09
**OH13**	0.111	RF	0.87	0.93	0.92	0.57	0.97	0.87	0.13
**OH14**	0.111	DT	0.79	0.90	0.73	0.57	0.90	0.79	0.09

Table S47. Hyperparameters associated to models **OH11-OH14**. The list is reported as python dictionaries.

# Model	Hyperparameters
**OH11**	{'class_weight': None, 'C': 78.18, 'kernel': 'linear', 'probability': True}
**OH12**	{'n_estimators': 111, 'max_depth': 7, 'min_samples_leaf': 0.12, 'min_samples_split': 0.27, 'max_features': 'log2'}
**OH13**	{'class_weight': 'balanced_subsample', 'n_estimators': 921, 'max_depth': 14, 'min_samples_split': 0.58, 'min_samples_leaf': 0.12}
**OH14**	{'class_weight': 'balanced', 'criterion': 'gini', 'splitter': 'best', 'max_depth': 7, 'min_samples_split': 0.2, 'min_samples_leaf': 0.11, 'max_features': None}

5.4. 100000 random iterations

For the random search with 10000 iterations, the ML classifiers and the thresholds were selected on the basis of models in Tables S14 and S15. Here the random search with 100000 iterations was run using SVM, GB, and RF and the thresholds values were reduced to 1.28 and 1.752.

Table S48. Refined best models obtained for each classifier

# **Model**	**Threshold**	**ML**	**Average MCC_{Pred}**	**Average Normalized MCC_{Pred}**	**MCC_{Fit}**	**MCC_{CV}**	**Average ACC_{Pred}**	**Average $F1_{Pred}$**	**Average ROC_AUC_{Pred}**
**OH15**	0.111	GB	0.93	0.97	1.00	0.43	0.98	0.93	0.08
**OH16**	0.111	RF	0.89	0.94	0.72	0.64	0.97	0.89	0.07

Table S49. Hyperparameters associated to models **OH15-OH16**. The list is reported as python dictionaries.

# **Model**	**Hyperparameters**
**OH15**	{'n_estimators': 109, 'max_depth': 14, 'min_samples_leaf': 0.12, 'min_samples_split': 0.92, 'max_features': 'sqrt'}
**OH16**	{'class_weight': 'balanced', 'n_estimators': 45, 'max_depth': 5, 'min_samples_split': 0.27, 'min_samples_leaf': 0.1}

6. ROO-RBD_{50}s

6.1. 100 random iterations.

During the random search with 100 iterations, the classifiers used were: SVM, GB, KNN, RF and DT with the threshold listed in Table S7.

Table S50. Coarse best models obtained for each classifier.

# **Model**	**Threshold**	**ML**	**Average MCC_{Pred}**	**Average Normalized MCC_{Pred}**	**MCC_{Fit}**	**MCC_{CV}**	**Average ACC_{Pred}**	**Average $F1_{Pred}$**	**Average ROC_AUC_{Pred}**
**ROO1**	164.548	SVM	0.58	0.79	1.00	0.16	0.82	0.67	0.25
**ROO2**	149.372	GB	0.48	0.74	1.00	0.24	0.78	0.63	0.26
**ROO3**	204.402	KNN	0.41	0.70	1.00	0.28	0.69	0.62	0.29
**ROO4**	172.46	RF	0.44	0.72	0.43	0.10	0.75	0.55	0.34
**ROO5**	250.942	DT	0.42	0.71	0.48	0.18	0.70	0.67	0.30

Table S51. Hyperparameters associated to models **ROO1-ROO5**. The list is reported as python dictionaries.

# **Model**	**Hyperparameters**
**ROO1**	{'class_weight': None, 'C': 95.96, 'kernel': 'linear', 'probability': True}
**ROO2**	{'n_estimators': 140, 'max_depth': 10, 'min_samples_leaf': 0.30, 'min_samples_split': 0.1, 'max_features': 'auto'}
**ROO3**	{'n_neighbors': 5, 'weights': 'distance', 'algorithm': 'brute', 'leaf_size': 25, 'metric': 'euclidean', 'metric_params': None, 'p': 2}
**ROO4**	{'class_weight': 'balanced', 'n_estimators': 450, 'max_depth': 4, 'min_samples_split': 0.30, 'min_samples_leaf': 0.30}
**ROO5**	{'class_weight': None, 'criterion': 'gini', 'splitter': 'best', 'max_depth': 13, 'min_samples_split': 0.1, 'min_samples_leaf': 0.4, 'max_features': None}

6.2. 1000 random iterations.

For the intermediate random search with 1000 iterations, the ML classifiers and the thresholds were selected on the basis of best 5 models (Tables S10 and S11). Here the random search with 1000 iterations was run using SVM, GB, KNN and RF and the thresholds values were reduced to 113.05, 121.264, 128.934, 138.086, 140.312, 149.372, 159.59, 164.548, 197.46, 197.55, 204.402, 209.534, 215.9 and 250.942.

Table S52. Coarse best models obtained for each classifier.

# **Model**	**Threshold**	**ML**	**Average MCC_{Pred}**	**Average Normalized MCC_{Pred}**	**MCC_{Fit}**	**MCC_{CV}**	**Average ACC_{Pred}**	**Average $F1_{Pred}$**	**Average ROC_AUC_{Pred}**
**ROO6**	164.548	SVM	0.58	0.79	1.00	0.16	0.82	0.67	0.25
**ROO7**	204.402	GB	0.49	0.75	1.00	0.31	0.74	0.74	0.26
**ROO8**	204.402	KNN	0.41	0.70	1.00	0.28	0.69	0.62	0.29
**ROO9**	197.55	RF	0.53	0.77	0.83	0.11	0.77	0.66	0.23

Table S53. Hyperparameters associated to models **ROO6-ROO9**. The list is reported as python dictionaries.

# **Model**	**Hyperparameters**
**ROO6**	{'class_weight': None, 'C': 96.97, 'kernel': 'linear', 'probability': True}
**ROO7**	{'n_estimators': 100, 'max_depth': 10, 'min_samples_leaf': 0.2, 'min_samples_split': 0.30, 'max_features': 'sqrt'}
**ROO8**	{'n_neighbors': 5, 'weights': 'distance', 'algorithm': 'kd_tree', 'leaf_size': 50, 'metric': 'euclidean', 'metric_params': None, 'p': 5}
**ROO9**	{'class_weight': 'balanced_subsample', 'n_estimators': 150, 'max_depth': 4, 'min_samples_split': 0.6, 'min_samples_leaf': 0.1}

6.3. 10000 random iterations.

For the random search with 10000 iterations, the ML classifiers and the thresholds were selected on the basis of best 5 models (Tables S12 and S13). Here the random search with 1000 iterations was run using SVM, GB, KNN and RF and the thresholds values were reduced to 113.05, 128.934, 164.548, 197.55, 209.534 and 215.9.

Table S54. Refined best models obtained for each classifier

# **Model**	**Threshold**	**ML**	**Average MCC_{Pred}**	**Average Normalized MCC_{Pred}**	**MCC_{Fit}**	**MCC_{CV}**	**Average ACC_{Pred}**	**Average $F1_{Pred}$**	**Average ROC_AUC_{Pred}**
**ROO10**	164.548	SVM	0.58	0.79	1.00	0.16	0.82	0.67	0.25
**ROO11**	197.55	GB	0.54	0.77	0.97	0.11	0.75	0.70	0.17
**ROO12**	209.534	KNN	0.40	0.70	1.00	0.15	0.69	0.66	0.38
**ROO13**	215.9	RF	0.52	0.76	0.28	0.05	0.75	0.76	0.23

Table S55. Hyperparameters associated to models **ROO10-ROO13**. The list is reported as python dictionaries.

# **Model**	**Hyperparameters**
**ROO10**	{'class_weight': None, 'C': 96.78, 'kernel': 'linear', 'probability': True}
**ROO11**	{'n_estimators': 135, 'max_depth': 1, 'min_samples_leaf': 0.15, 'min_samples_split': 0.83, 'max_features': 'auto'}
**ROO12**	{'n_neighbors': 8, 'weights': 'distance', 'algorithm': 'brute', 'leaf_size': 37, 'metric': 'chebyshev', 'metric_params': None, 'p': 2}
**ROO13**	{'class_weight': 'balanced', 'n_estimators': 51, 'max_depth': 10, 'min_samples_split': 0.68, 'min_samples_leaf': 0.21}

6.4. 100000 random iterations.

For the random search with 10000 iterations, the ML classifiers and the thresholds were selected on the basis of models in Tables S14 and S15. Here the random search with 100000 iterations was run using SVM, GB and RF and the thresholds values were reduced to 113.05, 164.548 and 215.9.

Table S56. Refined best models obtained for each classifier.

# Model	Threshold	ML	Average MCC_{Pred}	Average Normalized MCC_{Pred}	MCC_{Fit}	MCC_{CV}	Average ACC_{Pred}	Average $F1_{Pred}$	Average ROC_AUC_{Pred}
**ROO14**	164.548	SVM	0.58	0.79	1.00	0.16	0.82	0.67	0.33
**ROO15**	164.548	GB	0.47	0.73	0.75	0.37	0.77	0.54	0.45
**ROO16**	215.9	RF	0.58	0.79	0.84	0.11	0.77	0.75	0.28

Table S57. Hyperparameters associated to models **ROO14-ROO16**. The list is reported as python dictionaries.

# Model	Hyperparameters
**ROO14**	{'class_weight': None, 'C': 96.70, 'kernel': 'linear', 'probability': True}
**ROO15**	{'n_estimators': 48, 'max_depth': 1, 'min_samples_leaf': 0.40, 'min_samples_split': 0.46, 'max_features': 'sqrt'}
**ROO16**	{'class_weight': 'balanced', 'n_estimators': 141, 'max_depth': 2, 'min_samples_split': 0.15, 'min_samples_leaf': 0.16}

7. $OH\text{-}RBD_{50}$

7.1. 100 random iterations.

During the random search with 100 iterations, the classifiers used were: SVM, GB, KNN, RF and DT with the threshold listed in Table S7.

Table S58. Coarse best models obtained for each classifier

# Model	Threshold	ML	Average MCC_{Pred}	Average Normalized MCC_{Pred}	MCC_{Fit}	MCC_{CV}	Average ACC_{Pred}	Average $F1_{Pred}$	Average ROC_AUC_{Pred}
**OH-RBD1**	103.924	SVM	0.36	0.68	0.85	- 0.14	0.69	0.57	0.70
**OH-RBD2**	130.522	GB	0.42	0.71	0.96	- 0.15	0.73	0.58	0.32
**OH-RBD3**	144.346	KNN	0.54	0.77	1.00	0.01	0.77	0.78	0.28
**OH-RBD4**	144.346	RF	0.28	0.64	0.77	0.08	0.64	0.64	0.31
**OH-RBD5**	114.562	DT	0.31	0.65	0.36	- 0.10	0.69	0.53	0.35

Table S59. Hyperparameters associated to models **OH-RBD1-OH-RBD5**. The list is reported as python dictionaries.

# Model	Hyperparameters
**OH-RBD1**	{'class_weight': None, 'C': 81.81836363636364, 'kernel': 'sigmoid', 'probability': True}
**OH-RBD2**	{'n_estimators': 140, 'max_depth': 1, 'min_samples_leaf': 0.1, 'min_samples_split': 0.6, 'max_features': None}
**OH-RBD3**	{'n_neighbors': 20, 'weights': 'distance', 'algorithm': 'kd_tree', 'leaf_size': 10, 'metric': 'chebyshev', 'metric_params': None, 'p': 3}
**OH-RBD4**	{'class_weight': 'balanced', 'n_estimators': 950, 'max_depth': 11, 'min_samples_split': 0.4, 'min_samples_leaf': 0.1}
**OH-RBD5**	{'class_weight': None, 'criterion': 'entropy', 'splitter': 'best', 'max_depth': 7, 'min_samples_split': 0.9, 'min_samples_leaf': 0.2, 'max_features': None}

7.2. 1000 random iterations.

For the intermediate random search with 1000 iterations, the ML classifiers and the thresholds were selected on the basis of best 5 models (Tables S10 and S11). Here the random search with 1000 iterations was run using SVM, GB, KNN, RF and DT and the thresholds values were reduced to 80.93, 82.598, 83.76, 88.228, 89.69, 103.924, 130.522, 134.938, 138.076, 144.346, 150.16 and 157.662.

Table S60. Coarse best models obtained for each classifier

# Model	Threshold	ML	Average MCC_{Pred}	Average Normalized MCC_{Pred}	MCC_{Fit}	MCC_{CV}	Average ACC_{Pred}	Average $F1_{Pred}$	Average ROC_AUC_{Pred}
**OH-RBD6**	103.924	SVM	0.37	0.69	0.52	- 0.13	0.69	0.56	0.69
**OH-RBD7**	144.346	GB	0.45	0.73	1.00	0.01	0.73	0.72	0.72
**OH-RBD8**	144.346	KNN	0.54	0.77	1.00	0.01	0.77	0.78	0.28
**OH-RBD9**	144.346	RF	0.43	0.71	0.67	- 0.05	0.70	0.69	0.29
**OH-RBD10**	80.93	DT	0.43	0.72	0.31	- 0.09	0.77	0.55	0.30

Table S61. Hyperparameters associated to models **OH-RBD6-OH-RBD10**. The list is reported as python dictionaries.

# Model	Hyperparameters
**OH-RBD6**	{'class_weight': 'balanced', 'C': 76.77, 'kernel': 'sigmoid', 'probability': True}
**OH-RBD7**	{'n_estimators': 140, 'max_depth': 2, 'min_samples_leaf': 0.1, 'min_samples_split': 0.6, 'max_features': 'log2'}
**OH-RBD8**	{'n_neighbors': 20, 'weights': 'distance', 'algorithm': 'kd_tree', 'leaf_size': 30, 'metric': 'chebyshev', 'metric_params': None, 'p': 2}
**OH-RBD9**	{'class_weight': 'balanced', 'n_estimators': 1000, 'max_depth': 2, 'min_samples_split': 0.6, 'min_samples_leaf': 0.2}
**OH-RBD10**	{'class_weight': 'balanced', 'criterion': 'gini', 'splitter': 'best', 'max_depth': 9, 'min_samples_split': 0.5, 'min_samples_leaf': 0.2, 'max_features': 'log2'}

7.3. 10000 random iterations.

For the random search with 10000 iterations, the ML classifiers and the thresholds were selected on the basis of best 5 models (Tables S12 and S13). Here the random search with 1000 iterations was run using SVM, GB and KNN and the thresholds values were reduced to 80.93, 82.598, 103.924, 144.346 and 157.662.

Table S62. Refined best models obtained for each classifier.

# Model	Threshold	ML	Average MCC_{Pred}	Average Normalized MCC_{Pred}	MCC_{Fit}	MCC_{CV}	Average ACC_{Pred}	Average $F1_{Pred}$	Average ROC_AUC_{Pred}
**OH-RBD11**	103.924	SVM	0.37	0.69	0.52	- 0.13	0.69	0.56	0.55
**OH-RBD12**	144.346	GB	0.49	0.74	0.77	0.01	0.74	0.74	0.37
**OH-RBD13**	144.346	KNN	0.48	0.74	1.00	- 0.09	0.74	0.75	0.28

Table S63. Hyperparameters associated to models **OH-RBD11-OH-RBD13**. The list is reported as python dictionaries.

# Model	Hyperparameters
**OH-RBD11**	{'class_weight': 'balanced', 'C': 75.28, 'kernel': 'sigmoid', 'probability': True}
**OH-RBD12**	{'n_estimators': 94, 'max_depth': 8, 'min_samples_leaf': 0.48, 'min_samples_split': 0.89, 'max_features': 'sqrt'}
**OH-RBD13**	{'n_neighbors': 17, 'weights': 'distance', 'algorithm': 'brute', 'leaf_size': 25, 'metric': 'chebyshev', 'metric_params': None, 'p': 5}

7.4. 100000 random iterations.

For the random search with 10000 iterations, the ML classifiers and the thresholds were selected on the basis of models in Tables S14 and S15. Here the random search with 100000 iterations was run using SVM, GB and KNN and the thresholds values were reduced to 80.93, 144.346 and 157.662.

Table S64. Refined best models obtained for each classifier.

# **Model**	**Threshold**	**ML**	**Average MCC_{Pred}**	**Average Normalized MCC_{Pred}**	**MCC_{Fit}**	**MCC_{CV}**	**Average ACC_{Pred}**	**Average $F1_{Pred}$**	**Average ROC_AUC_{Pred}**
**OH-RBD14**	144.346	SVM	0.32	0.66	0.41	- 0.05	0.67	0.71	0.64
**OH-RBD15**	157.662	GB	0.52	0.76	0.83	0.03	0.77	0.82	0.28
**OH-RBD16**	157.662	KNN	0.35	0.67	1.00	0.16	0.67	0.77	0.36

Table S65. Hyperparameters associated to models **OH-RBD14-OH-RBD16**. The list is reported as python dictionaries.

# **Model**	**Hyperparameters**
**OH-RBD14**	{'class_weight': None, 'C': 6.31, 'kernel': 'sigmoid', 'probability': True}
**OH-RBD15**	{'n_estimators': 93, 'max_depth': 14, 'min_samples_leaf': 0.45, 'min_samples_split': 0.15, 'max_features': 'sqrt'}
**OH-RBD16**	{'n_neighbors': 24, 'weights': 'distance', 'algorithm': 'ball_tree', 'leaf_size': 34, 'metric': 'chebyshev', 'metric_params': None, 'p': 4}

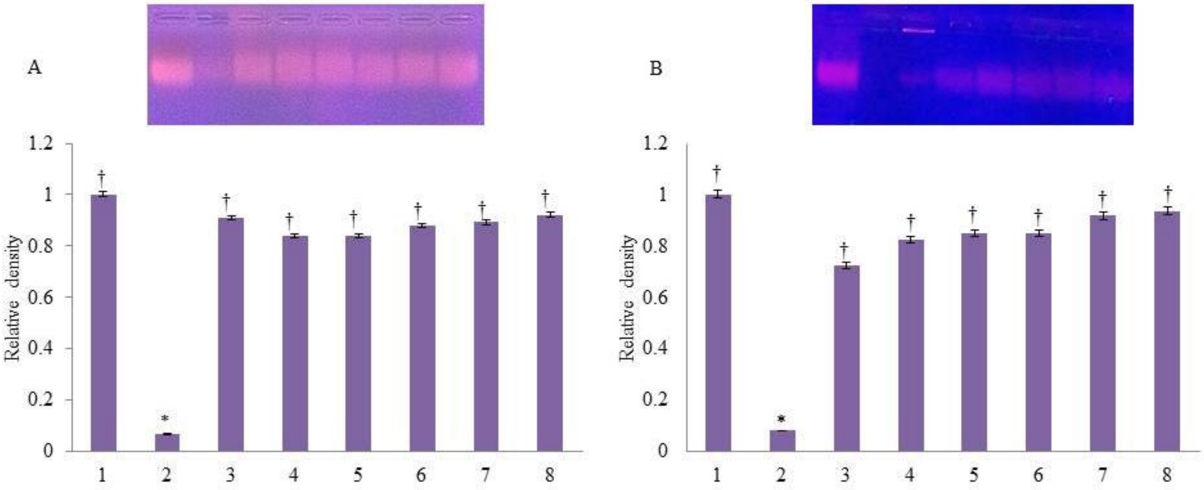

Figure S1. Protective effect of Chamomile morocco EO against peroxyl (A) and hydroxyl (B) radicals-induced DNA damage. DNA from salmon sperm (lane 1, negative control), DNA damage control (lane 2, positive control), quercetin (lane 3, 100 μg/mL, standard), essential oils in concentrations of 25, 50, 100, 200, and 400 μg/mL (lanes 4, 5, 6, 7, and 8). *$p < 0.05$ when compared with the negative control group †$p < 0.05$ when compared with the positive control group.

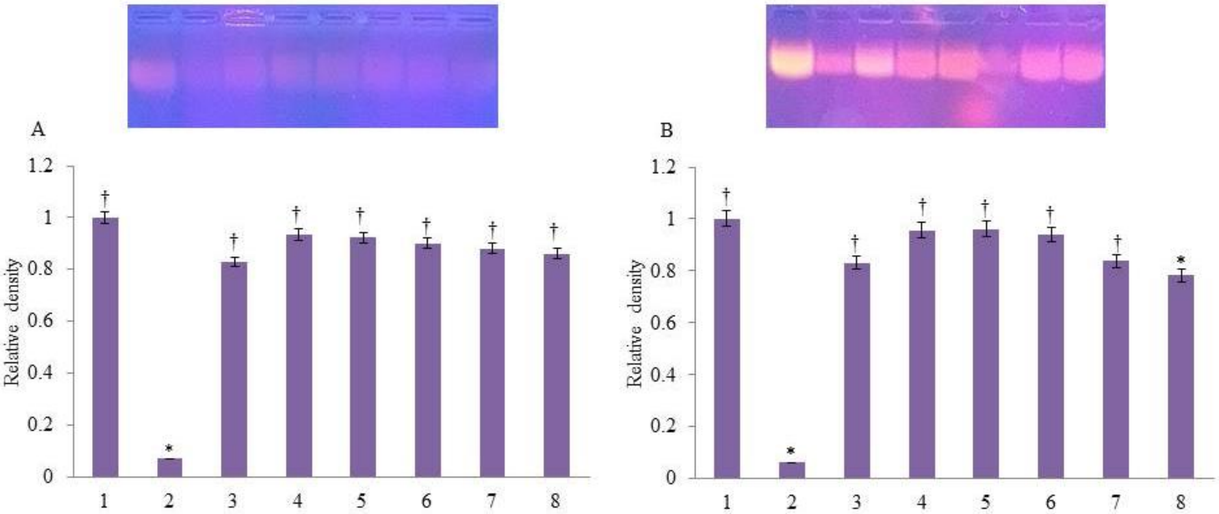

Figure S2. Protective effect of Clary sage EO against peroxyl (A) and hydroxyl (B) radicals-induced DNA damage. DNA from salmon sperm (lane 1, negative control), DNA damage control (lane 2, positive control), quercetin (lane 3, 100 μg/mL, standard), essential oils in concentrations of 25, 50, 100, 200, and 400 μg/mL (lanes 4, 5, 6, 7, and 8). *$p < 0.05$ when compared with the negative control group †$p < 0.05$ when compared with the positive control group.

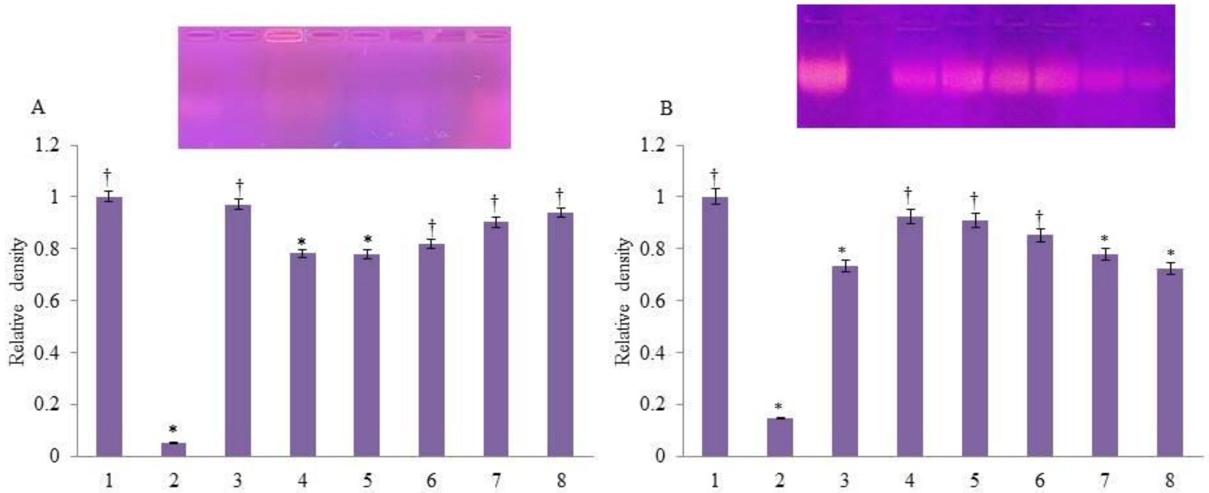

Figure S3. Protective effect of Sage EO against peroxyl (A) and hydroxyl (B) radicals-induced DNA damage. DNA from salmon sperm (lane 1, negative control), DNA damage control (lane 2, positive control), quercetin (lane 3, 100 μg/mL, standard), essential oils in concentrations of 25, 50, 100, 200, and 400 μg/mL (lanes 4, 5, 6, 7, and 8). $^{*}p < 0.05$ when compared with the negative control group $^{\dagger}p < 0.05$ when compared with the positive control group.

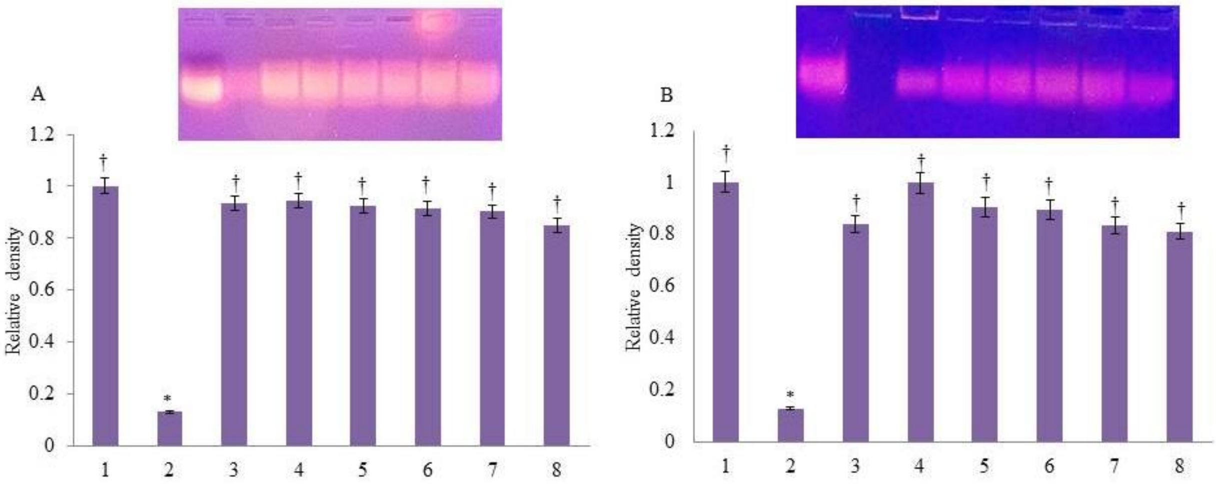

Figure S4. Protective effect of Red thyme EO against peroxyl (A) and hydroxyl (B) radicals-induced DNA damage. DNA from salmon sperm (lane 1, negative control), DNA damage control (lane 2, positive control), quercetin (lane 3, 100 μg/mL, standard), essential oils in concentrations of 25, 50, 100, 200, and 400 μg/mL (lanes 4, 5, 6, 7, and 8). $^{*}p < 0.05$ when compared with the negative control group $^{\dagger}p < 0.05$ when compared with the positive control group.

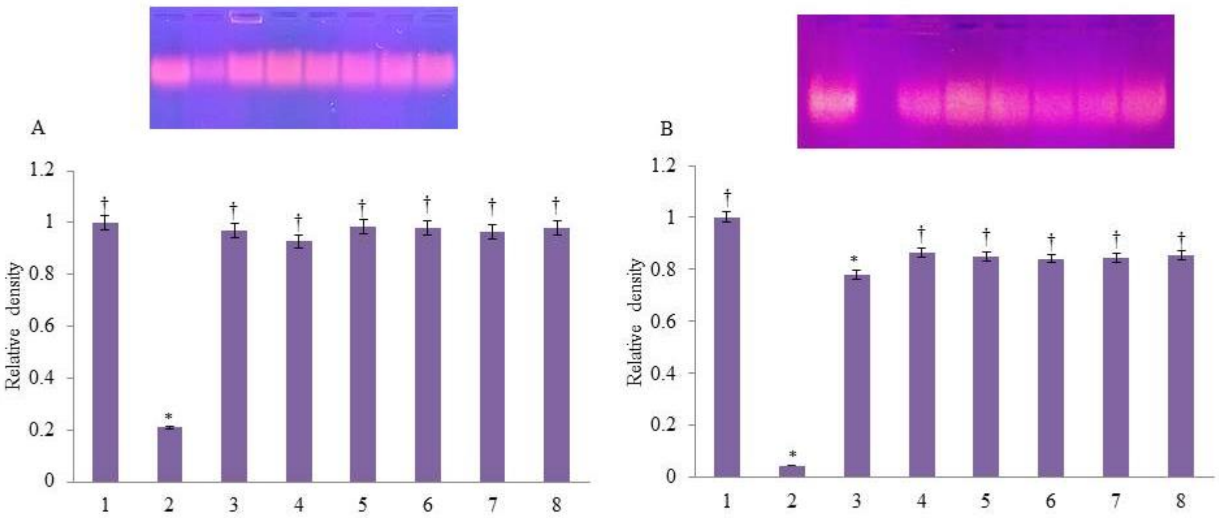

Figure S5. Protective effect Tea tree EO against peroxyl (A) and hydroxyl (B) radicals-induced DNA damage. DNA from salmon sperm (lane 1, negative control), DNA damage control (lane 2, positive control), quercetin (lane 3, 100 μg/mL,

standard), essential oils in concentrations of 25, 50, 100, 200, and 400 μg/mL (lanes 4, 5, 6, 7, and 8). *$p < 0.05$ when compared with the negative control group †$p < 0.05$ when compared with the positive control group.

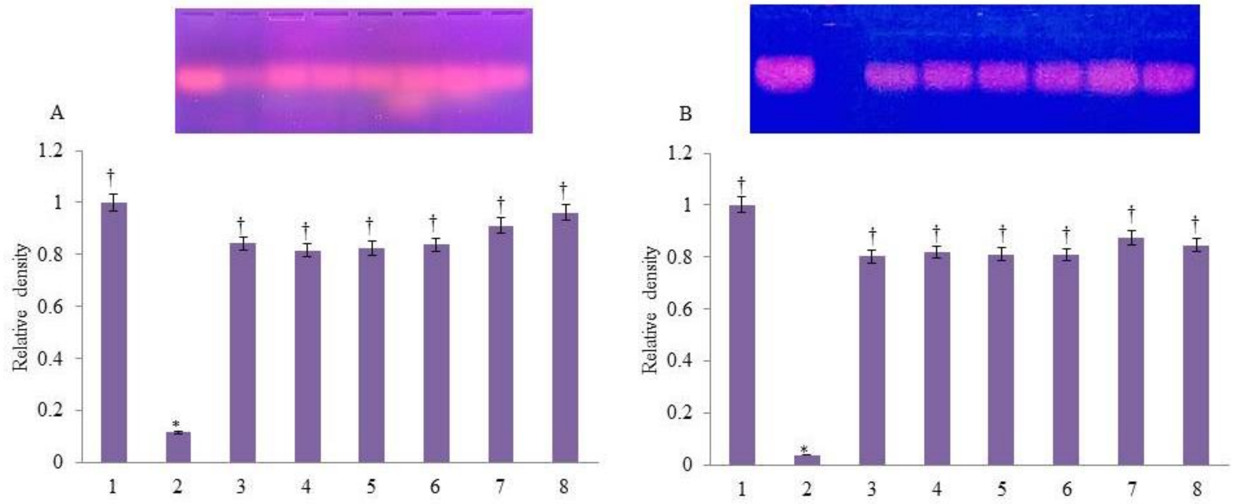

Figure S6. Protective effect of Melissa EO against peroxyl (A) and hydroxyl (B) radicals-induced DNA damage. DNA from salmon sperm (lane 1, negative control), DNA damage control (lane 2, positive control), quercetin (lane 3, 100 μg/mL, standard), essential oils in concentrations of 25, 50, 100, 200, and 400 μg/mL (lanes 4, 5, 6, 7, and 8). *$p < 0.05$ when compared with the negative control group †$p < 0.05$ when compared with the positive control group.

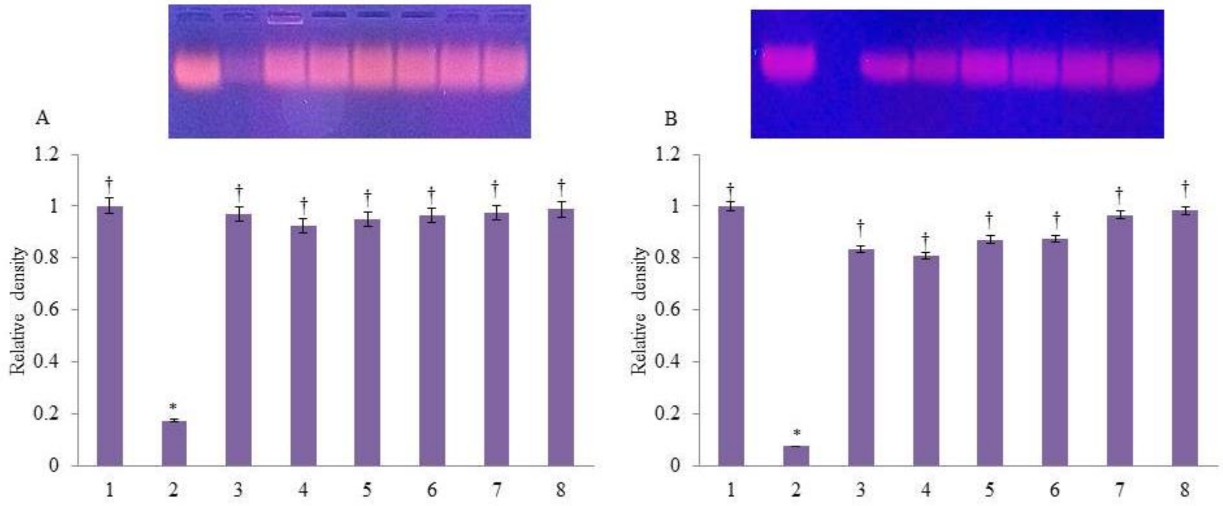

Figure S7. Protective effect of Mountain pine EO against peroxyl (A) and hydroxyl (B) radicals-induced DNA damage. DNA from salmon sperm (lane 1, negative control), DNA damage control (lane 2, positive control), quercetin (lane 3, 100 μg/mL, standard), essential oils in concentrations of 25, 50, 100, 200, and 400 μg/mL (lanes 4, 5, 6, 7, and 8). *$p < 0.05$ when compared with the negative control group †$p < 0.05$ when compared with the positive control group.

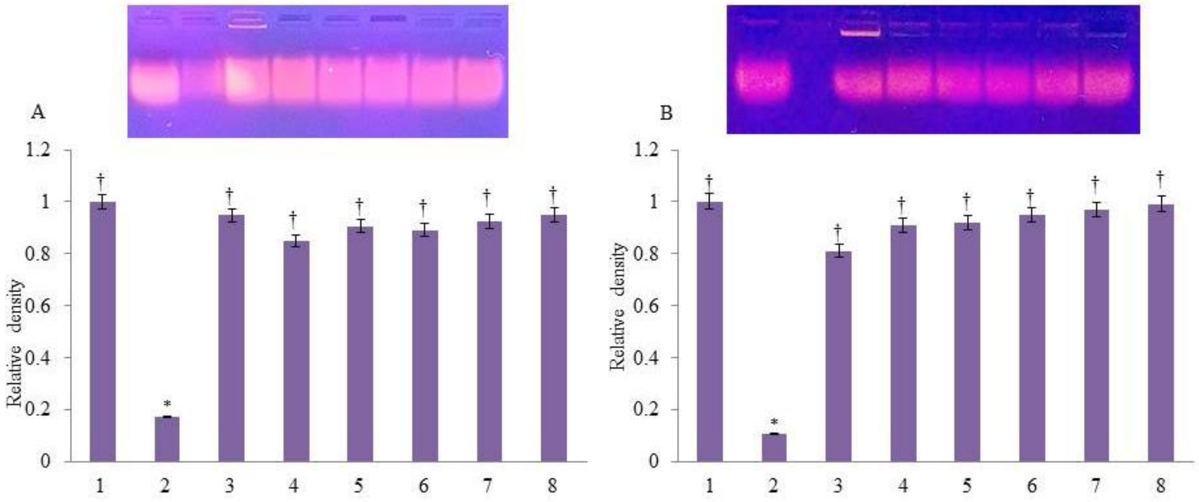

Figure S8. Protective effect of Geranium bourbon EO against peroxyl (A) and hydroxyl (B) radicals-induced DNA damage. DNA from salmon sperm (lane 1, negative control), DNA damage control (lane 2, positive control), quercetin (lane 3, 100 μg/mL, standard), essential oils in concentrations of 25, 50, 100, 200, and 400 μg/mL (lanes 4, 5, 6, 7, and 8). *$p < 0.05$ when compared with the negative control group $^{\dagger}p < 0.05$ when compared with the positive control group.

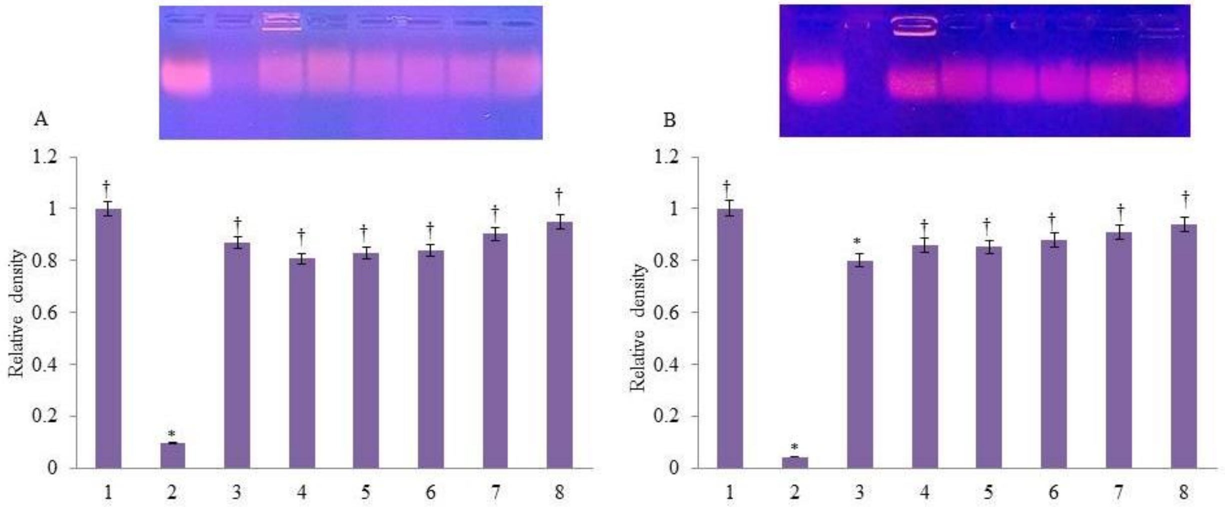

Figure S9. Protective effect of Oregano EO against peroxyl (A) and hydroxyl (B) radicals-induced DNA damage. DNA from salmon sperm (lane 1, negative control), DNA damage control (lane 2, positive control), quercetin (lane 3, 100 μg/mL, standard), essential oils in concentrations of 25, 50, 100, 200, and 400 μg/mL (lanes 4, 5, 6, 7, and 8). *$p < 0.05$ when compared with the negative control group $^{\dagger}p < 0.05$ when compared with the positive control group.

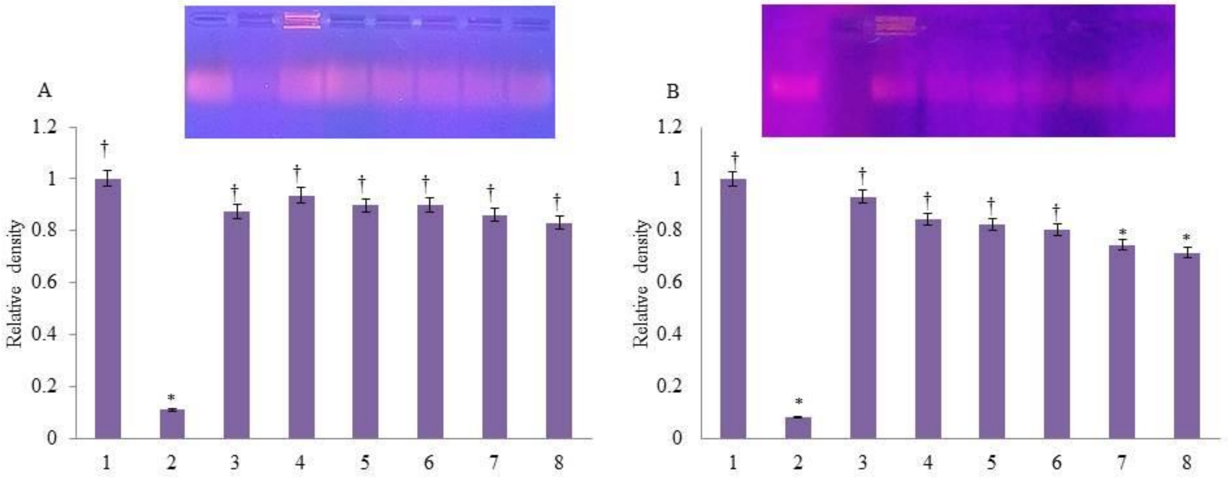

Figure S10. Protective effect of Coriander EO against peroxyl (A) and hydroxyl (B) radicals-induced DNA damage. DNA from salmon sperm (lane 1, negative control), DNA damage control (lane 2, positive control), quercetin (lane 3, 100 μg/mL, standard), essential oils in concentrations of 25, 50, 100, 200, and 400 μg/mL (lanes 4, 5, 6, 7, and 8). *$p < 0.05$ when compared with the negative control group $^{\dagger}p < 0.05$ when compared with the positive control group.

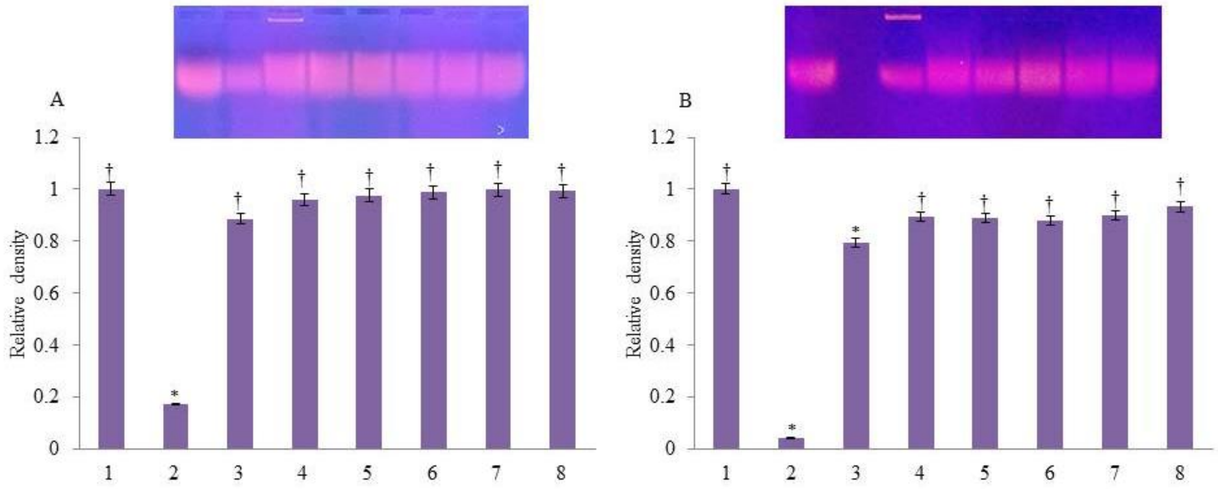

Figure S11. Protective effect of Lavander EO against peroxyl (A) and hydroxyl (B) radicals-induced DNA damage. DNA from salmon sperm (lane 1, negative control), DNA damage control (lane 2, positive control), quercetin (lane 3, 100 μg/mL, standard), essential oils in concentrations of 25, 50, 100, 200, and 400 μg/mL (lanes 4, 5, 6, 7, and 8). *$p < 0.05$ when compared with the negative control group †$p < 0.05$ when compared with the positive control group.

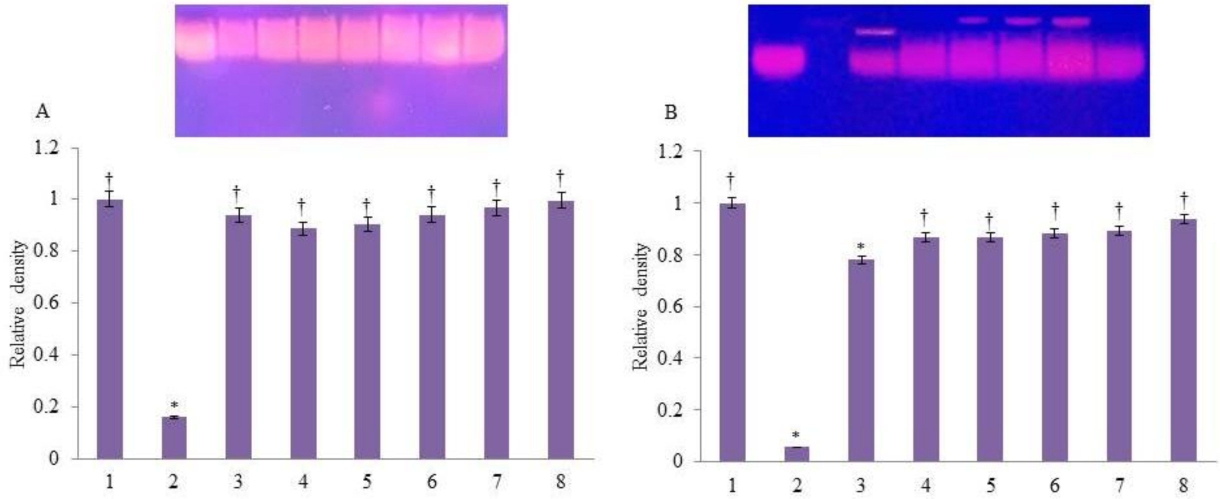

Figure S12. Protective effect of Myrtle EO against peroxyl (A) and hydroxyl (B) radicals-induced DNA damage. DNA from salmon sperm (lane 1, negative control), DNA damage control (lane 2, positive control), quercetin (lane 3, 100 μg/mL, standard), essential oils in concentrations of 25, 50, 100, 200, and 400 μg/mL (lanes 4, 5, 6, 7, and 8). *$p < 0.05$ when compared with the negative control group †$p < 0.05$ when compared with the positive control group.

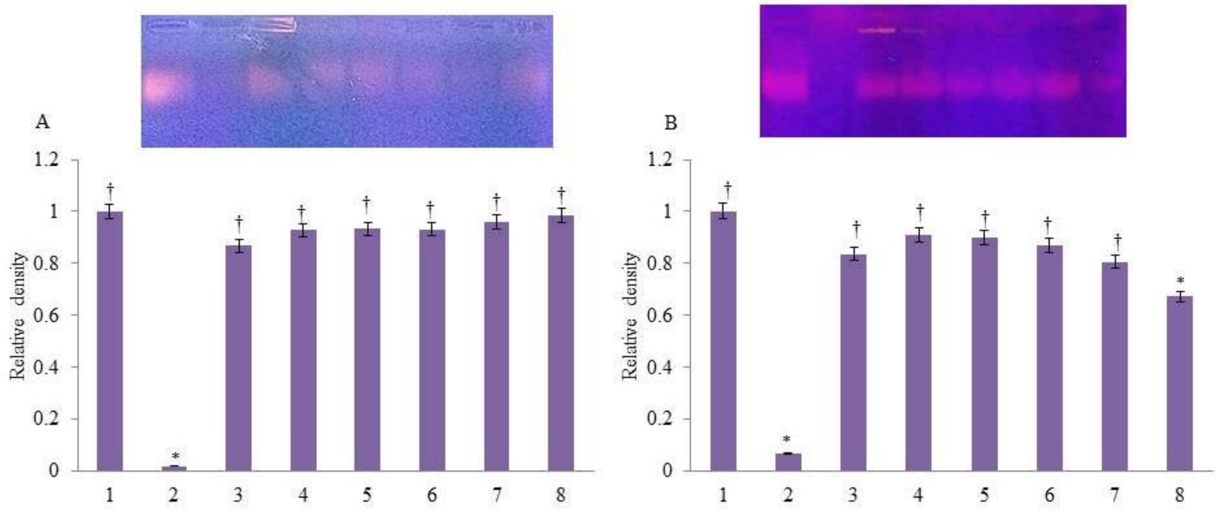

Figure S13. Protective effect of Garlic EO against peroxyl (A) and hydroxyl (B) radicals-induced DNA damage. DNA from salmon sperm (lane 1, negative control), DNA damage control (lane 2, positive control), quercetin (lane 3, 100 μg/mL,

standard), essential oils in concentrations of 25, 50, 100, 200, and 400 μg/mL (lanes 4, 5, 6, 7, and 8). $^{*}p < 0.05$ when compared with the negative control group $^{\dagger}p < 0.05$ when compared with the positive control group.

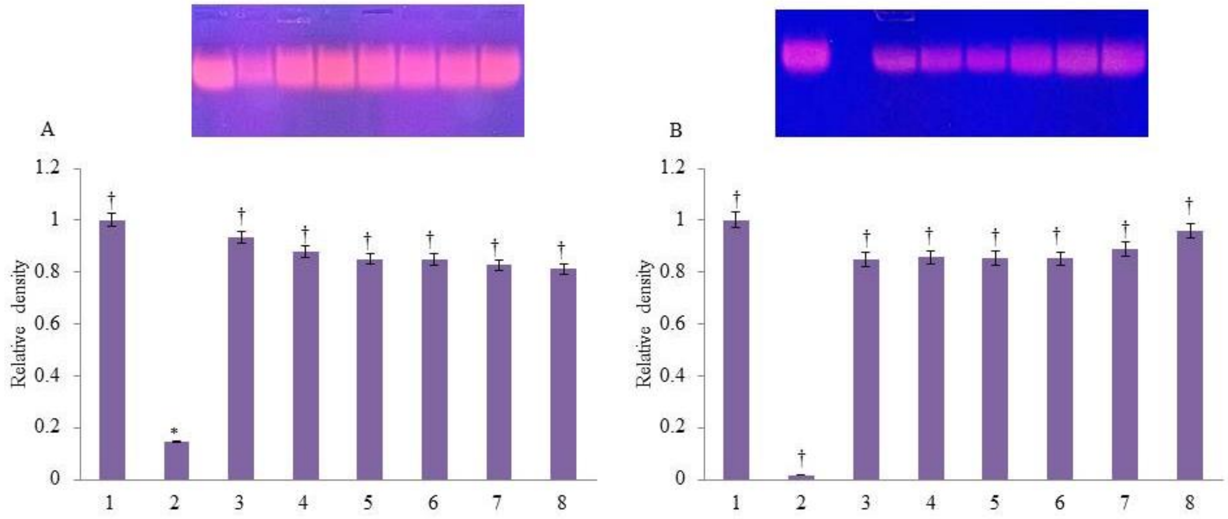

Figure S14. Protective effect of Cardamom EO against peroxyl (A) and hydroxyl (B) radicals-induced DNA damage. DNA from salmon sperm (lane 1, negative control), DNA damage control (lane 2, positive control), quercetin (lane 3, 100 μg/mL, standard), essential oils in concentrations of 25, 50, 100, 200, and 400 μg/mL (lanes 4, 5, 6, 7, and 8). $^{*}p < 0.05$ when compared with the negative control group $^{\dagger}p < 0.05$ when compared with the positive control group.

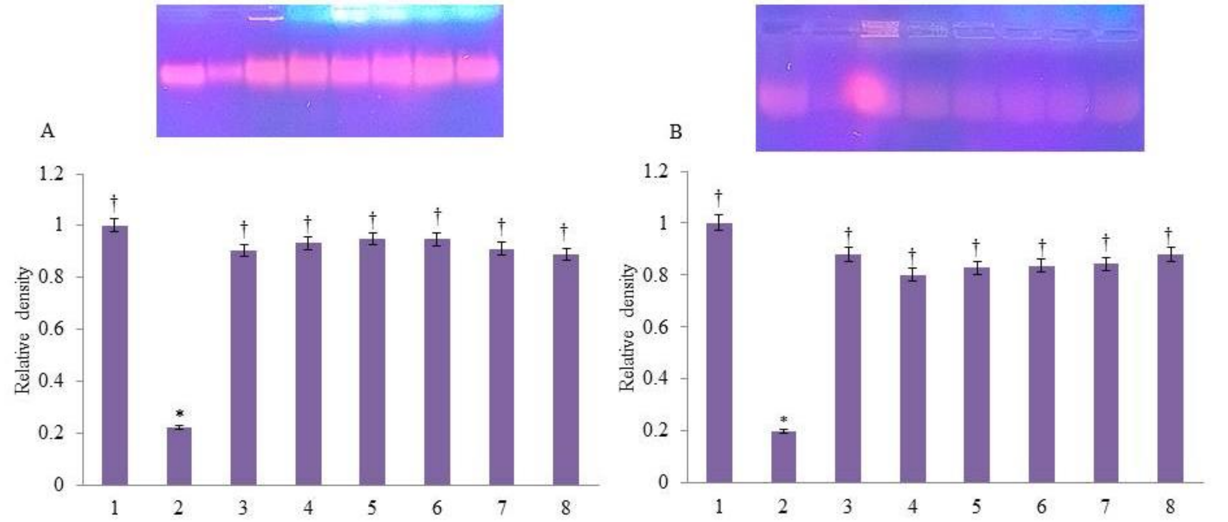

Figure S15. Protective effect of Mandarin EO against peroxyl (A) and hydroxyl (B) radicals-induced DNA damage. DNA from salmon sperm (lane 1, negative control), DNA damage control (lane 2, positive control), quercetin (lane 3, 100 μg/mL, standard), essential oils in concentrations of 25, 50, 100, 200, and 400 μg/mL (lanes 4, 5, 6, 7, and 8). $^{*}p < 0.05$ when compared with the negative control group $^{\dagger}p < 0.05$ when compared with the positive control group.

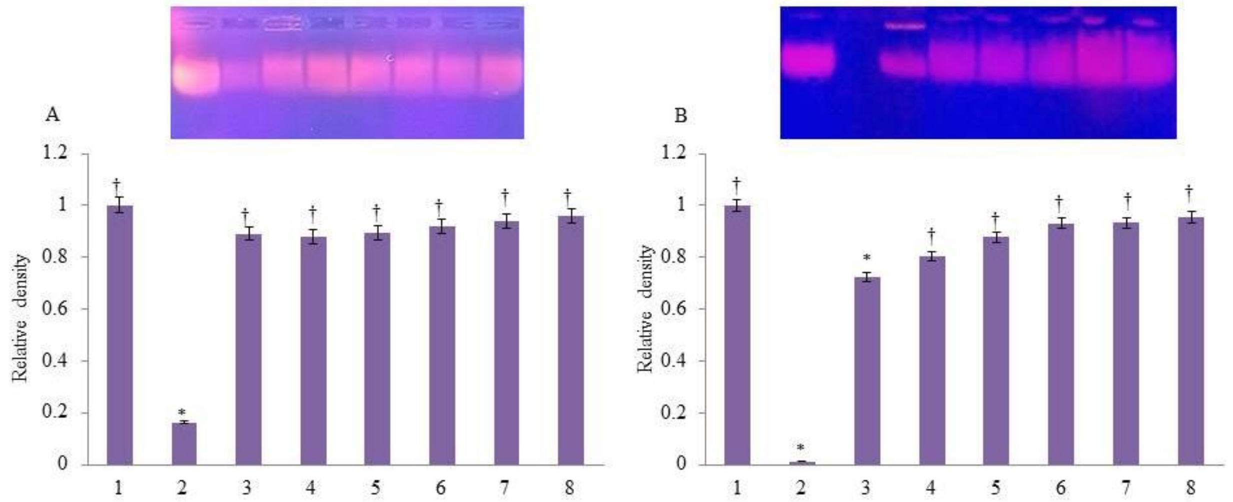

Figure S16. Protective effect of Hyssop EO against peroxyl (A) and hydroxyl (B) radicals-induced DNA damage. DNA from salmon sperm (lane 1, negative control), DNA damage control (lane 2, positive control), quercetin (lane 3, 100 μg/mL, standard), essential oils in concentrations of 25, 50, 100, 200, and 400 μg/mL (lanes 4, 5, 6, 7, and 8). $^{*}p < 0.05$ when compared with the negative control group $^{\dagger}p < 0.05$ when compared with the positive control group.

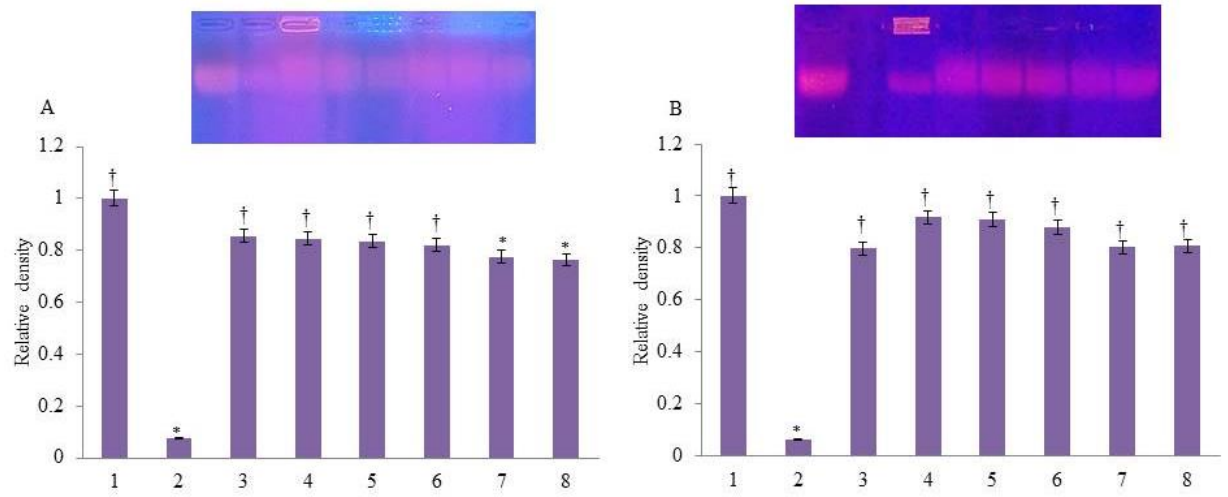

Figure S17. Protective effect of Grapefruit EO against peroxyl (A) and hydroxyl (B) radicals-induced DNA damage. DNA from salmon sperm (lane 1, negative control), DNA damage control (lane 2, positive control), quercetin (lane 3, 100 μg/mL, standard), essential oils in concentrations of 25, 50, 100, 200, and 400 μg/mL (lanes 4, 5, 6, 7, and 8). $^{*}p < 0.05$ when compared with the negative control group $^{\dagger}p < 0.05$ when compared with the positive control group.

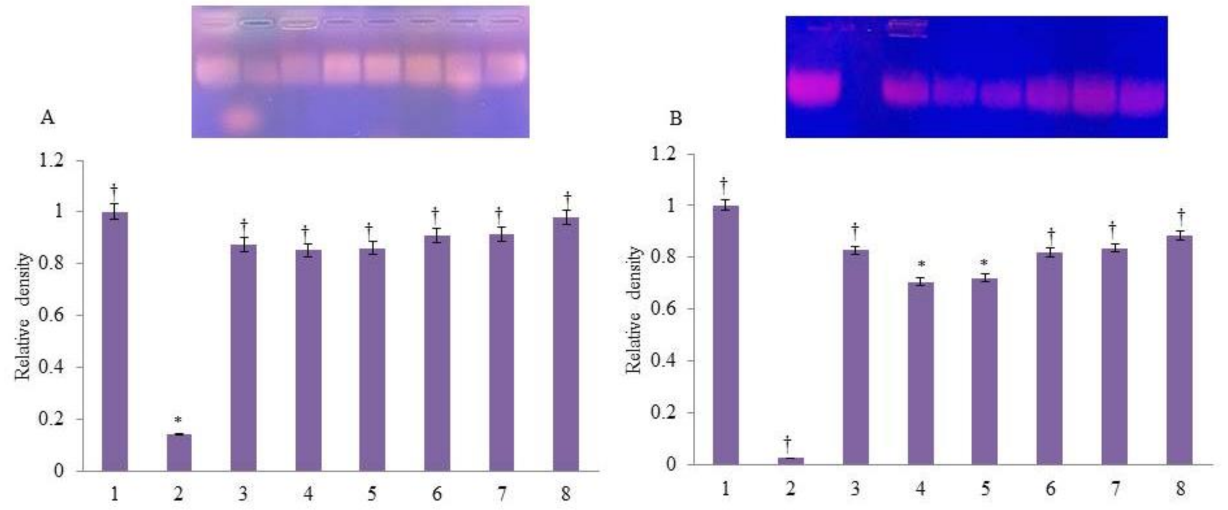

Figure S18. Protective effect of Lemongrass EO against peroxyl (A) and hydroxyl (B) radicals-induced DNA damage. DNA from salmon sperm (lane 1, negative control), DNA damage control (lane 2, positive control), quercetin (lane 3, 100 μg/mL,

standard), essential oils in concentrations of 25, 50, 100, 200, and 400 µg/mL (lanes 4, 5, 6, 7, and 8). *p < 0.05 when compared with the negative control group †p < 0.05 when compared with the positive control group.

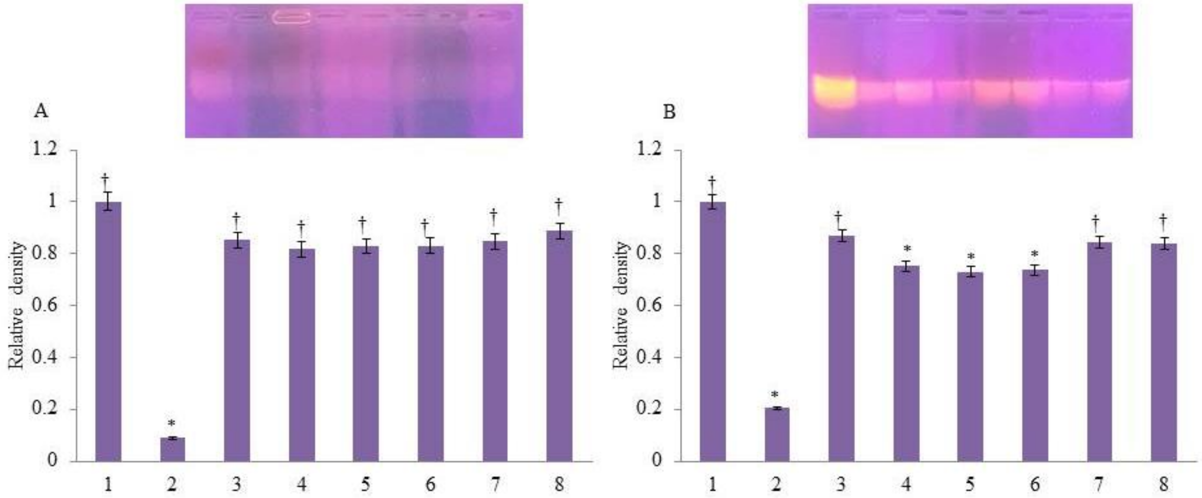

Figure S19. Protective effect of Siberian pine EO against peroxyl (A) and hydroxyl (B) radicals-induced DNA damage. DNA from salmon sperm (lane 1, negative control), DNA damage control (lane 2, positive control), quercetin (lane 3, 100 µg/mL, standard), essential oils in concentrations of 25, 50, 100, 200, and 400 µg/mL (lanes 4, 5, 6, 7, and 8). *p < 0.05 when compared with the negative control group †p < 0.05 when compared with the positive control group.

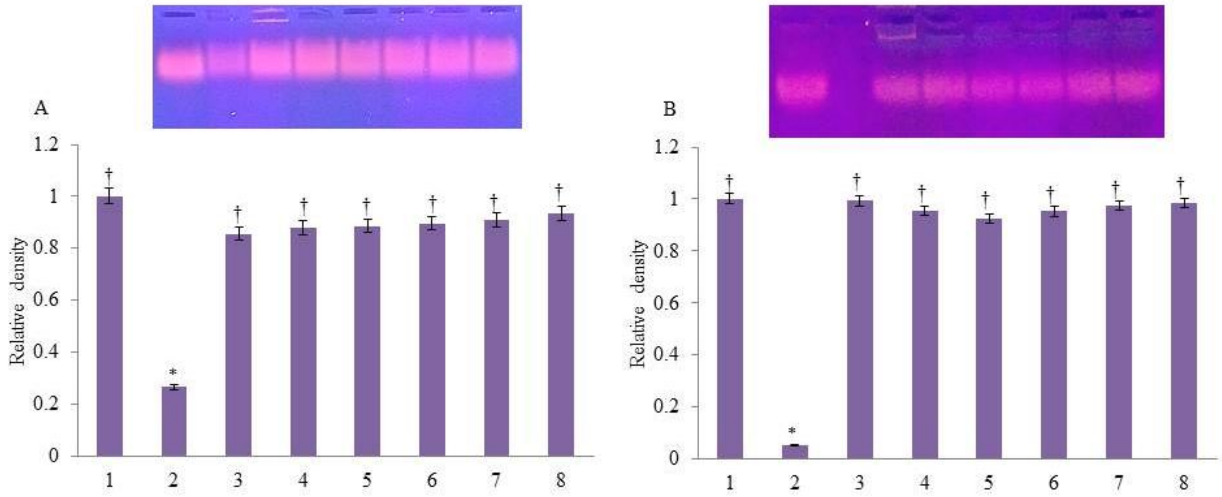

Figure S20. Protective effect of Camphor EO against peroxyl (A) and hydroxyl (B) radicals-induced DNA damage. DNA from salmon sperm (lane 1, negative control), DNA damage control (lane 2, positive control), quercetin (lane 3, 100 µg/mL, standard), essential oils in concentrations of 25, 50, 100, 200, and 400 µg/mL (lanes 4, 5, 6, 7, and 8). *p < 0.05 when compared with the negative control group †p < 0.05 when compared with the positive control group.

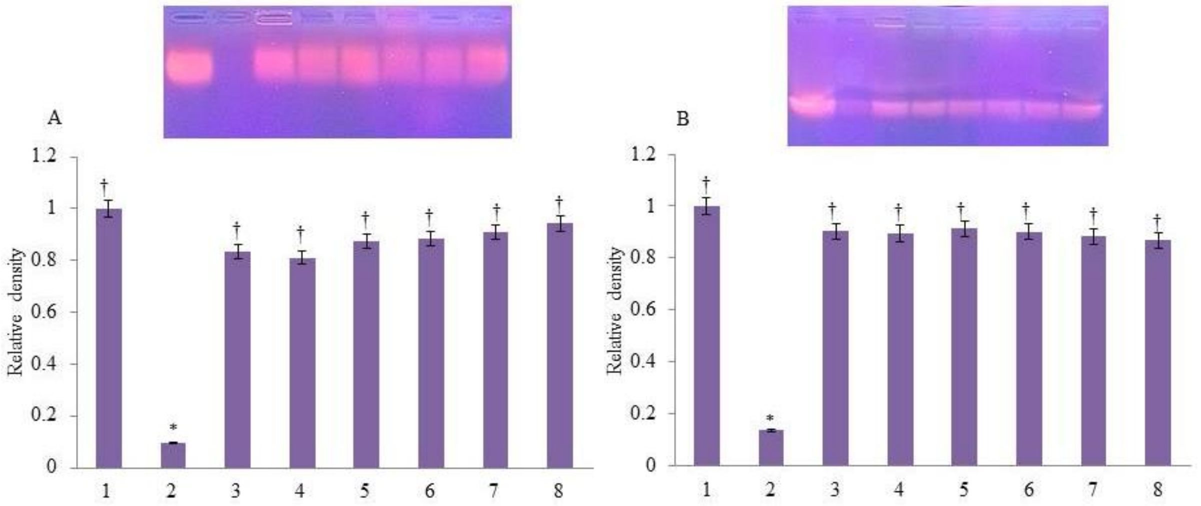

Figure S21. Protective effect of Cade EO against peroxyl (A) and hydroxyl (B) radicals-induced DNA damage. DNA from salmon sperm (lane 1, negative control), DNA damage control (lane 2, positive control), quercetin (lane 3, 100 µg/mL, standard), essential oils in concentrations of 25, 50, 100, 200, and 400 µg/mL (lanes 4, 5, 6, 7, and 8). *$p < 0.05$ when compared with the negative control group $^{\dagger}p < 0.05$ when compared with the positive control group.

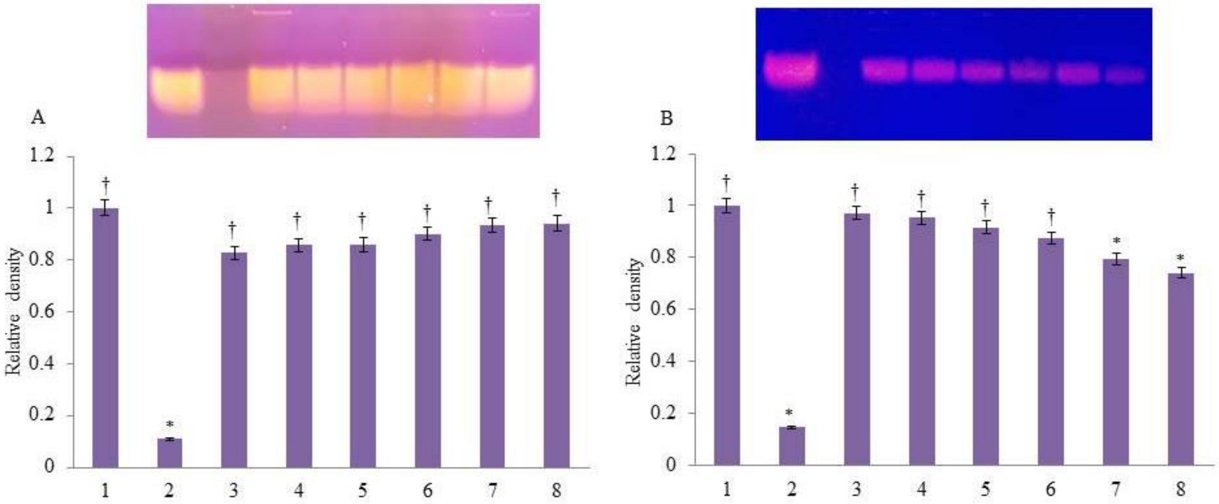

Figure S22. Protective effect of Cedar leaves EO against peroxyl (A) and hydroxyl (B) radicals-induced DNA damage. DNA from salmon sperm (lane 1, negative control), DNA damage control (lane 2, positive control), quercetin (lane 3, 100 µg/mL, standard), essential oils in concentrations of 25, 50, 100, 200, and 400 µg/mL (lanes 4, 5, 6, 7, and 8). *$p < 0.05$ when compared with the negative control group $^{\dagger}p < 0.05$ when compared with the positive control group.

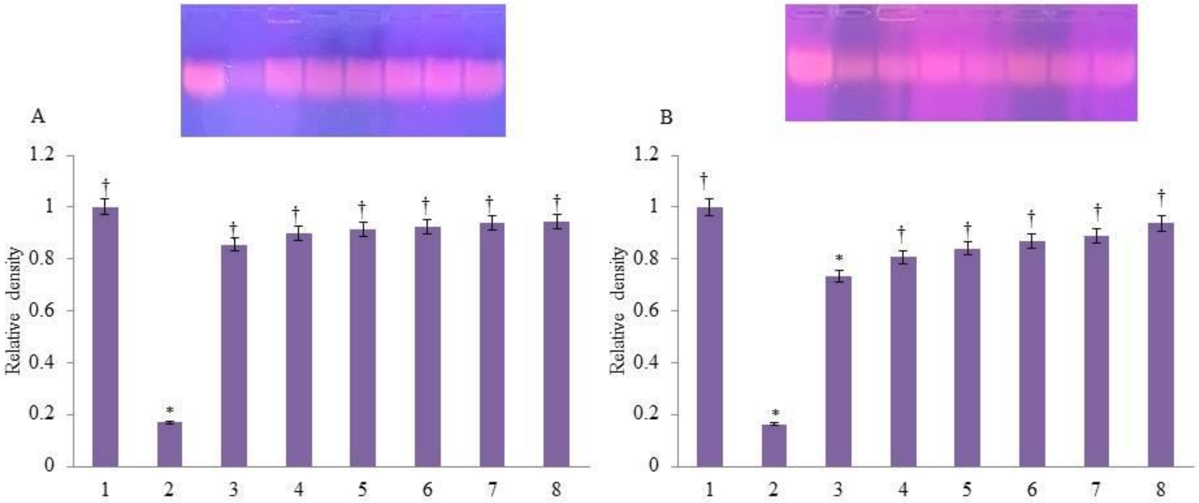

Figure S23. Protective effect of Ginger EO against peroxyl (A) and hydroxyl (B) radicals-induced DNA damage. DNA from salmon sperm (lane 1, negative control), DNA damage control (lane 2, positive control), quercetin (lane 3, 100 µg/mL, standard), essential oils in concentrations of 25, 50, 100, 200, and 400 µg/mL (lanes 4, 5, 6, 7, and 8). *$p < 0.05$ when compared with the negative control group $^{\dagger}p < 0.05$ when compared with the positive control group.

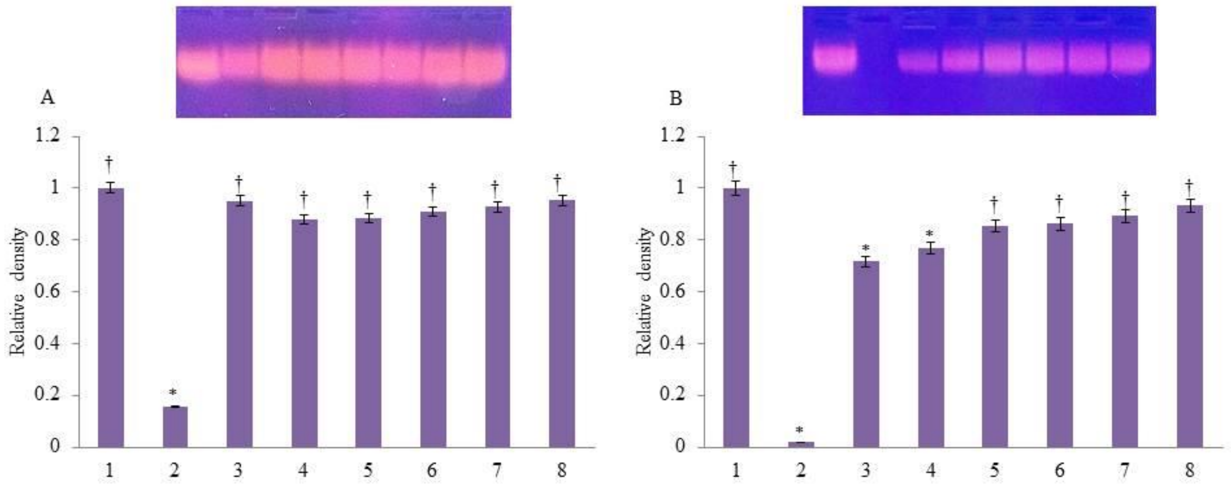

Figure S24. Protective effect of Cumin EO against peroxyl (A) and hydroxyl (B) radicals-induced DNA damage. DNA from salmon sperm (lane 1, negative control), DNA damage control (lane 2, positive control), quercetin (lane 3, 100 μg/mL, standard), essential oils in concentrations of 25, 50, 100, 200, and 400 μg/mL (lanes 4, 5, 6, 7, and 8). *$p < 0.05$ when compared with the negative control group †$p < 0.05$ when compared with the positive control group.

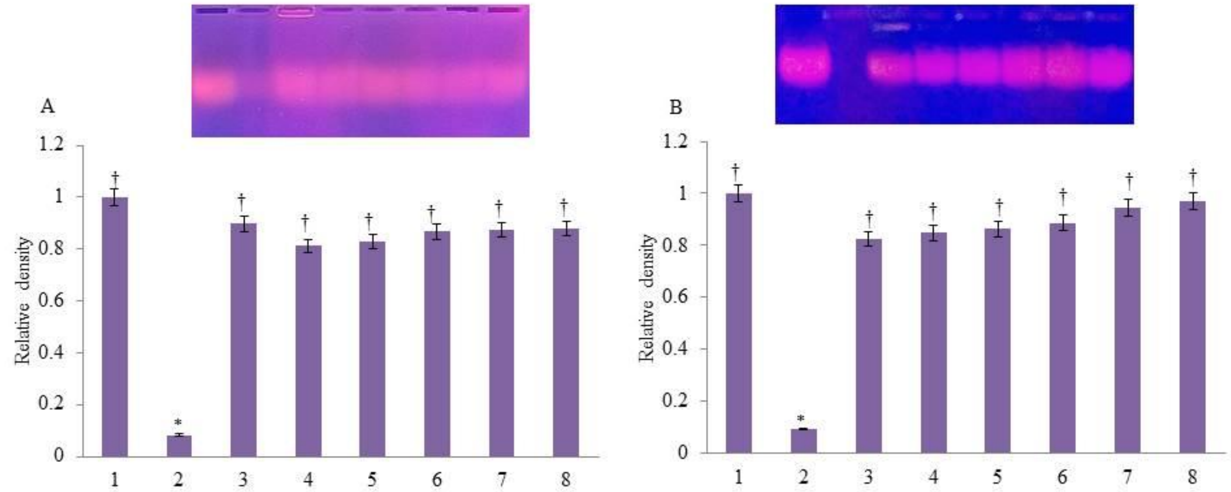

Figure S25. Protective effect of Patchouli EO against peroxyl (A) and hydroxyl (B) radicals-induced DNA damage. DNA from salmon sperm (lane 1, negative control), DNA damage control (lane 2, positive control), quercetin (lane 3, 100 μg/mL, standard), essential oils in concentrations of 25, 50, 100, 200, and 400 μg/mL (lanes 4, 5, 6, 7, and 8). *$p < 0.05$ when compared with the negative control group †$p < 0.05$ when compared with the positive control group.

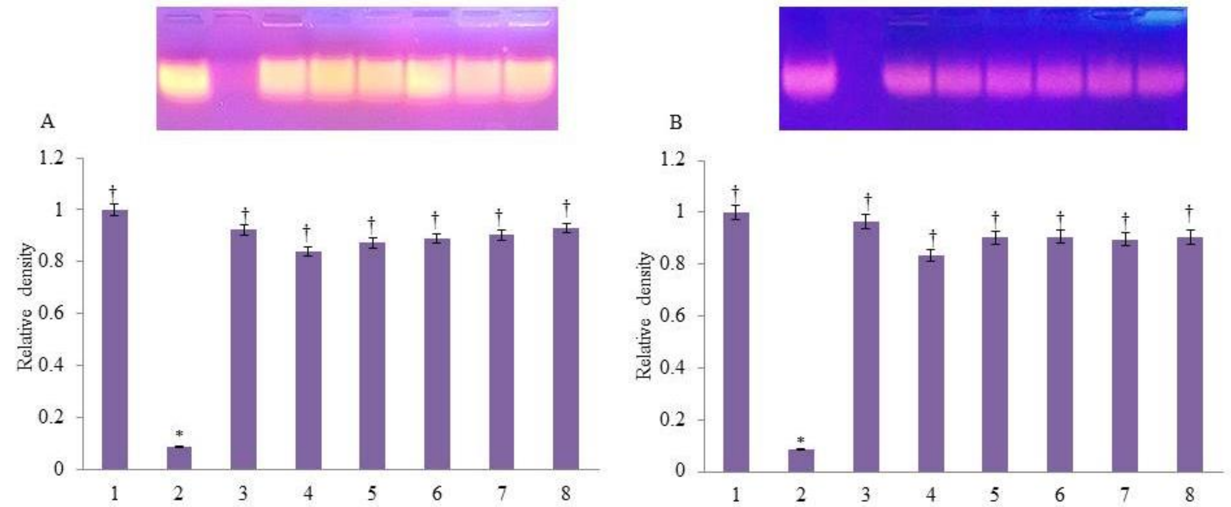

Figure S26. Protective effect of Orange bitter EO against peroxyl (A) and hydroxyl (B) radicals-induced DNA damage. DNA from salmon sperm (lane 1, negative control), DNA damage control (lane 2, positive control), quercetin (lane 3, 100 μg/mL,

standard), essential oils in concentrations of 25, 50, 100, 200, and 400 µg/mL (lanes 4, 5, 6, 7, and 8). $^*p < 0.05$ when compared with the negative control group $^{\dagger}p < 0.05$ when compared with the positive control group.

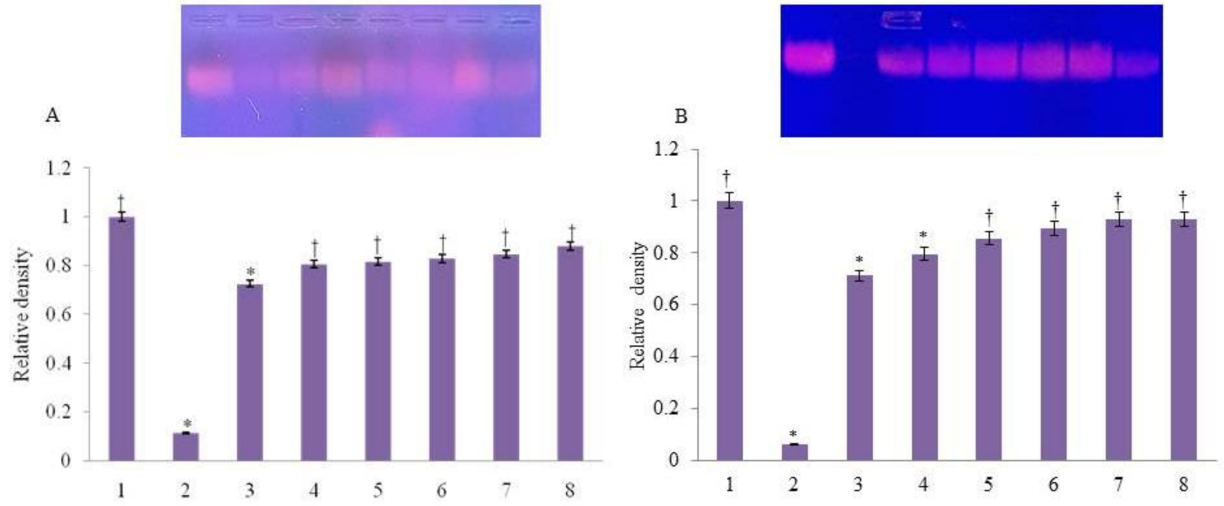

Figure S27. Protective effect of Eucalyptus EO against peroxyl (A) and hydroxyl (B) radicals-induced DNA damage. DNA from salmon sperm (lane 1, negative control), DNA damage control (lane 2, positive control), quercetin (lane 3, 100 µg/mL, standard), essential oils in concentrations of 25, 50, 100, 200, and 400 µg/mL (lanes 4, 5, 6, 7, and 8). $^*p < 0.05$ when compared with the negative control group $^{\dagger}p < 0.05$ when compared with the positive control group.

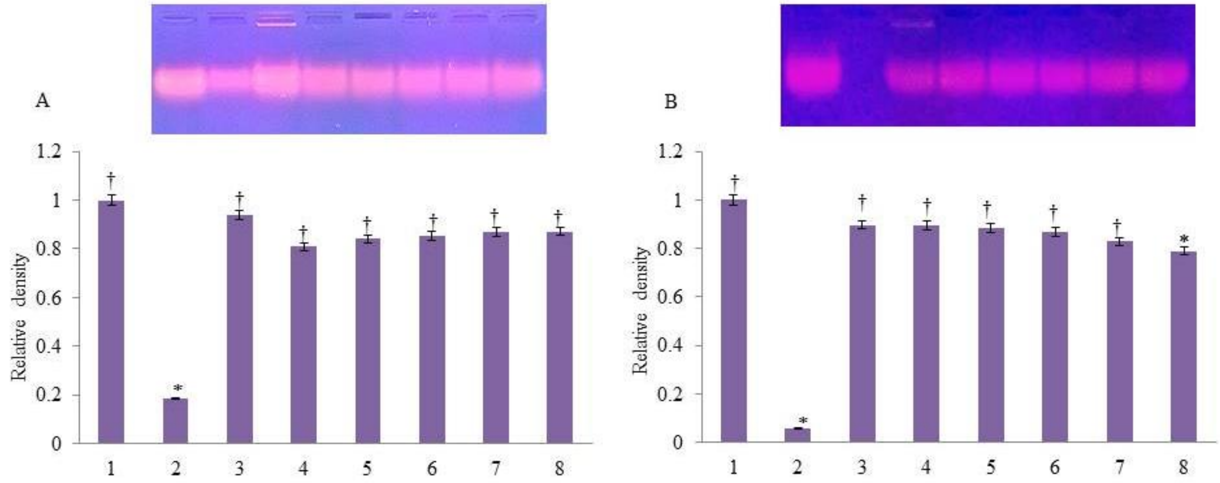

Figure S28. Protective effect of Pine silvestre natural EO against peroxyl (A) and hydroxyl (B) radicals-induced DNA damage. DNA from salmon sperm (lane 1, negative control), DNA damage control (lane 2, positive control), quercetin (lane 3, 100 µg/mL, standard), essential oils in concentrations of 25, 50, 100, 200, and 400 µg/mL (lanes 4, 5, 6, 7, and 8). $^*p < 0.05$ when compared with the negative control group $^{\dagger}p < 0.05$ when compared with the positive control group.

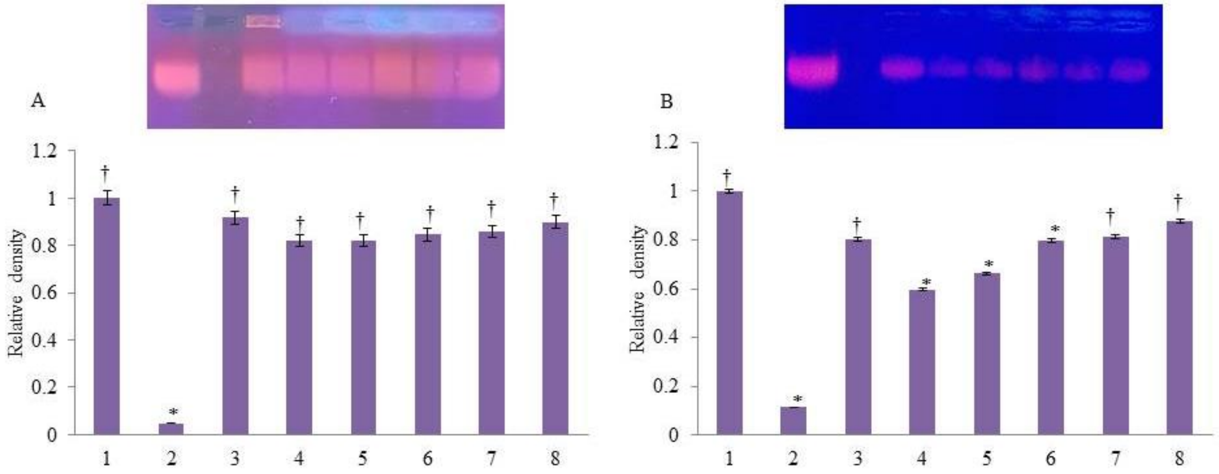

Figure S29. Protective effect of Bergamot EO against peroxyl (A) and hydroxyl (B) radicals-induced DNA damage. DNA from salmon sperm (lane 1, negative control), DNA damage control (lane 2, positive control), quercetin (lane 3, 100 µg/mL, standard), essential oils in concentrations of 25, 50, 100, 200, and 400 µg/mL (lanes 4, 5, 6, 7, and 8). *$p < 0.05$ when compared with the negative control group †$p < 0.05$ when compared with the positive control group.

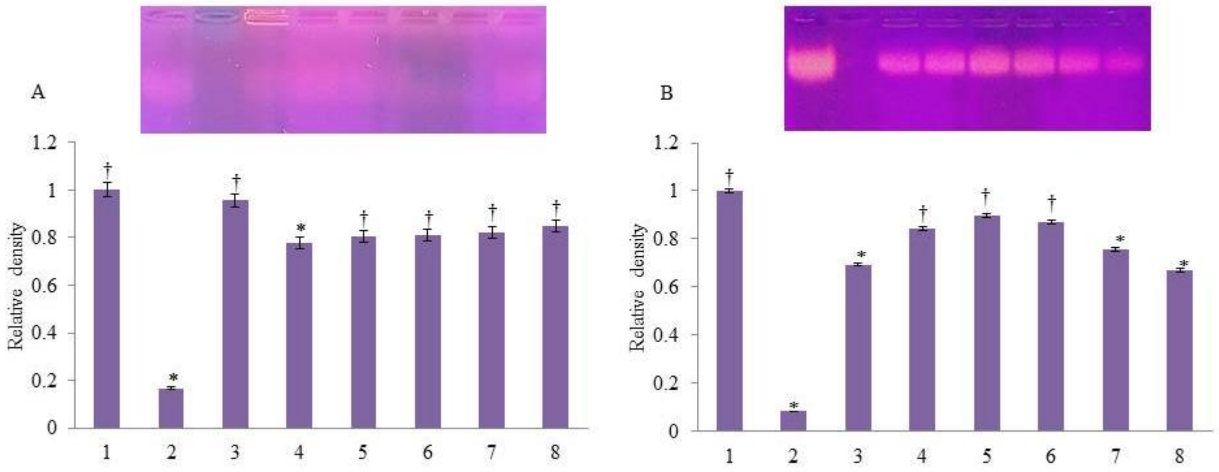

Figure S30. Protective effect of Juniper EO against peroxyl (A) and hydroxyl (B) radicals-induced DNA damage. DNA from salmon sperm (lane 1, negative control), DNA damage control (lane 2, positive control), quercetin (lane 3, 100 µg/mL, standard), essential oils in concentrations of 25, 50, 100, 200, and 400 µg/mL (lanes 4, 5, 6, 7, and 8). *$p < 0.05$ when compared with the negative control group †$p < 0.05$ when compared with the positive control group.

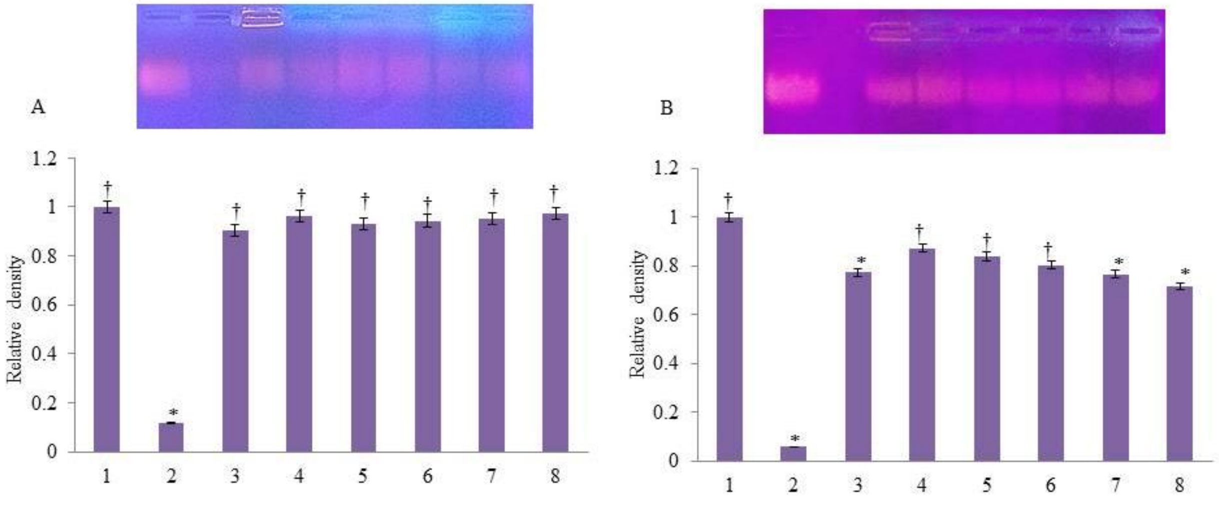

Figure S31. Protective effect of Birch EO against peroxyl (A) and hydroxyl (B) radicals-induced DNA damage. DNA from salmon sperm (lane 1, negative control), DNA damage control (lane 2, positive control), quercetin (lane 3, 100 µg/mL,

standard), essential oils in concentrations of 25, 50, 100, 200, and 400 μg/mL (lanes 4, 5, 6, 7, and 8). *$p < 0.05$ when compared with the negative control group †$p < 0.05$ when compared with the positive control group.

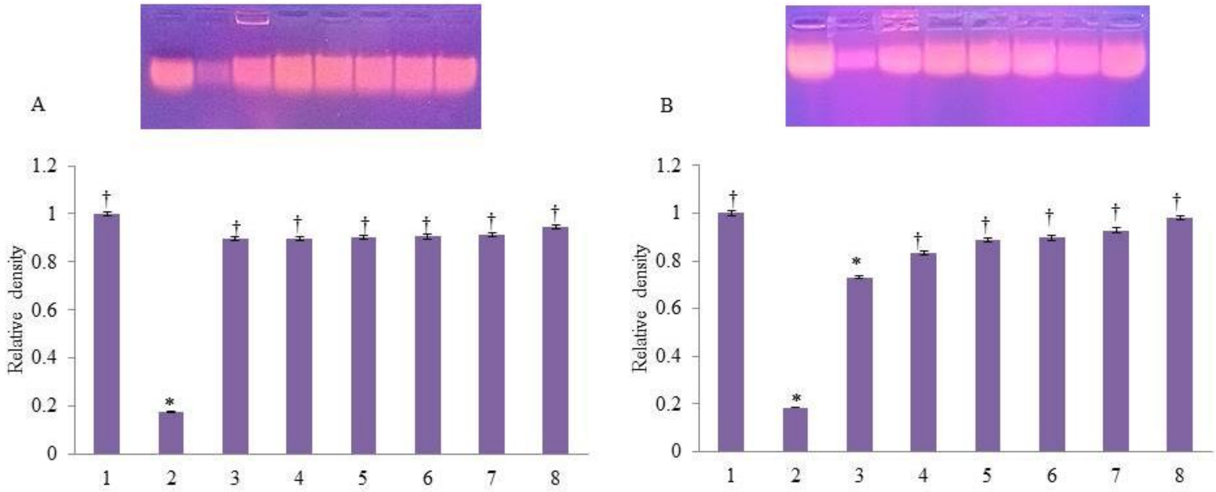

Figure S32. Protective effect of Fennel EO against peroxyl (A) and hydroxyl (B) radicals-induced DNA damage. DNA from salmon sperm (lane 1, negative control), DNA damage control (lane 2, positive control), quercetin (lane 3, 100 μg/mL, standard), essential oils in concentrations of 25, 50, 100, 200, and 400 μg/mL (lanes 4, 5, 6, 7, and 8). *$p < 0.05$ when compared with the negative control group †$p < 0.05$ when compared with the positive control group.

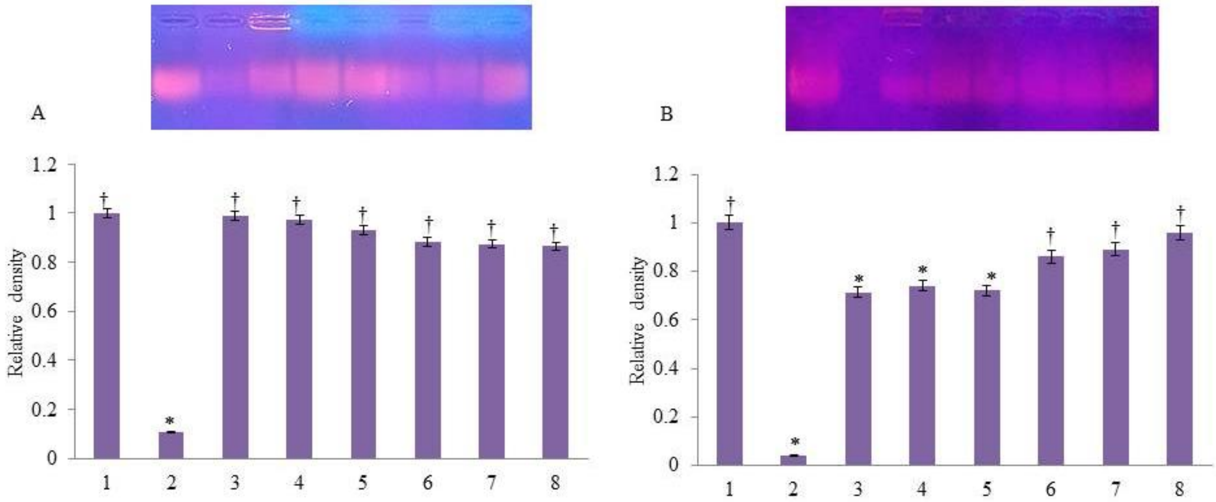

Figure S33. Protective effect of Cedar fruit EO against peroxyl (A) and hydroxyl (B) radicals-induced DNA damage. DNA from salmon sperm (lane 1, negative control), DNA damage control (lane 2, positive control), quercetin (lane 3, 100 μg/mL, standard), essential oils in concentrations of 25, 50, 100, 200, and 400 μg/mL (lanes 4, 5, 6, 7, and 8). *$p < 0.05$ when compared with the negative control group †$p < 0.05$ when compared with the positive control group.

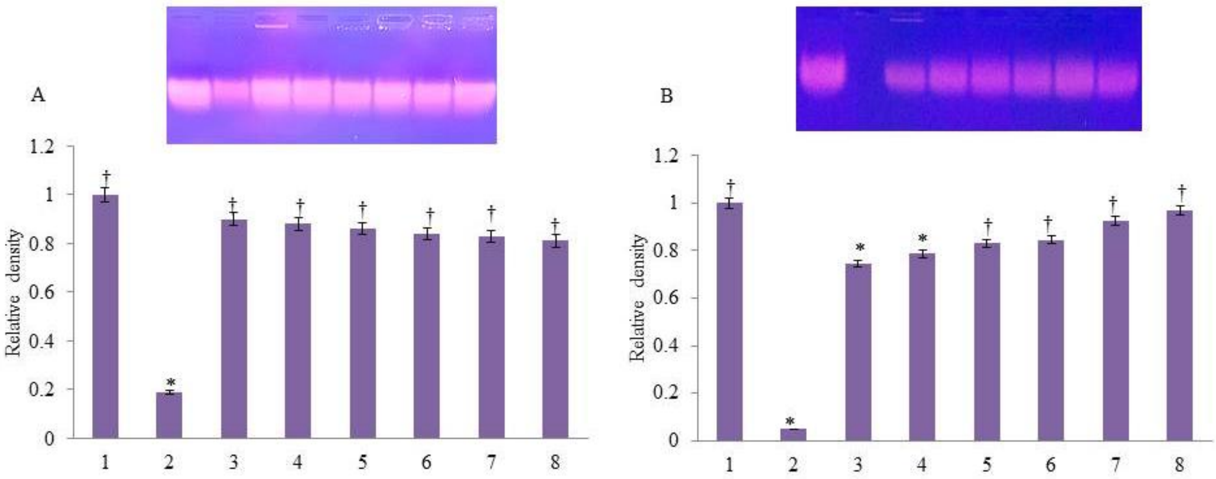

Figure S34. Protective effect of Lemon EO against peroxyl (A) and hydroxyl (B) radicals-induced DNA damage. DNA from salmon sperm (lane 1, negative control), DNA damage control (lane 2, positive control), quercetin (lane 3, 100 μg/mL, standard), essential oils in concentrations of 25, 50, 100, 200, and 400 μg/mL (lanes 4, 5, 6, 7, and 8). *$p < 0.05$ when compared with the negative control group †$p < 0.05$ when compared with the positive control group.

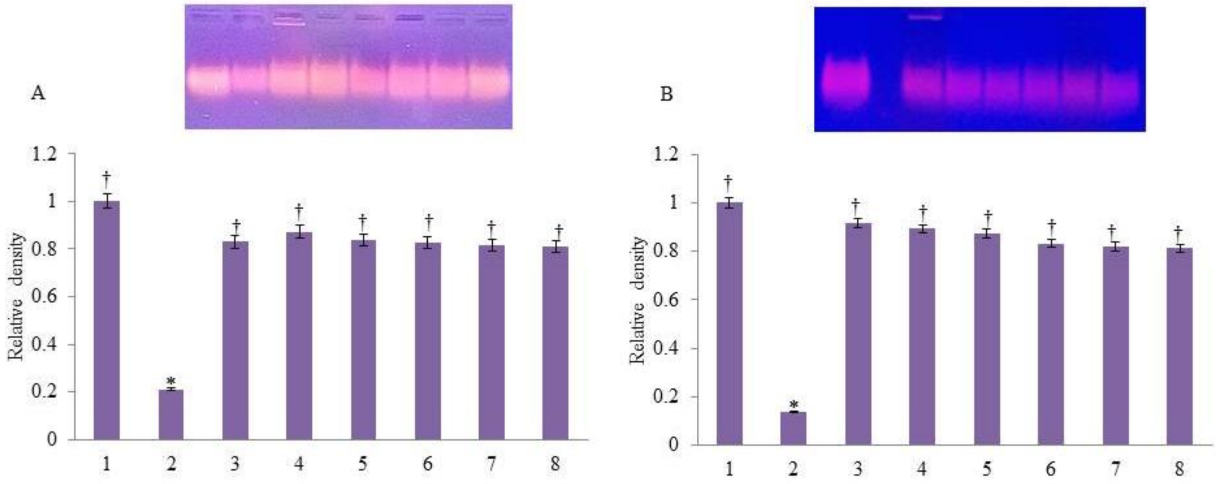

Figure S35. Protective effect of Roman chamomile EO against peroxyl (A) and hydroxyl (B) radicals-induced DNA damage. DNA from salmon sperm (lane 1, negative control), DNA damage control (lane 2, positive control), quercetin (lane 3, 100 μg/mL, standard), essential oils in concentrations of 25, 50, 100, 200, and 400 μg/mL (lanes 4, 5, 6, 7, and 8). *$p < 0.05$ when compared with the negative control group †$p < 0.05$ when compared with the positive control group.

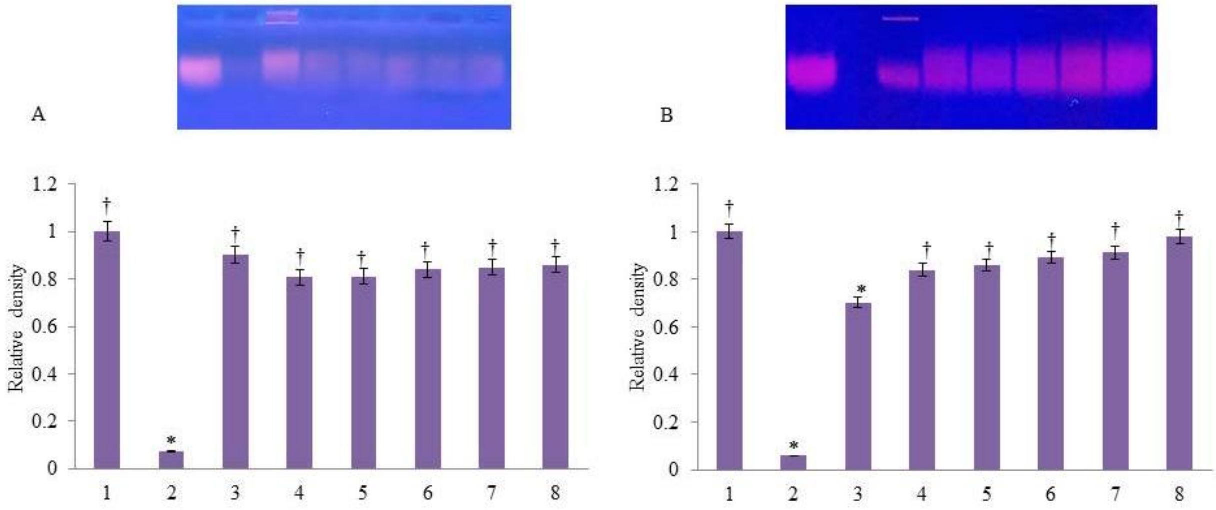

Figure S36. Protective effect of Savory EO against peroxyl (A) and hydroxyl (B) radicals-induced DNA damage. DNA from salmon sperm (lane 1, negative control), DNA damage control (lane 2, positive control), quercetin (lane 3, 100 μg/mL, standard), essential oils in concentrations of 25, 50, 100, 200, and 400 μg/mL (lanes 4, 5, 6, 7, and 8). *$p < 0.05$ when compared with the negative control group †$p < 0.05$ when compared with the positive control group.

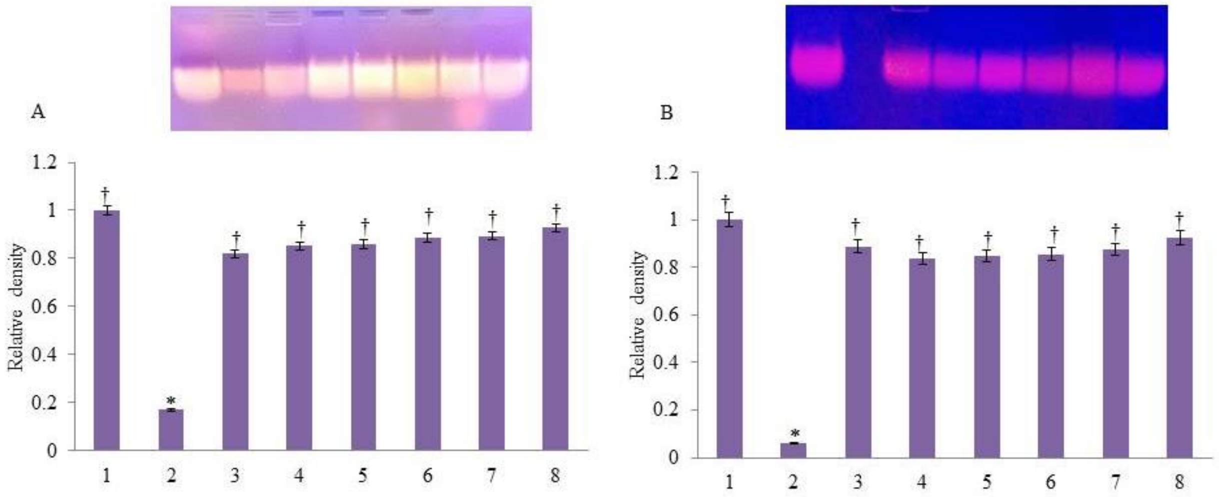

Figure S37. Protective effect of Rosemary EO against peroxyl (A) and hydroxyl (B) radicals-induced DNA damage. DNA from salmon sperm (lane 1, negative control), DNA damage control (lane 2, positive control), quercetin (lane 3, 100 μg/mL, standard), essential oils in concentrations of 25, 50, 100, 200, and 400 μg/mL (lanes 4, 5, 6, 7, and 8). *$p < 0.05$ when compared with the negative control group †$p < 0.05$ when compared with the positive control group.

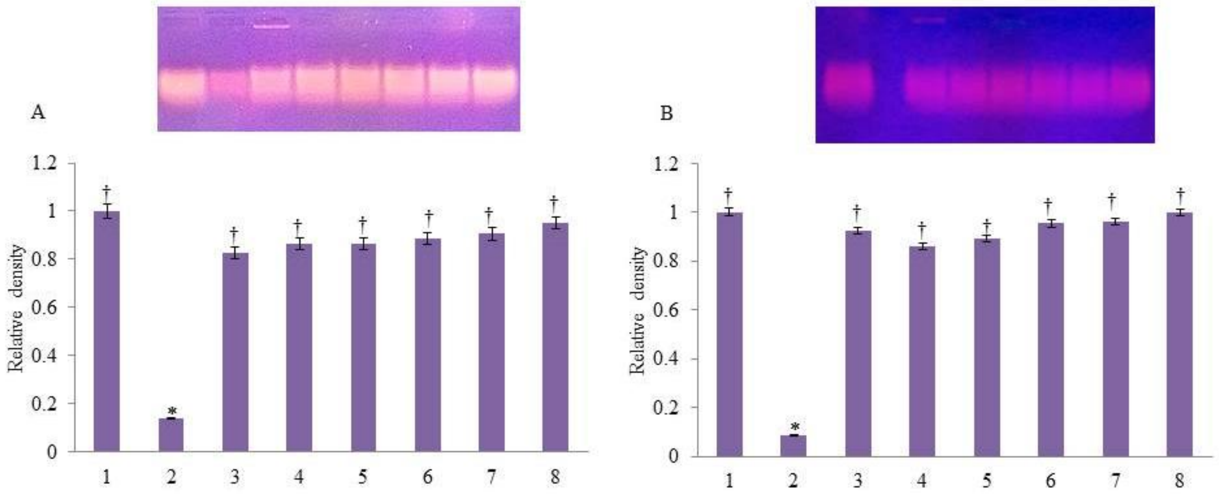

Figure S38. Protective effect of *Eucalyptus globulus* EO against peroxyl (A) and hydroxyl (B) radicals-induced DNA damage. DNA from salmon sperm (lane 1, negative control), DNA damage control (lane 2, positive control), quercetin (lane 3, 100 μg/mL, standard), essential oils in concentrations of 25, 50, 100, 200, and 400 μg/mL (lanes 4, 5, 6, 7, and 8). *$p < 0.05$ when compared with the negative control group †$p < 0.05$ when compared with the positive control group.

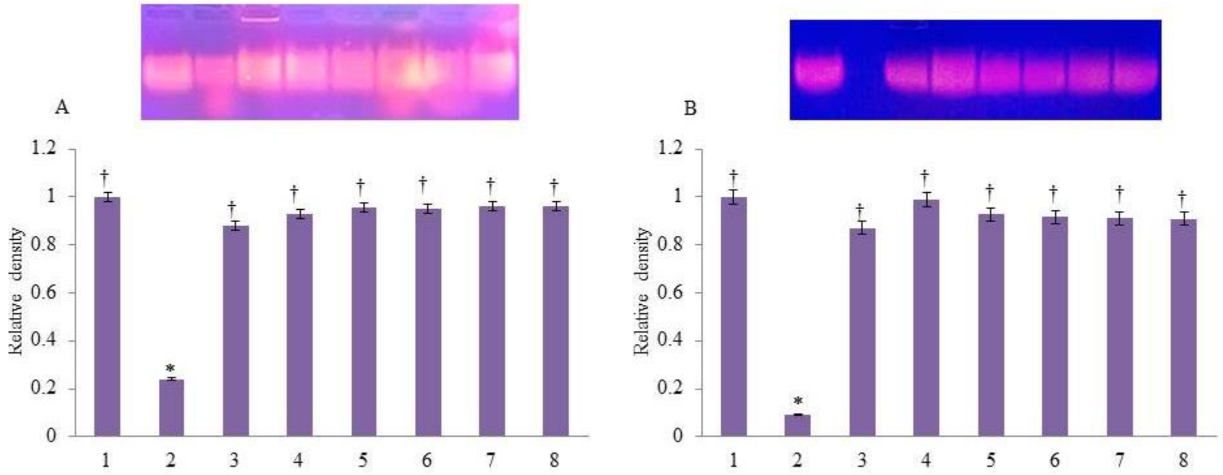

Figure S39. Protective effect of Orange sweet EO against peroxyl (A) and hydroxyl (B) radicals-induced DNA damage. DNA from salmon sperm (lane 1, negative control), DNA damage control (lane 2, positive control), quercetin (lane 3, 100

μg/mL, standard), essential oils in concentrations of 25, 50, 100, 200, and 400 μg/mL (lanes 4, 5, 6, 7, and 8). *$p < 0.05$ when compared with the negative control group †$p < 0.05$ when compared with the positive control group.

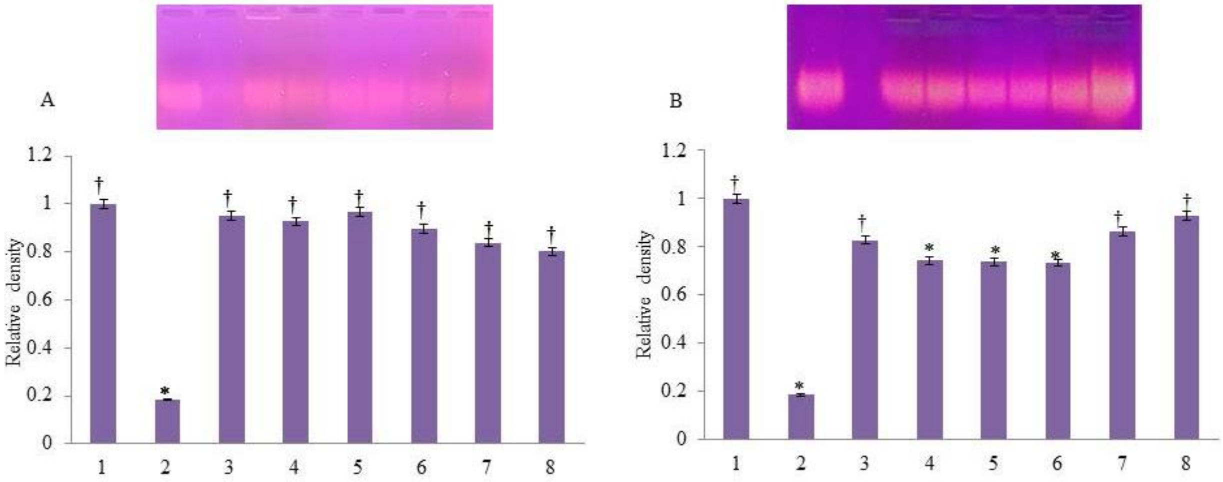

Figure S40. Protective effect of Niaouly EO against peroxyl (A) and hydroxyl (B) radicals-induced DNA damage. DNA from salmon sperm (lane 1, negative control), DNA damage control (lane 2, positive control), quercetin (lane 3, 100 μg/mL, standard), essential oils in concentrations of 25, 50, 100, 200, and 400 μg/mL (lanes 4, 5, 6, 7, and 8). *$p < 0.05$ when compared with the negative control group †$p < 0.05$ when compared with the positive control group.

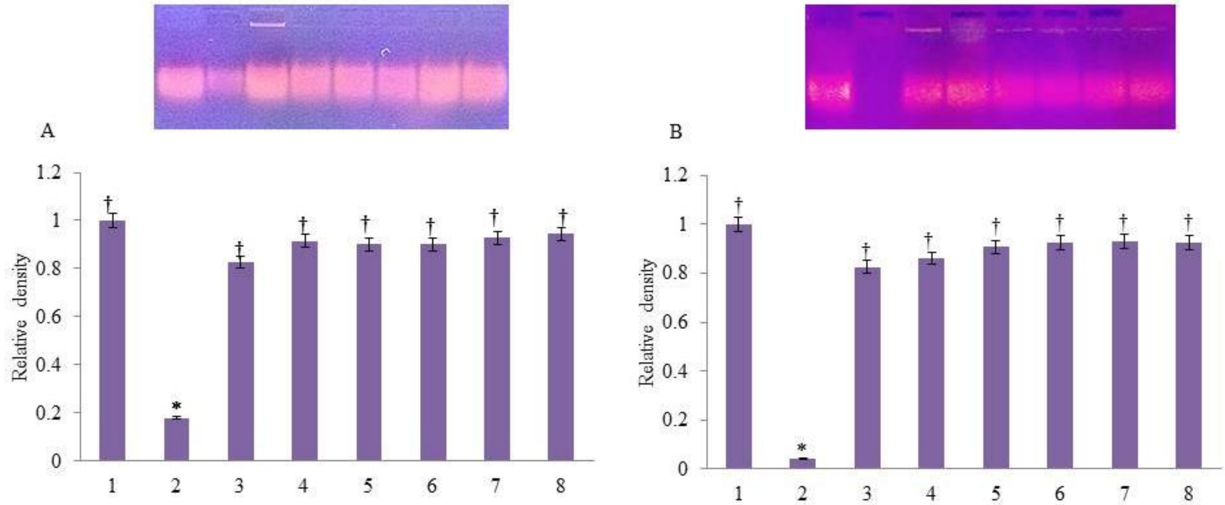

Figure S41. Protective effect of Artemisia EO against peroxyl (A) and hydroxyl (B) radicals-induced DNA damage. DNA from salmon sperm (lane 1, negative control), DNA damage control (lane 2, positive control), quercetin (lane 3, 100 μg/mL, standard), essential oils in concentrations of 25, 50, 100, 200, and 400 μg/mL (lanes 4, 5, 6, 7, and 8). *$p < 0.05$ when compared with the negative control group †$p < 0.05$ when compared with the positive control group.

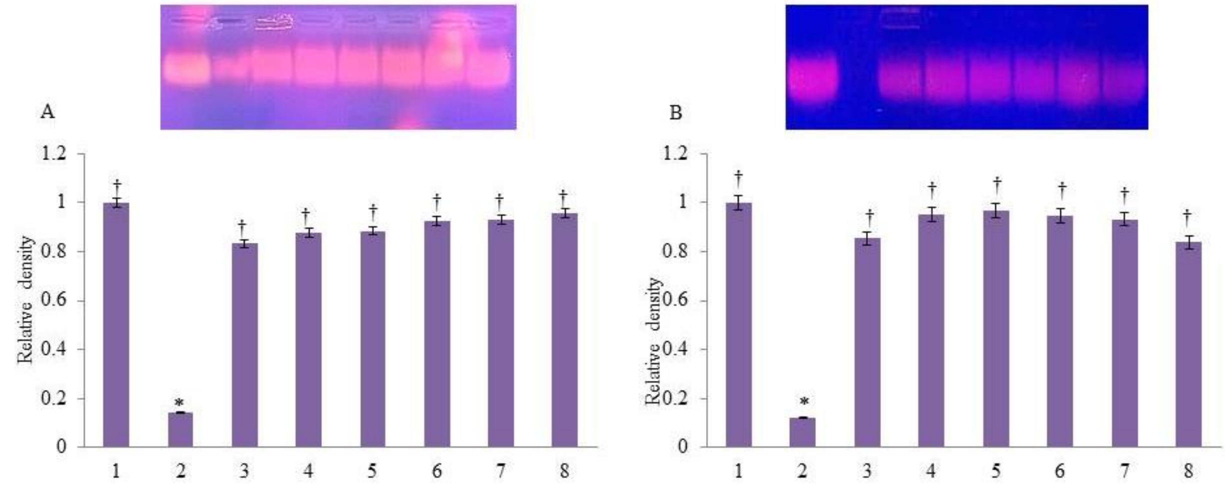

Figure S42. Protective effect of Cajeput EO against peroxyl (A) and hydroxyl (B) radicals-induced DNA damage. DNA from salmon sperm (lane 1, negative control), DNA damage control (lane 2, positive control), quercetin (lane 3, 100 μg/mL, standard), essential oils in concentrations of 25, 50, 100, 200, and 400 μg/mL (lanes 4, 5, 6, 7, and 8). *$p < 0.05$ when compared with the negative control group $^{+}p < 0.05$ when compared with the positive control group.

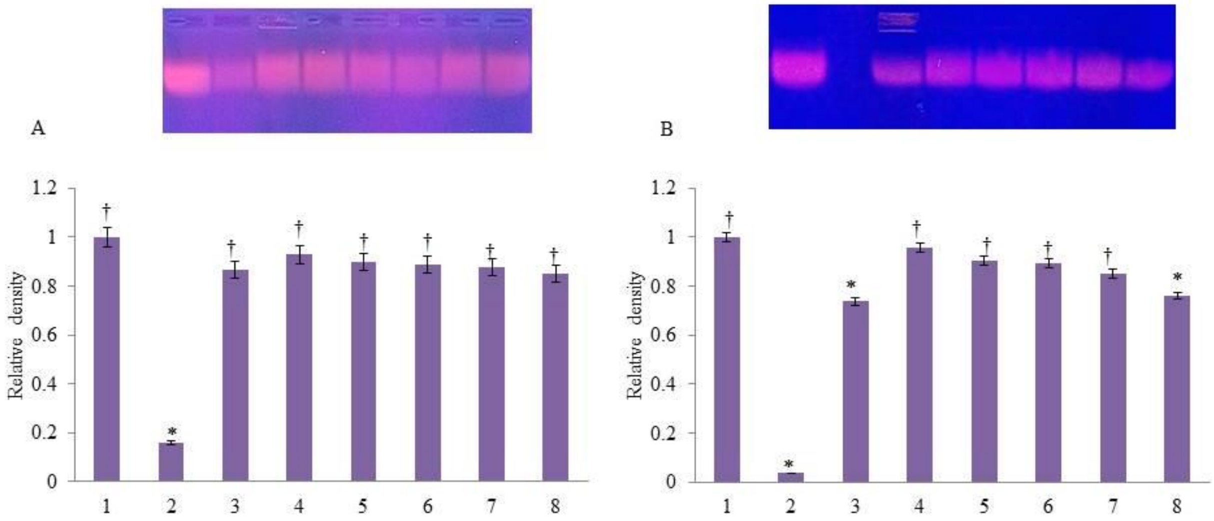

Figure S43. Protective effect of Black pepper EO against peroxyl (A) and hydroxyl (B) radicals-induced DNA damage. DNA from salmon sperm (lane 1, negative control), DNA damage control (lane 2, positive control), quercetin (lane 3, 100 μg/mL, standard), essential oils in concentrations of 25, 50, 100, 200, and 400 μg/mL (lanes 4, 5, 6, 7, and 8). *$p < 0.05$ when compared with the negative control group $^{+}p < 0.05$ when compared with the positive control group.

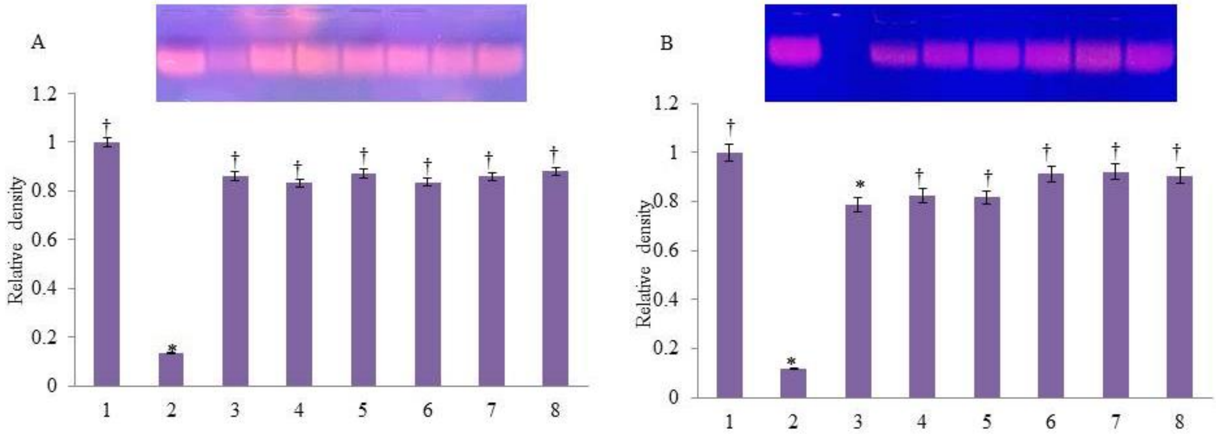

Figure S44. Protective effect of White thyme EO against peroxyl (A) and hydroxyl (B) radicals-induced DNA damage. DNA from salmon sperm (lane 1, negative control), DNA damage control (lane 2, positive control), quercetin (lane 3, 100 μg/mL, standard), essential oils in concentrations of 25, 50, 100, 200, and 400 μg/mL (lanes 4, 5, 6, 7, and 8). *$p < 0.05$ when compared with the negative control group $^{+}p < 0.05$ when compared with the positive control group.

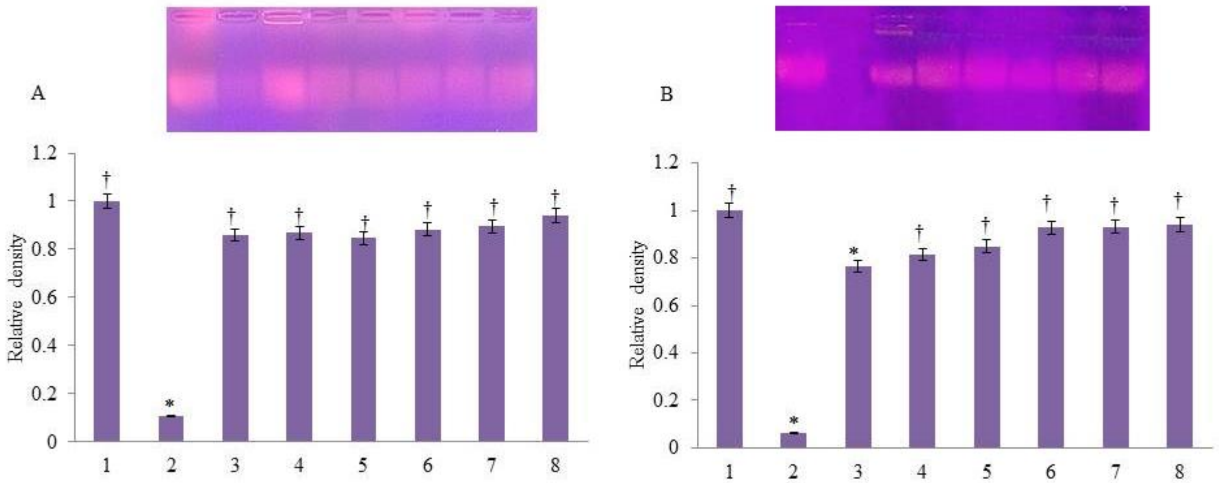

Figure S45. Protective effect of Marjoram EO against peroxyl (A) and hydroxyl (B) radicals-induced DNA damage. DNA from salmon sperm (lane 1, negative control), DNA damage control (lane 2, positive control), quercetin (lane 3, 100 μg/mL, standard), essential oils in concentrations of 25, 50, 100, 200, and 400 μg/mL (lanes 4, 5, 6, 7, and 8). *$p < 0.05$ when compared with the negative control group $^{\dagger}p < 0.05$ when compared with the positive control group.

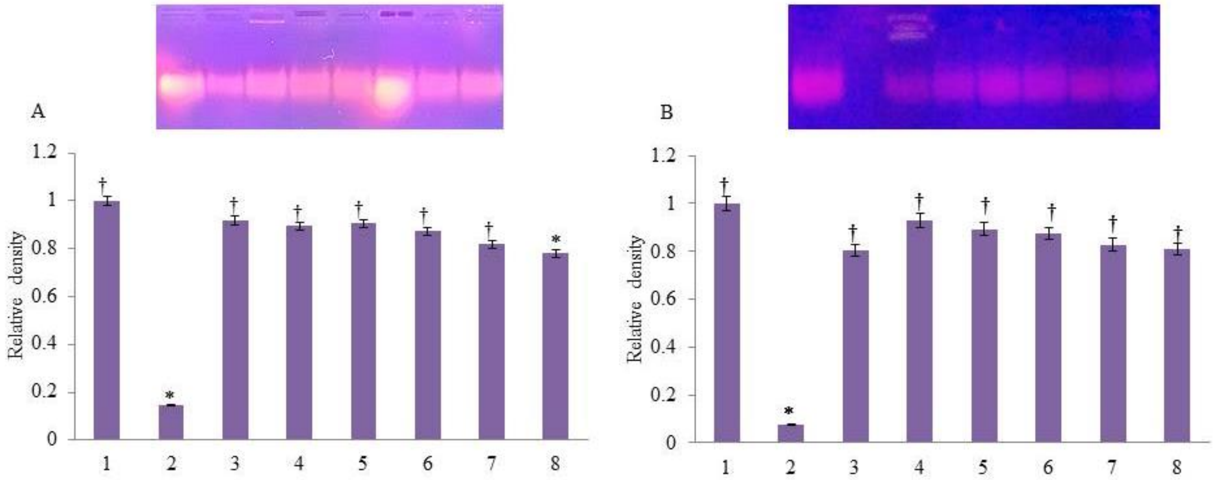

Figure S46. Protective effect of Clove EO against peroxyl (A) and hydroxyl (B) radicals-induced DNA damage. DNA from salmon sperm (lane 1, negative control), DNA damage control (lane 2, positive control), quercetin (lane 3, 100 μg/mL, standard), essential oils in concentrations of 25, 50, 100, 200, and 400 μg/mL (lanes 4, 5, 6, 7, and 8). *$p < 0.05$ when compared with the negative control group $^{\dagger}p < 0.05$ when compared with the positive control group.

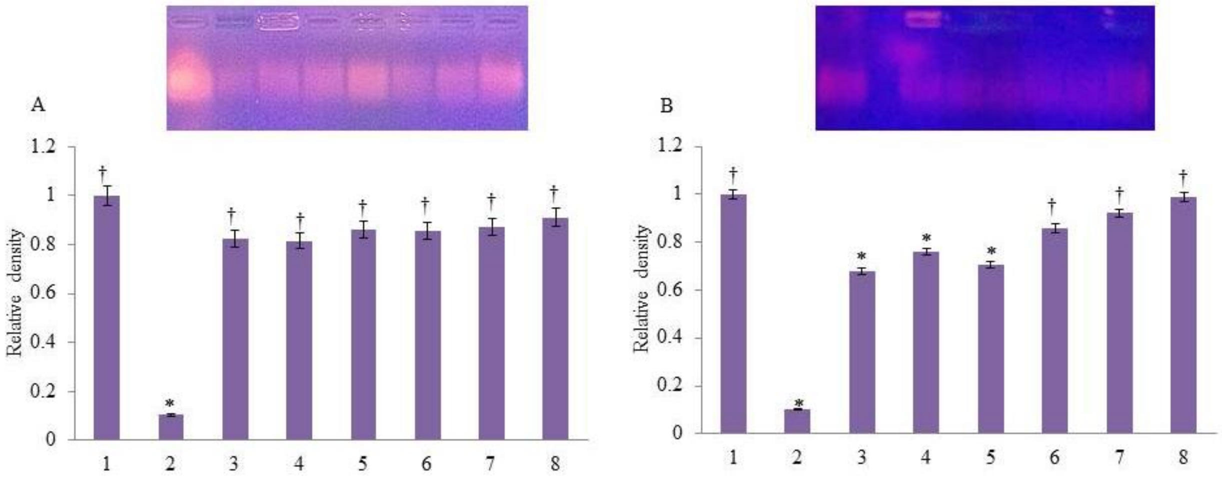

Figure S47. Protective effect of Cypress EO against peroxyl (A) and hydroxyl (B) radicals-induced DNA damage. DNA from salmon sperm (lane 1, negative control), DNA damage control (lane 2, positive control), quercetin (lane 3, 100 μg/mL,

standard), essential oils in concentrations of 25, 50, 100, 200, and 400 μg/mL (lanes 4, 5, 6, 7, and 8). *$p < 0.05$ when compared with the negative control group $^{\dagger}p < 0.05$ when compared with the positive control group.

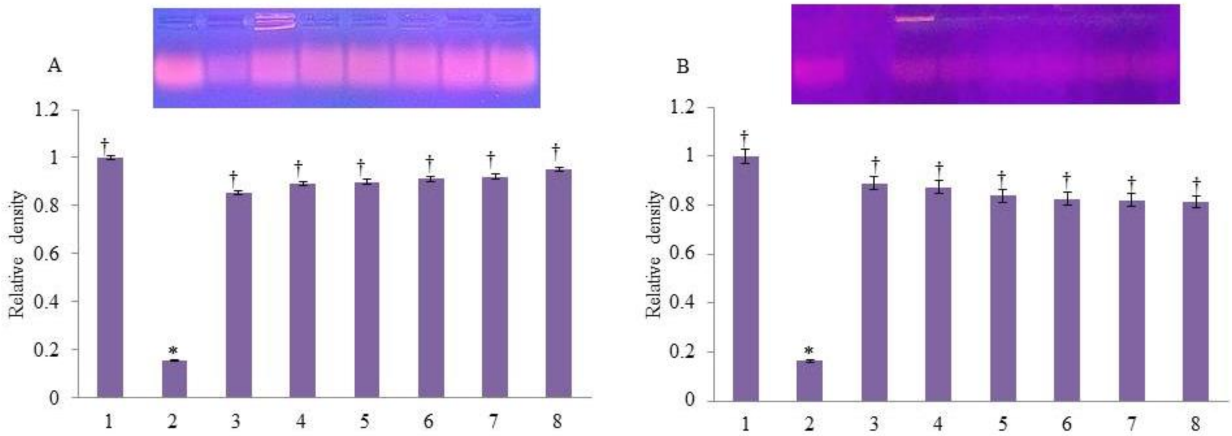

Figure S48. Protective effect of Nutmeg natural EO against peroxyl (A) and hydroxyl (B) radicals-induced DNA damage. DNA from salmon sperm (lane 1, negative control), DNA damage control (lane 2, positive control), quercetin (lane 3, 100 μg/mL, standard), essential oils in concentrations of 25, 50, 100, 200, and 400 μg/mL (lanes 4, 5, 6, 7, and 8). *$p < 0.05$ when compared with the negative control group $^{\dagger}p < 0.05$ when compared with the positive control group.

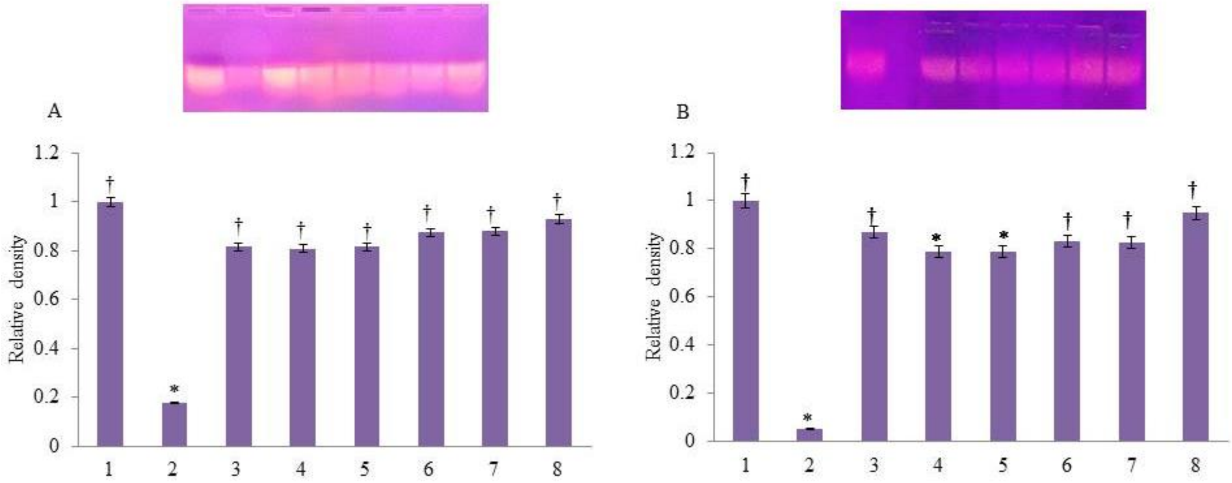

Figure S49. Protective effect of Peppermint EO against peroxyl (A) and hydroxyl (B) radicals-induced DNA damage. DNA from salmon sperm (lane 1, negative control), DNA damage control (lane 2, positive control), quercetin (lane 3, 100 μg/mL, standard), essential oils in concentrations of 25, 50, 100, 200, and 400 μg/mL (lanes 4, 5, 6, 7, and 8). *$p < 0.05$ when compared with the negative control group $^{\dagger}p < 0.05$ when compared with the positive control group.

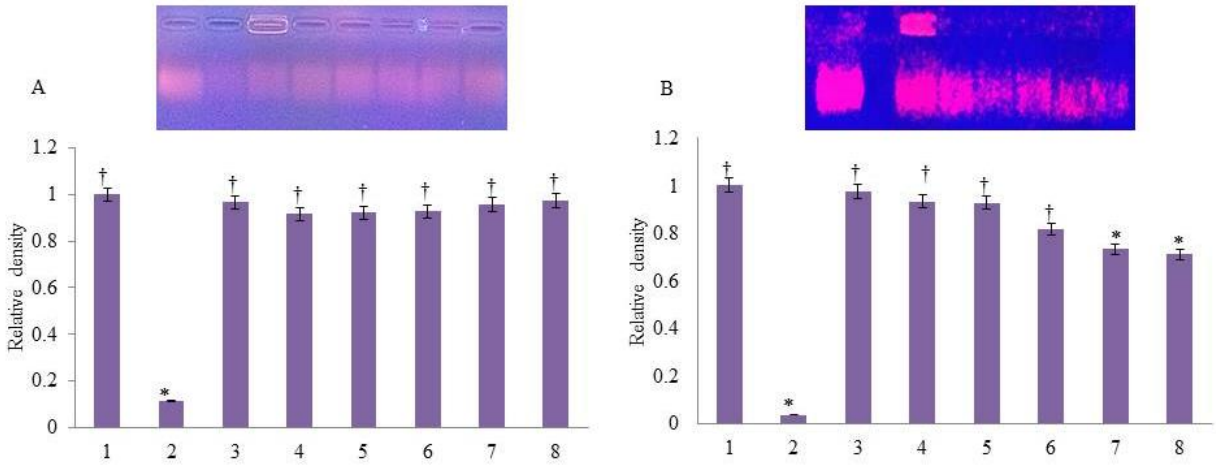

Figure S50. Protective effect of Verbena EO against peroxyl (A) and hydroxyl (B) radicals-induced DNA damage. DNA from salmon sperm (lane 1, negative control), DNA damage control (lane 2, positive control), quercetin (lane 3, 100 μg/mL,

standard), essential oils in concentrations of 25, 50, 100, 200, and 400 µg/mL (lanes 4, 5, 6, 7, and 8). *$p < 0.05$ when compared with the negative control group $^{\dagger}p < 0.05$ when compared with the positive control group.

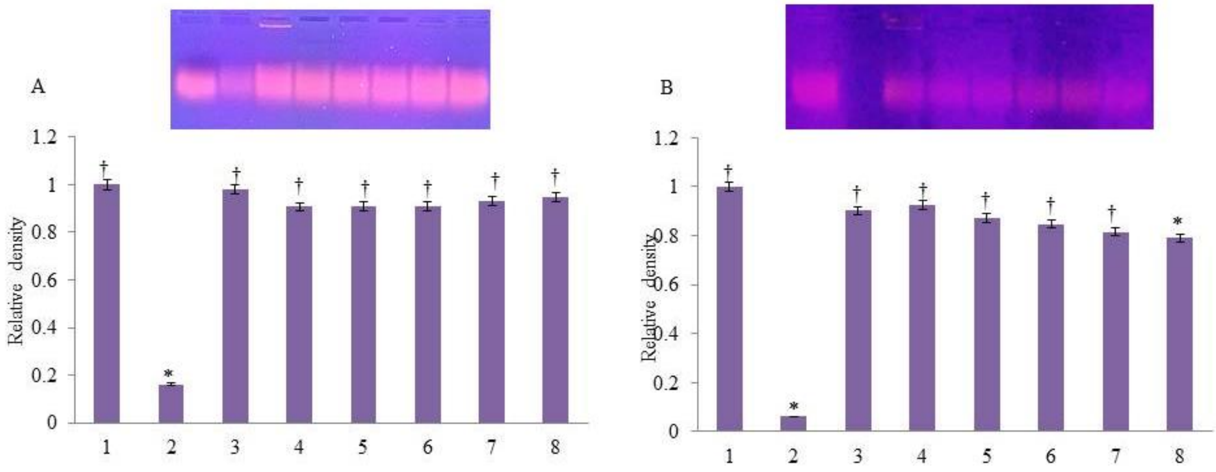

Figure S51. Protective effect of Basil EO against peroxyl (A) and hydroxyl (B) radicals-induced DNA damage. DNA from salmon sperm (lane 1, negative control), DNA damage control (lane 2, positive control), quercetin (lane 3, 100 µg/mL, standard), essential oils in concentrations of 25, 50, 100, 200, and 400 µg/mL (lanes 4, 5, 6, 7, and 8). *$p < 0.05$ when compared with the negative control group $^{\dagger}p < 0.05$ when compared with the positive control group.

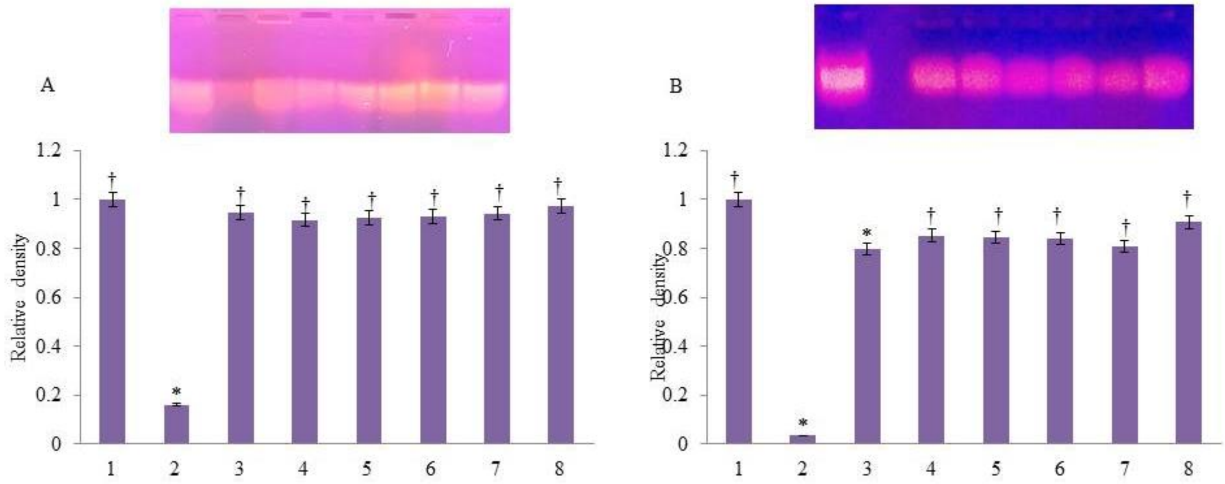

Figure S52. Protective effect of Palmarosa EO against peroxyl (A) and hydroxyl (B) radicals-induced DNA damage. DNA from salmon sperm (lane 1, negative control), DNA damage control (lane 2, positive control), quercetin (lane 3, 100 µg/mL, standard), essential oils in concentrations of 25, 50, 100, 200, and 400 µg/mL (lanes 4, 5, 6, 7, and 8). *$p < 0.05$ when compared with the negative control group $^{\dagger}p < 0.05$ when compared with the positive control group.

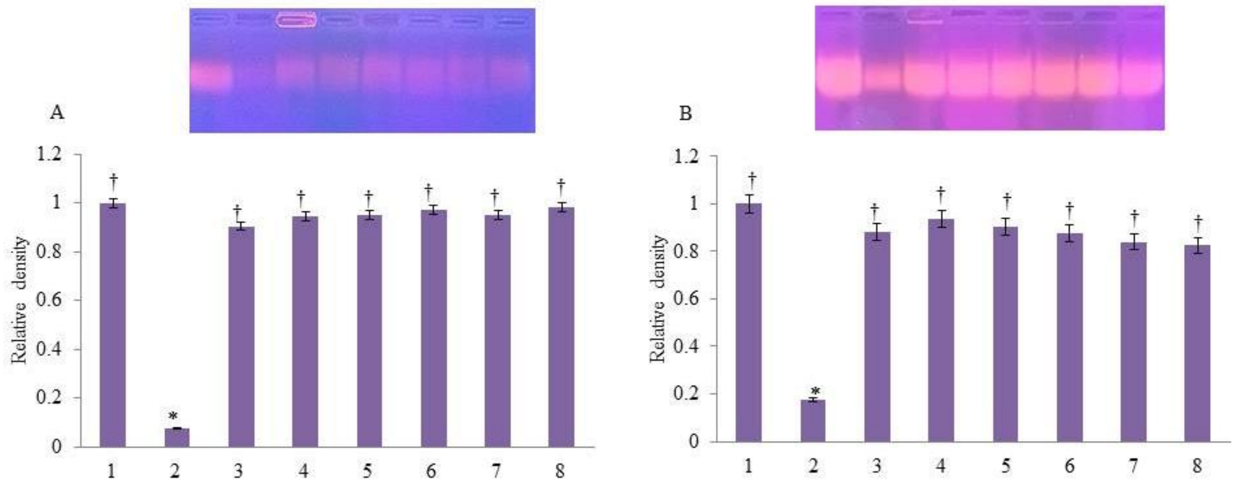

Figure S53. Protective effect of Laurel EO against peroxyl (A) and hydroxyl (B) radicals-induced DNA damage. DNA from salmon sperm (lane 1, negative control), DNA damage control (lane 2, positive control), quercetin (lane 3, 100 µg/mL,

standard), essential oils in concentrations of 25, 50, 100, 200, and 400 μg/mL (lanes 4, 5, 6, 7, and 8). *$p < 0.05$ when compared with the negative control group †$p < 0.05$ when compared with the positive control group.

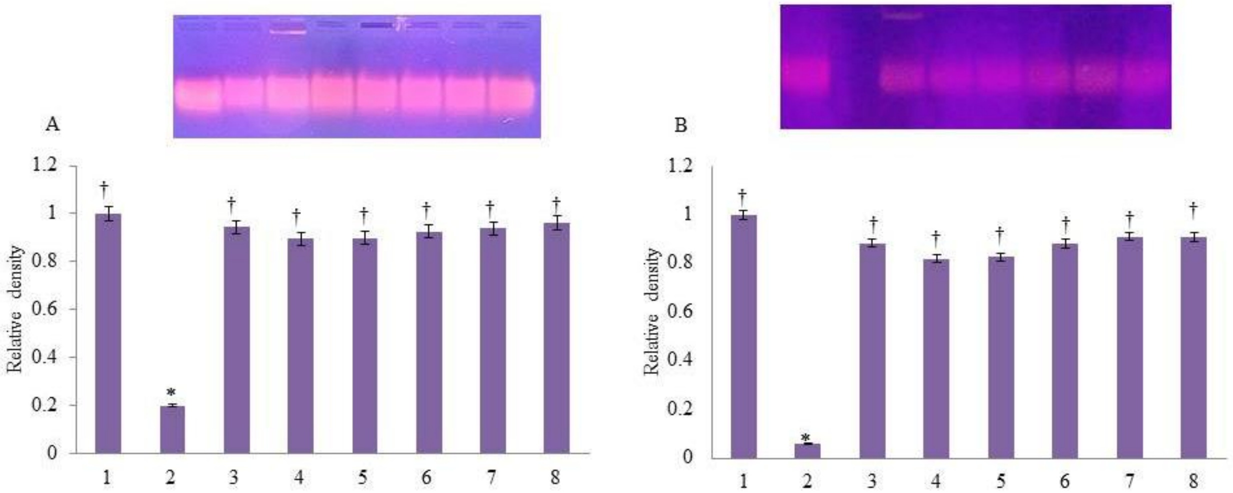

Figure S54. Protective effect of Narural anise pure EO against peroxyl (A) and hydroxyl (B) radicals-induced DNA damage. DNA from salmon sperm (lane 1, negative control), DNA damage control (lane 2, positive control), quercetin (lane 3, 100 μg/mL, standard), essential oils in concentrations of 25, 50, 100, 200, and 400 μg/mL (lanes 4, 5, 6, 7, and 8). *$p < 0.05$ when compared with the negative control group †$p < 0.05$ when compared with the positive control group.

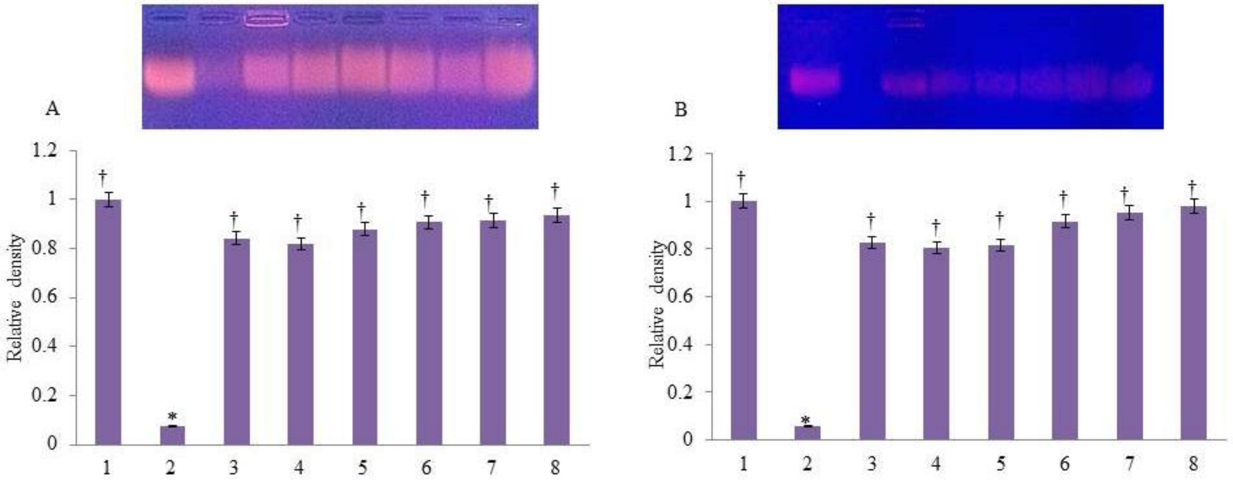

Figure S55. Protective effect of Incense EO against peroxyl (A) and hydroxyl (B) radicals-induced DNA damage. DNA from salmon sperm (lane 1, negative control), DNA damage control (lane 2, positive control), quercetin (lane 3, 100 μg/mL, standard), essential oils in concentrations of 25, 50, 100, 200, and 400 μg/mL (lanes 4, 5, 6, 7, and 8). *$p < 0.05$ when compared with the negative control group †$p < 0.05$ when compared with the positive control group.

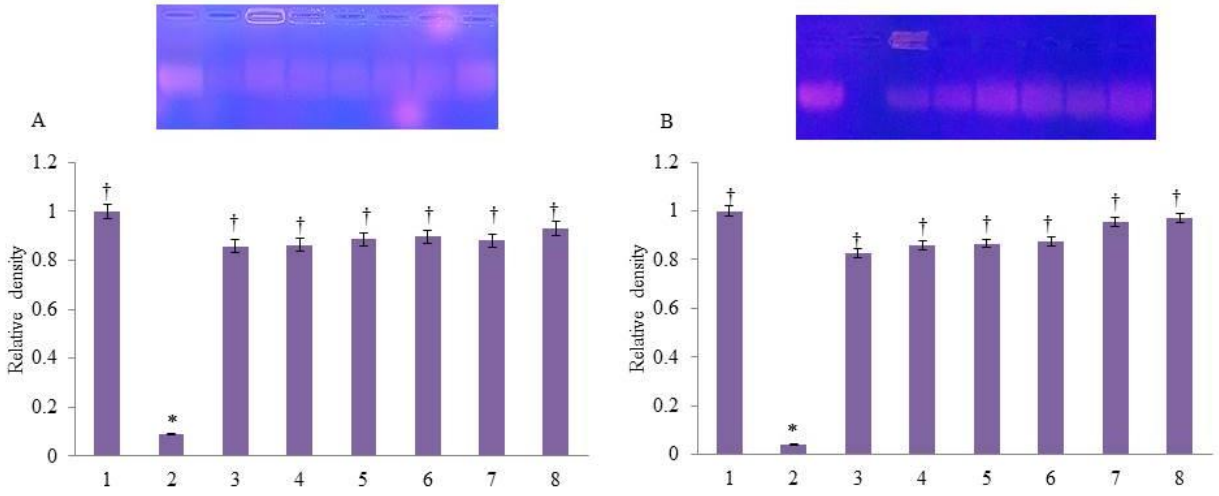

Figure S56. Protective effect of *Mentha suaveolens* EO against peroxyl (A) and hydroxyl (B) radicals-induced DNA damage. DNA from salmon sperm (lane 1, negative control), DNA damage control (lane 2, positive control), quercetin (lane 3, 100 µg/mL, standard), essential oils in concentrations of 25, 50, 100, 200, and 400 µg/mL (lanes 4, 5, 6, 7, and 8). *p < 0.05 when compared with the negative control group †p < 0.05 when compared with the positive control group.

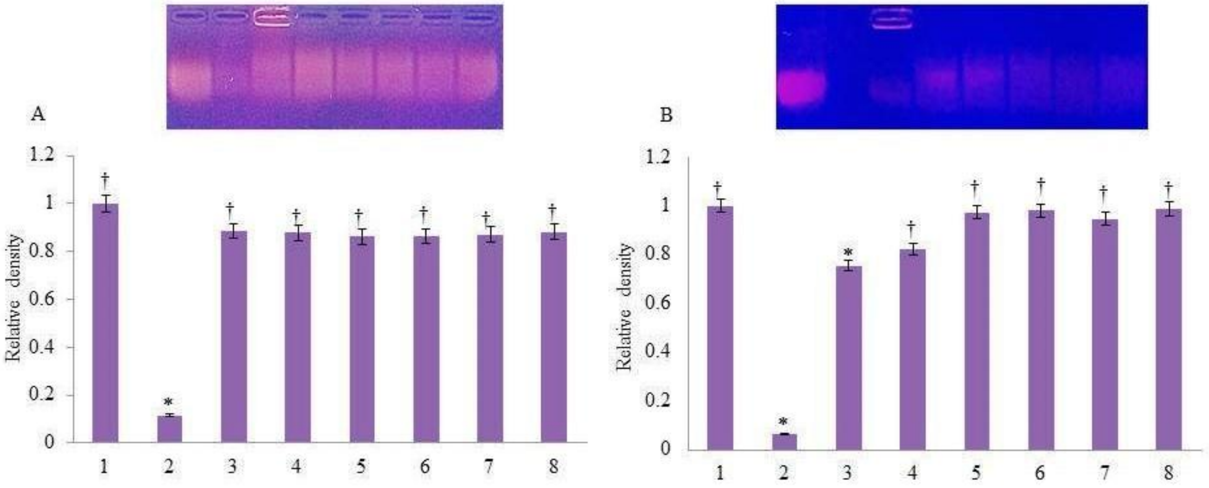

Figure S57. Protective effect of *Coridotthymus capitatus* EO against peroxyl (A) and hydroxyl (B) radicals-induced DNA damage. DNA from salmon sperm (lane 1, negative control), DNA damage control (lane 2, positive control), quercetin (lane 3, 100 µg/mL, standard), essential oils in concentrations of 25, 50, 100, 200, and 400 µg/mL (lanes 4, 5, 6, 7, and 8). *p < 0.05 when compared with the negative control group †p < 0.05 when compared with the positive control group.

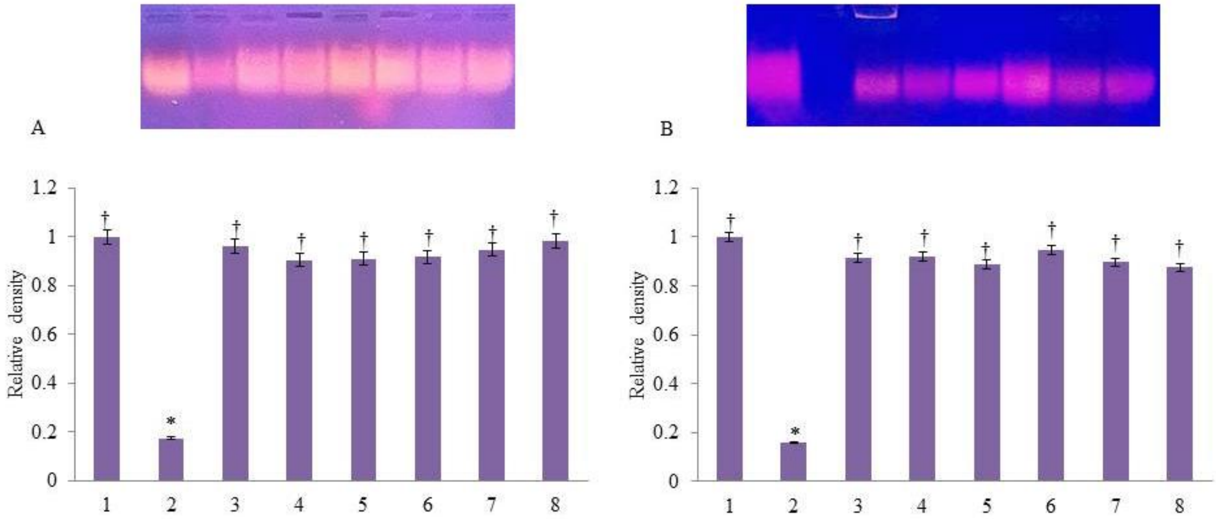

Figure S58. Protective effect of *Thymus vulgaris* EO against peroxyl (A) and hydroxyl (B) radicals-induced DNA damage. DNA from salmon sperm (lane 1, negative control), DNA damage control (lane 2, positive control), quercetin (lane 3, 100 µg/mL, standard), essential oils in concentrations of 25, 50, 100, 200, and 400 µg/mL (lanes 4, 5, 6, 7, and 8). *p < 0.05 when compared with the negative control group †p < 0.05 when compared with the positive control group.

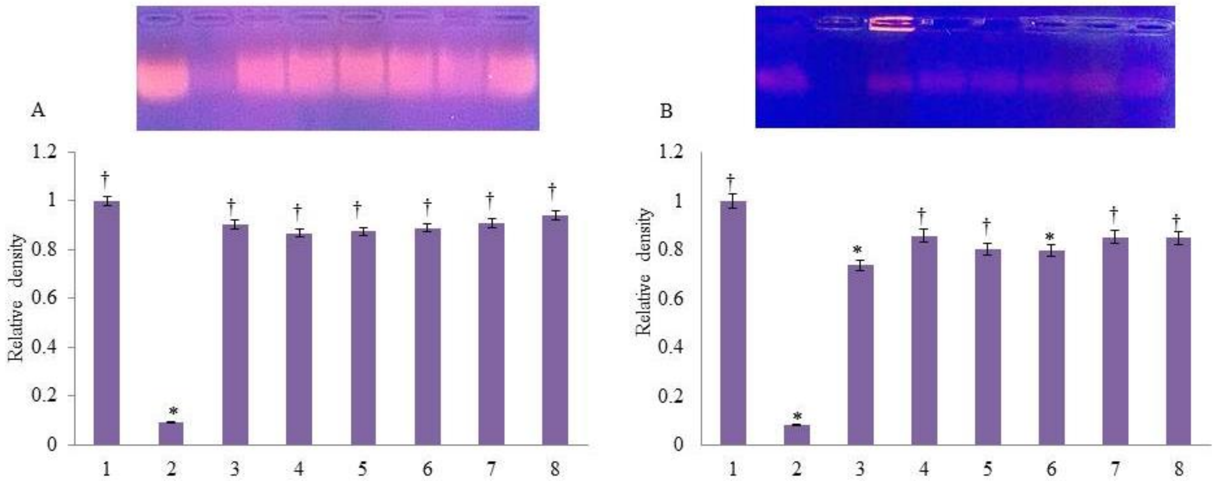

Figure S59. Protective effect of *Origanum hirtum* EO against peroxyl (A) and hydroxyl (B) radicals-induced DNA damage. DNA from salmon sperm (lane 1, negative control), DNA damage control (lane 2, positive control), quercetin (lane 3, 100 μg/mL, standard), essential oils in concentrations of 25, 50, 100, 200, and 400 μg/mL (lanes 4, 5, 6, 7, and 8). *$p < 0.05$ when compared with the negative control group †$p < 0.05$ when compared with the positive control group.

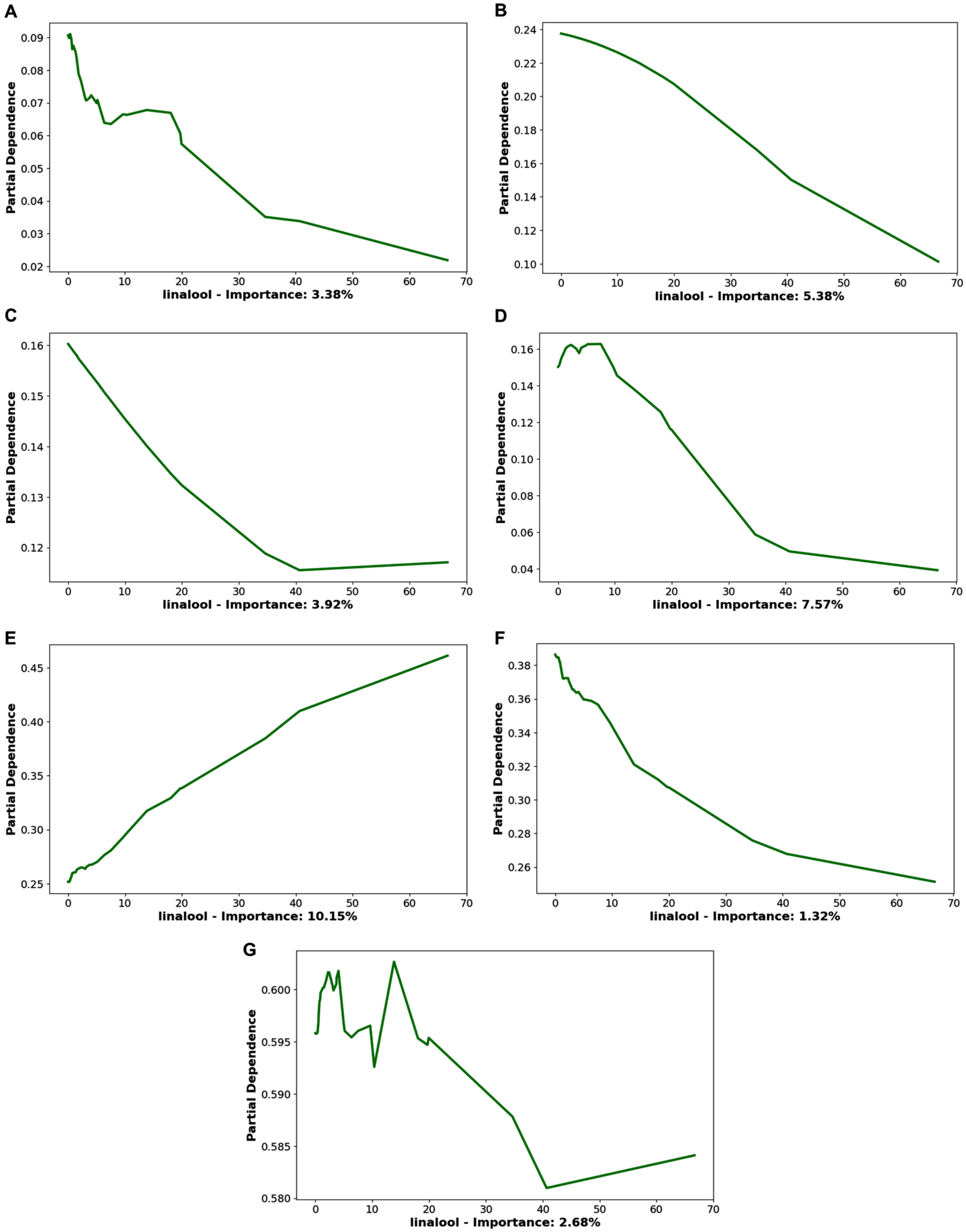

Figure S60. Partial dependence graphs of **limonene** in the model of M^{n+} (A), DPPH$^{\bullet}$ (B), LOO$^{\bullet}$ (C), ABTS$^{\bullet+}$ (D), OH$^{\bullet}$ (E), ROO-RBD$_{50}$ (F), HO-RBD$_{50}$ (G).

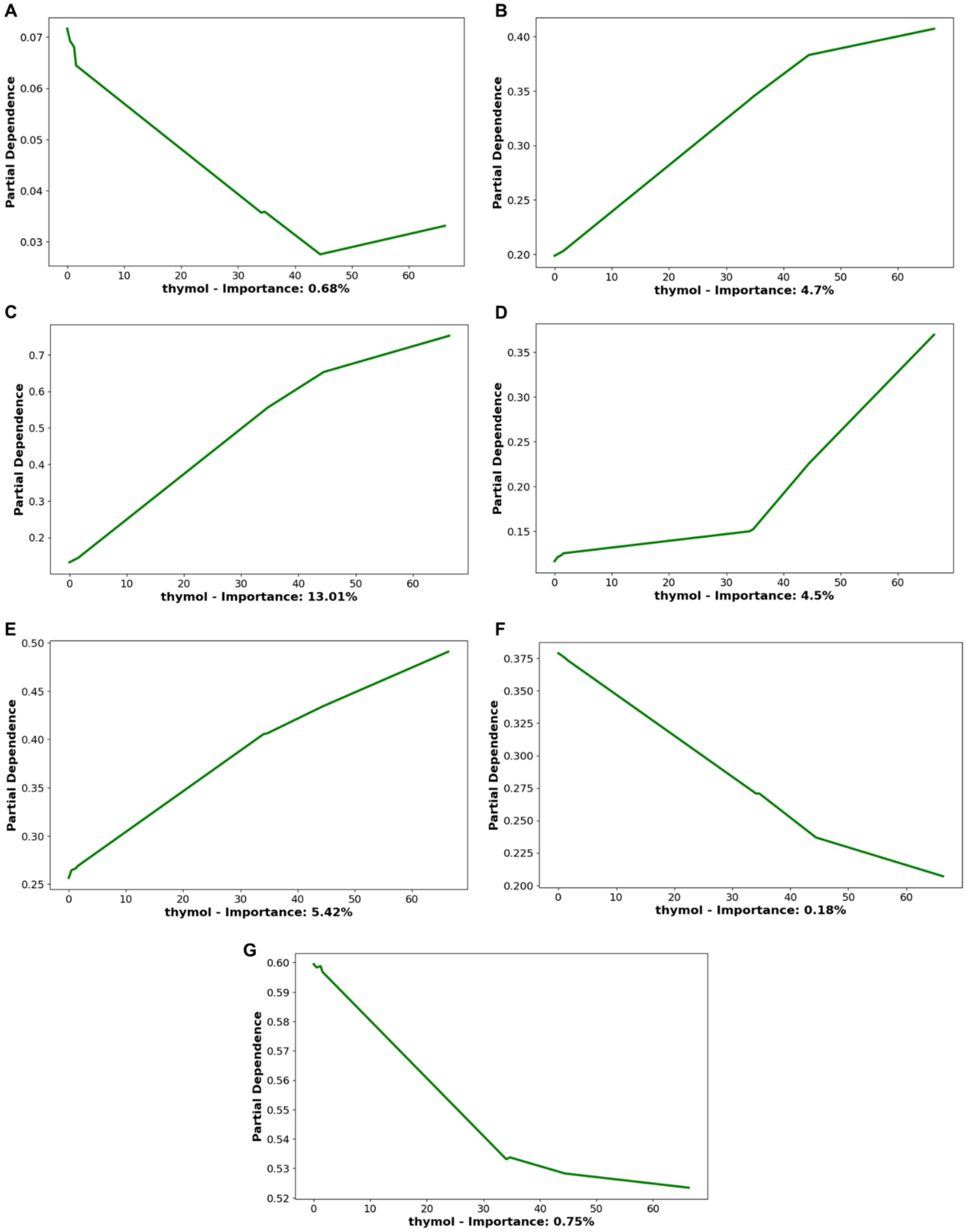

Figure S61. Partial dependence graphs of **thymol** in the model of M^{n+} (A), DPPH$^{\bullet}$ (B), LOO$^{\bullet}$ (C), ABTS$^{\bullet +}$ (D), OH$^{\bullet}$ (E), ROO-RBD$_{50}$ (F), HO-RBD$_{50}$ (G).

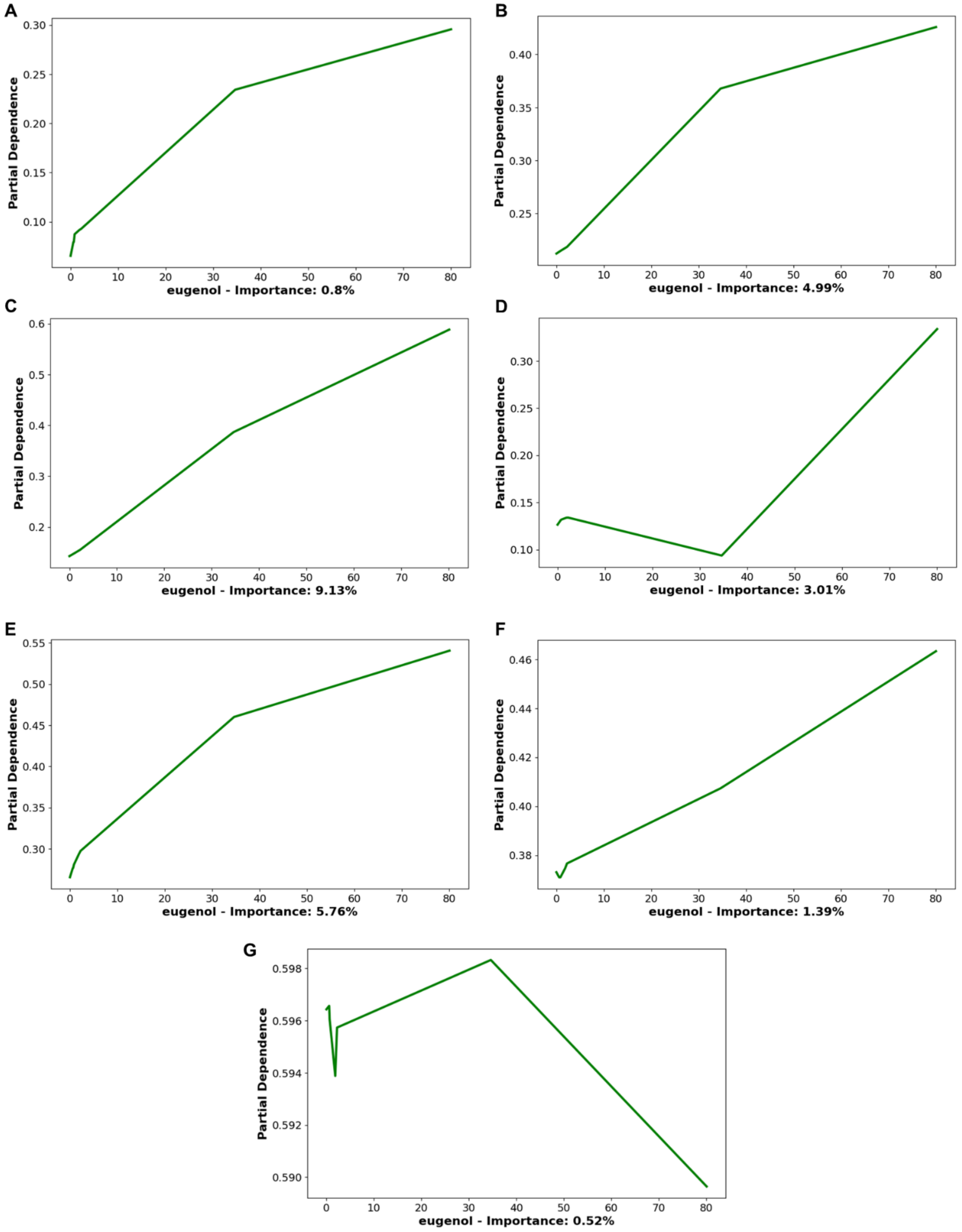

Figure S62. Partial dependence graphs of **eugenol** in the model of M^{n+} (A), DPPH$^{•}$ (B), LOO$^{•}$ (C), ABTS$^{•+}$ (D), OH$^{•}$ (E), ROO-RBD$_{50}$ (F), HO-RBD$_{50}$ (G).

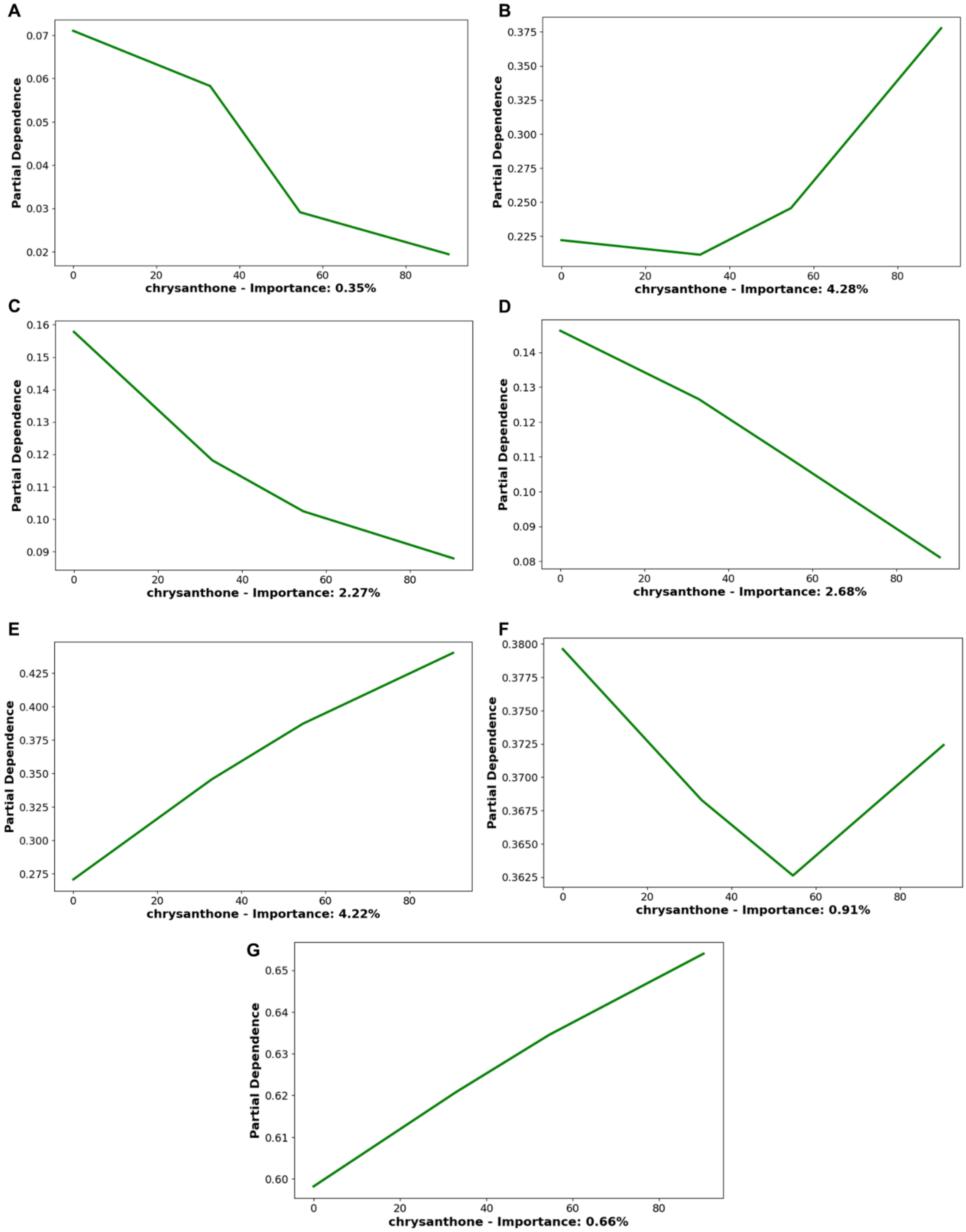

Figure S63. Partial dependence graphs of **chrysanthone** in the model of M^{n+} (A), DPPH$^{\bullet}$ (B), LOO$^{\bullet}$ (C), ABTS$^{\bullet+}$ (D), OH$^{\bullet}$ (E), ROO-RBD$_{50}$ (F), HO-RBD$_{50}$ (G).

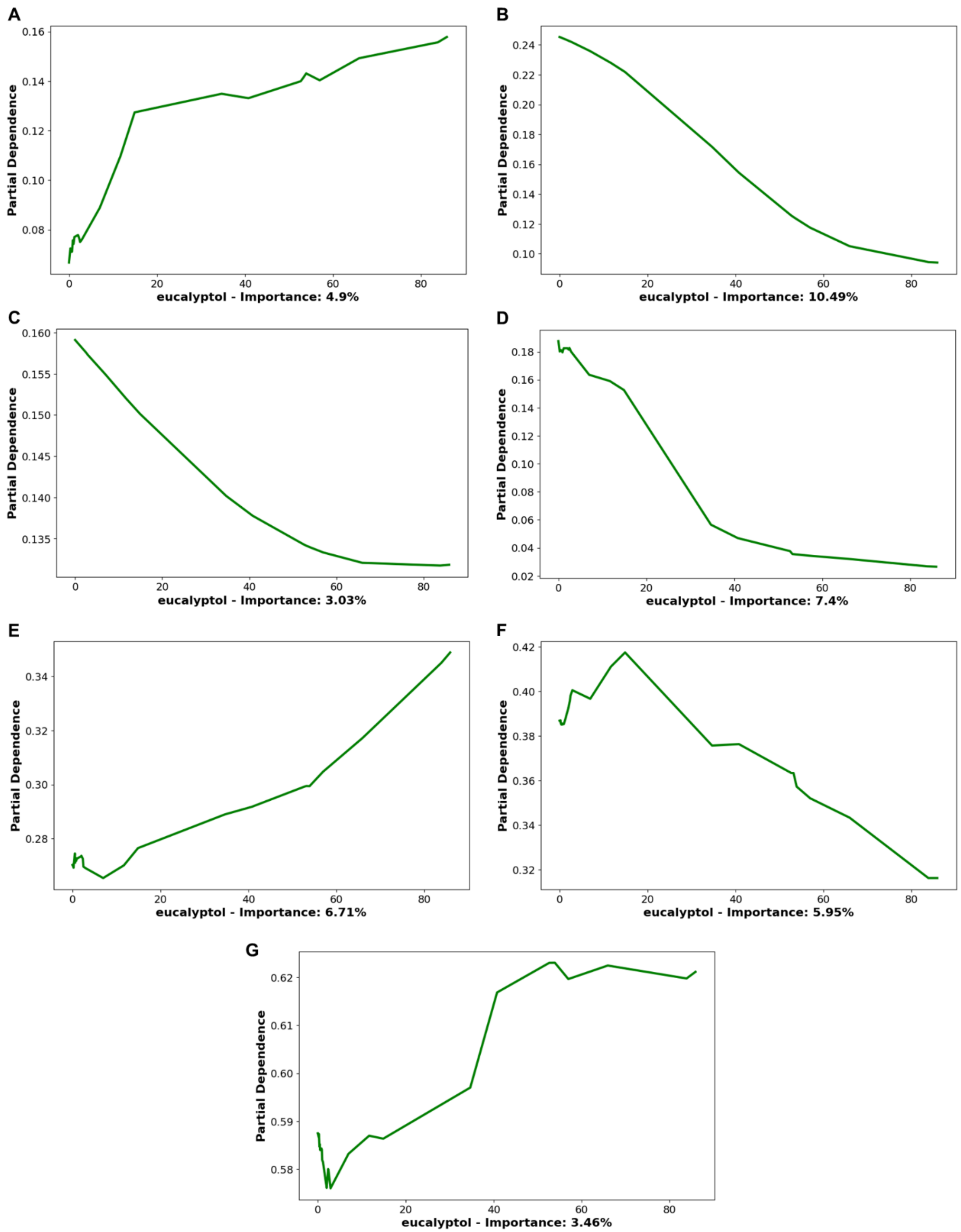

Figure S64. Partial dependence graphs of **chrysanthone** in the model of M^{n+} (A), DPPH$^{\bullet}$ (B), LOO$^{\bullet}$ (C), ABTS$^{\bullet+}$ (D), OH$^{\bullet}$ (E), ROO-RBD$_{50}$ (F), HO-RBD$_{50}$ (G).

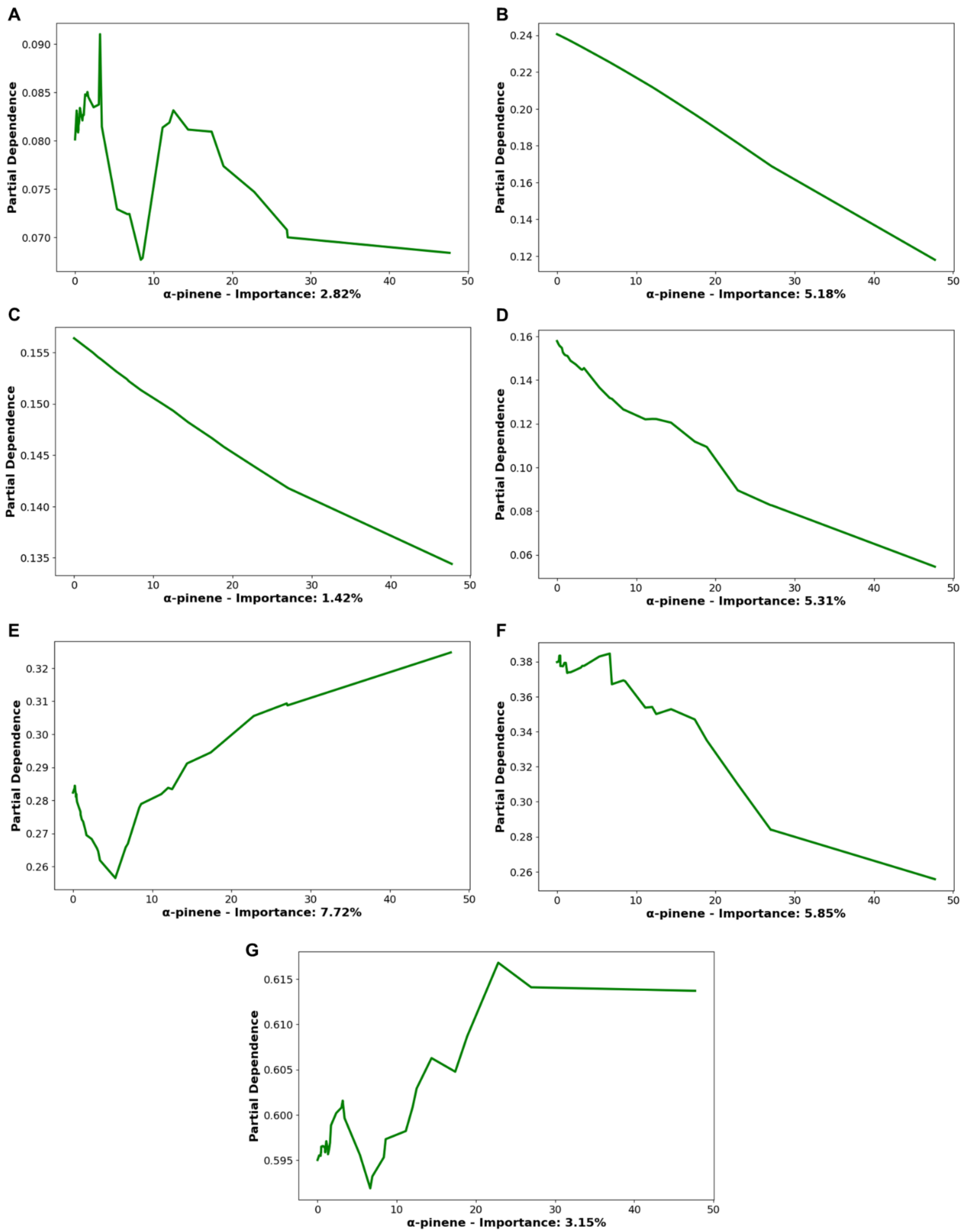

Figure S65. Partial dependence graphs of **α-pinene** in the model of M^{n+} (A), DPPH$^{\bullet}$ (B), LOO$^{\bullet}$ (C), ABTS$^{\bullet+}$ (D), OH$^{\bullet}$ (E), ROO-RBD$_{50}$ (F), HO-RBD$_{50}$ (G).

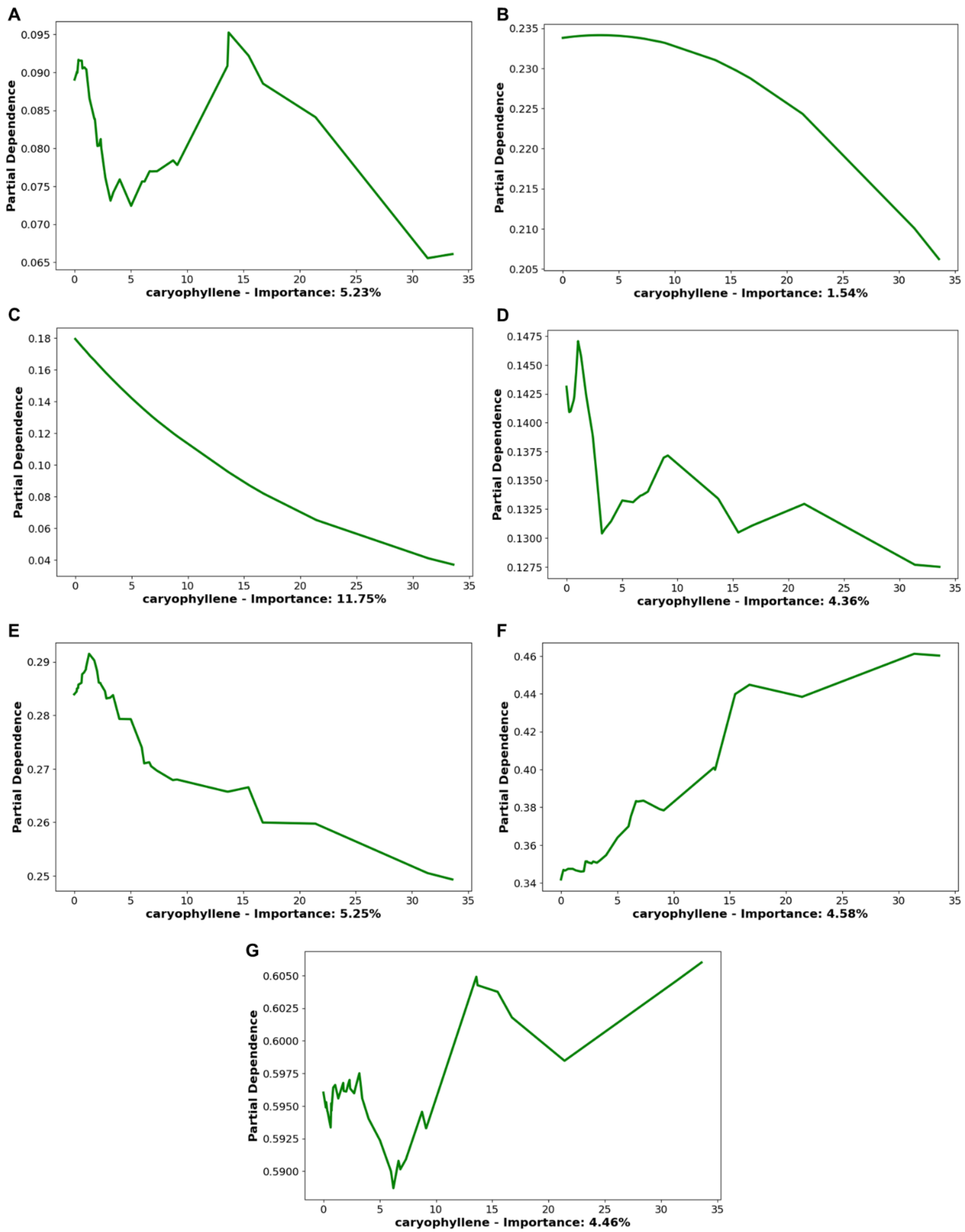

Figure S66. Partial dependence graphs of **caryophillene** in the model of M^{n+} (A), $DPPH^{\bullet}$ (B), $LOO^{\bullet}$ (C), $ABTS^{\bullet +}$ (D), $OH^{\bullet}$ (E), ROO-RBD_{50} (F), HO-RBD_{50} (G).

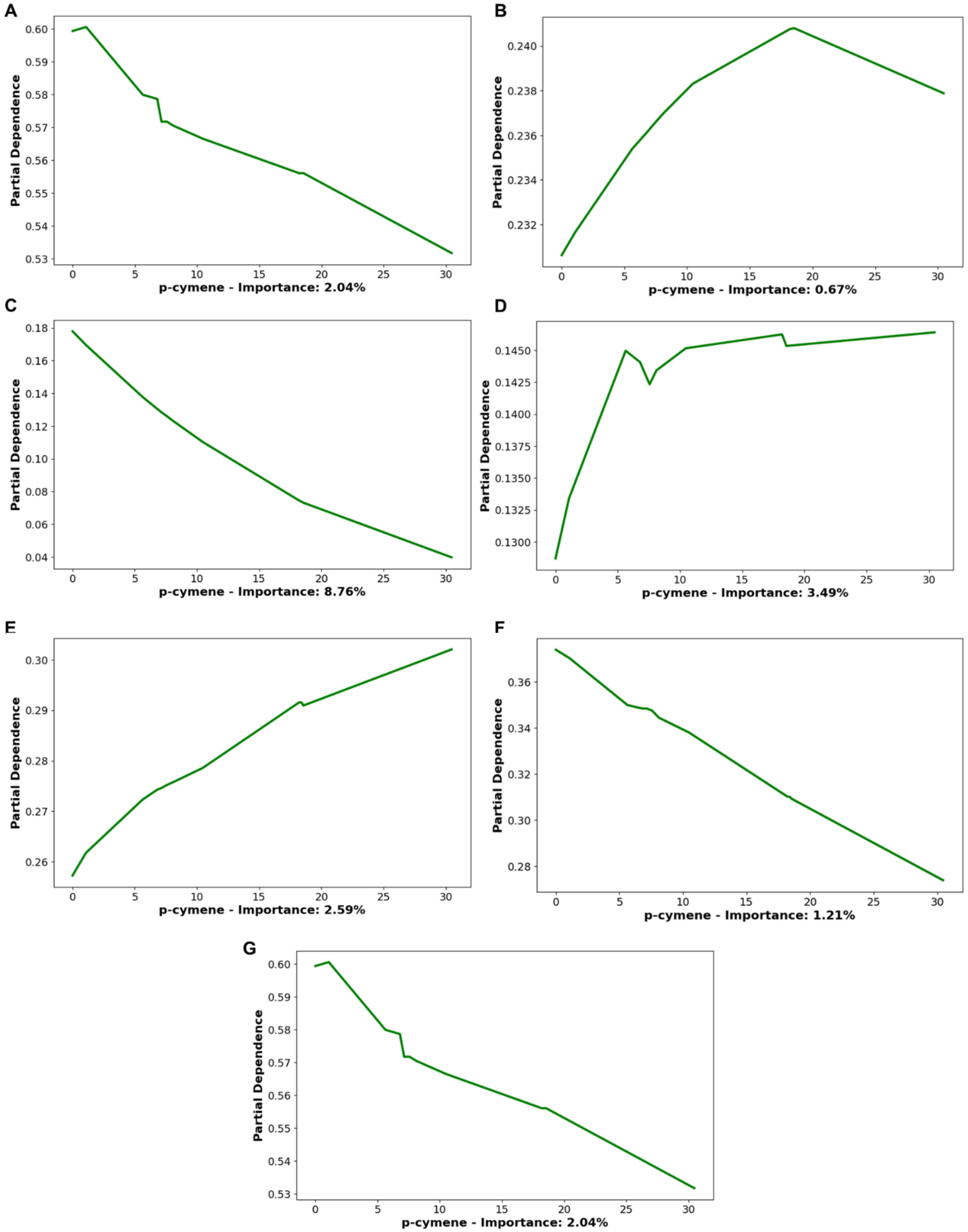

Figure S67. Partial dependence graphs of ***p*-cymene** in the model of M^{n+} (A), DPPH$^{\bullet}$ (B), LOO$^{\bullet}$ (C), ABTS$^{\bullet+}$ (D), OH$^{\bullet}$ (E), ROO-RBD$_{50}$ (F), HO-RBD$_{50}$ (G).

Results

ABTS cation radical neutralizing activity of EOs

According to the overall EC_{50} concentration range against $ABTS^{+\bullet}$, one may presume that EOs were generally more potent against $ABTS^{+\bullet}$ related to $DPPH^{\bullet}$ and $LOO^{\bullet}$. Indeed, even fourteen EOs, namely Red thyme, Oregano, *Coridotthymus capitatus*, Savory, Cedar leaves, *Origanum hirtum*, Rosemary, White thyme , *Mentha suaveolens, Thymus vulgaris, Origanum hirtum*, Lemongrass, Clove, and Birch EOs showed 76.67- (Red thyme) to 0.18-fold (Birch) better affinity for $ABTS^{+\bullet}$ (and subsequently for $OH^{\bullet}$), whereas only four of them, *viz*. Ylang-ylang , Cade, CCP, and Laurel EOs were more potent against $DPPH^{\bullet}$ (and thus for $LOO^{\bullet}$). Moreover, upon assigning the Laurel EO and *Mentha suaveolens* EO missing EC_{50}s equal to 1 μg/mL, a correlation $[EC_{50}]$ $ABTS^{+\bullet}$ = 0.9284 x $[EC_{50}]$ $DPPH^{\bullet}$ - 0.0236 (r^2 = 0.8467) was obtained, even though the neutralizations of $DPPH^{\bullet}$ and $ABTS^{+\bullet}$ are mechanistically different.

On the other hand, a brief analysis of EOs $ABTS^{+\bullet}/LOO^{\bullet}$ relations revealed that an even number of EOs had a higher affinity for $ABTS^{+\bullet}$ than for $LOO^{\bullet}$, and *vice versa.* Hence, nine EOs, namely Birch, *Mentha suaveolens*, Oregano, Clove, Cedar Leaves, *Origanum hirtum*, White thyme, Red thyme, and *Coridotthymus capitatus* EOs showed 5.71- (Birch EO) to 0.39-fold (*Coridotthymus capitatus* EO) better affinity for $ABTS^{+\bullet}$, whereas nine EOs, namely Cade, Ceylon cinnamon peel, Lemongrass, *Thymus vulgaris*, Nutmeg natural, Ylang-ylang, Laurel, Savory, and Rosemary EOs were 2.77- (Cade EO) to 0.26-fold (Rosemary EO) more potent against $LOO^{\bullet}$, yet with poorer correlation: $[EC_{50}]$ $LOO^{\bullet}$ = 0.7667 x $[EC_{50}]$ $ABTS^{+\bullet}$ + 0.0632 (r^2 = 0.74).

Antigenotoxic activity *in vitro*

EOs with increasing dose-dependent potency to protect DNA from $ROO^{\bullet}$ and $OH^{\bullet}$

As for $ROO^{\bullet}$, in the concentration of 25 μg/mL, the listed EOs displayed the ROO-RBDs in the range of 0.805 (Eucalyptus EO) to 0.959 (Lavander EO) units, with an average ROO-RBD of 0.860, thus generally exerting lower average protective ability than Q (the average ROO-RBD equal to 0.880). At the same concentration, EOs protected DNA from damage induced by $OH^{\bullet}$ with OH-RBDs in the range of 0.600 (Bergamot EO) to 0.959 (Camphor EO) units, and an average HO-RBD of 0.819, thus being slightly less potent than Q (an average OH-RBD of 0.823). Adding a 2-fold higher concentration of EOs endowed with the ROO-RBDs range of 0.812 (Savory EO) to 0.985 (Tea Tree EO) units, with the average ROO-RBD of 0.880, thus matching the protectivity of Q. As for $OH^{\bullet}$, a rise of DNA protection was evident 0.973 (*Coridotthymus capitatu*s EO). Furthermore, by matching the standard's concentration, EOs behaved just slightly more protectively than Q when considering $ROO^{\bullet}$, with an average ROO-RBD of 0.890 found in the range of 0.828 (Eucalyptus EO) to 0.988 (Lavander EO) units. On the other hand, they were notably more protective against the damage caused by $OH^{\bullet}$, with the HO-RBDs range of 0.735 (Siberian pine EO) to 0.891 (*Coridotthymus capitatu*s EO) units, and an average HO-RBD of 0.878. Somehow expected, with double-folding the standard's concentration, and with a ROO-RBD range of 0.847 (Eucalyptus EO) to 0.997 (Lavander EO), EOs achieved a very good level of protection against $ROO^{\bullet}$, better than Q, with an average ROO-RBD of 0.910. The level of protection was even more emphasized for $OH^{\bullet}$, being neutralized in the HO-RBD range from 0.809 (Palmarosa EO) to 0.974 (Cade EO) and with an average HO-RBD of 0.906 units. As already stated, the maximum protection of the DNA against $ROO^{\bullet}$ was achieved with the EOs concentration of 400 μg/mL, by exerting the ROO-RBDs from 0.860 (Savory EO) to 0.996 (Myrtle EO) units, with the average RBD of 0.996. The same conclusion is valued for the $OH^{\bullet}$, for which the HO-RBD range was from 0.838 (Siberian pine EO) to 0.999 (*Eucalyptus globulus* EO) and the average HO-RBD was 0.937 units.

By analyzing only ROO-RBDs, no clear picture of EOs potency was gained. However, according to the ROO-RBD_{50} values, the most potent protector of DNA was the Ginger EO sample (ROO-RBD_{50} equal to 8.24 μg/mL), whereas the least potent one was Marjoram EO (ROO-RBD_{50} equal to 324.83 μg/mL). Both EOs also notably neutralized the $OH^{\bullet}$ radical with the OH-RBD_{50}s of 56.34 and 60.02 μg/mL, respectively, but were discarded from further considerations due to no potency against single previously investigated species. On the other hand, the most prominent scavenger of $OH^{\bullet}$ was *Coridotthymus capitatu*s, with the HO-RBD_{50} equal to 24.26 μg/mL, thus correlating with the matching potency against 2′-deoxy-D-ribose (EC_{50} = 0.22 μg/mL), but it was surprisingly not so potent against $ROO^{\bullet}$, as judged by the ROO-RBD_{50} equal to 215.90 μg/mL, despite having high potency against $LOO^{\bullet}$ (EC_{50} of 0.232 μg/mL). The least active one was Lavander, characterized by the HO-RBD_{50} of 209.76 μg/mL (at the same time exerting ROO-RBD_{50} of 44.10 μg/mL). However, due to the failure against either M^{n+} (*Coridotthymus capitatu*s and Lavander) or $DPPH^{\bullet}$, $LOO^{\bullet}$, $ABTS^{\bullet+}$, and $OH^{\bullet}$ (Lavander), distinct EOs were discarded from future experiments.

EOs with decreasing dose-dependent potency to protect from ROO• and OH•

While counteracting the ROO•, in the lowest concentration, the listed EOs displayed the ROO-RBDs in the range of 0.843 (Grapefruit EO) to 0.945 (Red thyme EO) units, with the average ROO-RBD of 0.894, thus greatly surpassing the potency of Q. A more profound potency than Q was noticed against OH•, for which the EOs exerted HO-RBDs in the range of 0.843 (Coriander EO) to 0.997 (Red thyme EO) units, with the average HO-RBD of 0.924. Present in 2-fold lower quantity than Q, the EOs potencies against ROO• were found in the ROO-RBDs range of 0.836 (Grapefruit EO) to 0.923 (Red thyme EO) units, and with the descending average ROO-RBD of 0.889, still more potent than Q. As for OH•, EOs' HO-RBDs were in the range of 0.828 (Coriander EO) to 0.961 (Clary sage EO) units, with the average HO-RBD of 0.895. A trend of DNA protection decrease by listed EOs against either of radicals, toward below the potency of Q, continued with reaching the concentration of Q: the ROO-RBDs range has been 0.819 (Grapefruit EO) to 0.916 (Red thyme EO) units, while the average ROO-RBD was 0.874 the HO-RBDs range has been 0.805 (Coriander EO) to 0.938 (Clary sage EO) units, while the average HO-RBD was also 0.874. Surpassing 2-fold the concentration of Q, listed EOs even more severely lost their protective features against either ROO• or OH•, by means of an average ROO-RBD of 0.847 found in the ROO-RBDs range of 0.775 (Grapefruit EO) to 0.902 (Red thyme EO), as well as by virtue of an average HO-RBD of 0.816 found in the HO-RBDs range of 0.743 (Coriander EO) to 0.850 (Black pepper EO). Finally, in the highest of concentrations, the minimum of DNA protection was achieved, for either ROO• or OH•, by exerting the ROO-RBDs from 0.765 (Grapefruit EO) to 0.859 (Chamomile Morocco EO) units, and the average ROO-RBD of 0.821, as well as by displaying HO-RBDs from 0.713 (Coriander EO) to 0.813 (Roman chamomile EO) units, and the average HO-RBD of 0.784.

Within, the most potent preventer of ROO•-induced DNA damage was Grapefruit EO with the $ROO\text{-}RBD_{50}$ equal to 126.74 μg/mL, yet displaying better selectivity for OH•, as judged by the $OH\text{-}RBD_{50}$ of 103.10 μg/mL. Still, distinct EO has been previously only shown as a good chelator of M^{n+} and was thus not considered a candidate for further elaboration. Within a sub-group, the least active ROO• scavenger was Clary sage EO, with the $ROO\text{-}RBD_{50}$ of 176.43 μg/mL, again more selective toward OH•, evident by the $OH\text{-}RBD_{50}$ equal to 168.31 μg/mL. However, Clary sage EO showed virtually no affinity for DPPH•, LOO•, $ABTS^{+\bullet}$, M^{n+}, and OH•. Considering OH•, the most potent sample was Roman chamomile EO, with the $OH\text{-}RBD_{50}$ = 65.90 μg/mL but with the undetermined $ROO\text{-}RBD_{50}$ and only moderately active against M^{n+}. The least active OH• salmon DNA-level scavenger was the aforementioned Clary sage EO.

EOs with increasing and decreasing dose-dependent potency to protect DNA from ROO• and OH•, respectively

EOs labeled as Sage Oil, Garlic, Cade, Cedar leaves, Pine silvestre natural, Juniper, Birch, Orange sweet, Cajeput, Nutmeg natural, Verbena, Basil, Laurel, *Thymus vulgaris*, and *Origanum hirtum*, protected with more power the DNA from the damage induced by ROO• but were losing gradually the effectiveness against OH• with the increase of concentration applied.

Thus, while restraining the ROO•, the lowest-concentrated samples fitted in the range of ROO-RBDs from 0.788 (Juniper EO) to 0.962 (Birch EO) units, with the average ROO-RBD of 0.877, acting with lower potency than Q. The range of OH-RBDs was from 0.842 (Juniper EO) to 0.986 (White thyme EO) units, with an average OH-RBD of 0.910, for which the EOs were averagely more potent than Q. The affinity toward ROO• increased doubling the concentration of EOs, with ROO-RBD being on average 0.888 units. While matching Q in terms of concentration, listed EOs continued suppressing the ROO• (ROO-RBDs from 0.809 [Juniper EO] to 0.973 [Laurel EO] units, with the average ROO-RBD of 0.903), on one side, but were further losing the ability to fight the OH• (OH-RBDs form 0.803 [Birch EO] to 0.944 [Cajeput EO] units, with an average OH-RBD of 0.866), on the other. The disparity between the ROO•/OH• affinities enlarged with further concentration increment, having EOs highly potent against the ROO• (ROO-RBDs from 0.821 [Juniper EO] to 0.960 [Orange sweet EO] units, with the average ROO-RBD of 0.924) and weakly potent against the OH• (OH-RBDs form 0.731 [Verbena EO] to 0.944 [Cajeput EO] units, with an average OH-RBD of 0.827). In that sense, the maximal difference in the potency in favor of ROO• came with the maximal concentration of 400 μg/mL, where the 0.953/0.784 ratio was achieved between the average ROO-RBD and OH-RBD values (the ROO-RBDs ranged from 0.872 [Pine silvestre natural EO] to 0.984 [Laurel EO] the OH-RBDs ranged from 0.668 [Juniper EO] to 0.908 [Orange sweet EO]).

Among the subgroup, the Cedar leaves EO neutralized both ROO• and OH• ($ROO\text{-}RBD_{50}$ = 96.44 μg/mL $OH\text{-}RBD_{50}$ = 150.16 μg/mL), not surprising owing to the potency against the LOO• (EC_{50} = 0.600 μg/mL) and OH• while protecting 2'-deoxy-D-ribose (EC_{50} = 0.270 μg/mL). Still, Cedar leaves EO has been able to neutralize DPPH• and $ABTS^{+\bullet}$ but not to chelate M^{n+} as well, and was thus discarded from further consideration. The least potent scavenger of ROO• was *Thymus vulgaris* EO ($ROO\text{-}RBD_{50}$ = 232.47 μg/mL), likewise demonstrating a low affinity for OH• ($OH\text{-}RBD_{50}$ = 200.03 μg/mL), but with no inability to chelate M^{n+}. Regarding the neutralization of OH•, Laurel EO showed some potential with the $OH\text{-}RBD_{50}$ equal to 78.70 μg/mL, but had no satisfactory dose-dependent profile for calculating the $ROO\text{-}RBD_{50}$. A similar scenario was found

for Pine silvestre natural EO as the least active $OH^•$ scavenger ($OH\text{-}RBD_{50}$ = 213.01 μg/mL). The remaining EOs within this subgroup besides some perspective as judged by $ROO\text{-}RBD_{50}$ and $OH\text{-}RBD_{50}$ values, had no potency against M^{n+}, $DPPH^•$, $LOO^•$, $ABTS^{•+}$, and $OH^•$.

EOs with decreasing and increasing dose-dependent potency to protect DNA from $ROO^•$ and $OH^•$, respectively.

The remaining 6 EOs, namely Ylang-ylang, Cardamom, Mandarin, Cedar Fruit, Lemon, and Niaouly, with the increase of concentration, exerted decreased abilities to protect the DNA from the damage induced by $ROO^•$ but showed increasing effectiveness against $OH^•$.

Thus, in the lowest concentration, the listed EOs counteracted the $ROO^•$ by means of the ROO-RBDs in the range of 0.840 (YY EO) to 0.971 (Cedar fruit EO) units, and an average ROO-RBD of 0.899, acting more potent than Q. While considering the protection against $OH^•$, it occurred in between the 0.740 (Cedar fruit EO) and 0.878 (Ylang-ylang EO) μg/mL, characterized by an average of OH-RBD equal to 0.794 μg/mL. A second trial was endowed in the ROO-RBDs range of 0.831 (YY EO) to 0.964 (Lavander EO) units, yielding a descending average ROO-RBD of 0.888, during which EOs were still more potent than Q. Oppositely, the range of OH-RBDs was 0.721 (YY EO) to 0.895 (Lavander EO), with increasing average OH-RBD equal to 0.800 μg/mL. A similar trend continued during the third trial, as well, with the ROO-RBDs range of 0.811 (YY EO) to 0.896 (Niaouly EO) units, and the average ROO-RBD of 0.856, after which EOs lost their potency related to Q. As for OH-RBDs, they were in between the 0.732 (Niaouly EO) and 0.908 (YY EO), while the average value was 0.827, just slightly better than Q's. In the concentration of 200 μg/mL, listed EOs continued to lose the effectiveness against $ROO^•$, as judged by an average ROO-RBD of 0.836 extracted from the range of 0.809 (YY EO) to 0.873 (Cedar fruit EO). In the same concentration, EOs on average overpowered Q with an average OH-RBD of 0.890, found between 0.843 (Mandarin EO) and 0.931 (YY EO) RBD units. As indicated, the lowest level of DNA protection from $ROO^•$ by discussed EOs was displayed in their highest concentration of 400 μg/mL, where ROO-RBDs ranged from 0.801 (Niaouly EO) to 0.913 (Cardamom EO) units, with an average of 0.839. On the other hand, the highest concentration applied resulted in the highest level of DNA protection from $OH^•$ by discussed EOs, where HO-RBDs ranged from 0.879 (Mandarin EO) to 0.968 (Lemon EO) units, with an average of 0.940.

Within the cluster of EOs, the highest potency to neutralize the $ROO^•$ was exerted by Cedar fruit EO, as judged by the $ROO\text{-}RBD_{50}$ equal to 54.50 μg/mL. The particular oil was also acceptably active against the $OH^•$ ($OH\text{-}RBD_{50}$ of 95.01 μg/mL), but was in the initial testing only potent to chelate the M^{n+}. The worst protector of DNA against $ROO^•$ was Mandarin EO ($ROO\text{-}RBD_{50}$ = 197.55 μg/mL), still undetermined by means of potencies $OH\text{-}RBD_{50}$ and M^{n+}, $DPPH^•$, $LOO^•$, $ABTS^{•+}$, and $OH^•$. Of the $OH^•$-active ones at the level of DNA, again the best one was the Cedar fruit EO, whereas the weakest one was the Cardamom EO ($OH\text{-}RBD_{50}$ = 202.89 μg/mL), yet undetermined by means of $ROO\text{-}RBD_{50}$ and neutralization potencies of $DPPH^•$, $LOO^•$, $ABTS^{+•}$, M^{n+}, and $OH^•$. The Lemon EO behaved similarly to Cardamom EO, dough with slightly higher potency toward $OH^•$ ($OH\text{-}RBD_{50}$ = 136.18 μg/mL) and with some potential to chelate M^{n+}. The most interesting oil within the sub-group was YY EO, having $ROO\text{-}RBD_{50}$ of 64.14 μg/mL and $OH\text{-}RBD_{50}$ equal to 64.14 μg/mL (Figure 2), thus being among all the previously discussed sub-groups/extracts, the second one alongside the CCP EOwith exceptional affinity to likewise neutralize $DPPH^•$, $LOO^•$, $ABTS^{+•}$, M^{n+}, and $OH^•$. Therefore, YY EO was thus last selected for the *in vivo* administration.

Liver redox status

The *r*TBARS concentrations

In the lowest of concentration the protective response was less intensive (Table 4: III *vs.* I and V *vs.* I, 74.19 and 81.05% of the *r*TBARS concentration measured in the negative control, III *vs.* II and VI *vs.* II, 41.82 and 45.68% of the *r*TBARS concentration caused by CCl_4). The more intensive protection was in the concentration of 200 mg/kg bwt (Table 4: IV *vs.* I and VII *vs.* I, 33.87 and 37.90% of the *r*TBARS concentration measured in the negative control, IV *vs.* II and VII *vs.* II, 19.00 and 21.36% of the *r*TBARS concentration caused by CCl_4),

The *r*SOD catalytic activities

With such a low quantity of redox stress inducer, both EOs again replied very well even at a minimal dosage (Table 4: III *vs.* I, and VI *vs.* I, 93.93 and 91.65% of the catalytic activity of *r*SOD, respectively III *vs.* II, and VI *vs.* II, 1.79- and 1.75-fold higher catalytic activity of *r*SOD, respectively) and were very efficient in the medium (Table 4: IV *vs.* I and VII *vs.* I, 96.20 and 96.96% of the *r*SOD concentration, IV *vs.* II and VII *vs.* II, 1.84- and 1.85-fold higher *r*SOD).

The *r*CAT catalytic activities

The lowest-concentrated formulations of either YY EO or CCP EO did not fully manage to compensate for the harmful effect of CCl_4 but were still able to able to increase the catalytic activity of *r*CAT, thus giving the signs of liver recovery (Table 4: III *vs.* I and VI *vs.* I, 65.30 and 60.48% of the *r*CAT basal catalytic activity III *vs.* II and VI *vs.* II, 1.12 and 1.05-fold higher catalytic activity of *r*CAT than caused by CCl_4). Seemingly insignificant, these results agreed with the prediction for YY EO and CCP EO obtained while neutralizing the $ABTS^{+\bullet}$ (Table 1) that even the lowest concentrations of distinct EOs could be beneficial for CAT. The 200-folding YY EO and CCP EO dosages were more efficient (Table 4: IV *vs.* I and VI *vs.* I, 93.69 and 92.46% of the *r*CAT basal catalytic activity, IV *vs.* II and VI vs. II, 1.61 and 1.59-fold higher *r*CAT catalytic activity).

The *r*GSH concentrations

The counteraction with either YY EO or CCP EO resulted in just mild hepatoprotection (Table 4: III *vs.* I and VI *vs.* I, 48.88 and 53.19% of the basal *r*GSH concentration III *vs.* II and VI *vs.* II, 1.08- and 1.17-fold higher *r*GSH concentration than caused by CCl_4, respectively), but hepatoprotective features against CCl_4 became more expressed with the increase of concentrations (200 and 400 mg/kg, Table 4: IV *vs.* I and VII *vs.* I, 52.61 and 62.84% of the basal *r*GSH concentration IV *vs.* II and VII *vs.* II, 1.16- and 1.17-fold higher *r*GSH concentration than caused by CCl_4 V *vs.* I and VIII *vs.* I, 62.84 and 90.94% of the basal *r*GSH, V *vs.* II and VIII *vs.* II, 1.17- and 2.01-fold higher *r*GSH concentration than caused by CCl_4).

The hepatocytes toxicity status

The *r*AST and *r*ALT catalytic activities

It was interesting to observe that both *r*AST and *r*ALT were the sensitive indicators of YY EO and CCP EO as xenobiotics, given that the lowest of EOs concentrations provoked the strongest cellular responses (Table 5: III *vs.* I and VI *vs.* I, 7.64- and 2.61-fold over the basal *r*AST catalytic activity III *vs.* II and VI *vs.* II, 64.14 and 34.23% of the catalytic activity of *r*AST caused by CCl_4 III *vs.* I and VI *vs.* I, 2.49- and 2.20-fold over the basal *r*ALT catalytic activity III *vs.* II and VI *vs.* II, 43.63 and 38.52% of the catalytic activity of *r*ALT caused by CCl_4). The lowest-concentration YY EO administration caused a more severe cellular response than CCP EO. Hepatocytes, however, adapted to the remedies with the concentrations increase, evident by the gradual decrease of enzymes' catalytic activities (Table 5: IV *vs.* I, and VII *vs.* I, 3.09- and 2.52-fold over the basal *r*AST catalytic activity IV *vs.* II and VII *vs.* II, 40.50 and 33.02% of the catalytic activity of *r*AST caused by CCl_4 IV *vs.* I and VII *vs.* I, 1.14- and 1.42-fold over the basal *r*ALT catalytic activity IV *vs.* II and VII *vs.* II, 19.90 and 24.74% of the catalytic activity of *r*ALT caused by CCl_4).

The *r*ALP and γ-GT catalytic activities

Hence, considering the catalytic activities of *r*ALP, YY EO was more effective than CCP EO while lowering them in the lowest (Table 5: III *vs.* I and VI *vs.* I, 1.72- and 1.96-fold over the basal *r*ALP catalytic activity III *vs.* II and VI *vs.* II, 84.01 and 95.55% of the catalytic activity of *r*ALP caused by CCl_4) and medium concentration (III *vs.* I and VI *vs.* I, 1.48- and 1.82-fold over the basal *r*ALP catalytic activity III *vs.* II and VI *vs.* II, 71.94 and 88.75% of the catalytic activity of *r*ALP caused by CCl_4) than for CCP EO.

As for γ-GT, it was CCP EO that lowered the catalytic activity of the enzyme with more potency than YY EO in all concentrations (Table 5: III *vs.* I, and VI *vs.* I, 3.06- and 2.81-fold over the basal γ-GT catalytic activity III *vs.* II and VI *vs.* II, 61.91 and 57.01% of the catalytic activity of γ-GT caused by CCl_4 IV *vs.* I, and VII *vs.* I, 1.93- and 1.83-fold over the basal γ-GT catalytic activity IV *vs.* II and VII *vs.* II, 39.00 and 37.00 % of the catalytic activity of γ-GT caused by CCl_4 V *vs.* I, and VIII *vs.* I, 1.32- and 1.21-fold over the basal γ-GT catalytic activity V *vs.* II and VIII *vs.* II, 26.78 and 24.55% of the catalytic activity of γ-GT caused by CCl_4) for which some organ selectivity was confirmed.

Kidneys redox status

The *r*TBARS concentrations

The lowest concentrations of YY EO and CCP EO were not so efficient in counteracting CCl_4 (Table 6: III *vs.* I, and VI *vs.* I, 8.95- and 6.44-fold higher than the basal *r*TBARS value III *vs.* II, and VI *vs.* II, 2.21- and 1.59 higher than the *r*TBARS value induced by CCl_4, respectively), whereas in the medium concentration, CCP EO was more potent, even to the certain point restoring the kidneys membrane (Table 6: IV *vs.* I, and VII *vs.* I, 8.65- and 6.35-fold higher than the basal *r*TBARS value IV *vs.* II, and VII *vs.* II, 2.14-fold higher than the *r*TBARS value induced by CCl_4 and 99.00% of *r*TBARS concentration induced by CCl_4, respectively).

The *r*SOD catalytic activities

EOs were of notable efficiency while restoring the catalytic activity of *r*SOD, which has not been too high after the application of CCl_4, only 1.20-fold increased related to the basal value (Table 6: group II vs. group I), this time indirectly pointing to the high-intensity lipid peroxidation of nephrons' cell membrane. Thus, the lowest and medium dosages of either YY EO or CCP EO were considerably efficient (Table 6: III *vs.* I, and VI *vs.* I, 83.37 and 80.63% of the catalytic activity of *r*SOD, respectively III *vs.* II, and VI *vs.* II, 1.37- and 1.32-fold higher catalytic activity of *r*SOD IV *vs.* I and VII *vs.* I, 86.89 and 84.54% of the *r*SOD concentration, IV *vs.* II and VII *vs.* II, 1.42- and 1.38-fold higher *r*SOD).

The *r*CAT catalytic activities

An excess of *r*H_2O_2 in kidneys, caused by CCl_4 and confirmed by a significant decrease in the catalytic activity of *r*CAT (Table 6: II *vs.* I, 30.76% of the *r*CAT catalytic activity), seemed to be efficiently neutralized by either of EOs assessed. Even in the lowest of concentrations, the *r*CAT's catalytic activity was restored in a great manner (Table 6: III *vs.* I and VI *vs.* I, 86.10 and 89.10% of the *r*CAT basal catalytic activity III *vs.* II and VI *vs.* II, 2.80 and 2.90-fold higher catalytic activity of *r*CAT than caused by CCl_4). Starting from 200 mg/kg bwt, YY EO was slightly more potent than CCP EO (Table 6: IV *vs.* I and VI *vs.* I, 97.36 and 96.12% of the *r*CAT basal catalytic activity, IV *vs.* II and VI vs. II, 3.16 and 3.12-fold higher *r*CAT catalytic activity).

The *r*GSH concentration

An evaluation of EOs impact on kidneys' *r*GSH, revealed that they have been capable of restoring the marker's concentration downregulated by CCl_4 (Table 6: II *vs.* I, 49.70% of basal concentration. In the lowest of concentrations, both EOs restored in more than 50% (Table 6: III *vs.* I and VI *vs.* I, 62.25 and 53.56% of the basal *r*GSH concentration III *vs.* II and VI *vs.* II, 1.25- and 1.08-fold higher *r*GSH concentration than caused by CCl_4, respectively), more than 70% in the mid concentration (Table 6: IV *vs.* I and VII *vs.* I, 80.29 and 70.31% of the basal *r*GSH concentration IV *vs.* II and VII *vs.* II, 1.62- and 1.41-fold higher *r*GSH concentration than caused by CCl_4) whereas the maximal dosage restored it almost entirely (Table 6: V *vs.* I and VIII *vs.* I, 93.94 and 99.09% of the basal V *vs.* II and VIII *vs.* II, 1.17- and 2.01-fold higher *r*GSH concentration than caused by CCl_4).

Chronic kidney disease markers

The *r*XO catalytic activities

Still, the *r*XO's catalytic activity was well downregulated by either YY EO or Ceylon cinnamon peel EO, with lower intensities in the 1 and 200 mg/kg btw concentrations (Table 6: III *vs.* I, and VI *vs.* I, 2.07- and 1.82-fold higher than the basal catalytic activity of *r*XO III *vs.* II, and VI *vs.* II, 75.26 and 66.05% of the *r*XO catalytic activity induced by CCl_4 IV *vs.* I, and VII *vs.* I, 1.69- and 1.61-fold higher than the basal catalytic activity of *r*XO IV *vs.* II, and VII *vs.* II, 61.13 and 58.31% of the *r*XO catalytic activity induced by CCl_4 respectively), but with the high intensity in 400 mg/kg bwt concentrations (Table 6: V *vs.* I, and VIII *vs.* I, 1.18- and 1.40-fold higher than the basal catalytic activity of *r*XO V *vs.* II, and VIII *vs.* II, 42.71 and 50.61% of the *r*XO catalytic activity induced by CCl_4).

The *r*XO catalytic activities

The lowest and mid concentrations of either YY EO or CCP EO managed to just alleviate the damage caused by CCl_4 (Table 7: III *vs.* I and VI *vs.* I, 3.30- and 2.54-fold higher *r*NOX catalytic activity than the basal value III *vs.* II and VI *vs.* II, 89.88 and 69.26% *r*NOX catalytic activity caused by CCl_4 IV *vs.* I and VII *vs.* I, 1.51- and 1.42-fold higher *r*NOX catalytic activity than the basal value IV *vs.* II and VII *vs.* II, 41.05 and 38.72% *r*NOX catalytic activity caused by CCl_4), while homeostasis was nearly restored with the administration of the highest doses (Table 7: V *vs.* I and VIII *vs.* I, 1.30- and 1.19-fold higher *r*NOX catalytic activity than the basal value III *vs.* II and VI *vs.* II, 35.41 and 32.30% *r*NOX catalytic activity caused by CCl_4).

The *r*NO concentrations

Still, both YY EO and CCP EOas supplements managed to restore the *r*NO concentration in great manner even in lowest and medium concentrations administered (Table 7: III *vs.* I and VI *vs.* I, 69.49 and 54.43% of the basal *r*NO concentration III *vs.* II and VI *vs.* II, 1.81- and 1.42-fold higher *r*NO concentration than caused by CCl_4 IV *vs.* I and VII *vs.* I, 92.80 and 70.18% of the basal *r*NO concentration IV *vs.* II and VII *vs.* II, 2.48- and 1.83-fold higher *r*NO concentration than caused by CCl_4).

The GPx catalytic activities

As expected, both EOs managed to gradually restore *r*GPx's catalytic activity with the rise of concentration (Table 7: III *vs*. I and VI *vs*. I, 60.00 and 72.00% of the basal *r*GPx catalytic activity III *vs*. II and VI *vs*. II, 1.25- and 1.50-fold higher *r*GPx catalytic activity than caused by CCl_4 IV *vs*. I and VII *vs*. I, 80.00 and 84.00% of the basal *r*GPx catalytic activity IV *vs*. II and VII *vs*. II, 1.67- and 1.75-fold higher *r*GPx catalytic activity than caused by CCl_4 V *vs*. I and VIII *vs*. I, 96.00 and 88.00% of the basal *r*GPx, V *vs*. II and VIII *vs*. II, 1.17- and 2.01-fold higher *r*GPx catalytic activity than caused by CCl_4), where YY EO acted better.

EOs antigenotoxic activity *in vivo*

Antigenotoxicity in liver

The counter-treatment with 1 mg/kg bwt of Ylang-ylang EO significantly reduced the liver DNA damage caused by CCl_4, as verified by 2.72-fold higher total comet score (TCS) value related to negative control (Table 8, III *vs*. I), 48.45% of the TCS found for CCl_4 (Table 8, III *vs*. II), and the percentage reduction level (%R) of 62.7%. At the same concentration, CCP EOwas slightly more efficient (Table 8, VI *vs*. I, 2.51-fold higher TS III *vs*. II, 44.57% of the TCS found for CCl_4, and the %R of 67.4%. The 200-fold higher concentration of YY EO reduced the level of CCl_4-induced DNA damage as associated with the 2.17-fold higher TCS than found for the negative control (Table 8, IV *vs.* I), 38.62% of the TS of CCl_4 (Table 8, IV *vs.* II), and a %R of 74.7%, whereas the comparable concentration of CCP EO yielded the TCS 1.9-fold higher than in the negative control (Table 8, VII *vs.* I) and 33.76% of the value found for CCl_4, still inducing better %R of 80.60%.

Antigenotoxicity in kidneys

The potential protective activity of selected EOs against the CCl_4-induced DNA damage was in the end assessed in the kidney cells of albino Wistar rats (Table 9) and again compared to either negative control group, in which most of the comets showed no DNA damage (type T_0) and a few of them indicated very low damage (type T_1) (Table 9, I), or CCl_4 alone, which significantly increased a TSC in kidney cells as compared with the CCl_4-free group (Table 9, II *vs.* I), all because of large-damage comets (*viz.* types T_3 and T_4) presence.

Canceling out the CCl_4's harmful effect on kidneys' DNA by means of YY EO application at the lowest dose was evident upon observing the significant reduction in the DNA damage as indicated in the TCS value 2.50-fold higher than in the hazardous agent-free group (Table 9, III *vs.* I), 39.37% of the TSC value of CCl_4 (Table 9, III *vs.* II), and a %R of 71.9%. The matching action of CCP EO endowed with the 2.01-fold higher TCS level than measured in olive oil sample (Table 9, VI *vs.* I), 31.65% of TSC compared to the single treatment of CCl_4 (Table 9, VI *vs.* II), and %R of 81.1%, again with better potency than YY EO in the lowest concentration. Further, highly emphasized antagonistic features of YY EO related to CCl_4 were noted after being co-administered at 200 mg/kg bwt, causing a 1.95-fold higher TCS than the basal value (Table 9, IV *vs.* I), decrease in the TCS from 138.7 (CCl_4 alone) to 42.5 (Table 9, IV *vs.* II) and a %R of 82.3%. The equivalent dosage of CCP EO produced the 1.54-fold TCS increment related to native value (Table 9, VII *vs.* I), a reduced the value of TCS by 4.13-fold than for CCl_4 (Table 9, II *vs.* VII), and a %R of 89.9%, retaining its supremacy compared to YY EO. Differently to liver, CCP EOwas more potent than YY EO in the highest of concentration. Still, both EOs even more pronouncedly decrease protected the DNA, lowering the TCSs to the 1.69 and 1.31-fold higher values than basal ones (Table 9, V *vs.* I and VIII *vs.* I), to the 26.53 and 20.55% of TCSs observed for CCl_4 (Table 9, V *vs.* II and VIII *vs.* II), and causing the %Rs equal to 87.2 and 94.4%, respectively.

Canceling out the CCl_4's harmful effect on kidneys' DNA by means of YY EO application at the lowest dose was evident upon observing the significant reduction in the DNA damage as indicated in the TCS value 2.50-fold higher than in the hazardous agent-free group (Table 9, III *vs.* I), 39.37% of the TSC value of CCl_4 (Table 9, III *vs.* II), and a %R of 71.9%. The matching action of CCP EOendowed with the 2.01-fold higher TCS level than measured in olive oil sample (Table 9, VI *vs.* I), 31.65% of TSC compared to the single treatment of CCl_4 (Table 9, VI *vs.* II), and %R of 81.1%, again with better potency than YY EO in the lowest concentration. Further, highly emphasized antagonistic features of YY EO related to CCl_4 were noted after being co-administered at 200 mg/kg bwt, causing a 1.95-fold higher TCS than the basal value (Table 9, IV *vs.* I), decrease in the TCS from 138.7 (CCl_4 alone) to 42.5 (Table 9, IV *vs.* II) and a %R of 82.3%. The equivalent dosage of CCP EOproduced the 1.54-fold TCS increment related to native value (Table 9, VII *vs.* I), a reduced the value of TCS by 4.13-fold than for CCl_4 (Table 7, II *vs.* VII), and a %R of 89.9%, retaining its supremacy compared to YY EO.
